# Gastrointestinal Microbiome and Multiple Health Outcomes: Umbrella Review

**DOI:** 10.3390/nu14183726

**Published:** 2022-09-09

**Authors:** Chengting Chang, Xingzhu Yuan, Xingxia Zhang, Xinrong Chen, Ka Li

**Affiliations:** 1 West China School of Nursing, Sichuan University/West China Hospital, Sichuan University, 37 Guo Xue Rd., Chengdu 610041, China; 2 Department of Organization, West China Hospital, Sichuan University, 37 Guo Xue Rd., Chengdu 610041, China

**Keywords:** gastrointestinal microbiome, gut microbiota, gut flora, probiotics, prebiotic, synbiotics, health, meta analysis, systematic reviews

## Abstract

In recent years, there has been growing concern about the impact of the gastrointestinal microbiome on human health outcomes. To clarify the evidence for a link between the gastrointestinal microbiome and a variety of health outcomes in humans, we conducted an all-encompassing review of meta-analyses and systematic reviews that included 195 meta-analyses containing 950 unique health outcomes. The gastrointestinal microbiome is related to mortality, gastrointestinal disease, immune and metabolic outcomes, neurological and psychiatric outcomes, maternal and infant outcomes, and other outcomes. Existing interventions for intestinal microbiota (such as probiotics, fecal microbiota transplant, etc.) are generally safe and beneficial to a variety of human health outcomes, but the quality of evidence is not high, and more detailed and well-designed randomized controlled trials are necessary.

## 1. Introduction

There are hundreds of millions of microorganisms in the human gut [1]. Collectively known as gut flora, the human gastrointestinal microbiota is more than 10 times the number of cells in the human body, providing metabolic, immune, and protective functions and playing a vital role in human health [2,3,4,5,6]. The gut microbiota produces bioactive metabolites that influence many aspects of host physiology. For example, as a product of intestinal flora, short-chain fatty acids (SCFAs) are playing an increasingly important role. Butyrate can induce the apoptosis of colon cancer cells and activate intestinal gluconeogenesis, which has beneficial effects on glucose and energy homeostasis [7]. Butyrate and propionate appeared to control gut hormones, reducing appetite and food intake in mice [8]. Other specific products of the gut microbiome are directly relevant to human health outcomes. Trimethylamine, for example, is oxidized in the liver to trimethylamine N-oxide, which is positively associated with an increased risk of adverse cardiovascular events [9,10]. The gastrointestinal microbiota is influenced by many factors, including genetics, host physiology (age, disease, stress, etc.), and environmental factors (living conditions, drug use, diet, etc.) [8,11,12,13]. In recent years, the consumption of specific dietary components (such as fiber and prebiotics) and fecal microbiota transplantation have become common ways to regulate the microbiome. In addition, the concept of the brain–gut–microbiota axis has attracted much attention. The central nervous system (CNS) is able to respond directly or indirectly to the microbiota and its metabolites through the network of CNS neurons. Gut microbiota can affect brain function from the bottom up through the microbiota–gut–brain axis, and it can participate in the occurrence and development of psychiatric, neurodevelopmental, age-related, and neurodegenerative disorders. The brain may also affect gut microbiota and its metabolites from the top down through the brain–gut–microbiota axis. The concrete mechanism of two-way communication between the gut and brain is not entirely clear, but communication between the gut microbiome and the brain/central nervous system is closely related to other systems, such as the immune system, the endocrine system, the intestinal barrier and blood–brain barrier, microbial metabolites and gut hormones, as well as the sensory and autonomic nervous systems [14,15,16,17,18].

However, it is important to systematically evaluate and summarize the advanced evidence on the impact of gastrointestinal microbiota on all health outcomes before interventions are undertaken. To date, many epidemiological studies (case controls or cohort) and clinical trials (cross-sectional or randomized control trials) have been widely used to investigate the relationship between gut microbes and a range of health outcomes. However, different studies have come to conflicting conclusions. If carried out and interpreted properly, umbrella reviews can provide evidence of the highest quality. Therefore, we conducted an umbrella review by integrating evidence from multiple meta-analyses to help determine the presence and extent of associations between gastrointestinal microbiota and different health outcomes and to assess any risks that may be associated with changes in gastrointestinal microbiota in existing studies. The research results can provide a basis for policymakers to formulate or update relevant guidelines.

## 2. Methods

### 2.1. Umbrella Review Method and Assessment of Methodological Quality

The umbrella review was carried out as previously published in the relevant literature [19,20]. The AMSTAR [21] and GRADE [22] were used to independently evaluate the methodological quality of the selected meta-analyses or systematic reviews and the quality of the original literature by two investigators (Chengting Chang and Xingzhu Yuan). If there was any inconsistency, a third researcher was consulted. There are 16 items in AMSTAR 2 [21] in total, among which 2, 4, 7, 9, 11, 13, and 15 are the key items. The evaluation results of each item are divided into yes (Y) and part of yes (PY) and no (N). If the evaluation result was not, it was considered to be inconsistent. If there was no or only one noncritical item considered as N, the meta-analysis was rated as highly reliable. If more than one noncritical item is rated as nonconformance, the analysis is considered to have medium credibility. If the analysis does not meet one key item regardless of whether it meets any nonkey item, it is rated as low reliability. If more than one key item does not meet the criteria, no matter whether the nonkey item meets the criteria, the credibility is very low. GRADE [23] has four grades, high, mediate, low, and very low, and it evaluates the quality of evidence of outcome indicators through five degrading factors: the risk of bias, imprecision, inconsistency, indirectness, and magnitude of effect.

### 2.2. Literature Search

We searched meta-analyses of observational or interventional studies, which examined the link between gut microbiota and any health outcomes, published between inception and September 2021 in PubMed, Web of Science, Embase, and the Cochrane Database of Systematic Reviews. We used the following terms/keywords to search: (meta analys* or meta-analys* or systematic review*) and (gastrointestinal microbiome or intestine flora or related free words) using truncated terms for all fields, following the SIGN guidance recommended search terms for systematic reviews and meta-analyses [24]. See Supplementary Materials for detailed retrieval strategies. We also conducted a manual search for references to eligible articles.

### 2.3. Eligibility Criteria

Two researchers (Chengting Chang and Xingzhu Yuan) independently screened the titles and abstracts and selected articles for full-text review. They then independently reviewed the full-text articles for eligibility. A third researcher, Xinrong Chen, arbitrated any differences that could not be resolved by consensus.

Meta-analyses and/or systematic reviews assessing the link between the human gut microbiota (containing microbial metabolites) and any health outcomes were included, regardless of whether and for what reason the gut microbiota changed. However, considering that antibiotics may have a direct impact on health outcomes such as inflammatory factors or disease recovery, the article on antibiotics as an intervention was excluded. Participants of any age, gender, and race from any country and setting were allowed to be included, and participants could be healthy, have pre-existing conditions, or be pregnant. We did not use language restrictions, but animal or in vitro experiments were excluded. There was no limit to the types of meta-analyses but the network meta-analysis, which means observational (cohort, case-control, and cross-sectional studies with binary outcomes) and interventional studies (randomized controlled trials) can be included. However, at least one of the relative risks, odds ratios, relative rates, hazard ratios, standardized mean difference, weighted mean difference, mean difference, risk difference, or median difference should be reported. If there was more than one meta-analysis and/or systematic review of the same research question, we included the most recent study with the largest number of studies and participants. If an article conducted a separate meta-analysis of more than one health outcome, we included each one separately.

### 2.4. Data Extraction

Two investigators (Chengting Chang and Xingzhu Yuan) independently extracted the following information from the included literature: the first author, year of publication, populations, number of studies, number of cases/control or total participants, study design (cohort, case-control, randomized controlled trial (RCT)), intervention duration or length of follow-up, outcome(s) of interest (multiple health outcomes), outcome comparison (e.g., highest versus lowest/none), meta-analysis metric (e.g., odds ratio), estimated summary effect with the 95% confidence intervals (95% CIs), type of effect model (fixed or random), heterogeneity and publication bias. Any discrepancies in the data extracted by the two researchers were resolved by consensus.

### 2.5. Data Analysis

After incorporating appropriate systematic reviews and meta-analyses, we added major trials missing from the largest or the most recent meta-analysis. The estimated summary effect and its 95% confidence interval for each included meta-analysis were then extracted. We used the I2 metric [25] (0% to 25%: might not be necessary; 25% to 50%: might be represented as moderate heterogeneity; 50% to 75%: might be shown as substantial heterogeneity; 75% to 100%: considerable heterogeneity). Heterogeneity was assessed using *p* values generated by the Egger test [26] (*p* < 0.05) to assess publication bias. We did not reanalyze the other data or primary studies included in the meta-analysis.

## 3. Results

### 3.1. Characteristics of Included Articles

**Figure 1:**
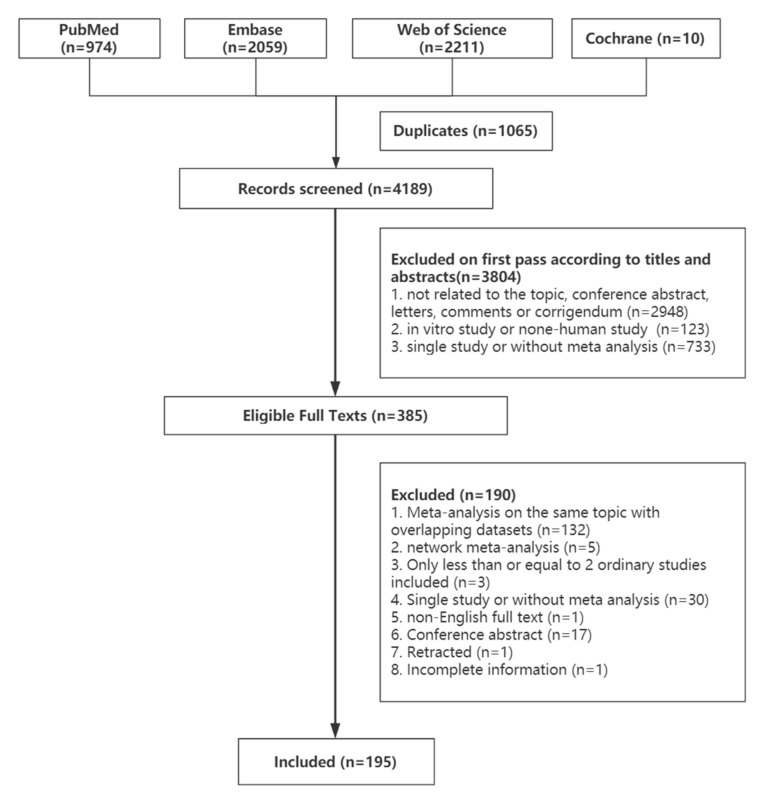
Flowchart of the selection of studies for inclusion in the umbrella review on gastrointestinal microbiome and multiple health outcomes.

**Figure 2:**
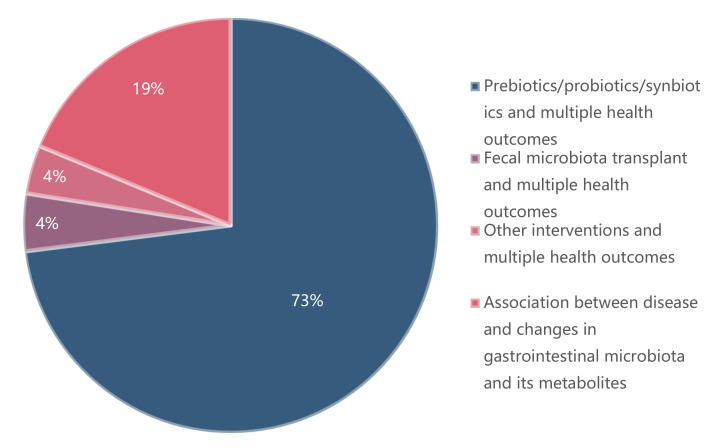
Map of outcomes related to the gastrointestinal microbiome.

**Figure 3:**
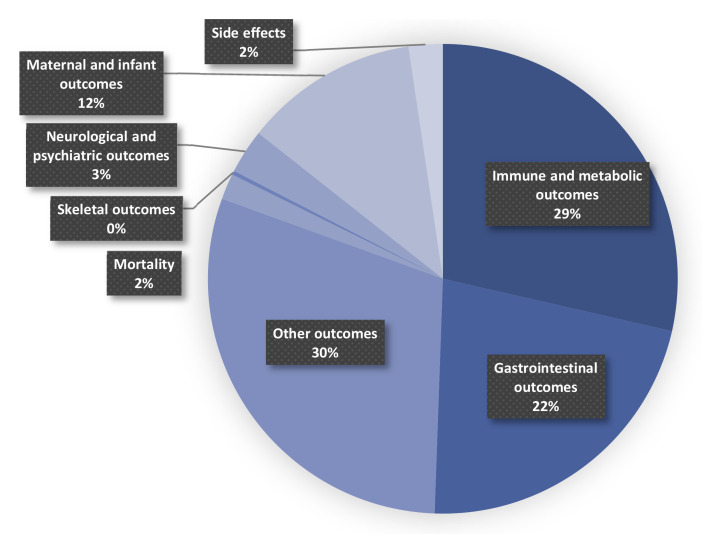
Map of outcomes related to prebiotics/probiotics/synbiotics.

Figure 1 shows the process of the systematic search and selection of eligible studies. A total of 5254 records were retrieved, 385 of which were eligible for the stage of full-text review. Finally, 195 recent meta-analyses spanning 2009–2021 with 970 unique results were included in our umbrella review (Figure 2). Outcomes related to prebiotics/probiotics/synbiotics are shown in Figure 3.

The number of meta-analyses of single outcomes ranged from 3 to 54. The detailed associations between prebiotics/probiotics/synbiotics and multiple health outcomes are shown in Supplementary Table S1. The relationship between fecal microbiota transplantation and multiple health outcomes is presented in Supplementary Table S9. Supplementary Table S10 shows other outcomes related to the gastrointestinal microbiome. The associations between disease and changes in gastrointestinal microbiota and its metabolites are shown in Supplementary Table S11.

### 3.2. Prebiotics/Probiotics/Synbiotics and Multiple Health Outcomes

#### 3.2.1. Mortality

In very preterm (28–32 week of gestation), preterm infants (<36 or 37 weeks), low birth weight infants (<2500 g), and very low birth weight (<1500 g) infants, prebiotic/probiotic use is associated with reduced mortality [27,28,29,30,31,32,33]. However, in extremely preterm (<28 weeks’ gestation) or extremely low birth weight (<1000 g) infants, surgical patients and people with any grade of acute or chronic hepatic encephalopathy, prebiotic/probiotic use has not been associated with reduced mortality [28,34,35]. Additionally, there was no association between the use of a prebiotic, probiotic, or synbiotic agent and the improvement of the death rate in patients who underwent abdominal surgery [36]. See Supplement Table S2 for a more detailed summary.

#### 3.2.2. Immune and Metabolic Outcomes

A number of meta-analyses have proven that related indicators of liver function (e.g., bilirubin, total cholesterol, low-density lipoprotein cholesterol (LDL-c), high-density lipoprotein cholesterol (HDL-c), triglycerides, etc.) were associated with prebiotics/probiotics/synbiotics [37,38,39,40,41,42,43]. However, the association was uncertain for patients with liver disease, kidney disease or metabolic syndrome [42,44,45,46].

In addition, immune-related indicators (such as immunoglobulin A, immunoglobulin G, immunoglobulin M, C-reactive protein (CRP), tumor necrosis factor α, etc.) were related to prebiotics/probiotics/synbiotics [47,48,49,50], but in patients with liver disease or rheumatoid disease or human immunodeficiency virus (HIV), adult athletes, kidney disease patients, healthy elderly individuals aged > 60 years, or obese adults (age ≥ 18 years) who had undergone bariatric surgery, correlations are no longer proven [42,44,51,52,53,54,55,56].

Evidence has been shown to link blood sugar control (such as fasting plasma glucose, glucose, fasting insulin, glycated hemoglobin, etc.) [39,40,44,45,48,57,58,59] and enzymes (lidiamine oxidase, gamma-glutamyl transpeptidase, etc.) [41,42,47] to prebiotics/probiotics/synbiotics.

Protein results (albumin, prealbumin, total protein, hemoglobin, etc.) were associated with prebiotics/probiotics/synbiotics [47] but not in adult chronic kidney disease [53]. Meanwhile, prebiotics/probiotics/synbiotics were related to total antioxidant capacity [39,49], nitric oxide (NO) [49], total glutathione [39,49], plasma ammonia [34,60], malondialdehyde [49,61], serum calcium levels, parathyroid hormone, urinary calcium [62], and the rise of butyrate and total SCFAs [35].

However, there was no evidence of the relationship between prebiotics/probiotics/synbiotics and urea, uric acid, and creatinine in adult chronic kidney disease [53,63,64]. See Supplementary Table S3 for a more detailed summary.

#### 3.2.3. Gastrointestinal Disease

Prebiotics/probiotics/synbiotics are correlated with gastrointestinal symptoms and functional level. Prebiotics/probiotics/synbiotics have been linked to a lower risk of diarrhea [47,65,66,67,68,69,70,71,72,73,74,75] but not in adults who received mechanical ventilation (MV) therapy in hospitals [65] and healthy infants or children, aged 0–18 years [76]. In all patients who met the criteria of severe stroke, early enteral nutrition combined with probiotics was associated with many improvements in gastrointestinal function [47]. The use of combination probiotics is associated with the improvement of persistence of symptoms and bloating scores but not flatulence scores in adults (participants aged > 16 years) [77]. However, regarding abdominal pain scores in patients with irritable bowel syndrome, the association with prebiotics/probiotics/synbiotics is still controversial [78,79,80,81]. Whether probiotics are related to stool frequency and consistency varies with the disease and the population [33,80,82,83,84,85]. In addition, the overall symptom response to treatment, integrative symptom score, severity of abdominal pain, bloating, and flatulence in adult patients ≥ 18 and ≤ 64 years with irritable bowel syndrome or other functional bowel disorders were irrelevant to prebiotics/probiotics/synbiotics [86].

There is also evidence of an association between a positive stool *Clostridium difficile* cytotoxin assay or culture [68,70], a decrease in *Eubacterium rectale* or *Clostridium coccoides* [87], the elevation of beneficial bacteria such as *bifidobacterial* [88], and prebiotics/probiotics/synbiotics. Additionally, the eradication of *Helicobacter pylori (H. pylori)* in *H. pylori*-infected patients was related to probiotics [89]. In adults who received mechanical ventilation therapy in hospitals, oropharyngeal colonization, gastric colonization, and the rate of Gram-negative bacterial positivity were associated with prebiotics/probiotics/synbiotics, while a positive rate of Gram-positive bacterial culture and positive fungal culture rates were irrelevant [65].

The reduction in colon endoscopic scores in children or adults with ulcerative colitis was also related to prebiotics/probiotics/synbiotics [90,91] but not only using *Escherichia coli Nissle (EcN) 1917* [92]. For people of any age with ulcerative colitis in remission, clinical relapse and maintenance of clinical remission were not associated with probiotics [93]. Prebiotics/probiotics/synbiotics were related to clinical remission and clinical response in adults or children with active ulcerative colitis [94,95,96,97] and inducing/maintaining inflammatory bowel disease (IBD) remission [98], but preventing relapse is irrelevant [97,99]. See Supplementary Table S4 for a more detailed summary.

#### 3.2.4. Neurological and Psychiatric Outcomes

Probiotics are associated with cognitive improvement in adults [100] but not with cognitive function in dementia patients [101]. However, when focusing on patients with a diagnosis of Alzheimer’s disease (AD) or mild cognitive impairment (MCI), a correlation between probiotics and cognitive promotion has been shown [49].

Probiotics are associated with adult healthy volunteers’ preclinical psychological symptoms of anxiety, depression, and stress [102] but not the symptoms of depressive symptoms [103]. For the general population or clinical population, patients with major depressive disorder or other clinical diagnosis populations, probiotics are related to the improvement of depressive symptoms [103]. Probiotics did not show a correlation with schizophrenia symptoms in patients with at least moderately severe psychotic symptoms aged 18–65 years [104]. Probiotics were associated with a reduction in stress levels and stress-related subthreshold anxiety/depression levels in healthy people [105]. When probiotics were associated with the improvement of depression and anxiety in patients with depression or anxiety, prebiotics and anxiety in adult patients with 18 and 64-year irritable bowel syndrome or other functional bowel disorders (FBDs) showed no correlation [86]. Additionally, depression and anxiety in patients with depression or anxiety did not show any relation to prebiotics [106]. See Supplementary Table S5 for a more detailed summary.

#### 3.2.5. Maternal and Infant Outcomes

Prebiotics/probiotics/synbiotics were associated with the duration of crying in infants [107,108,109]. The risk of necrotizing enterocolitis (before hospital discharge), invasive infection (before hospital discharge) in very preterm (<32 weeks’ gestation) or very low birth weight (<1500 g), but not in infants extremely preterm (<28 weeks’ gestation) or extremely low birth weight (<1000 g) infants, was related to prebiotics/probiotics/synbiotics [28]. The duration of birth hospitalization in very preterm (<32 weeks’ gestation) or very low birth weight (<1500 g) was also reduced [28]. Prebiotics/probiotics/synbiotics were associated with preventing necrotizing enterocolitis in preterm neonates [29,30,31,32], decreasing the death rate in preterm infants <37 weeks and/or birth weight < 2500 g [30,31,32], and reducing total cholesterol and triglycerides in pregnant women [110]. A mixture of Lactobacillus and Bifidobacterium was associated with the risk of eczema in children [111], but Lactobacillus rhamnosus GG is irrelevant [112].

The occurrence of new cases of colic [107] in infants, the risk of neurodevelopmental impairment, cerebral palsy, visual impairment, hearing impairment in very preterm (<32 weeks’ gestation) or very low birth weight (<1500 g) [28], sepsis, Hirschsprung-associated enterocolitis (HAEC), and age reaching full feeds in preterm infants [29,31,32,113] have not been found to be related to prebiotics/probiotics/synbiotics. Mean cognitive and motor scores, the risk of cognitive impairment, the risk of motor impairment, the risk of neurodevelopment impairment (NDI), the risk of cerebral palsy, and the risk of hearing impairment in children under 5 years of age in preterm infants (<37 weeks gestation and/or birth weight < 1500 g) [114] have not been proven to be related to prebiotics/probiotics/synbiotics use. Additionally, preterm birth < 37 weeks’ gestation [115], newborn birth weight [116], and HDL-C and LDL-C in pregnant women [110] were irrelevant to prebiotics/probiotics/synbiotics.

It is contradictory whether prebiotics/probiotics/synbiotics are related to preventing gestational diabetes and some related infant outcomes 105], [110 in pregnant women not previously diagnosed with diabetes mellitus. However, in adult pregnant women regardless of weight status (normal, overweight, or obese), who were diagnosed with gestational diabetes mellitus (GDM) according to the oral glucose tolerance test and were not on any hypoglycemic agents, they were related to blood sugar control in pregnant women and some related infant outcomes [117].

There is no evidence that prebiotics/probiotics/synbiotics are associated with adverse effects including parental depression and mental illness, choking, bacterial infection, or apparent life-threatening/serious events (dichotomous outcome) in infants [107]. See Supplementary Table S6 for a more detailed summary.

#### 3.2.6. Other Outcomes

In participants taking antibiotics, there is no evidence to prove that the detection of *C. difficile* in stool [67] and length of hospital stay [67] are related to probiotics. Overall, prebiotics/probiotics/synbiotics were associated with a reduction in the incidence of infection [36,47,74,87,118,119,120,121], the enteral nutrition target time [47], and the length of hospital stay [33,35,47,119,120]. Parts of the outcomes of respiratory tract infections in healthy children (from birth to 18 years) were also relevant [122]. Prebiotics/probiotics/synbiotics were related to the improvement of polymorphonuclear phagocytic capacity, natural killer (NK) cell tumoricidal activity, and reduction in No-recovery [34,123].

The association between the risk of atopic and food sensitization and prebiotics/probiotics/synbiotics in children varied according to the time of administration [124]. The risk of developing asthma, allergic rhinitis, wheezing, and positive aeroallergen skin-prick test (SPT) results in healthy children were not associated with prebiotics/probiotics/synbiotics [125]. 

Liver stiffness was measured by the FibroScan in adult patients with nonalcoholic fatty liver disease (NAFLD) or nonalcoholic steatohepatitis (NASH) and BMI in male and female patients of any age who presented at least one of the following: NAFLD, steatosis, liver fibrosis, and steatohepatitis [42,126] were proven to be related to prebiotics/probiotics/synbiotics [44,127].

In surgical patients, prebiotics/probiotics/synbiotics were related to superficial incisional, duration of postoperative pyrexia, fluid diet, hospital stay, and solid diet [35]. There is evidence that prebiotics/probiotics/synbiotics may be useful to improve quality-of-life (QoL) in adults of both sexes and of all ages with irritable bowel syndrome (IBS) [86,128]. In adults who received mechanical ventilation (MV) therapy in hospitals, ventilator-associated pneumonia (VAP) incidence, length of ICU stay, length of hospital stay, days of antibiotics use, the incidence of bacteremia, and rate of multidrug-resistant (MDR) infections were related to prebiotics/probiotics/synbiotics [65]. In addition, improvement in the corresponding score of adults with relapsing–remitting multiple sclerosis [129], physical growth during the first year of life of full-term neonates [130], and reduction in the Severity Scoring of Atopic Dermatitis (SCORAD) values at 8 weeks in patients with atopic dermatitis [131] were associated with prebiotics/probiotics/synbiotics. For the patients with minimal hepatic encephalopathy (MHE), the development of overt HE (week 4 but not 12 weeks), and improvement in MHE (week 12) were also relevant [60].

For adults (≥18 years old) with body mass index (BMI) > 25 kg/m^2^, prebiotics/probiotics/synbiotics were related to waist circumference [132], fat mass, fat percentage, waist-to-hip ratio [48], total abdominal fat area, and subcutaneous abdominal fat area [57]. Probiotic fermented milk products (PFMPs) were associated with body weight, but BMI, waist circumference, and body fat percentage were irrelevant [133]. The meta-analysis did not prove that waist circumference and hip circumference in women with polycystic ovary syndrome (PCOS) are irrelevant to prebiotics/probiotics/synbiotics [40], but the modified Ferriman–Gallway score is relevant [39].

The blood pressure of adults (18 years or older) with overweight or obesity [48], number of pulmonary exacerbations, and forced expiratory volume (FEV)1 (% predicted) in participants who fulfilled consensus diagnostic criteria for cystic fibrosis (CF) were not associated with prebiotics/probiotics/synbiotics [134]. In the participants who were diagnosed with chronic kidney disease (CKD), weight, BMI and estimated glomerular filtration rate preservation [63,64] were irrelevant to prebiotics/probiotics/synbiotics. Additionally, in adult diabetic patients, BMI is irrelevant [135], but blood pressure is relevant [135]. Probiotics had no significant effect on sleep [136]. Weight, BMI and waist circumference in adults with metabolic syndrome were not associated with prebiotics/probiotics/synbiotics [46], while BMI and blood pressure control in hypertension patients were relevant [59]. Whether probiotics are related to the immune response to influenza in adults differs from the virus strain [137,138]. There was no correlation between probiotic use and spinal bone mineral density and total hip bone mineral density of adults [62]. See Supplementary Table S7 for a more detailed summary.

#### 3.2.7. Side Effects

In hospitalized or outpatients (adult or children) taking antibiotics [66,67,69,70,73], patients with ulcerative colitis (UC) [92], people with any grade of acute or chronic hepatic encephalopathy [34], adults with inflammatory bowel disease [86,99] or patients with celiac disease [88] or with functional dyspepsia [139], there is no evidence that prebiotics/probiotics/synbiotics are associated with adverse events [77,138,140]. See Supplementary Table S8 for a more detailed summary.

### 3.3. Fecal Microbiota Transplant and Multiple Health Outcomes

Fecal microbiota transplant (FMT) was related to diarrhea in adult human participants with diagnoses of C. difficile diarrhea aged over 19 years old [141] but not in patients with documented recurrent Clostridium difficile infection [142] and adult patients (≥18 years) with IBS [143]. Clinical remission or response of patients with IBD was associated with FMT [144,145,146,147], but it is still controversial whether endoscopic mission/response in adult subjects with endoscopically and clinically active UC is related to FMT [145,146,148]. In adults diagnosed with metabolic syndrome, FMT was relevant to the increase in HDL cholesterol and LDL cholesterol, the reduction in glycated hemoglobin (HbA1c) and total cholesterol, but not fasting glucose, triglycerides, BMI, weight, and homeostasis model assessment of insulin resistance (HOMA-IR) [149]. In addition, FMT was related to achieving antimicrobial resistance remission in adults (>18 years) with achieving antimicrobial resistance colonization [150]. Regarding the serious adverse events, there is no evidence linking them to FMT [95]. See Supplementary Table S9 for a more detailed summary.

### 3.4. Other Interventions and Multiple Health Outcomes

Chinese herbal compounds, Chinese patent medicine, and single Chinese medical herbs were associated with reductions in fasting blood glucose, HbA1c, 2 h postprandial blood glucose, and HOMA-IR [151] through affecting the gastrointestinal microbiome. Dietary fiber (including fruit and vegetable) was related to the risk of Crohn’s disease (CD) and UC [152] and depression in adults and children [153]. Additionally, the low-FODMAP (fermentable oligo-, di- and monosaccharides, and polyols) diet in adult human subjects with IBS was relevant to the decline in IBS severity [154]. The incidence of gastrointestinal acute graft-versus-host disease but not the incidence of mucositis grade III–IV and overall survival at day + 100 was associated with enteral nutrition with or without the addition of parenteral nutrition [155]. In young children (preferably younger than 5 years of age) with acute diarrhea, postbiotics (bioactive compounds produced during a fermentation process, including microbial cells, cell constituents, and metabolites) that support health and/or wellbeing were related to the duration of diarrhea episodes [156]. In addition, kefir beverages have a certain effect on blood glucose control in type II diabetic patients [157], and starch type 2 in adult CKD patients receiving regular hemodialysis has some benefits [158]. However, treatment of *Helicobacter pylori* infection was not related to the improvement of rosacea-related skin symptoms [159]. See Supplementary Table S10 for a more detailed summary.

### 3.5. Association between Disease and Changes in Gastrointestinal Microbiota and Its Metabolites

In patients with chronic pancreatitis [160], chronic liver disease [161], cirrhosis [162], systemic sclerosis [163], or Parkinson’s disease [164], but not participants with obesity, the risk of small intestinal bacterial overgrowth increased [165]. However, the relationship is still controversial in populations with IBS [166,167,168]. Similarly, whether patients have small intestinal bacterial overgrowth (SIBO) is associated with a decrease in the risk of ascites, minimal hepatic encephalopathy, and spontaneous bacterial peritonitis in patients aged ≥ 18 years patients with cirrhosis and nonalcoholic fatty liver disease [162,169]. The risk of prevalence of SIBO on upper gut aspirate culture, and the prevalence of the positive glucose hydrogen breath test but not the positive lactulose hydrogen breath test is related to whether patients have IBS [166].

Whether adult patients are diagnosed with CKD [170] or stroke [171] is associated with high circulating trimethylamine N-oxide (TMAO) concentrations. Additionally, the increase in TMAO concentrations is related to an increase in all-cause mortality [172], the incidence of major adverse cardio and cerebrovascular events (MACCEs) [173,174], major adverse cardiovascular events (MACEs) [10,172,175], hypertension prevalence [176], diabetes [177], cardiovascular events (CVEs) risk [9], heart failure [178] and CRP concentrations [179], but diastolic blood pressure, HDL-cholesterol, LDL-cholesterol, triglycerides, total cholesterol and BMI are irrelevant [180,181]. *H. pylori* infection is elevated in patients with Guillain–Barre Syndrome or IBD but not IBS [182,183,184]. Similarly, if a patient is infected with *H pylori* infection, NAFLD increases [185].

In children with autism, patients with NAFLD, IBD, or colorectal cancer, some species change [186,187,188,189,190,191]. For example, the bacterial counts of *F. prausnitzii, Clostridium coccoides, Clostridium leptum, Faecalibacterium prausnitzii,* and *Bifidobacterium* decrease in patients with IBD [186]. Some metabolites, such as acetate, valerate, butyrate, and total SCFAs, also change in the population with IBD or IBS [189,192,193].

In addition, the alpha diversity (Simpson index) decreased in men who had sex with men (MSM) or people who were HIV + [194]. Overall, the number of observed species and CHAO1 index, but not the Shannon index and Simpson index, are related to psychiatric diagnosis [195]. However, there is no evidence of the relationship between major depressive disorder and the Shannon index or Simpson index [196]. See Supplementary Table S11 for a more detailed summary.

### 3.6. Heterogeneity of Included Studies

Of all included outcomes, approximately 16.7% had low heterogeneity (I2 < 25%). About 64.9% of the outcomes had moderate heterogeneity (I2 ranging from 25 to 75%); 13.2% of the outcomes were highly heterogeneous (I2 > 75%). However, heterogeneity was not reported in 5.2% of outcomes, and we could not reanalyze it because the information was not available.

### 3.7. Publication Bias of Included Studies

Funnel plots and Egger’s test were used in this umbrella review. No publication bias was found in 20.4% of the studies. Other meta-analyses did not report the results of publication bias. However, in many of the included studies, there was a high probability of unreported publication bias.

### 3.8. GRADE Classification and AMSTAR 2 Score of Included Studies

The studies were classified into four levels, with approximately 4.2% rated high, about 92.7% rated low, and 2.6% rated very low by AMSTAR 2. The reason was that most studies did not report the source of funding for the studies included in the meta-analysis (Item 10). Approximately 25.8% were rated as very low, 38.0% as low, and 36.1% as the medium in terms of GRADE. The detailed results of AMSTAR 2 and GRADE are presented in Supplementary Tables S12 and S13, respectively.

## 4. Discussion

In this review, we identified a total of 195 meta-analyses and 970 unique results. Based on the available evidence, the gastrointestinal microbiome is more often associated with a range of health-related outcomes than with harm. There has been some research into how gastrointestinal microbes affect health outcomes. In addition to acting as a microbial barrier, the gut microbiota also participates in the composition of other intestinal barriers through various pathways, and it jointly plays an important role in the gut. Studies have shown that the intestinal microbiota has a protective effect on host intestinal epithelial cells, thus further strengthening the role of the intestinal mechanical barrier. On the contrary, the intestinal microbial disorder will lead to an increase in intestinal permeability, the damage of tight junction proteins to a certain extent, and the damage to the intestinal mechanical barrier [197].

Probiotics play a positive role in human health mainly by maintaining microbial balance and inhibiting the growth of harmful bacteria or pathogens. Studies have shown that whether probiotics can inhibit the growth of harmful bacteria through competition mainly depends on the ability of probiotic strain combinations to inhibit, displace, or interfere in the process of adhesion of pathogenic strains [198,199,200]. The inhibition of adhesion of probiotics to pathogens is achieved by steric hindrance at the level of intestinal receptors, competitive exclusion of nutrients and mucosal adhesion sites, and promotion of intestinal mucin changes [201,202]. Specifically, probiotics contribute to the maintenance of intestinal barrier function by promoting mucus secretion (a gel layer that provides protection against harmful bacteria or antigens by acting as a lubricant to improve intestinal movement and binding carbohydrates to the surface of epithelial cells) [203,204]. In addition, probiotics have beneficial effects by producing enzymes and beneficial metabolites, facilitating synthesis, and enhancing the absorption of beneficial substances. Another important mechanism by which probiotics participate in the regulation of gut microbiota is through the production of different antibacterial substances such as bacteriocin, SCFAs, and deconjugated bile acid. SCFAs such as butyric, propionic, lactic and acetic acid are some of the compounds produced by probiotics after they metabolize carbohydrates, which reduce the total pH of the small intestine and inhibit the growth of pathogenic bacteria [205]. The bactericidal action of bacteriocin mainly involves the formation of pores in the membrane, which are harmful to target cells and inhibit cell wall synthesis to achieve the relative stability of intestinal microorganisms [206]. In addition, probiotics may improve intestinal immunity by stimulating secretory IgA production and enhancing gut–brain communication. Recent studies have highlighted the potential of probiotics to stimulate the intestinal immune system by activating the aromatic hydrocarbon receptor (AhR) pathway, which is an important inflammatory regulator [207,208].

Prebiotics are defined as health-beneficial substrates selectively utilized by host microbes that preferentially stimulate the growth of a limited number of health-promoting bacteria in the gut and exert health benefits, including beneficial effects on gastrointestinal cognitive function, cardiometabolic health, and bone strength [205].

Synbiotics refer to the combination of prebiotics and probiotics: a mixture of living microbes and substrates selectively utilized by host microbes that exhibit synergistic effects and have a positive effect on the host [209]. The independence of the synbiotic components has been interpreted differently. One is independent selection, meaning that each ingredient is responsible for its specific effects, in which prebiotics are not necessarily preferentially metabolized by the probiotic strain and can be fermented by the host microbiota. The other is synergistic, in which prebiotics are specifically selected as substrates for particular strains of probiotics to support their growth [210].

Although there is evidence of the effectiveness and mechanisms of various interventions to modify gastrointestinal microbiota, there is still much work that needs to be completed. For example, differences in patient clinical characteristics lead to different responses to the administration of the same prebiotics/probiotics/synbiotics, which means that various factors, such as age, sex, diet, bowel habits, and microbiome composition can influence and help predict the outcome of interventions. At the same time, the effectiveness of various interventions should be evaluated from overall symptom improvement to more specific and overall symptom improvement, such as increased fecal frequency, metabolic protein levels, or reduction in abdominal distention or pain.

In addition, existing studies have focused on probiotics, and the effectiveness of other measures to improve the gut microbiota is not completely clear. There is a lack of reliable prospective studies for repeated validation. For example, existing trials still differed in FMT delivery methods, bacterial dose, fecal filtration methods, and administration frequency, and they failed to control for other donor factors, such as diet. Although original literature on brain-derived neurotrophic factors in Alzheimer’s or cortisol in stress may exist, no meta-analysis on the relationship between gastrointestinal microbiome changes and them was found. This might be a good direction for future research. In addition, many studies have proved that changes in the gastrointestinal microbiome are related to the occurrence of diseases, but no consensus has been reached on the causal relationship between the two. In other words, whether the disease breaks the balance of the gastrointestinal microbiome or the disorder of the gastrointestinal microbiome leads to the disease or whether there is a two-way effect between the two still needs to be confirmed by further reliable studies [167,168,171].

Overall, a number of factors contribute to changes in gut microbiota, which in turn have an impact on health outcomes. However, due to the large heterogeneity, the diversity of intestinal flora detection and the uncontrollability of other influencing factors, rigorous and credible meta-analyses are in short supply. Furthermore, there is still a lack of direct evidence linking specific changes in gut flora to health outcomes.

## 5. Conclusions

In summary, the gastrointestinal microbiome is closely associated with human health outcomes. Probiotics, prebiotics, synbiotics and FMT commonly used today are generally safe and beneficial to a variety of human health outcomes. Other interventions, such as traditional Chinese medicine, low FODMAP diet, and dietary fiber, have gradually shown positive results. In addition, the gastrointestinal microbiome is associated with the occurrence and development of a variety of diseases, but the causal relationship is still worth further investigation. However, the quality of evidence is still insufficient, so high-quality prospective studies are needed in the future.

## Data Availability

Not applicable.

## Supplementary Materials

The following supporting information can be downloaded at: https://www.mdpi.com/article/10.3390/nu14183726/s1, Table S1: Prebiotics/probiotics/synbiotics and multiple health outcomes; Table S2: Prebiotics/probiotics/synbiotics and mortality; Table S3: Prebiotics/probiotics/synbiotics and immune and metabolic outcomes; Table S4: Prebiotics/probiotics/synbiotics and immune and gastrointestinal disease; Table S5: Prebiotics/probiotics/synbiotics neurological and psychiatric outcomes; Table S6: Prebiotics/probiotics/synbiotics and maternal and infant outcomes; Table S7: Prebiotics/probiotics/synbiotics and other outcomes; Table S8: Prebiotics/probiotics/synbiotics and side effects; Table S9: Fecal microbiota transplant and multiple health outcomes; Table S10: Other interventions and multiple health outcomes; Table S11: Association between disease and changes in gastrointestinal microbiota and its metabolites; Table S12: AMSTAR2; Table S13: GRADE.
