# Supplementary material for: Gastrointestinal Microbiome and Multiple Health Outcomes: Umbrella Review
Source: Nutrients. 2022 Sep 9;14(18):3726. doi: 10.3390/nu14183726 (PMC9505003; doi:10.3390/nu14183726)
Supplement: Supplementary file 1 [file nutrients-14-03726-s001.zip › Supplementary Table S1.pdf]

Supplement Table S1. Prebiotics/probiotics/synbiotics and multiple health outcomes.

outcome(s) of interest	estimated summary effect (95% CI)	number of studies / total studies	number of intervention group or total participants	Heterogeneity (I^2, %)	the first author + year of publication	Intervention	Duration of Intervention/follow-up	study design	populations	outcome comparison	meta-analysis metric	type of effect model	publication bias
Immune and metabolic outcomes													
total cholesterol (TC) concentrations after prebiotic supplementation	-0.25 (-0.48 to -0.02)	9	313	14.4	BESERRA B 2015[1]	prebiotic or fructo-oligosaccharide or fructooligosaccharide or galacto-oligosaccharide or galactooligosaccharide or inulin or lactulose or FOS or GOS or Oligofructose or synbiotic	NP	All RCTs	Overweight (body mass index ≥ 25 kg/m²) or obese individuals	any versus none	SMD	random	No serious bias
LDL-c concentrations after prebiotic supplementation	-0.2 (-0.44 to -0.00)	9	313	0.0									
triglycerides concentrations after synbiotics supplementation	-0.43 (-0.70 to 0.15)*	3	148	0.0									
fasting insulin after synbiotics supplementation	-0.39 (-0.75 to -0.02)	3	180	0.0									
Triglycerides (TG) concentrations after prebiotic supplementation	-0.72 (-1.20 to -0.23)	9	313/513	32.7									
HDL-c concentrations after prebiotic supplementation	0.49 (0.01 to 0.97)	9	313/513	0.0									
diamine oxidase	-0.78(-0.93 to -0.63)	3	147/293	0.0	CHEN X R 2021[2]	early enteral nutrition (EEN) combined with probiotics	≥ 14 days	All RCTs	All patients met the criteria of severe stroke set by the fourth national Cerebrovascular Disease Conference, and were diagnosed by head CT and MRI; the onset was ≤3 days; Glasgow Coma Scale ≤9 points;	EEN combined with probiotics versus EEN	MD	Random	No serious bias
D-lactic acid	-0.06(-0.07 to -0.05)	4	162/324	0.0									
dysbacteriosis rate	0.17(0.07 to 0.41)	3	132/262	0.0							OR		
albumin	3.38(2.74 to 4.02)	15	637/1271	45.0							MD		
prealbumin	32.20(24.42 to 39.98)	7	302/601	44.0									
total protein	4.91(3.20 to 6.62)	7	336/672	77.0									
hemoglobin	9.62(7.92 to 11.32)	10	437/872	0.0									
immunoglobulin A	0.23(0.12 to 0.34)	4	144/280	20.0									

immunoglobulin G	0.33(0.21 to 0.45)	3	106/208	0.0					gastrointestinal function was normal or basically normal. Exclusion criteria were: patients with previous history of gastrointestinal and metabolic diseases, malignant tumor and malnutrition; patients with severe heart, liver, and kidney failure.				
immunoglobulin M	0.14(0.00 to 0.28)	4	147/290	46.0									
TC concentrations	-3.04(-4.88 to -1.21)mg/dL	12	767	45.9	YAN S 2019[3]	probiotics	6 weeks to 6 months	All RCTs	overweight or obesity was defined according to local standards	the probiotics vs. control groups	WMD	random	NP
LDL concentrations	-2.28(-3.60 to -0.96)mg/dL	11	737	36.9								fixed	
HDL concentrations	-0.26(-2.39 to 1.87)mg/dL*	12	767	95.5								random	
TG concentrations	-0.86(-2.54 to 0.83)17.0mg/dL*	11	726	17.0								fixed	
TC concentrations	-1.23(-3.52 to 1.06)mg/dL*	5	NP	0.0		single probiotic						fixed	
TC concentrations	-3.82(-6.14 to -1.50)mg/dL	7		49.8		multiple probiotic						fixed	
TG concentrations	9.10(1.38 to 16.81)mg/dL	4		0.0		single probiotic						fixed	
TG concentrations	-1.36(-3.08 to 0.37)mg/dL*	7		0.0		multiple probiotic						fixed	
Effects on Fasting Plasma Glucose (FPG)	-0.35 (-0.67 to -0.02)	6	215/436	64.0	WANG Z 2019[4]	Probiotics	NP	All RCTs	adults (≥18 years old) individuals with a BMI > 25 kg m-2	the probiotics vs. control groups	SMD	random	0.791
Effects on C-reactive protein (CRP)	-0.45 (-0.99 to 0.08) mg/L*	9	314/649	84.0	PONTES K S D S 2021 [5]	probiotics	NP	All RCTs	adults (18 years or older) with overweight or obesity defined according tolocal standards for body mass index (BMI)	Probiotics versus placebo	MD	random	No serious bias
Effects on tumor necrosis factor α	-0.16 (-0.24 to -0.08) pg/ml	4	96/191	11.0									
Effects on interleukin-6	0.05 (-0.28 to 0.39) pg/ml*	3	66/142	57.0									
Effects on glucose	-0.07 (-0.26 to 0.13)	13	418/977	92.0									0.01

	mmol/L*												
Effects on fasting insulin	-0.85 (-1.50 to -0.21) mcU/ml	10	322/776	30.0									No serious bias
Effects on glycated hemoglobin (HbA1C)	-0.03 (-0.14 to 0.09) %*	7	246/505	84.0									
adiponectin concentration	-0.04 (-0.28 to 0.19)*	8	NP	32.5	ROUHANI M H 2019[6]	probiotic supplementation	6-24 weeks	All RCTs	adults	probiotic versus controls	Hedges's adjusted g	random	0.65
leptin concentration	-0.30 (-0.86 to 0.26)*	6		81.6									0.81
fasting plasma glucose	-3.45 (-6.03 to -0.88)	5	169/337	84.0	COZZOLINO M 2020 [7]	therapy with probiotics or synbiotics.	administration of probiotics or synbiotics at least for 8 weeks	All RCTs	women with Polycystic ovary syndrome (PCOS) according to Rotterdam criteria undergoing therapy with probiotics or symbiotic	administration of probiotics or Synbiotics versus without therapy with probiotics or synbiotics or placebo	MD	random	NP
Homeostatic Model Assessment of Insulin Resistance (HOMA-IR)	-2.31 (-3.84 to -0.77)	5	169/337	94.0									
Insulin-Sensitivity Check Index	-0.62 (-1.07 to -0.17)	5	169/337	89.0									
TG	-23.35 (-35.23 to -11.47)	2	60/120	0.0									
total testosterone serum	-0.23 (-0.36 to -0.11)	4	113/226	52.0									
High sensitivity C reactive protein	-1.69 (-2.38 to -1.01)	4	120/240	49.0									
Nitric oxide	2.72 (0.21 to 5.23)	3	90/180	26.0									
Total antioxidant capacity	70.55 (38.84 to 102.25)	3	90/180	46.0									
Total glutathione	22.42 (2.08 to 42.75)	3	90/180	25.0									
fasting plasma glucose (FPG)	-1.35 (-2.22 to -0.49)	8	496	94.6	LI Y L 2021 [8]	probiotics, prebiotics, synbiotics intake separately or in combination with other drugs	8 weeks to 3 months	All RCTs	women with a definite diagnosis of PCOS	probiotics, prebiotics, synbiotics intake separately or in combination with other drugs versus placebo	SMD	random	No serious bias
fasting insulin	-0.68 (-1.08 to -0.27)	7	216/434	76.7									
homeostatic model of assessment for insulin resistance (HOMA-IR)	-0.73 (-1.15 to -0.31)	7	434	78.1									
quantitative insulin sensitivity check index (QUICKI)	2.00 (-0.79 to 3.22)*	6	189/379	96.1									
TG	-0.85 (-1.59 to -0.11)	7	428	92.2									
TC	-1.09 (-1.98 to -0.21)	7	428	94.3									
HDL-c	0.53 (-0.33 to 1.39) *	7	428	94.3									
low-density lipoprotein	-0.84 (-1.64 to -0.03)	7	428	93.4									

cholesterol (LDL-c)													
very-low-density lipoprotein cholesterol (VLDL-c)	-0.44 (-0.70 to -0.18)	4	235	0.0									
CRP	-0.63 (-1.37 to 0.10)*	9	558	93.9									
total anti-oxidant capacity (TAC)	0.04 (-0.75 to 0.83) *	3	82	83.9	DENG H Y 2020 [9]	Probiotic	12 weeks	All RCTs	Adult human participants who had a diagnosis of AD or MCI (aged over 18 y)	Probiotics versus pla-	SMD	rando m	NP
total glutathione (GSH)	0.04 (-0.28 to 0.35)	3	82	44.1						cebo		fixed	
malondialdehyde (MDA)	-0.60 (-0.91 to -0.28)	3	82	0.0									
nitric oxide (NO)	-0.16 (-0.47 to 0.15)	3	82	4.4									
high-sensitivity C-reactive protein (hs-CRP)	-0.57(-0.95 to -0.20)	2	57	0.0									
Plasma ammonia concentration (at one month)	-5.55 (-10.67 to -0.42) μmol/L	5	179/357	56.0	DALAL R 2017 [10]	probiotics	at one month	randomised clinical trials	people with any grade of acute or chronic hepatic encephalopathy	probiotics in any dosage vs. placebo or no interventio n, or with any other treatment in people with hepatic encephalo pathy.	MD	rando m	NP
Plasma ammonia concentration (at 2 months)	-5.11 (-14.56 to 4.34) μmol/L*	4	109/211	62.0			at 2 months						
Plasma ammonia concentration	-8.29 (-13.17, -3.41) μmol/L	10	357/705	79.0			follow-up: 1 month to 6 months						
Effect on plasma ammonia levels (week 4)	-6.86 (-19.01 to 5.30) μmol/L*	5	132/255	98.0	CAO Q 2018 [11]	Probiotic treatment	4-12 weeks	All RCTs	Patients with Minimal hepatic encephalopathy (MHE)	Probiotic treatment vs placebo or no treatment	MD	rando m	NP
Effect on plasma ammonia levels (week 4)	-0.80 (-1.56 to -0.08) μmol/L	4	112/220	0.0									
Reduction in serum endotoxin	-0.12 (-0.23 to -0.02)	3	58/109	10.0									
Effect on plasma ammonia levels (week 4)	-0.33 (-5.39 to 4.74) μmol/L*	4	164/325	0.0						Probiotic treatment vs lactulose treatment	MD		
serum ALT concentrations	-8.05 (-13.07 to -3.04) IU/L	16	990	93.0	KHALESI S 2018[12]	Probiotic; Synbiotic;	NP	RCT or non-rand omised	Adults older than 18 years of	probiotics vs. control	MD	rando m	NP
serum AST concentrations	-7.79 (-13.93 to -1.65)IU/L	16	990	97.7									

serum ALP level	-0.27 (-4.00 to 3.47)IU/L*	6	518	70.0		prebiotic		controlled	age				
serum GGT levels: gamma-glutamyl transpeptidase	-8.40 (-12.61 to -4.20)IU/L	8	438	53.0									
serum levels of albumin	-0.29 (-0.74 to 0.16)*	11	662	40.0									
serum bilirubin level	0.95 (0.48 to 1.42)μmol/L	13	806	4.0									
ALT	-7.31 (-9.89 to -4.74) U/L	27	NP	98.4	HERNANDEZ-SAAVEDRA D 2018 [13]	Any prebiotic or probiotic treatment, or a combination of both (synbiotic)	NP	All RCTs	Male and female patients of any age that presented at least 1 of the following: NAFLD, steatosis, liver fibrosis, steatohepatitis	Vs. placebo	MD	random	No serious bias
AST	-4.94 (-6.89 to -2.98) U/L	25		98.8									
γ-GT: gamma-glutamyl transferase	-7.86 (-11.36 to -4.36) U/L	9		94.8									
TNF-α	-2.04 (-4.70 to 0.61) ng/mL*	6		99.7									
hepatic cytokine CRP	-0.74 (-1.85 to 0.37) mg/L*	6		99.3									
total serum cholesterol	-7.97 (-11.41 to -4.54) mg/dL	15		99.3									
Serum LDL-c	-3.81 (-8.31 to 0.69) mg/dL*	12		97.0									
HDL-c	0.56 (-1.55 to 2.67)*	12		99.8									
Circulating TAG	-8.28 (-16.35 to -0.22)	16		97.8									
necrosis factor-α	-0.59 (-1.34 to 0.16)*	6	276	88.0	KHAN M Y 2019[14]	probiotics and/or synbiotics	NP	All RCTs	adult patients older than 18 years of age with NAFLD or NASH	Vs. placebo	SMD	random	NP
LDL	-0.48 (-1.04 to -0.08)	7	316	82.0									
HDL	-0.03 (-0.29 to 0.23)*	7	316	24.0									
total cholesterol level.	-0.48 (-0.94 to -0.01)	8	391	79.0									
triglycerides level.	-0.23 (-0.48 to 0.03)*	8	391	36.0									
insulin resistance	-0.12 (-0.63 to 0.40)*	6	289	93.0							MD		
fasting blood sugar level	-0.47 (-1.02 to 0.08)*	6	305	81.0							SMD		
high-sensitivity C-reactive protein level	-0.45 (-0.76 to -0.15)	4	234	25.0									
CRP	-0.40 (-0.79 to -0.02)	6	NP		SKONIECZNA-ZYDECKAK 2018 [15]	treatment with pro-/pre-/synbiotics	Preoperation 0-15 days, postoperation 0-until discharg	All RCTs	surgical patients	Probiotics vs. No probiotics	SMD	random	NP
IL-6	-0.41 (-0.70 o -0.12)	4											
white blood cells (WBC)	-0.60 (-1.45 to 0.24)*	6											
lactulose/mannitol (L/M) ratio	-0.28 (-0.82 to 0.27)*	4											

the concentration of butyrate ratio	0.67 (0.37 to 0.97)	4					e						
short chain fatty acids (SCFAs)-acetic	1.78 (0.80 to 2.76)	4											
propionic acids	0.46 (0.18 to 0.73)	4											
hs-CRP	-0.39 (-0.50 to -0.28)	31	NP	83.8	MILAJERDIA 2020[16]	probiotic	NP	All RCTs	adult	probiotics vs. placebo	SMD	fixed	NP
serum IL-6	-0.37 (-0.51 to -0.24)	16		69.7									
serum TNF-a concentrations	-0.21 (-0.34 to -0.08)	18		85.5									
serum Interleukin-10 (IL-10)	0.21 (0.04 to0.38)	11		48.5									
serum interleukin-1 beta (IL-1β)	-0.17 (-0.37 to 0.02)*	9		80.7									
serum IL-12	-0.47 (-0.67 to -0.27)	8		85.2									
serum IL-8	-0.01 (-0.30 to 0.28)*	5		73.0									
Interferon-gama (IFN-γ)	-0.08 (-0.31 to 0.15)*	5		0.0									
IL-17	006 (-0.34 to 0.46)*	3		0.0									
IL-4	-0.48 (-0.76 to -0.20)	3		0.0									
CRP	-0.77 (-1.48 to -0.05) mg/L	4	153	75.0	PAN H D 2017[17]	probiotic	Follow up: 8-48 weeks	All RCTs	patients with a clear diagnosis of RA and stable treatment of disease-modifying anti-rheumatic drugs (DMARDs)	probiotics vs. Placebo or no probiotics	SMD	random	NP
TNF-α	-1.35 (-1.99 to -0.71)	3	118	25.0							MD		
IL-6	-8.69 (-27.32 to 9.94)*	3	118	62.0									
IL-1β	-6.13 (-11.41 to -0.86)	3	118	50.0									
IL-10.	0.63 (-0.05 to 1.31)*	3	118	45.0									
CD4 counts	3.86 (-24.72 to 32.45) *	16	1025	55.7	FU Y S 2020[18]	probiotics, prebiotics, or synbiotics	NP	All RCTs	HIV-1-infected adults over 18 years of age	Vs. placebo or control groups	WMD	random	0.936
CD4 counts	21.24 (-12.95 to 55.39)*	10	385	0.0	ZHANG X L 2021[19]	probiotic	2-48 weeks	RCTs, clinical cohort studies, pilot study with controls, or other clinical	HIV/AIDS patients and patients diagnosed with similar recognized criteria based on laboratory evidence or methods of HIV	Probiotic vs. No probiotic	MD	fixed	No serious bias

								studies	infection				
lymphocyte T cell count	0.00 (-0.03 to 0.02) cells*10⁹/L*	4	NP	83.8	TAVAKOLY R 2021[20]	probiotics (supplement or fortified food)	4-20 weeks	All RCTs	adult athletes	placebo or non-fortified food	WMD	random	0.005
T helper	0.00 (-0.07 to 0.07) cells*10⁹/L*	5		0.0									0.089
T cytotoxic	-0.08 (-0.15 to -0.01) cells*10⁹/L	5		59.1									0.53
lymphocyte B	0.01 (-0.07 to 0.09)*	9		0.0									0.32
Lymphocyte	0.01 (-0.05 to 0.08)*	5		27.0									0.84
NK cells	0.04 (-0.01 to 0.08)*	4		3.2									0.679
leukocyte	0.23 (-0.09 to 0.55)*	10		49.0									0.446
monocyte	-0.00 (-0.03 to 0.02)*	8		3.8									0.577
neutrophil cells count	0.14 (-0.06 to 0.33)*	7		0.0									0.460
IgM	-0.22 (-0.63 to 0.19)*	3		0.0									0.885
IgA	0.24 (-0.09 to 0.57)*	4		14.0									0.729
IgA secretion rate	4.21 (-8.35 to16.77)*	3		13.8									0.208
The efficacy of syn and probiotics on hs-CRP	-0.58 (-0.79 to-0.37)	8	359	0.0	ASKARI G 2021[21]	Synbiotic or probiotics	NP	clinical trials	age >12 years old and disease duration between 2 and 20 years	Vs. Control group	SMD	random	0.195
IL-10	-0.12 (-2.11 to 1.87) pg/ml*	5	184	93.9							WMD		0.660
IL-6	-7.79 (-13.81 to -1.77) pg/ml	6	214	91.9									0.191
TNF-α	-1.05 (-2.01 to -0.10) pg/ml	5	184	85.2									0.102
ESR: Erythrocyte Sedimentation Rate	3.05 (-7.60 to 13.7) mm/h*	3	90	71.5									0.450
GSH: Glutathione	1311 (-55.68 to 81.91) μmol/L	4	222	88.8									0.099
MDA: Malondialdehyde	-0.36 (-0.68 to -0.04)	5	268	41.0							SMD		0.05

												8
TAC	0.08 (-0.16 to -0.32)	5	268	0.0								0.286
HOMA-IR	-0.71 (-1.05 to -0.37)	4	222	0.0							WMD	0.133
HOMA-β	-15.18 (-22.08 to -8.28)	3	174	0.0								0.096
serum creatinine	0.08 (-0.13 to 0.28) mg/dL*	5	126	0.0	JIA L P 2018[22]	probiotics	1-6 momths	All RCTs	adult chronic kidney disease (CKD) patients	probiotics vs controls	MD	random	NP
blood urea	-1.38 (-9.26 to 6.50)*	3	164	0.0									
p-cresyl sulfate	-0.57 (-0.99 to -0.14)	3	125	25.0							SMD		
hemoglobin	0.21 (-0.48 to 0.91)*	3	93	20.0							MD		
IL-6	0.37 (0.03 to 0.72)*	3	134	0.0							SMD		
CRP	0.49 (-6.45 to 3.11)*	3	NP	28.0							MD		
urea	-8.17 (-24.57 to 8.23)*	6	259	73.0	TAO S 2019 [23]	probiotics (specified probiotic or probiotic mixes) in any form (e.g., capsule, tablet or other food supplements or components), the probiotics could be administered at any dose and for any duration	Follow up: 6 weeks - 6 months	All RCTs	the participants were diagnosed with CKD, whether undergoing dialysis or not	probiotics vs placebo	MD	random	0.293
uric acid	-0.43 (-1.19 to 0.33)*	3	131	67.0									0.659
C-reactive protein	-0.48 (-1.29 to 0.33)*	6	227	52.0									0.770
creatinine	-0.18 (-0.82 to 0.47)	6	259	69.0									0.686
serum C-reactive protein	-0.37 (-0.72 to 0.03)*	7	178	0.0	THONGPRAYOON C 2019[24]	probiotic	NP	clinical trials	end-stage renal disease (ESRD) patients	Vs. Control group	SMD	random	0.09
total cholesterol	-0.25 (-0.46 to -0.04)	7	NP	0.0	BAKHTIARY M 2021 [25]	probiotic, prebiotic, or synbiotic	Follow up: 4-12 weeks	All RCTs	chronic kidney disease (CKD) patients	Vs. usual care or placebo	SMD	random	NP
HDL levels	-0.01 (-0.62 to 0.60)*	6		84.6									
LDL	-0.19 (-0.49 to 0.10)*	6		45.8									
triglyceride	-0.10 (-0.33 to 0.12)*	7		6.1									
VLDL	-0.21 (-0.47 to 0.05)*	4		0.0									
Fasting blood glucose	-0.41 (-0.65 to -0.17)	5		0.0									

(FBG)													
homeostatic model assessment of insulin resistance (HOMA-IR)	-0.63 (-0.95 to -0.30)	5		43.3									
insulin levels	-0.49 (-0.90 to -0.08)	5		65.2									
quantitative insulin sensitivity check index (QUICKI)	0.78 (0.51 to 1.05)	4		0.0									
hs-CRP	-0.52 (-0.81 to -0.22)	8		52.7									
CRP	0.21 (-0.17 to 0.59)*	4		0.0									
IL-6	-0.30 (-0.84 to 0.23)*	4		55.8									
Malondialdehyde	-0.79 (-1.22 to -0.37)	6		69.8									
glutathione	0.52 (0.19 to 0.86)	4		37.0									
nitric oxide	0.06 (-0.20 to 0.32)*	4		1.0									
serum urea	-0.30 (-2.20 to 1.61) mmol/L*	9	345	53.0	MCFARLANE C 2019[26]	prebiotics, probiotics, or synbiotics for a duration of at least 1 week	NP	RCT including crossover, cluster, or quasi-RCT designs	participants with CKD as defined by the Kidney Disease Outcome Quality Initiative Guidelines	Vs. comparison groups	MD	random	NP
Serum indoxyl sulfate	-0.02 (-0.09 to 0.05) mg/dL*	4	144	0.0									
serum PCS: p-cresyl sulfate	-0.13 (-0.41 to 0.15) mg/dL*	4	144	0.0									
HbA1c	-2.17 (-4.37 to 0.03) mmol/mol*	12	717	66.0	BOCK P M 2021[27]	any probiotic, prebiotic or synbiotic supplementation or combination of interventions with the aim of adjusting the gut microbiota	Follow up: 4-12 weeks	clinical trials	adult participants with type 1 or 2 diabetes diagnosis	Vs. Control group	MD	Random	No serious bias
fasting blood glucose	-0.58 (-0.86 to -0.30) mmol/l	36	1982	60.0									
insulinemia	-10.51 (-16.68 to -4.33) pmol/l	21	1194	74.0									
total cholesterol	-0.14 (-0.26 to -0.02) mmol/l	27	1524	39.0									
triacylglycerol	-0.11 (-0.20 to -0.02) mmol/l	29	1619	21.0									
HDL-cholesterol	0.04 (0.01 to 0.07) mmol/l	29	1619	24.0									
LDL-cholesterol	-0.05 (-0.14 to 0.05) mmol/l*	27	1531	37.0									
hs-CRP	-0.38 (-0.51 to -0.24)	13	NP	43.0	ZHENG H J 2019[28]	probiotics or synbiotics	6-12 weeks	All RCTs	diabetic patients (defined as age > 18 years)	Vs. placebo	SMD	fixed	0.3
TAC	0.31 (0.09 to 0.52)	13		58.9									0.10
MDA	-0.61 (-0.89 to -0.32)	10		66.7									0.03
NO	0.62 (0.25 to 0.99)	8		76.3								rando	0.03

												m	5
GSH	0.41 (0.26 to 0.55)	11		26.8								fixed	0.53 3
HbA1c	-0.24 (-0.44 to -0.04) %	8	489	27.0	TAO Y 2020[29]	probiotic	6-12 weeks	All RCTs	T2DM patients	probiotics vs placebo	MD	rando m	0.29
FBG	-0.44 (-0.74 to -0.15) mmol/L	14	842	0.0									0.86
HOMA-IR	-1.07 (-1.58 to -0.56)	8	524	18.0									0.07
blood glucose	-0.18 (-0.30 to -0.05)	4	186	0.0	CHI C 2020[30]	Probiotics	3-24 weeks	All RCTs	hypertension patients	probiotics vs placebo	MD	rando m	0.24
FBS	-2.28 (-7.37 to 2.80) mg/dL*	8	NP	81.0	HADI A 2021 [31]	probiotics or synbiotics	3-28 weeks	All RCTs	adults with metabolic syndrome	probiotics vs placebo	MD	rando m	0.30
HOMA-IR	-0.07 (-0.63 to 0.49)*	6		73.6									0.74
Insulin	-0.25 (-0.56 to 0.07)*	7		42.7						probiotics vs control group			0.36
TC	-6.66 (-13.25 to 0.07) mg/dL*	7		28.8									0.83
TG	-3.15 (-24.59 to 18.29)mg/dL*	7		68.5									0.22
LDL-c	-6.60 (-14.41 to 1.21) mg/dL*	7		52.7									0.69
HDL-c	0.27 (-2.80 to 3.34) mg/dL *	7		75.7									0.89
cortisol	-0.28 (-0.65 to 0.09)*	3	NP	0.0	AS'HABI A 2020 [32]	probiotics (supplement or food)	2-20 weeks	All RCTs	athletes	Vs. control	SMD	rando m	0.20
HDL- C	2.51 (1.04 to 6.05) mg/dL	6		50.3									0.25
LDL-C	-4.06 (-14.70 to 6.58) mg/dL*	3		0.0									0.98
TC	-5.99 (-10.83 to -6.04) mg/dL	6		67.5									0.70
TG	-32.26 (-60.44 to -4.08) mg/dL	6		93.0									0.48
serum calcium levels	3.82 (1.05 to 6.59)	4	NP	98.0	EJTAHED H 2021[33]	probiotic	NP	clinical trial	adults	Vs. control diets	SMD	Rando m	No serio us bias
serum phosphorus levels	1.14 (-0.44 to 2.73)*	4		96.1									
parathyroid hormone (PTH)	-5.35 (-9.83 to -0.86)	3		98.2									
urinary calcium	4.85 (1.16 to 8.53)	3		97.6									

CRP	-0.60 (-0.98 to -0.23)	7	342	64.0	MCLOUGHLIN R F 2017[34]	prebiotics, delivered orally, intravenously, or perrectum (enema)	2-12 weeks	All RCTs	human participants of any age and sex	vs. placebo or control	SMD	random	NP
IL-6	0.35 (-0.84 to 0.13)*	6	313	75.0									
TNF-α	-0.49 (-1.20 to 0.22)*	4	219	84.0									
postprandial ghrelin	-71.66 (-148.83 to 5.50) pg/ml*	3	NP	84.6	DA SILVA BORGES D 2020[35]	prebiotic or synbiotic	NP	clinical trials	overweight or obesity diagnosis (BMI ≥ 25 kg/m^2), (3) adults and older adults (≥ 18 y)	Vs. control	WMD	random	NP
levels of TNF-α	-0.24 (-0.69 to 0.21)*	7	NP	82.7	QU H 2019[36]	microbiota-driven therapy (probiotic, prebiotic or symbiotic)	14-180 days	the study described a randomized, controlled, parallel or crossover trial	healthy elderly individuals with age>60 years	vs. placebo	SMD	random	0.37
IL-6	-0.13 (-0.74 to 0.49)*	6		90.7									0.26
IL-10	1.00 (-0.15 to 2.15)*	6		96.3									0.20
CRP	-1.28 (-2.62 to 0.06)*	4		96.2									NP
IL-8	-0.03 (-0.67 to 0.61)*	4		88.0									
CRP	0.02 (-0.32 to 0.36)*	3	135	0.0	ZHU H F 2020[37]	treated with microecological preparations (probiotics, prebiotics, synbiotics, or a combination of two preparations)	15 days to 6months	All RCTs	obese adults (age ≥18 years) who had undergone bariatric surgery (a type of surgery was not restricted)	Vs. placebo or conventional treatment	SMD	Random	NP
IL-6	-0.10 (-0.81 to 0.61)*	3	135	0.0								random	
TNF-α	-0.29 (-0.64 to 0.05)*	3	135	0.0								fixed	
Gastrointestinal outcome													
Incidence of C. difficile-associated diarrhea	0.4 (0.3 to 0.52)	31	4525/8672	0.0	GOLDENBERG J Z 2017[38]	probiotics	NP	All RCTs	participants taking antibiotics	any versus none	RR	random	No serious bias
Antibiotic-associated diarrhea	0.58 (0.48 to 0.70)	33	8870	61.0									
the occurrence of diarrhea and positive stool C . difficile cytotoxin assay or culture	0.38 (0.22 to 0.67)	6	597/1194	39.0	WU Z J 2013[39]	Lactobacillus	NP	All RCTs	With or without antibiotics	any versus none	RR	fixed	NA
	0.63(0.31 to 1.28)*	4	431/861	0.0		Lactobacillus acidophilus							
incidence of CDI (diarrhea and either positive stool	0.42(0.3 to 0.57)	19	6261	0.0	SHEN N T	probiotics	NP	All RCTs	Hospitalized adults (18 years	any versus	RR	rando	No serio

cytotoxin, culture, or polymerase chain reaction testing for C difficile)					2017[40]				or older) receiving antibiotic therapy (intravenous, oral, or both) for any reason	none		m	us bias
	0.32 (0.22 to 0.48)	14	NP	0.0		Timing: study protocol specifying maximum interval from starting antibiotics to starting probiotics, 1-2 d							NP
	0.7 (0.4 to 1.23)*	5	NP	0.0		Timing: study protocol specifying maximum interval from starting antibiotics to starting probiotics, 3-7 d							NP
	0.73 (0.29 to 1.88)*	3	NP	0.0		L rhamnosus GG							NP
	0.63 (0.29 to 1.37)*	5	NP	0.0		S boulardii							NP
Incidence of Clostridium difficile–associated diarrhea(CDAD)	0.34(0.24 to 0.49)	20	40/1974	0.0	JOHNSTON B C 2012[41]	any dose of a specified probiotic of any strain	NP	All RCTs	adult or pediatric patients treated with antibiotics	any versus none	RR	random	No serious bias
clinical remission	2.40(1.49 to 3.88)	3	71/162	29.0	DANG X 2020[42]	probiotic VSL#3 as induction therapy	6-12weeks	All RCTs	patients with active UC	any versus none	OR	Fixed	1.34
clinical response	3.09(1.53 to 6.25)	3	89/162	46.0								Random	0.47
Clinical remission	1.73(1.19 to 2.54)	9	594	55.0	KAUR L 2020[43]	Probiotics	NP	All RCTs	People of any age with active ulcerative colitis previously diagnosed by clinical, endoscopic, histologic or surgical remissionas defined by study authors	any versus placebo	RR	Random	NP
	2.02(1.31 to 3.12)	5	374	32.0						probiotics with multiple strains versus placebo			
	1.44(0.79 to 2.63)	4	220	57.0						probiotics with single strain versus placebo			
improvement in colon endoscopic scores	-0.58(-1.11 to -0.05)	3	93	85.0	RUFINO M N 2021[44]	Synbiotic treatment	4weeks to 1 year	All RCTs	Human patients with ulcerative	conventional plus a synbiotic	RR	Fixed	NP

									colitis	or synbiotic treatment alone versus conventional plus placebo or placebo treatment alone			
remission rate	1.40(1.27 to 1.53)	27	1942	0.0	PENG L J 2019 [45]	probiotics in conjunction with mesalazine or sulfasalazine or aminosalicylic acid	4-12 weeks	All RCTs	children or adults with UC regardless of clinical setting	probiotics in conjunction with mesalazine or sulfasalazine or aminosalicylic acid versus mesalazine or sulfasalazine or aminosalyicylic acid alone	RR	Fixed	0.000
	1.33(1.16 to 1.54)	11	NP	33.4					children or adults with UC in both mild to moderate stages				
	1.40(1.27 to 1.64)	16	NP	0.0					children or adults with UC in active stage				
	1.36(1.20 to 1.53)	17	NP	19.5					children or adults with UC regardless of clinical setting	probiotics combined with aminosalicylic acid on UC in 3 types of protiotics			
	1.45(1.24 to 1.70)	8	NP	0.0						probiotics combined with aminosalicylic acid on UC in 2 types of protiotics			
	1.40(1.27 to 1.64)	2	NP	0.0						probiotics combined with			

										aminosalicylic acid on UC in 1 types of protiotics			
the efficacy of EcN in inducing remission for ulcerative colitis	0.92(0.15 to 5.66)*	3	267	86.0	LOSURDO G 2015[46]	Escherichia coli Nissle (EcN) 1917	NP	All RCTs	Patients with UC	EcN versus standard of care (mesalazine or ciprofloxacin)	OR	Fixed	No serious bias
										Regimen or placebo			
Maintenance of remission	1.07(0.70 to 1.64)*	4	442	0.0						EcN versus standard of care (mesalazine)			
Clinical relapse	0.87(0.63 to 1.18)*	4	361	17.0	IHEOZOR-EJIOFOR Z 2020[47]	Probiotics administered in any form (drink, powder, capsule), orally as a single species, or as a cocktail of multiple species	12-52 weeks	All RCTs	People of any age with ulcerative colitis in remission	Probiotics versus placebo	RR	Random	NP
Maintenance of clinical remission	1.16(0.98 to 1.37)*	2	141	0.0									
Clinical relapse	1.01(0.84 to 1.22)*	2	452	0.0						Probiotics versus 5-aminosalicylic acid (5-ASA) (mesalazine)			
Maintenance of clinical remission	1.06(0.90 to 1.25)*	1	125	NP									
Clinical relapse	1.11(0.66 to 1.84)*	2	242	14.7						Probiotic + 5-ASA (mesalazine) versus 5-ASA (mesalazine)			
Maintenance of clinical remission	1.05(0.89 to 1.24)*	1	122	NP									
remission in active UC patients, assessed with the Ulcerative Colitis Disease Activity Index (UCDAI) and Disease Activity Index (DAI)/Mayo scores	1.55(1.13 to 2.15)	6	503	29.0	ASTO E 2019[48]	probiotic, prebiotic, inulin, fructo-oligosaccharides, FOS, and synbiotic	NP	All RCTs	adults or children with active UC	administration (oral or rectal) of probiotics versus placebo or active	RR	random	NP
Efficacy of	1.73(1.23 to 2.43)	6	424	35.0		bifidobacteria-con							

inducing/maintaining remission						taining probiotics				treatment of UC to induce remission or to maintain remission			
	1.04(0.35 to 3.06)*	3	168	78.0		Probiotics without bifidobacteria							
	1.99(1.25 to 3.15)	4	348	49.0		VSL#3							
	1.38(0.86 to 2.21)*	4	313	56.0		probiotic, prebiotic, inulin, fructo-oligosaccharides, FOS, and synbiotic			adults or children with inactive UC				
Efficacy of Probiotics at Maintaining Remission	0.92(0.82 to 1.04)*	3	513	0.0		the probiotic Mutaflor			adults or children with active UC or inactive UC	the probiotic Mutaflor with mesalazine			
the incidence of abdorminal distension	0.27(0.17 to 0.43)	11	493/981	0.0	CHEN X R 2021[2]	early enteral nutrition (EEN) combined with probiotics	≥ 14 days	All RCTs	All patients met the criteria of severe stroke	early enteral nutrition (EEN) combined with probiotics versus EEN	OR	Random	No serious bias
the incidence of diarrhea	0.20(0.12 to 0.32)	14	604/1205	0.0									
the incidence of vomiting	0.20(0.12 to 0.32)	8	301/602	0.0									
the incidence of gastric retention	0.25(0.11 to 0.55)	3	148/296	0.0									
the incidence of constipation	0.20(0.12 to 0.32)	14	590/1177	0.0									
the incidence of reflux	0.47(0.21 to 1.02)*	7	316/632	19.0									
the incidence of digestive tract hemorrhage	0.31(0.18 to 0.52)	12	537/1072	0.0									
the incidence of stress ulcer	0.64(0.21 to 1.92)*	3	118/236	0.0									
the incidence of gastrointestinal complications	0.29(0.24 to 0.36)	17	3169/6352	0.0									
incidence of antibiotic-associated diarrhoea	0.63 (0.54 to 0.73)	42	5985/11305	60.0	GOODMAN C 2021 [49]	probiotic	NP	All RCTs	adult population to whom antibiotics were administered	probiotics versus any control	RR	Random	No serious bias
	0.54 (0.38 to 0.76)	4	324/651	0.0						High dose vs low dose			
incidence of antibiotic-associated diarrhoea	0.45 (0.36 to 0.56)	33	6352	57.0	GUO Q 2019 [50]	specific, identified probiotic in any form (e.g. capsule, sachet, yogurt).	5 days to 12 weeks	All RCTs	children (0 to 18 years) receiving antibiotics	Probiotic Versus Control (placebo or	RR	Random	No serious

	0.37 (0.30 to 0.46)	20	4038	NP		Trials investigating non-specific probiotic or yogurt agents (e.g. products that do not label the probiotic strain and dose) were not considered				non-active control) Probiotic dose (≥5 billion CFUs of probiotics/ day) Versus Control			bias
Duration of diarrhea (days)	-0.91 (-1.38 to -0.44)	8	1263	84.0			10 days to 12 weeks			Probiotic Versus Control	MD		
incidence of antibiotic-associated diarrhoea	0.58 (0.48 to 0.71)	30	3805/7023	58.0	JAFARNEJAD S 2016[51]	oral probiotic intake (in either supplements or foods)	NP	All RCTs	individuals aged >18	Probiotic Versus Control	RR	random	No serious bias
	0.47 (0.40 to 0.56)	25	20873826	43.0					adults (18–64 years)				
	0.94 (0.76 to 1.15)*	5	1718/3434	65.0					the elderly (>65 years)				
the incidence of AAD	0.49 (0.36 to 0.66)	17	1832/3611	58.0	BLAABJERGS 2017[52]	Probiotics	NA	All RCTs	outpatients taking oral antibiotics	probiotics vs. Placebo or no treatment	RR	random	No serious bias
	0.49 (0.36 to 0.66)	4	590/1139	0.0		S. boulardii							NP
Incidence of Antibiotic-Associated Diarrhea Using the Criteria Defined by WHO	0.54 (0.36 to 0.82)	7	876/1724	27.0		Probiotics							NP
the incidence of AAD	0.42 (-0.23 to 0.77)	7	726/1446	76.0					outpatients taking oral antibiotics, children (<15 years of age)		RD (risk difference)		
	0.42 (-0.23 to 0.77)	9	975/1936	47.0					outpatients taking oral antibiotics, adults (>15 years of age)				
inducing/maintaining IBD remission	1.13 (1.02 to 1.26)	21	918/1816	55.1	ZHANG X 2021 [53]	probiotics, prebiotics or	NP	All RCTs	Patient with Inflammatory	probiotics, prebiotics	RR	random	0.336

UC disease activity index	1.00 (0.27 to 1.73)	10	1026	0.0		synbiotics			bowel disease (IBD)	and synbiotics vs. Control group	SMD		0.463
CD disease activity index	-0.33 (-1.05 to 0.38)*	4	206	79.9							SMD		NP
IBD recurrence	0.48 (0.65 to 1.08)*	13	1372	52.7							RR		0.034
preventing relapse in active UC	1.02 (0.85 to 1.23)*	3	277/555	0.0	DERWA Y 2017 [54]	probiotics	NP	All RCTs	Adults (>90% of patients aged >16 years) with inflammatory bowel disease	probiotics vs. placebo	RR	random	NA
preventing relapse in CD in remission following a surgical resection	1.06 (0.59 to 1.92)*	4	105/213	37.0						Probiotics vs. 5-ASA			
prevention of endoscopic relapse of disease activity (Rutgeerts score 1 or more)	1.09 (0.92 to 1.29)*	4	163/233	53.0						probiotics vs. placebo			
Endoscopic relapse of disease activity (Rutgeerts score 2 or more)	1.04 (0.82 to 1.31)*	4	163/233	32.0									
Endoscopic relapse of disease activity (Rutgeerts score 3 or more)	1.13 (0.84 to 1.52)*	4	163/233	0.0									
Efficacy of probiotics in inducing remission in active UC	0.81 (0.72 to 0.91)	9	362/759	32.0	PABON-CARRASCO M 2020 [55]	probiotics	0.5-12 months	All RCTs	Adult with inflammatory bowel disease	probiotics vs. placebo	RR	fixed	No serious bias
Efficacy of probiotics in preventing relapse in quiescent UC	0.89 (0.68 to 1.15)*	3	147/284	0.0									
Efficacy of the Probiotics for the Remission of Crohn's Disease	0.90 (0.77 to 1.06)*	5	253/494	37.0									
effect on persistence of symptoms	0.79 (0.68 to 0.91)	21	1931	72.0	FORD A C 2018[56]	Combination probiotics	Minimum treatment duration of 7 days Minimum follow-up duration	All RCTs	Adults (participants aged >16 years)	probiotics vs. Control group	RR	random	0.06
	0.82 (0.63 to 1.06)*	8	893	83.0		Lactobacillus							NP
	0.67 (0.51 to 0.87)	3	314	63.0		Only Lactobacillus plantarum DSM 9843							
	0.70 (0.48 to 1.01)*	3	528	72.0		Bifidobacterium							
effect on global symptom or abdominal pain scores	-0.31 (-0.44 to -0.17)	19	1341	24.0		Combination probiotics					SMD		0.06

	-0.09 (-0.25 to 0.036)	9	989	25.0		Lactobacillus	of 7 days						NP
	0.12 (-0.27 to 0.50)*	4	388	70.0		Saccharomyces							
	-0.46 (-0.92 to 0.00)	3	501	77.0		Bifidobacterium							
bloating scores	-0.15 (-0.31 to 0.01)*	17	1155	36.0		Combination probiotics							
	-0.30 (-0.68 to 0.09)*	3	501	68.0		Bifidobacterium							
	-0.01 (-0.36 to 0.34)*	3	209	40.0		Saccharomyces							
flatulence symptom scores	-0.29 (-0.51 to -0.07)	8	318	0.0		Combination probiotics							
global symptom scores	-0.23 (-0.44 to -0.02)	17	2401	72.0	ASHA M Z 2020[57]	Probiotics or prebiotics	2-24 weeks	All RCTs	adult IBS patients (aged ≥18 years)	Probiotics or prebiotics vs. Control group	SMD	random	NP
abdominal pain scores	-0.18 (-0.43 to 0.07)*	29	3688	92.0		Probiotics or prebiotics or synbiotics							
	-0.71 (-1.33 to -0.10)	5	660	NP		probiotics containing Lactobacillus species							
Abdominal pain score	-1.15 (-2.05 to -0.24)	7	508	95.0	XU H L 2021 [58]	probiotics	4-8 weeks	All RCTs	Patients aged between 4 and 18 years with IBS met the diagnostic criteria (Rome II~IV)	probiotics vs. Control group	SMD	random	NP
Abdominal pain treatment success	3.44 (1.73 to 6.87)	3	163	0.0							RR		
Abdominal pain relief	1.48 (0.96 to 2.28)*	3	167	40.0							RR		
Frequency of abdominal pain	-0.82 (-1.57 to -0.07)	3	147	2.0							MD		
Standard abdominal pain	-0.15 (-0.27 to -0.04)	6	508	94.0							MD		
Abdominal pain	0.03 (-0.22 to 0.29)*	5	243	0.0	CONNELL M 2018[59]	VSL#3	4-8 weeks	All RCTs	IBS patient	VSL#3 vs. placebo	SMD	fixed	NP
Stool consistency	-0.00 (-0.09 to 0.08)*	3	177	0.0							MD		
Overall response	1.39 (0.98 to 1.96) *	3	177	0.0							RR		
Abdominal bloating	0.15 (-0.11 to 0.40)*	5	243	5.0							SMD		
Quality of Life	-0.08 (-0.39 to 0.22)*	3	170	0.0							SMD		
Abdominal pain	0.25 (-0.27 to 0.77)*	3	554	≥ 50%	YUAN F Q 2017[60]	B. Infantis only	4-8 weeks	All RCTs	patients with IBS, age greater than 15 years old	Probiotics Including B. Infantis versus placebo	SMD	random	NP
	0.23 (-0.03 to 0.49)*	5	666	≥ 50%		B. Infantis only or composite probiotic						random	

						Including B. Infantis							
Bloating/distention	0.20 (-0.19 to 0.58)*	3	554	≥ 50%		B. Infantis only						Fixed	
	0.21 (0.07 to 0.35)	5	666	≤ 50%		B. Infantis only or composite probiotic Including B. Infantis						fixed	
Bowel habit satisfaction	0.66 (-0.11 to 1.43)*	3	554	≥ 50%		B. Infantis only						rando m	
stool frequency	1.29 (0.69 to 1.89)*	10	1039	90.0	WEN Y 2020 [61]	Any species/strains/dos e/treatment regimen of live probiotics	2-12 weeks	All RCTs	Adult populations aged ≥16 y with functional chronic constipation defined by clinical symptoms, a physician's opinion, or the Rome I, II, or III criteria	probiotics vs. placebo	MD	rando m	0.18 3
Effect of probiotics on stool consistency for IBS-C	0.55 (0.27 to 0.82)	9	NP	80.0							SMD		0.58 3
	0.46 (0.08 to 0.85)	4	NP	NP		B. lactis							NP
Satisfaction in relief	0.95 (0.77 to 1.18)*	3	315			Any species/strains/dos e/treatment regimen of live probiotics					RR	fixed	
Bloating	-0.77 (-1.46 to -0.07)	4	460								SMD	rando m	
Flatulence	-0.34 (-0.70 to 0.02)*	3	158									fixed	
Incomplete evacuation	-0.77 (-1.14 to -0.39)	6	502									rando m	
Hard stools	-0.74 (-1.19 to -0.28)	5	280										
Ease of expulsion	0.81 (0.15 to 1.48)	4	392										
incidence of diarrhea	0.51 (0.37 to 0.70)	4	404	0.0	WU X D 2018 [62]	prophylactic probiotics in combination with antibiotics preoperatively	Preoper ation 1-14 days, postope ration 0-14 days	All RCTs	adult patients who undergo colorectal surgery	Vs. placebo or other base ingredients in combinatio n with antibiotics preoperati vely	RR	rando m	No serio us bias
abdominal distention	0.63 (0.48 to 0.83)	5	394	0.0									
stool frequency	-0.09 (-0.47 to 0.29)*	4	NP	0.0	ZHONG C Q 2017[63]	Probiotic	NP	RCTs, crossover trials, prospecti ve single-ar m trials and	Human	Probiotics vs. No probiotics	WMD	rando m	NP

								retrospective studies					
AIDS-related diarrhea	0.60 (0.44 to 0.82)	6	360	0.0	ZHANG X L 2021[19]	probiotic	2-48 weeks	RCTs, clinical cohort studies, pilot study with controls, or other clinical studies	HIV/AIDS patients and patients diagnosed with similar recognized criteria based on laboratory evidence or methods of HIV infection	Probiotic vs. No probiotic	MD	fixed	No serious bias
Stool frequency	0.98 (0.39 to 1.56)	15	1189	87.0	ZHANG C 2020[64]	Probiotics administered in the form of any formulation or dairy product	2-16 weeks	All RCTs	adults	probiotics vs placebo	MD	random	0.32
Gut transit time	-13.75 (-21.93 to -5.56)	5	325	69.0									0.81
Stool consistency	1.30 (0.22 to 2.38)	6	386	96.0									0.21
Bloating	-0.24 (-0.55 to 0.07)*	4	282	27.0									0.77
stool frequency	0.73 (0.14 to 1.31)	6	444	84.0	HUANG R 2017[65]	Any type of culture/strain/dose/therapy regimen of probiotics (include synbiotics)	3-12 weeks	All RCTs	children aged ≤ 18 years with constipation identified by relative clinical symptoms, pediatric physicians, or the Rome I, II, or III criteria	probiotics vs control group	MD	random	No serious bias
stool consistency	-0.07 (-0.21 to 0.06)*	3	267	92.0									
eradication of H. pylori	7.91 (2.97 to 21.05)	6	352	0.0	LOSURDO G 2018[66]	probiotic supplementation	NP	All RCTs	H. pylori-infected patients	Vs. placebo	OR	fixed	0.02
oropharyngeal colonization	0.87 (0.76 to 0.99)	6	915	0.0	JI T 2020[67]	probiotic	NP	All RCTs	adults who received mechanical ventilation (MV) therapy in hospitals	Vs. control	RR	fixed	NP
gastric colonization	0.65 (0.49 to 0.84)	4	519	30.2								fixed	
rate of gram-negative (GN) bacteria positivity	0.58 (0.58 to 0.86)	9	1247	60.0								random	
positive rate of gram-positive bacteria culture	0.95 (0.67 to 1.35)*	8	1175	31.3								fixed	
positive fungal culture rates	0.98 (0.67 to 1.43)*	7	886	26.5								fixed	
the incidence of diarrhea	0.69 (0.47 to 1.02)*	8	1219	59.4								rando	

													m	
fecal zonulin	-0.63 (-1.17 to -0.08)	3	NP	79.4	AS'HABI 2020 [32]	A	probiotics (supplement or food)	2-20 weeks	All RCTs	athletes	Vs. control	SMD	random	0.19
Stool pH	-0.65 (-0.76 to -0.54)	6	470	81.0	RAO 2009[68]	S	combination of prebiotics and probiotics vs controls	NP	All RCTs	full-term neonates	formula had different composition vs. the control formula (apart from prebiotics)	WMD	fixed	No serious bias
Diarrheal episodes	0.71 (0.35 to 1.43)*	3	606	99.0	LOHNER 2014[69]	S	prebiotics (prebiotics added to food in the manufacturing process or as a separate supplement)	a supplementation time of at least 2 months and an observation time of at least 4 months	All RCTs	healthy infants or children, aged 0–18 years	Vs. control (placebo or no supplemen-tation)	RR	random	NP
overall symptom response to treatment	0.62 (0.07 to 5.69)*	3	191	91.0	WILSON 2019[70]	B	prebiotics	2-12 weeks	All RCTs	Adult patients ≥18 and ≤64 y with Irritable bowel syndrome (IBS) or other Functional bowel disorders (FBDs)	vs. placebo	OR	random	No serious bias
integrative symptom score	-0.39 (-1.43 to 0.64)*	8	NP	97.0								SMD		
severity of abdominal pain	-0.83 (-1.84 to 0.18)*	10		97.0										
bloating	-0.57 (-1.67 to 0.52)*	9		97.0										
flatulence	-0.53 (-2.04 to 0.98)*	6		98.0										
necrotizing enterocolitis	0.79 (0.14 to 1.44)*	6	737	21.0	CHI 2019[71]	C	prebiotics	7-57 days	All RCTs	low birth weight infants (<2500 g) or preterm infants (<36 weeks)	vs. placebo	RR	random	0.73
feeding intolerance	0.87 (0.52 to 1.45)*	4	413	0.0										NA
full enteral feeding	-0.99 (-1.15 to -0.83)	7	576	0.0										0.63
stool frequency	0.52 (0.30 to 0.73)	6	294	13.0										0.32
Clinical remission	1.21 (1.18 to 1.24)	45	1816/3624	NP	Sohail 2018[72]	G	probiotic Medilac-S	4-96 weeks	All RCTs	human	Vs. control	RR	fixed	No serious
Endoscopy scores	0.71 (0.35 to 1.07)	7	270									MD	random	

Histological scores	1.07 (0.92 to 1.23)	8	501								MD	fixed	bias
incidence of Hirschsprung-associated entero-colitis (HAEC)	0.72 (0.37 to 1.39)*	5	93/198	37.0	NAKAMURA H 2018 [73]	probiotics	NP	NP	premature infants	Probiotics vs. control	OR	fixed	NP
intestinal transit time (ITT)	0.38 (0.23 to 0.53)	17	NP	20.0	MILLER L E 2016[74]	Probiotic or synbiotic	10-56 days	All RCTs	adult	probiotics vs. control	SMD	random	0.44
gastrointestinal symptoms (any questionnaire), continuous outcome	-0.48 (-1.21 to 0.25)*	4	197	81.0	SEILER C L 2020[75]	probiotics of any type and dose, administered orally in any form (drink/powder/capsule)	at least 2 weeks	All RCTs	patients With Celiac disease (CD)	probiotics vs placebo	MD	random	NP
Change in bifidobacteria species (log colony-forming units per gram [CFU/g]) from baseline to end of study, continuous outcome	0.85 (0.38 to 1.32)	4	224	62.0									
Change in lactobacilli species (log CFU/g) from baseline to end of study, continuous outcome	1.13 (-0.67 to 2.93)*	4	224	96.0									
functional dyspepsia (FD) symptom improvement	1.15 (1.01 to 1.30)*	5	409	0.0	ZHANG J 2020[76]	probiotic or prebiotic	≥ 7 days	All RCTs	adult patients (over the age of 16 years) with functional dyspepsia (FD)	Vs. placebo	RR	fixed	NP
percentages of Bifidobacterium species	31.75 (26.04 to 37.45)	3	NP	49.3	SORENSEN K 2021[77]	any amino acid formula (AAF) with synbiotics (AAF-Syn) or extensively hydrolysed formula (eHF) with synbiotics (eHF-Syn), Studies of infant formulae containing cow's milk protein, and eHF or AAF without synbiotics (including those with only a prebiotic or a probiotic) were excluded	7 days to 12 months	All RCTs	infants and children aged <3 years with IgE or non IgE mediated Cow's milk protein allergy (CMPA)	AAF-Syn than with AAF alone	MD	fixed	NP
percentages of Eubacterium rectale and Clostridium coccoides	-19.06 (-23.15 to -14.97)	3		18.1									

Other outcomes													
Detection of C. difficile in stool	0.86 (0.67 to 1.10)*	15	1214	0.0	GOLDENBERG J Z 2017[38]	probiotics	NP	All RCTs	participants taking antibiotics	any versus none	RR	random	No serious bias
Length of hospital stay	-0.17 (-1.03 to 0.68)*	4	NP	0							MD		
the incidence of infection	0.27(0.21 to 0.36)	16	1243/2483	0.0	CHEN X R 2021[2]	early enteral nutrition (EEN) combined with probiotics	≥ 14 days	All RCTs	All patients met the criteria of severe stroke	early enteral nutrition (EEN) combined with probiotics versus EEN	OR	Random	No serious bias
the incidence of pulmonary infection	0.28(0.20 to 0.39)	16	698/1391	0.0									
the incidence of urinary tract infection	0.23(0.13 to 0.43)	9	384/768	0.0									
the incidence of gastrointestinal infection	0.30(0.14to 0.66)	4	161/324	0.0									
the enteral nutrition target time	-2.18(-2.56 to -1.80)	4	163/324	84.0							MD		
the length of hospital stay	-8.70(-13.24 to -4.16)	3	131/260	98.0									
Effects on Body Weight	-0.55 (-0.91 to -0.19) kg	10	315/641	64.0	WANG Z 2019[4]	Probiotics	NP	All RCTs	adults (≥18 years old) individuals with a BMI > 25 kg m-2	the probiotics vs. control groups	MD	random	0.446
Effects on Body Mass Index (BMI)	-0.30 (-0.43 to -0.18) kg/m^2	11	357/717	59.0									0.006
	-0.29 (-0.46 to -0.12) kg m-2	6	213	66.6						Probiotics dosage ≥ 10^{10} CFU vs. control groups			NP
	-0.18 (-0.48 to 0.12) kg m-2*	4	119	46.9						Probiotics dosage < 10^{10} CFU vs. control groups			
	-0.36 (-0.52 to 0.20) kg m-2*	4	138	56.8						Single probiotics species vs. control groups			
	-0.15 (-0.39 to 0.10) kg m-2*	6	194	55.5						Multiple probiotics species vs. control			

					groups	
Effects on Waist Circumference	-1.20 (-2.21 to -0.19) cm	8	281/573	90.0	the probiotics vs. control groups	0.403
	-1.53 (-2.64 to -0.41) cm	6	212	86.9	Probiotics dosage ≥ 10^{10} CFU vs. control groups	NP
	-0.32 (-0.84 to 0.20) cm*	2	69	0.0	Probiotics dosage < 10^{10} CFU vs. control groups	
	-1.69 (-3.04 to 0.33) cm	4	168	91.6	Single probiotics species vs. control groups	
	-0.42 (-0.91 to 0.07) cm*	4	113	0.0	Multiple probiotics species vs. control groups	
	-1.34 (-2.76 to 0.08) cm*	6	187	92.3	Form of probiotics: Capsule or powder vs. control groups	
	-1.11 (-1.64 to -0.59) cm	2	94	0.0	Form of probiotics: Food vs. control groups	
Effects on Fat Mass	-0.91 (-1.19 to -0.63) kg	11	311/632	43.0	the probiotics vs. control groups	0.335
Effects on Fat Percentage	-0.92 (-1.27 to -0.56) %	6	224/450	57.0	the probiotics vs. control	0.068

										groups			
the effect on body weight	-0.54 (-1.09 to 0.01) kg*	18	660/1239	0.0	SUZUMURA E A 2019 [78]	Oral supplementation with probiotics or synbiotics as an isolated intervention or as part of a dietary plan	3-24weeks	Randomized or quasi-randomized controlled trials	Overweight or obese adults (BMI ≥ 25 kg/m^2 and age ≥ 18 y) with no health conditions that potentially affect metabolism or body weigh	probiotics or synbiotics oersus Control	MD	random	No serious bias
the effect on BMI	-0.19 (-0.43 to 0.04) kg/m^2*	15	447/895	51.0									
the effect on waist circumference	-0.82 (-1.43 to -0.21) cm	12	455/864	46.0									
the effect on body weight	-1.24 (-2.57 to 0.01) kg*	4	147/301	0.0						synbiotics vs control			NP
the effect on BMI	-0.33 (-1.22 to 0.57) kg/m^2*	2	50/100	0.0									
the effect on waist circumference	-3.39 (-5.07 to -1.72) cm	2	72/148	34.0									
effect of the PFMP on body weight	-0.91 (-1.59 to -0.22) kg	16	NP	67.3	MOHAMMADI H 2021 [79]	Probiotics fermented milk products (PFMP)	2-12weeks	All RCTs	adult individuals >18 years old	PFMP versus placebo	WMD	random	0.555
effect of the PFMP on BMI	-0.33 (-0.48 to 0.18) kg/m^2*	15		0.0									0.404
effect of the PFMP on waist circumference	-0.59 (-1.55 to 0.36) cm*	6		0.0									0.348
effect of the PFMP on body fat percentage	-3.75 (-9.39 to 1.90)*	5		90.3									0.402
The effects of synbiotic supplementation on BMI	-0.12 (-0.40 to 0.16) kg/m^2*	22	NP	0.0	HADI A 2020 [80]	synbiotic supplements	≥4 weeks	All randomized clinical trials	participants over18 years	synbiotic versus placebo	WMD	random	0.21
The effects of synbiotic supplementation on WC	-2.07 (-3.11 to -1.03) cm	12		0.0						synbiotic versus placebo			0.68
	-1.72 (-3.49 to 0.05) cm*	7		0.0						Baseline BMI ≥ 30 versus placebo			NP
	-2.25 (-3.53 to -0.97) cm	5		0.0						Baseline BMI 25-30 versus placebo			
	-1.30 (-3.44 to 0.83) cm*	6		0.0						Duration ≥ 12 Weeks versus placebo			
	-2.31 (-3.49 to -1.12) cm	6		0.0						Duration < 12 Weeks			

				versus placebo	
	-1.97 (-3.06 to -0.86) cm	9	0.0	Number of used strain: Multi versus placebo	
	-2.70 (-5.54 to 0.15) cm*	3	0.0	Number of used strain: Single versus placebo	
	-2.269(-4.76 to 0.37) cm*	3	0.0	Probiotic Dose > 2×10^{8} CFU versus placebo	
	-1.69 (-3.23 to -0.16) cm	8	0.0	Probiotic Dose ≤ 2×10^{8} CFU versus placebo	
The effects of synbiotic supplementation on weight	-0.80 (-1.56 to -0.03) kg	20	0.0	synbiotic versus placebo	0.37
	-0.54 (-2.93 to 1.85) kg*	9	0.0	Duration ≥ 12 Weeks versus placebo	NP
	-0.83 (-1.63 to -0.02) kg	11	0.0	Duration < 12 Weeks versus placebo	
	-1.48 (-2.68 to -0.28) kg	14	0.0	Number of used strain: Multi versus placebo	
	-0.33 (-1.32 to 0.67) kg*	6	0.0	Number of used	

										strain: Single versus placebo			
The effects of synbiotic supplementation on body fat	0.02 (-1.27 to 1.87) %*	6		67.3						synbiotic versus placebo			0.38
Effects on waist-to-hip ratio	-0.01 (-0.01 to -0.01)	5	192/395	24.0	PONTES K S D S 2021 [5]	probiotics	NP	All RCTs	adults (18 years or older) with overweight or obesity defined according to local standards for body mass index (BMI)	Probiotics versus placebo	MD	random	No serious bias
Effects on total abdominal fat area	-9.69 (-14.47 to -4.91) cm^2	4	350	0.0									
Effects on subcutaneous abdominal fat area	-6.24 (-8.94 to -3.54) cm^2	5	445	0.0									
Effects on visceral abdominal fatarea	-3.46 (-6.55 to 0.38) cm^{2*}	4	350	41.0									
Effects on Homeostasis Model of Assessment for Insulin Resistance Index (HOMA-IR)	-0.08 (-0.34 to 0.18) *	9	322/765	47.0									
Effects of probiotics on systolic blood pressure	-0.23 (-0.57 to 0.12)mmHg*	7	318/641	0.0									
Effects of probiotics on diastolic blood pressure	-0.35 (-0.76 to 0.06)mmHg*	7	318/641	0.0									
the risk of atopic sensitization	0.89 (0.77 to 1.03)*	17	2974	9.0	ZHANG G 2016 [81]	probiotics/synbiotics	Follow-up:1y - 2y of age	All RCTs	children in whom outcome assessment performed during infancy or childhood (ie, up to 12 years of age), without atopic diseases at the time of probiotic administration	any species/strains/doses regimen of live probiotics administered prenatally and/or postnatally within the first year of life versus placebo or no probiotics	RR	random	0.988
	0.78 (0.66 to 0.92)	12	1159/2085	0.0						probiotics were administered both			NP

											prenatally and postnatally versus placebo or no probiotics			
Food Sensitization	0.92 (0.75 to 1.12)*	9	1506	7.0							any species/strains/doses regimen of live probiotics administered prenatally and/or postnatally within the first year of life versus placebo or no probiotics			0.806
	0.77 (0.61 to 0.98)	5	477/807	0.0							probiotics were administered both prenatally and postnatally versus placebo or no probiotics			NP
number of pulmonary exacerbations	-0.32 (-0.68 to 0.03) episodes per participant*	4	113/225	41.0		COFFEY M J 2020 [82]	oral probiotic formulation	4-12 months	All RCTs	participants who fulfil consensus diagnostic criteria for CF (Cystic fibrosis)	Any oral probiotic formulation (any strain(s), dose or formulation, with or without a prebiotic) compared to any other probiotic	MD	random	NP
intestinal i. calprotectin	-47.41 (-93.28 to -1.54) µg/g	4	88/177	0.0										
FEV1 (% predicted)	1.36 (-1.20 to 3.91)% *	5	143/284	0.0										

									formulation, placebo or no treatment control			
body mass index	-0.25 (-0.48 to -0.03) kg/m²	7	208/416	97.0	COZZOLINO M 2020 [7]	therapy with probiotics or synbiotics.	administration of probiotics or synbiotics at least for 8 weeks	All RCTs	women with Polycystic ovary syndrome (PCOS) according to Rotterdam criteria undergoing therapy with probiotics or symbiotic	administration of probiotics or Synbiotics versus without therapy with probiotics or synbiotics or placebo	MD	random	NP
body weight	-0.75 (-1.45 to -0.05) kg	7	208/416	98.0									
the modified Ferriman–Gallway score	-1.00 (-1.75 to -0.26)	3	90/180	83.0									
Body Weight	-0.11 (-0.34 to 0.13)*	12	731	61.5	LI Y L 2021 [8]	probiotics, prebiotics, synbiotics intake separ- ately or in combination with other drugs	8 weeks to 3 months	All RCTs	women with a definite diagnosis of Polycystic ovary syndrome (PCOS)	probiotics, prebiotics, synbiotics intake separately or in combination with other drugs versus placebo	SMD	random	0.321
BMI	-0.10 (-0.30 to 0.09)*	13	791	47.3									0.150
Waist circumference (WC)	0.37 (-0.78 to 1.53)*	5	316	95.5									No serious bias
hip circumference (HC)	-0.25 (-0.78 to 0.27)*	4	NP	76.9									
polymorphonuclear phagocytic capacity	0.74 (0.38 to 1.11)	3	NP	NP	MILLER L E 2017 [83]	Bifidobacterium (B.) animalis ssp. lactis HN019	3-6 weeks	randomized or non-randomized controlled studies	healthy, elderly (≥60 years) adults	consumption of B. lactis HN019 vs. control	SMD	random	NP
natural killer (NK) cell tumoricidal activity	0.43 (0.08 to 0.78)	3	NP	NP									
Polymorphonuclear cell phagocytic capacity	1.37 (0.86 to 1.88)	9	NP	80.0	Miller LE 2019[84]	probiotic consumption	3-12 weeks	prospective controlled studies	healthy older adults (mean age ≥ 60 years)	consumption of probiotic vs control	SMD	random	0.38
natural killer (NK) cell tumoricidal activity	0.55 (0.37 to 0.73)	15		43.0									0.51
No-recovery (incomplete resolution of clinical symptoms)	0.67(0.56 to 0.79)	10	574	48.0	DALAL R 2017 [10]	probiotics	follow-up: 1 month to 3 months	randomised clinical trials	people with any grade of acute or chronic hepatic encephalopathy	probiotics in any dosage vs. placebo or no interventio	RR	random	NP

										n, or with any other treatment in people with hepatic encephalopathy.			
Effects of Probiotics on Late-Onset Sepsis	0.88 (0.80 to 0.97)	28	4304/8535	23.0	DERMYSHI E 2017 [85]	enteral adminis- tration of probiotics	NP	All RCTs	very low birth weight (VLBW) (<1,500 g) preterm (<34 weeks gestational age) infants with enteral administration of probiotics initiated within 10 days	enteral administration of probiotics vs. Control group	RR	fixed	0.242
incidence of Late onset sepsis (LOS)	0.79 (0.71 to 0.88)	25	2934/5868	0.0	ACETI A 2017 [86]	any probiotic	NP	Randomized and quasi-randomized controlled trials	preterm infants (gestational age (GA) <37 weeks) received within one month of age any probiotic	any probiotic vs. placebo or no treatment	RR	fixed	No serious bias
	0.75 (0.65 to 0.86)	20	1705/3402	0.0					preterm infants (gestational age (GA) <37 weeks) (exclusively human-milk -fed)				
	0.77 (0.51 to 1.17)*	16	398/800	1.0					preterm infants (gestational age (GA) <37 weeks) (mixed feeding)				
Effect on body mass index	-0.39 (-0.49 to -0.29) kg/m^2	20	NP	97.9	HERNANDEZ-SAAVEDRA D 2018 [13]	Any prebiotic or probiotic treatment, or a combination of both (synbiotic)	NP	All RCTs	Male and female patients of any age that presented at least 1 of the following: NAFLD, steatosis, liver fibrosis,	Vs. placebo	MD	random	No serious bias

									steatohepatitis				
Liver stiffness as measured by the fibro scan	-0.62 (-0.89 to -0.35)	4	217	83.0	KHAN M Y 2019[14]	probiotics and/or synbiotics	NP	All RCTs	adult patients older than 18 years of age with NAFLD or NASH	Vs. placebo	MD	random	NP
Infections Rate	0.21 (0.11 to 0.41)	4	246	1.0	SAWAS T 2015 [87]	probiotics	NP	controlled studies	Liver transplantation	Vs. placebo	RR	fixed	NP
urinary tract infection (UTI)	0.14 (0.04 to 0.47)	4	246	0.0									
intra-abdominal infection	0.27 (0.09 to 0.78)	4	246	0.0									
length of hospital stay	-1.41 (-1.97 to -0.86) days	3	196	0.0							MD		
duration of antibiotic use	-3.89 (-4.17 to -3.60) days	3	196	80.0									
Acute graft rejection	0.73 (0.44 to 1.22)*	3	179	0.0							RR		
postoperative infection rate	0.35 (0.24 to 0.50)	23	1696	47.0	YANG Z 2017 [88]	Probiotic or synbiotic	NP	All RCTs	perioperative patient	Vs. placebo or no probiotic inter- vention.	OR	random	0.006
pneumonia	0.44 (0.28 to 0.68)	16	1276	0.0									0.238
urinary tract infection	0.30 (0.16 to 0.55)	13	1151	0.0									0.666
wound infection	0.58 (0.42 to 0.80)	18	1723	0.0									0.097
intra-abdominal abscess	0.65 (0.28 to 1.55)	10	495	0.0									0.744
Postoperative mortality	1.19 (0.52 to 2.74)	8	1648	21.0									0.179
lengths of hospital stay	-3.20 (-4.87 to -1.54) days	12	807	89.0							MD		0.094
antibiotic therapy	-3.40 (-4.67 to -2.13)	11	922	92.0									0.954
ICU stay	-0.46 (-1.07 to 0.14)*	7	453	70.0									0.151
the incidence of surgical site infections (SSI)	0.72 (0.56 to 0.92)	13	1374	0.0	WU X D 2018 [62]	prophylactic probiotics in combination with antibiotics preoperatively	Preoperation 1-14 days, postoperation	All RCTs	adult patients who undergo colorectal surgery	Vs. placebo or other base ingredients in combinatio	RR	random	No serious bias
pneumonia	0.50 (0.29 to 0.84)	10	807	0.0									

urinary tract infection	0.50 (025 to 0.98)	8	705	0.0			0-14 days			n with antibiotics preoperatively			
cumulative duration of antibiotic therapy	-1.24 (-1.61 to -0.86) days	5	504	0.0									
length of stay	-1.16 (-1.91 to -0.41) days	7	514	0.0							MD		
prevention of bacterial translocation	0.75 (0.46 to 1.21)*	7	NP	NP	SKONIECZNA-ZYDECKAK 2018 [15]	treatment with pro-/pre-/synbiotics	Preoperation 0-15 days, postoperation 0-until discharge	All RCTs	surgical patients	Probiotics vs. No probiotics	SMD	random	NP
pneumonia	0.55 (0.38 to 0.80)	21		0.0							RR		
surgical site infection	0.55 (0.39 to 0.78)	10		7.89									
Abdominal distention	0.64 (0.47 to 0.86)	4		0.0									
Anastomotic leakage	0.73 (0.43 to 1.24)*	9		3.69									
Deep organ space	0.62 (0.31 to 1.25)*	5		0.0									
Diarrhea	0.49 (0.37, 0.67)	7		0.0									
Intraabdominal abscess	0.69 (0.35 to 1.37)*	8		0.0									
MRSA infection	0.34 (0.11 to 1.06)*	4		0.0									
Peritonitis	0.34 (0.09 to 1.32)*	4		0.0									
Reoperation	0.43 (0.14 to 1.29)*	3		0.0									
Sepsis	0.69 (0.58, 0.81)	7		0.0									
Superficial incisional	0.53 (0.38 to 0.74)	15		0.0									
UTI	0.32 (0.18 to 0.57)	13		0.0									
Blood loss	0.004 (-0.120 to 0.128)*	7		0.0									
Duration of antibiotic therapy	-0.60 (-1.09 to -0.10)	10		91.0							SMD		
Duration of postoperative pyrexia	-0.44 (-0.68 to -0.192）	4		38.1									
Fluid diet	-0.35 (-0.58 to -0.12)	3		0.0									
Hospital stay	-0.48 (-0.66, -0.30)	10		45.7									
ICU stay	-0.10 (-0.36, 0.16)*	4		0.0									
Operating time	0.02 (-0.14 to 0.18)*	11		49.0									
Solid diet	-0.31 (-0.50, -0.12)	4		0.0									

the incidence of small intestinal bacterial overgrowth (SIBO)	0.54 (0.19 to 1.52)*	3	NP	83.7	ZHONG C Q 2017[63]	Probiotic	NP	RCTs, crossover trials, prospective single-arm trials and retrospective studies	Human	Probiotics vs. No probiotics	RR	random	NP
SIBO Decontamination Rate	1.61 (1.19 to 2.17)*	5		25.7							RR		
H_2 Volume in HBT	-36.35 (-44.23 to -28.47) ppm	4		0.0							WMD		
disease activity score in 28 joints (DAS28)	-0.11 (-0.47 to 0.24)*	3	132	87.0	PAN H D 2017[17]	probiotic	Follow up: 8-48 weeks	All RCTs	patients with a clear diagnosis of RA and stable treatment of disease-modifying anti-rheumatic drugs (DMARDs)	probiotics vs. Placebo or no probiotics	MD	random	NP
quality-of-life (QoL)	0.36 (0.07 to 0.64)	11	1501	86.0	Le Morvan De Sequeira 2021[89]	probiotic	4 weeks-6 months	All RCTs	adults of both sexes and of all ages with IBS	probiotics vs. Placebo	SMD	random	NP
seroprotection rate of H1N1 strain	1.83 (1.19 to 2.82)	6	386	0.0	LEI W T 2017[90]	probiotics, prebiotics, or synbiotics	NP	All RCTs	human adults	Probiotic vs. Control group	OR	random	0.37
seroconversion rate of H1N1 strain	0.52 (0.75 to 3.09)*	6	553	51.0									0.95
seroprotection rate of H3N2 strain	2.85 (1.59 to 5.10)	7	388	0.0									0.86
seroconversion rate of H3N2 strain	2.54 (0.93 to 6.91)*	6	553	83.0									0.76
seroprotection rate of B strain	0.99 (0.65 to 1.52)*	7	389	0.0									0.61
seroconversion rate of B strain	2.11 (1.38 to 3.21)	6	553	0.0									0.47
hemagglutination inhibition titers of A/H1N1 strain after influenza vaccination	7.14 (2.73 to 11.55)	12	688	96.0	YEH T L 2018[91]	probiotics or prebiotics	NP	Control trials	adults	probiotics or prebiotics vs. placebo	MD	random	No serious bias
hemagglutination inhibition titers of A/H3N2 strain after influenza vaccination	17.19 (3.39 to 30.99)	12	688	100.0									

hemagglutination inhibition titers of B strain after influenza vaccination	4.17 (0.37 to 7.96)	12	688	94.0									
estimated glomerular filtration rate preservation	2.10 (-1.31 to 5.52)*	4	212	68.0	TAO S 2019 [23]	probiotics (specified probiotic or probiotic mixes) in any form (e.g., capsule, tablet or other food supplements or components), the probiotics could be administered at any dose and for any duration	Follow up: 6 weeks - 6 months	All RCTs	the participants were diagnosed with CKD, whether undergoing dialysis or not	probiotics vs placebo	MD	random	0.051
total antioxidant capacity	0.42 (0.18 to 0.66)	5	NP	0.0	BAKHTIARY M 2021 [25]	probiotic, prebiotic, or synbiotic	Follow up: 4-12 weeks	All RCTs	chronic kidney disease (CKD) patients	Vs. usual care or placebo	SMD	random	NP
BMI	0.05 (-0.17 to 0.27)*	6		0.0									
eGFR, estimated glomerular filtration rate	0.34 (-2.20 to 2.89 mL/min/1.73 m^{2*}	3	132	0.0	MCFARLANE C 2019[26]	prebiotics, probiotics, or synbiotics for a duration of at least 1 week	NP	RCT including crossover, cluster, or quasi-RCT designs	participants with CKD as defined by the Kidney Disease Outcome Quality Initiative Guidelines	Vs. comparison groups	MD	random	NP
weight	0.40 (-0.95 to 1.74) kg*	7	357	0.0									
BMI	-0.06(-0.53 to 0.41) kg/m^{2*}	NP	NP	0.0	BOCK P M 2021[27]	any probiotic, prebiotic or synbiotic supplementation or combination of interventions with the aim of adjusting the gut microbiota	Follow up: 4-12 weeks	clinical trials	adult participants with type 1 or 2 diabetes diagnosis	Vs. Control group	MD	Random	No serious bias
Systolic blood pressure	-0.93 (-1.58 to -0.27)	6	NP	89.6	HENDIJANI F 2018[92]	probiotics in the form of any pharmaceutical formulations or dairy 15 products	NP	(all) 10 randomized controlled trials (RCTs) and 1 single blinded trial and 2	adult diabetic patients	Vs. placebo	SMD	random	No serious bias
Diastolic blood pressure	-0.88 (-1.76 to -0.01)	7		94.0									

								crossover trials					
the risk of developing asthma	0.86 (0.73 to 1.01)*	14	4138	0.0	DU X Z 2019[93]	Probiotics were supplemented since the pre- and/or postnatal periods, with ? 3 months of intervention duration	Follow up: 1-15 y	All RCTs	healthy children, with or without a high risk of developing allergic diseases and without concurrent treatments	probiotics vs placebo	RR	fixed	No serious bias
Allergic rhinitis	1.03 (0.77 to 1.39)*	5	1897	68.0								random	
Wheeze	0.94 (0.81 to 1.08)*	8	1957	20.0								fixed	
Positive aeroallergen skin-prick test (SPT) result	0.74 (0.50 to 1.09)*	4	870	0.0									
systolic blood pressure	-2.05 (-3.87 to -0.24)	11	628	10.0	CHI C 2020[30]	Probiotics	3-24 weeks	All RCTs	hypertension patients	probiotics vs placebo	MD	random	0.68
diastolic blood pressure	-1.26 (-2.51 to -0.00)	12	688	0.0									0.44
BMI	-1.03 (-1.28 to -0.79)	8	398	0.0									0.27
Weight	-1.54 (-4.83 to 1.75) kg*	5	NP	0.0	HADI A 2021 [31]	probiotics or synbiotics	3-28 weeks	All RCTs	adults with metabolic syndrome	probiotics vs control group	MD	random	0.53
BMI	-0.06 (-0.56 to 0.44) kg/m2*	7		0.0						probiotics vs placebo			0.31
WC	-1.33 (-3.30 to 0.64) cm*	6		28.9									0.92
eczema	0.59 (0.45 to 0.78)	17	4011	69.0	SUN S Y 2021[94]	Probiotic started during pregnancy or within 12 months of age	NP	All RCTs	participants should be high risk, defined as at least one parent or older sibling of the enrolling infants who had doctor-diagnosed allergic disease	Vs. placebo	OR	random	NP
atopic eczema	0.66 (0.48 to 0.91)	6	1193	8.0									
asthma	0.88 (0.69 to 1.10)*	12	3209	15.0									
rhinitis	0.83 (0.55 to 1.24)*	5	1214	42.0									
wheeze	0.82 (0.67 to 1.01)*	8	1922	0.0									
allergic disease	0.83 (0.58 to 1.17)*	5	1556	44.0									
sensation	0.99 (0.75 to 1.30)*	7	1174	10.0									
Self-assessed eczema symptoms on a 20-point visual analogue scale of itch and sleep disturbance (SCORAD part C	-0.90 (-2.84 to 1.04)*	5	309	79.0	BOYLE R J 2009[95]	live orally ingested microorganisms, including bacteria, fungi or yeasts, ingested singly or in combination-organisms were presumed to be live unless the authors specified	NP	All RCTs	participants of any age with doctor-diagnosed eczema	Vs. control group received no treatment, placebo or another non-microbial intervention	MD	random	NP
nvestigator-rated eczema severity (total SCORAD) at the end of treatment	-2.46 (-7.45 to 2.53)*	7	588	76.0									

						that they were killed							
Ventilator-associated pneumonia (VAP) Incidence	0.67 (0.59 to 0.77)	20	2428	11.3	JI T 2020[67]	probiotic	NP	All RCTs	adults who received mechanical ventilation (MV) therapy in hospitals	Vs. control	RR	fixed	0.273
length of ICU stay	-1.42 (-2.52 to -0.31)	15	1895	90.7							WMD	random	0.891
length of hospital stay	-1.79 (-3.89 to -0.31)	8	1161	86.3							WMD		NA
mortality rates	0.83 (0.70 to 1.02)*	15	1959	0.0							RR		0.301
days of MV	-1.06 (-2.54 to 0.43)*	12	1563	96.9							WMD		0.558
Days of using antibiotics	-2.28 (-4.02 to -0.55)	5	664	72.7									NP
the onset of VAP following intubation	2.09 (-0.15 to 4.32)*	4	265	92.7									
incidence of bacteremia	0.51 (0.35 to 0.76)	6	688	39.8							RR	Random	
rate of multidrug resistant (MDR) infections	0.64 (0.52 to 0.79)	9	1487	45.3								fixed	
Symptom scores	-0.09 (-0.44 to 0.26)*	3	251	46.0	WANG F Y 2021[96]	probiotics or prebiotics	NP	All RCTs	human	Vs. placebo	SMD	random	NP
Infection rate	0.80 (0.64 to 1.01)*	5	286	73.0							RR	random	
the number of subjects having at least 1 RTI episode	0.89 (0.82 to 0.96)	17	4513	82.0	WANG Y Z 2016[97]	probiotic	5 days to 2years	All RCTs	healthy children (from birth to 18 years), who developed upper respiratory tract infections (URTIs) or lower respiratory tract infections (LRTIs) at some point during the study	Vs. placebo	RR	random	NP
the duration of RTI illness episodes	-0.60 (-1.49 to 0.30)*	9	2817	88.0							MD	random	
the number of days of RTI illness	-0.16 (-0.29 to -0.02)	6	2067	0.0									
the days absent from day care/school	-0.94 (-1.72 to -0.15)	8	1499	87.0									
ΔPittsburgh Sleep Quality Index (PSQI) score	0.78 (0.40 to 1.17)*	11	452	0.0	IRWIN C 2020 [98]	administered live bacteria (probiotics) or heat-inactivated/killed probiotic bacteria	NP	randomized controlled trials or quasi-experimental	adults >18 years of age	Vs. placebo	MD	random	NP
sleep efficiency	0.02 (-1.21 to 1.24)*	6	453	0.0						change from	MD		
sleep latency	0.69 (-1.36 to 0.51)*	7	467	0.0							MD		

score derived from other subjective ratings scales	0.08 (-0.14 to 0.30)*	8	549	36.0			(paraprobiotics) as a daily oral supplement (e.g., capsules/tablets, fermented milk, yoghurt,powder sachets)		(nonrandomized controlled trials)		baseline	SMD		
EDSS scores	-1.22 (-2.40 to -0.03)	3	173	92.0	JIANG 2021[99]	J	probiotic	NP	All RCTs	Adult human participants with ages between 18 and 55 with clinically definite relapsing-remitting multiple sclerosis (RRMS), diagnosed according to McDonald criteria and an expanded disability status scale (EDSS) score ≤4.5	probiotics vs. placebos	SMD	random	NP
BDI total scores	-1.58 (-3.03 to -0.12)	3	173	94.0										
GHQ scores	-0.71 (-1.02 to -0.40)	3	173	0.0										
Physical Growth During the First Year of Life	1.07 (0.14 to 1.99) g	4	436	0.0	RAO 2009[68]	S	combination of prebiotics and probiotics vs controls	NP	All RCTs	full-term neonates	formula had different composition vs. the control formula (apart from prebiotics)	WMD	fixed	No serious bias
Incidence of infections requiring antibiotic therapy	0.68 (0.61 to 0.77)	3	1122	0.0	LOHNER 2014[69]	S	prebiotics (prebiotics added to food in the manufacturing process or as a separate supplement)	a supplementation time of at least 2 months and an observation time of at least 4 months	All RCTs	healthy infants or children, aged 0–18 years	Vs. control (placebo or no supplemen-tation)	RR	random	NP

QoL, quality of life	0.06 (-0.14 to 0.25)	4	322	0.0	WILSON B 2019[70]	prebiotics	2-12 weeks	All RCTs	Adult patients ≥18 and ≤64 y with Irritable bowel syndrome (IBS) or other Functional bowel disorders (FBDs)	vs. placebo	SMD	random	No serious bias
Effects on sepsis	0.64 (0.51 to 0.78)	11	1106	0.0	CHI C 2019[71]	prebiotics	7-57 days	All RCTs	low birth weight infants (<2500 g) or preterm infants (<36 weeks)	vs. placebo	RR	random	0.80
length of hospital stay	-5.18 (-8.94 to -1.41)	8	733	83.0									0.08
BMI	-0.49 (-0.86 to -0.13)	4	NP	41.3	STACHOWSKA E 2020[100]	treatment with prebiotic (soluble fiber or insoluble fiber) in the form of a supplement, e.g., pills, powder, etc.	NP	All RCTs	patients with confirmed non-alcoholic steatohepatitis (NASH)	Vs. control	SMD	random	0.83
liver stiffness measurement (LSM)	-0.70 (-1.00 to -0.40) kpa	4	235	93.4	SHARPTONS R 2019[101]	interventions in any of the following 4 categories: antibiotics, probiotics, synbiotics, or fecal microbiota transplantation	NP	All RCTs	patients with nonalcoholic fatty liver disease (NAFLD)	Vs. placebo, usual care, or no intervention in patients with NAFLD	WMD	random	NP
improvement in hepatic steatosis, as graded by ultrasound	2.40 (1.50 to 3.84)	6	384	22.4							OR		
SCORAD values at 8 weeks	-6.56 (-11.43 to -1.68)	6	NP	77.1	CHANG Y 2016[102]	synbiotics	NP	All RCTs	patients with atopic dermatitis (AD)	Vs. control	WMD	random	NP
Quality of Life	-0.14(-0.45 to 0.17)*	3	159	0.0	ZHU H F 2020[37]	treated with microecological preparations (probiotics, prebiotics, synbiotics, or a combination of two preparations)	15 days to 6months	All RCTs	obese adults (age ≥18 years) who had undergone bariatric surgery (a type of surgery was not restricted)	Vs. placebo or conventional treatment	SMD	fixed	NP
Excess Weight Loss	0.45 (-0.16 to 1.05)*	4	154	68.0								random	
Infection Rates	0.46 (0.31 to 0.67)	11	913	43.0	KAHN J 2020[103]	pro-/synbiotics perioperatively	NP	Randomized and non-randomized control studies	Patients accepted liver resection (LR) or liver transplantation (LT).	vs. controls	RR	random	NP

the number of participants who experienced ≥1 Respiratory Tract Infections (RTI) episode	0.84 (0.73 to 0.96)	9	2845	43.8	CHAN C K Y 2020[104]	Synbiotics were all administered through oral ingestion and composed of ≥1 probiotic bacterial strain plus ≥1 type of prebiotics in different forms, such as powdered milk, yogurt, or capsules	7 days to 12 months	randomized controlled trials (RCTs) and placebo-controlled trials	individuals of any age (including full-term infants) with no pretrial symptoms	Vs. without Synbiotics	Rate ratio	random	0.20
the proportion of participants experiencing RTIs	0.84 (0.74 to 0.95)	7	7273	47.7							Risk ratio		0.55
the proportion of infants who had infections	0.35 (0.19 to 0.67)	3	NP	0.0	SORENSEN K 2021[77]	any amino acid formula (AAF) with synbiotics (AAF-Syn) or extensively hydrolysed formula (eHF) with synbiotics (eHF-Syn), Studies of infant formulae containing cow's milk protein, and eHF or AAF without synbiotics (including those with only a prebiotic or a probiotic) were excluded	7 days to 12 months	All RCTs	infants and children aged <3 years with IgE or non IgE mediated Cow's milk protein allergy (CMPA)	AAF-Syn than with AAF alone	OR	fixed	NP
septic complications	0.42 (0.23 to 0.75)	5	NP	NP	Kinross JM 2013[105]	prebiotic,	NP	All RCTs	patients underwent abdominal surgery with the use of a prebi- otic, probiotic, or a synbiotic agent	Vs. either received a placebo or received no therapy	OR	random	0.25
	0.25 (0.10 to 0.60)	9				synbiotic							0.08
Postoperative Pneumonia	0.38 (0.07 to 2.03)	3				prebiotic,							NA
	0.89 (0.31 to 2.55)	7				synbiotic							0.14
Wound Infection	0.73 (0.29 to 1.81)	3				prebiotic,							NA
	0.48 (0.19 to 1.20)	7				synbiotic							0.14
Urinary Tract Infection	0.54 (0.03 to 10.73)	3				prebiotic,							NA
	0.29 (0.07 to1.16)	6				synbiotic							0.14
Length of Hospital Stay	-0.07(-0.55 to 0.42)	7				synbiotic					WMD		0.15
Length of Antibiotic	-1.71(-3.2 to-0.21)	4				synbiotic							0.02

Treatment													
Effect of probiotics on development of overt HE (week 4)	0.22 (0.07 to 0.67)	3	95/191	0.0	CAO Q 2018 [11]	Probiotic treatment	4-12 weeks	All RCTs	Patients with Minimal hepatic encephalopathy (MHE)	Probiotic treatment vs placebo or no treatment	OR	random	NP
Effect of probiotics on development of overt HE (week 12)	0.48 (0.21 to 1.12) *	3	104/205	0.0									
Improvement in Minimal hepatic encephalopathy (MHE) (week 12)	0.15 (0.07 to 0.32)	3	104/205	0.0									
Spinal bone mineral density (BMD)	0.65 (-0.18 to 1.47)*	4	NP	90.3	EJTAHED H 2021[33]	probiotic	NP	clinical trial	adults	Vs. control diets	SMD	Random	No serious bias
total hip bone mineral density (BMD)	1.45 (-0.38 to 3.28)*	3		96.3									
Mortality													
All-cause mortality	0.58(0.23 to 1.44)*	7	404	0.0	DALAL R 2017 [10]	probiotics	follow-up: 2 weeks to 3 months)	randomised clinical trials	people with any grade of acute or chronic hepatic encephalopathy	probiotics in any dosage vs. placebo or no intervention, or with any other treatment in people with hepatic encephalopathy.	RR	random	NP
Effects of Probiotics on Mortality	0.77 (0.65 to 0.92)	27	4117/8156	16.0	DERMYSHI E 2017 [85]	enteral administration of probiotics	NP	All RCTs	very low birth weight (VLBW)(<1,500 g) preterm (<34 weeks gestational age) infants with enteral administration	enteral administration of probiotics vs. Control group	RR	fixed	0.012

									of probiotics initiated within 10 days				
Mortality (all-cause before hospital discharge)	0.76 (0.65 to 0.89)	51	10170	0.0	SHARIF S 2020 [106]	any probiotic or probiotic combination for at least one week	at least one week	RCTs and quasi-RCTs	very preterm (< 32 weeks' gestation) or very low birth weight (< 1500 g) infants	any probiotic or probiotic combination vs. placebo or no treatment.	RR	fixed	0.11
Mortality (all-cause before hospital discharge)	0.91 (0.71 to 1.16)*	6	1661	0.0					extremely preterm (< 28 weeks' gestation) or extremely low birth weight (< 1000 g) infants				NP
mortality	0.58 (0.46 to 0.75)	14	1789/3583	4.5	YANG Y 2014 [107]	enteral administration of any probiotic started within the first 10 days of life	at least 7 days	All RCTs	preterm infants (without consideration of birth weight)	probiotic vs. Placebo group	RR	fixed	No obvious bias
The effect of probiotics in decreasing death rate	0.69 (0.55 to 0.87)<br>0.52 (0.34 to 0.80)	27<br>12	4399/8717<br>1635/3175	36.2<br>42.7	JIANG T L 2020 [108]	probiotics<br>mixed probiotics	NP	All RCTs	preterm infants < 37 weeks and/or birth weight < 2500 g	Probiotics vs. control	RR	random	No obvious bias
Incidence of death.	0.48 (0.36 to 0.64)	20	4286	0.0	LIU D P 2020[109]	administration of Lactobacillus for preventing NEC	NP	All RCTs	neonates of gestational age < 37 weeks	Lactobacillus with placebo	RR	fixed	No obvious bias
Incidence of death.	0.74 (0.60 to 0.92)	15	2579/5053	29.0	ZHU X L 2019[110]	bifidobacteria treatment for preventing NEC	NP	All RCTs	neonates with gestational age (GA) < 37 weeks	Bifidobacteria vs. control	RR	fixed	NP
Mortality	1.17 (0.54 to 2.57)*	7	NP	20.4	SKONIECZNA-ZYDECKAK 2018 [15]	treatment with pro-/pre-/synbiotics	Preoperation 0-15 days, postoperation 0-until discharge	All RCTs	surgical patients	Probiotics vs. No probiotics	SMD	random	NP
mortality	0.58 (0.36 to 0.94)	9	924	0.0	CHI C 2019[71]	prebiotics	7-57 days	All RCTs	low birth weight infants (<2500 g)	vs. placebo	RR	random	0.73

									or preterm infants (<36 weeks)				
operative mortality	1.39 (0.57 to 3.44)*	5	NP	NP	Kinross JM 2013[105]	prebiotic	NP	All RCTs	patients underwent abdominal surgery with the use of a prebiotic, probiotic, or a synbiotic agent	Vs. either received a placebo or received no therapy	WMD	random	NA
Neurological and psychiatric outcomes													
effect of probiotics on cognition	0.24 (0.05 to 0.42)	7	NP	0.0	LV T 2021[111]	probiotics intervention (any dose, strain, or administration method)	8-12 weeks	All RCTs	adults age ≥18 years	probiotics intervention (any dose, strain, or administration method) versus control	SMD	Random	NP
cognitive function in individuals with Alzheimer's disease	0.56 (-0.06 to 1.18)*	3	82/161	73.0	KRÜGER J F 2021[112]	Probiotic or synbiotic, orally or enterally administered, with no restriction on strains, doses, and frequency and duration of administration, provided information was reported	NP	All RCTs	individuals with dementia who underwent an intervention with probiotics or synbiotics	probiotics or synbiotics intervention versus control or placebo group	SMD	Random	NP
cognitive enhancement	0.37 (0.14 to 0.61)	5	154/297	24.0	DENG H Y 2020 [9]	Probiotic	12 weeks	All RCTs	Adult human participants who had a diagnosis of AD or MCI (aged over 18 y)	Probiotics versus placebo	SMD	fixed	0.54
alleviating anxiety	-0.12 (-0.28 to 0.04)*	12	1551	51.0	LIU B 2018 [113]	probiotics	4-24 weeks	All RCTs	Adult (≥ 18 y)	probiotics vs. placebo	SMD	random	0.139

Preclinical psychological symptoms of anxiety, depression, and stress	0.34 (0.07 to 0.61)	9	785	67.0	MCKEAN J 2017[114]	probiotics	21-56 days	All RCTs	adult healthy volunteers<br>(aged ≥ 18 years)	probiotics vs. placebo	SMD	random	NP
depressive symptoms	-0.31 (-0.56 to -0.07)	19	1901	82.0	GOH K K 2019 [115]	probiotics	4-20 weeks	All RCTs	general population or clinical population	probiotics vs. placebo	SMD	random	No serious bias
	-0.75 (-1.09 to -0.41)	3	144	0.0					patients with major depressive disorder				
	-0.26 (-0.70 to -0.17)	7	722	84.0					Other clinical diagnosis population				
	-0.25 (-0.60 to 0.11)*	12	1035	82.0					Health population				
	-0.01 (-0.30 to 0.27)*	11	1070	71.0		Single strain probiotics			general population or clinical population				
	-0.57 (-0.96 to -0.18)	13	831	85.0		multiple strain probiotics							
schizophrenia symptoms (total PANSS score)	-0.09 (-0.38 to 0.20)*	3	172	0.0	NG Q X 2019[116]	probiotic	NP	All RCTs	patients with at least moderately severe psychotic symptoms, aged 18–65 years	probiotics vs. Placebo	SMD	fixed	NA
Subjective stress level	-0.14 (-0.27 to -0.01)	6	1043	0.0	ZHANG N 2020[117]	Probiotics	30 days to 24 weeks	All RCTs	participants were in a healthy state, without known major health problems	probiotics vs placebo	SMD	fixed	NA
Stress-related subthreshold anxiety/depression level	-0.13 (-0.26 to -0.00)	9	940	11.0									
Anxiety	-0.23 (-0.54 to 0.08)*	3	171	0.0	WILSON B 2019[70]	prebiotics	2-12 weeks	All RCTs	Adult patients ≥18 and ≤64 y with Irritable bowel syndrome (IBS) or other Functional bowel disorders (FBDs)	vs. placebo	SMD	random	No serious bias
continuous anxiety outcomes	-0.03(-0.21 to 0.14)*	5	617	12.0	COHEN KADOSH K 2021[118]	pro- or prebiotic administration (any form)	8-84 days	Controlled trials	Mean age in the range of 10–24 years old	vs. controls	SMD	random	NP

depression	-0.08 (-0.30 to 0.15)*	5	NP	NP	Liu RT 2019[119]	prebiotic	four hours to four weeks	controlled clinical trial	Patient with depression or anxiety	Vs. placebo	SMD	random	NP
anxiety	0.12 (-0.03 to 0.27)*	4											
depression	-0.24 (-0.36 to -0.12)	23		48.2		probiotic	eight days to 45 weeks						0.08
anxiety	-0.10 (-0.19 to -0.01)	22		5.0									0.49
Maternal and infant outcomes													
Occurrence of new cases of colic at study end, as defined by the Wessel criteria.	0.46 (0.18 to 1.19)*	3	571/1148	72.0	ONG T G 2019 [120]	any probiotic, alone or in combination with a prebiotic (also known as synbiotics)	NP	All RCTs	newborn infants less than one month of age without the diagnosis of infantile colic	any probiotic, alone or in combination with a prebiotic (also known as synbiotics), versus no intervention, another intervention(s) or placebo	RR	random	NA
Adverse effects, including parental depression and mental illness, choking, bacterial infection or apparent life-threatening/serious events (dichotomous outcome)	1.02 (0.14 to 7.21)*	6 (only one trial got estimated)	1851(1014)	-									
Duration of crying	-32.57 (-55.60 to -9.54) min per day	3	355/707	93.0							MD		
	-20.65 (-47.23 to -5.92) min per day	2	345/687	89.0					term babies only				
Effects on Crying Duration	-2.01 (-2.76 to -1.26)	12	NP	95.2	SKONIECZNA-ŻYDECKAK 2020 [121]	probiotics/synbiotics	21-90 days	All RCTs	An infant who is less than 5 months of age when the symptoms start and finish	newborns taking probiotics vs. placebos (controls)	SMD	random	0.0021
Response (The probability of at least a 50% reduction in crying duration) to Probiotic Intervention	1.98 (1.50 to 2.62)	9		68.9									0.5
mean cognitive	0.20 (-1.42 to 1.83)*	4	1388	0.0	UPADHYAY R P 2020 [122]	probiotic and/or prebiotic supplementation	NP	All RCTs	preterm infants (<37 weeks gestation and/or birth weight <1500 g) with intervention being probiotic and/or prebiotic supplementation	probiotic vs. Control group	WMD	random	No serious bias
motor scores in children under 5 years of age	1.04 (-0.43 to 2.50)*	4	1388	0.0									
the risk of cognitive impairment	0.98 (0.75 to 1.27)*	4	1388	0.0							RR		
the risk of motor impairment	1.06 (0.79 to 1.42)*	4	1388	0.0									

the risk of neurodevelopment impairment (NDI)	0.90 (0.75 to 1.09)*	5	1637	9.9									
the risk of cerebral palsy	1.33 (0.84 to 2.11)*	4	1388	0.0									
the risk of hearing impairment	0.38 (0.14 to 1.07)*	3	1139	37.5									
Necrotising enterocolitis (before hospital discharge)	0.54 (0.45 to 0.65)	54	10604	17.0	SHARIF S 2020 [106]	any probiotic or probiotic combination for at least one week	at least one week	RCTs and quasi-RCTs	very preterm (< 32 weeks' gestation) or very low birth weight (< 1500 g) infants	any probiotic or probiotic combination vs. placebo or no treatment.	RR	fixed	0.04
Invasive infection (before hospital discharge)	0.89 (0.82 to 0.97)	47	9762	19.0									0.86
Duration of birth hospitalisation	-1.93 (-3.78 to -0.08) days	22	5458	26.0							MD		No serious bias
neurodevelopmental impairment	1.03 (0.84 to 1.26)*	5	1518	0.0							RR		NP
Cerebral palsy	1.13 (0.74 to 1.72)*	5	1512	18.0									
Visual impairment	0.50 (0.14 to 1.80)*	4	1356	0.0									
Hearing impairment	0.46 (0.18 to 1.17)*	4	1356	32.0									
Necrotising enterocolitis (before hospital discharge)	0.90 (0.68 to 1.21)*	8	1712	0.0					extremely preterm (< 28 weeks' gestation) or extremely low birth weight (< 1000 g) infants				
Invasive infection (before hospital discharge)	0.90 (0.76 to 1.06)*	6	1471	0.0									
Effect of probiotics on Stage I or higher necrotizing enterocolitis (NEC) (Bell stage ≥I; gestational age <37 weeks)	0.35 (0.27 to 0.44)	27	3298/6655	0.0	YANG Y 2014 [107]	enteral administration of any probiotic started within the first 10 days of life	at least 7 days	All RCTs	preterm infants (without consideration of birth weight)	probiotic vs. Placebo group	RR	fixed	No obvious bias
the effect of probiotics on Stage II or higher NEC(Bell Stage ≥II; gestational age <37 weeks)	0.34 (0.25 to 0.48)	17	2042/4198	0.0									
the influence on NEC in early preterm infants (Bell Stage ≥I; gestational age <34 weeks)	0.39 (0.27 to 0.56)	12	1266/2495	0.0									
culture-positive sepsis	0.94 (0.83 to 1.06)*	17	1950/4043	24.2									

age reaching full feeds	-1.66 (-3.60 to 0.27)*	9	821/1621	89.9							WMD	random	
The effect of probiotics in preventing stage II-III severe NEC	0.51 (0.38 to 0.67)	27	4491/8901	28.4	JIANG T L 2020 [108]	probiotics	NP	All RCTs	preterm infants < 37 weeks and/or birth weight < 2500 g	Probiotics vs. control	RR	random	No obvious bias
	0.39 (0.26 to 0.57)	11	1590/3083	0.0		mixed probiotics							
The effect of probiotics in decreasing death rate	0.69 (0.55 to 0.87)	27	4399/8717	36.2		probiotics							
	0.52 (0.34 to 0.80)	12	1635/3175	42.7		mixed probiotics							
incidence of Hirschsprung-associated entero-colitis (HAEC)	0.72 (0.37 to 1.39)*	5	93/198	37.0	NAKAMURA H 2018 [73]	probiotics	NP	NP	premature infants	Probiotics vs. control	OR	fixed	NP
Incidence of Bell stage ≥ II NEC	0.34 (0.25 to 0.46)	23	2359/4686	0.0	LIU D P 2020[109]	administration of Lactobacillus for preventing NEC	NP	All RCTs	neonates of gestational age < 37 weeks	Lactobacillus with placebo	RR	fixed	No obvious bias
Incidence of sepsis	0.90 (0.72 to 1.12)*	10	2129	41.0								random	
Incidence of death.	0.48 (0.36 to 0.64)	20	4286	0.0								fixed	
Incidence of NEC Stage ≥ II (Bell staging criteria)	0.38 (0.25to 0.58)	20	2836/5568	43.0	ZHU X L 2019[110]	bifidobacteria treatment for preventing NEC	NP	All RCTs	neonates with gestational age (GA) < 37 weeks	Bifidobacteria vs. control	RR	random	NP
Incidence of sepsis	0.87 (0.73 to 1.03)*	17	2075/4034	43.0									
Incidence of death.	0.74 (0.60 to 0.92)	15	2579/5053	29.0								fixed	
infant crying/distress, measured as duration or the number of episodes	-65.10 (-85.85 to -44.38)	3	209	55.0	SUNG V 2013 [123]	probiotics	NP	All RCTs	infants who were younger than 3 months	oral probiotic supplementation vs. placebo or standard care or no care	Median Difference	Random	NP
	-67.72 (-99.79 to -35.64)	3	209	70.0							Mean Difference		
Preterm birth < 37 weeks' gestation	0.92(0.32 to 2.67)*	4	518	14.0	GREV J 2018 [124]	Before delivery (probiotics given only prior to delivery)	NP	All RCTs	pregnant women	probiotics administered to pregnant women (< 36 weeks' gestation) versus placebo or no intervention	RR	fixed	NP
Newborn Birth Weight*	-10.27 (-90.17 to 69.63)*	7	1093	34.0	WANG C C	probiotic or	NP	All RCTs	Pregnant women	probiotic	SMD	rando	0.60

					2020[125]	synbiotic			(> 18 years) with body mass index (BMI) ≥ 25 kg/m2 or diagnosed as having GDM, gestational age < 34 weeks	or synbiotic vs. placebo		m	
fasting blood glucose	-0.11 (-0.16 to -0.05)mmol/L	10	1139	71.0	HAN M M 2019 [126]	probiotic	NP	All RCTs	pregnant women >18 years-of-age with or without gestational diabetes mellitus (GDM)	probiotic vs. placebo	MD	random	No obvious bias
serum insulin	-2.06 (-2.98 to -1.15)μU/mL	8	340/682	77.0									
(HOMA-IR)	-0.38 (-0.54 to -0.21)μU/mL	8	340/682	64.0									
QUICKI	0.01 (0.00 to 0.03)	4	165/327	85.0									
total cholesterol	-0.56 (-1.07 to -0.05)	6	579	89.0							SMD		
triglycerides	-0.66 (-1.28 to -0.04)	6	579	92.0									
HDL-C	-0.13 (-0.34 to 0.07)*	6	579	33.0									
LDL-C	-0.45 (-0.97 to 0.06)*	6	579	89.0									
Diagnosis of gestational diabetes mellitus	0.80 (0.54 to 1.20)*	6	1440	64.0	DAVIDSON S 2021[127]	probiotics	NP	All RCTs	pregnant women not previously diagnosed with diabetes mellitus	Probiotics vs. placebo	RR	random	NP
Hypertensive disorders of pregnancy (including pre-eclampsia, pregnancy-induced hypertension and eclampsia)	1.39 (0.96 to 2.01)*	4	955	0.0									
pre-eclampsia	1.85 (1.04 to 3.29)*	4	955	0.0									
Caesarean section	1.00 (0.86 to 1.17)*	6	1520	0.0									
Large-for-gestational age	0.99 (0.72 to 1.36)*	4	919	0.0									
Perinatal mortality (including stillbirth and neonatal death)	0.33 (0.01 to 8.02)*	3	709	0.0									
Maternal weight gain during pregnancy	0.30 (-0.67 to 1.26) kg*	4	853	40.0							MD		
Maternal fasting plasma glucose	-0.14 (-0.12 to 0.05) mmol/L*	7	1519	69.0									
Maternal plasma glucose at one hour of a 75 g oral	-0.07 (-0.27 to 0.13) mmol/L*	4	1110	0.0									
glucose tolerance test													

(OGTT)													
Maternal plasma glucose at two hours of a 75 g OGTT	0.02 (-0.13 to 0.18) mmol/L*	4	1186	0.0									
Infant Stillbirth	0.59 (0.14 to 2.46)*	5	1128	0.0							RR		
Preterm birth (less than 37 weeks' gestation and less than 32 weeks' gestation)	1.36 (0.82 to 2.01)*	6	1484	0.0									
Infant Macrosomia	1.13 (0.86 to 1.48)*	3	952	20.0									
Small-for-gestational age	0.51 (0.30 to 0.85)*	3	814	0.0									
Birthweight	26.87 (-49.52 to 103.26) g*	6	1524	42.0							MD		
Infant Head circumference	-0.04 (-0.27 to 0.18) cm*	3	789	21.0									
Infant Length	0.02 (-0.54 to 0.59) cm*	3	786	59.0									
fasting plasma glucose	-3.10 (-5.11 to -1.09) mg / dL	9	594	72.0	HASAIN Z 2021[128]	probiotics supplementation after GDM diagnosis, regardless of gestational age, for at least a 4-week duration	at least 4-week	All RCTs	adult pregnant women regardless of weight status (normal, overweight, or obese) diagnosed with GDM according to oral glucose tolerance test and were not on any hypoglycemic agents	probiotics vs. placebo	MD	random	NP
insulin	-2.17 (-3.55 to -0.79) μIU/mL	7	450	72.0									
HOMA, homeostasis model assessment index	-0.56 (-0.86 to -0.26)	7	450	64.0									
QUICKI, quantitative insulin sensitivity check index	0.01 (0.00 to 0.01)	4	221	60.0									
total cholesterol level	-3.60 (-16.26 to 9.07) mg/dL	4	265	39.0									
high-density lipoprotein cholesterol	-1.25 (-5.48 to 2.99) mg/dL	4	265	59.0									
low-density lipoprotein cholesterol level	-6.36 (-14.89 to 2.16) mg/dL	4	265	0.0									
triglycerides levels	-10.40 (-24.68 to 3.89)mg/dL	4	265	0.0									
high-sensitivity C-reactive protein (hs-CRP)	-1.37 (-1.94 to -0.81) mg/L	4	275	24.0									
nitric oxide (NO)	2.42 (0.80 to 4.04) μmol/L	3	165	0.0									
malondialdehyde (MDA)	-0.77 (-0.99 to -0.56) μmol/L	4	221	0.0									
glutathione (GSH)	13.73 (-35.84 to 63.31) μmol/L	3	165	48.0									
total antioxidant capacity (TAC)	93.46 (-7.31 to 194.22) mmol/L	3	165	78.0									

incidence of pre-eclampsia	0.89 (0.32 to 2.46)	3	217	0.0						probiotics vs. control	OR		
gestational age at delivery	0.04 (-0.30 to 0.38) week	4	301	0.0						probiotics vs. placebo	MD		
the likelihood of cesarean delivery	0.66 (0.34 to 1.29)*	4	301	39.0							OR		
macrosomia	0.42 (0.19 to 0.94)	5	361	12.0									
newborns' hospitalization	0.37 (0.18 to 0.74)	4	301	6.0									
babies with hypoglycemia at birth	0.68 (0.28 to 1.64)*	3	174	0.0						probiotics vs. control			
Infant Eczema	0.60 (0.47 to 0.78)	10	2093	67.0	SUN M 2021[129]	a mixture of Lactobacillus and Bifidobacterium	0.5-2 years	All RCTs	healthy or pregnant women and infants under three years old with a family history of atopic disease	Vs. placebo intervention, and no other probiotic interventions or no intervention	RR	random	NP
Occurrence of Adverse Events	1.09 (0.83 to 1.44)*	6	1309	0.0									
the risk of eczema in children	0.90 (0.67 to 1.21)*	5	778	45.0	Szajewska H 2018[130]	Lactobacillus rhamnosus GG regardless of the timing of LGG administration	NP	All RCTs	healthy (1) pregnant women at high risk for having an allergic child; (2) breastfeeding mothers of infants at high risk of developing allergy; or (3) healthy term infants at high risk of developing allergy	vs. placebo or no intervention	RR	random	NA
Side effects													
Incidence of adverse events	0.83 (0.71 to 0.97)	32	4329/8305	49.0	GOLDENBERG J Z 2017[38]	probiotics	NP	All RCTs	participants taking antibiotics	any versus none	RR	random	No serious bias
Adverse Events	0.97 (0.86 to 1.09)*	15	6431	NP	SHEN N T 2017[40]	probiotics	NP	All RCTs	Hospitalized adults (18 years or older)	any versus none	RR	random	NP

									receiving antibiotic therapy (intravenous, oral, or both) for any reason				
Incidence of Adverse Events	0.82(0.65 to 1.05)*	17	166/1783	18.0	JOHNSTON B C 2012[41]	any dose of a specified probiotic of any strain	NP	All RCTs	adult or pediatric patients treated with antibiotics	any versus none	RR	random	No serious bias
the side effects observed in EcN treatment.	1.44(0.80 to 2.59)*	6	719	48.0	LOSURDO G 2015[46]	Escherichia coli Nissle (EcN) 1917	NP	All RCTs	Patients with UC	EcN versus standard of care (mesalazine or ciprofloxacin)	OR	Random	No serious bias
Adverse events	0.00 (-0.01 to 0.01)*	24	4415	75.0	GUO Q 2019 [50]	specific, identified probiotic in any form (e.g. capsule, sachet, yogurt). Trials investigating non-specific probiotic or yogurt agents (e.g. products that do not label the probiotic strain and dose) were not considered	5 days to 4 weeks	All RCTs	children (0 to 18 years) receiving antibiotics	Regimen or placebo Probiotic Versus Control	RD(RD: Risk difference)	Random	No serious bias
Incidence of Adverse Events	0.00 (-0.02 to 0.02)*	7	1183/2363	66.0	BLAABJERGS 2017[52]	Probiotics	NA	All RCTs	outpatients taking oral antibiotics	probiotics vs. Placebo or no treatment	RD (risk difference)	random	NP
Adverse events - Overt hepatic encephalopathy	0.29(0.16 to 0.51)*	10	585	0.0	DALAL R 2017 [10]	probiotics	follow-up: 2 weeks to 3 months	randomised clinical trials	people with any grade of acute or chronic hepatic encephalopathy	probiotics in any dosage vs. placebo or no intervention, or with any other treatment in people	RR	random	NP
Adverse events - Change of/or withdrawal from treatment	0.70(0.46 to 1.07)*	9	551	11.0			follow-up: 1 month to 3						

							months			with hepatic encephalopathy.			
any adverse event	1.09 (0.91 to 1.29)*	36	4183	36.0	FORD A C 2018[56]	probiotics	Minimum treatment duration of 7 days<br>Minimum follow-up duration of 7 days	All RCTs	Adults (participants aged >16 years)	probiotics vs. Control group	RR	random	0.08
Adverse events in active UC	1.21 (0.64 to 2.27)*	6	281/507	-	DERWA Y 2017 [54]	probiotics	NP	All RCTs	Adults (>90% of patients aged >16 years) with inflammatory bowel disease	probiotics vs. placebo	RR	random	NA
Adverse events in quiescent UC	1.09 (0.71 to 1.67)*	3	277/555	-						Probiotics vs. 5-ASA			
Adverse events in CD in remission following a surgical resection	0.81 (0.61 tp 1.08)*	3	129/263	-						probiotics vs. placebo			
the incidence of adverse effect	0.80 (0.43 to 1.50)*	10	762	0.0	YEH T L 2018[91]	probiotics or prebiotics	NP	Control trials	adults	probiotics or prebiotics vs. placebo	OR	random	No serious bias
Number of adverse events, dichotomous outcome	-0.00 (-0.03 to 0.03)*	6	279	0.0	SEILER C L 2020[75]	Probiotics of any type and dose, administered orally in any form (drink/<br>powder/capsule)	at least 2 weeks	All RCTs	Patients with Celiac disease (CD)	probiotics vs placebo	MD	random	NP
Adverse events	0.77 (0.47 to 1.26)*	4	355	0.0	WILSON B 2019[70]	prebiotics	2-12 weeks	All RCTs	Adult patients ≥18 and ≤64 y with Irritable bowel syndrome (IBS) or other Functional bowel disorders (FBDs)	vs. placebo	OR	random	No serious bias

Adverse events	0.27 (0.07 to 1.05)*	5	409	0.0	Zhang 2020[76]	J	prebiotics, probiotics, and synbiotics	At least 7 days	All RCTs	adult patients (over the age of 16 years) with functional dyspepsia (FD)	Vs. placebo	RR	fixed	NP
adverse events	0.72 (0.55 to 0.94)	30	2430	NP	Sohail 2018[72]	G	probiotic Medilac-S	4-96 weeks	All RCTs	human	Vs. control	RR	fixed	No serious bias

* No statistical significance; CI, confidence interval; RCT, randomized controlled trial; RR, relative risk; HR, hazard ratio; MD, mean difference; SMD, standard mean difference; WMD, weighted mean difference; OR, odds ratio; NA, not available; NP, not published.

References

1. Beserra, B.T.S.; Fernandes, R.; Do Rosario, V.A.; Mocellin, M.C.; Kuntz, M.G.F.; Trindade, E.B.S.M. A systematic review and meta-analysis of the prebiotics and synbiotics effects on glycaemia, insulin concentrations and lipid parameters in adult patients with overweight or obesity. *Clin. Nutr.* **2015**, *34*, 845-858, doi: 10.1016/j.clnu.2014.10.004.
2. Chen, X.; Hu, Y.; Yuan, X.; Yang, J.; Ka, L. Effect of early enteral nutrition combined with probiotics in patients with stroke: a meta-analysis of randomized controlled trials. *Eur. J. Clin. Nutr.* **2022**, *76*, 592-603, doi: 10.1038/s41430-021-00986-3.
3. Yan, S.M.; Tian, Z.W.; Li, M.; Li, B.; Cui, W.W. Effects of probiotic supplementation on the regulation of blood lipid levels in overweight or obese subjects: a meta-analysis. *Food Funct.* **2019**, *10*, 1747-1759, doi: 10.1039/c8fo02163e.
4. Wang, Z.; Xin, S.; Ding, L.; Ding, W.; Hou, Y.; Liu, C.; Zhang, X. The Potential Role of Probiotics in Controlling Overweight/Obesity and Associated Metabolic Parameters in Adults: A Systematic Review and Meta-Analysis. *Evid.-Based Compl. Alt.* **2019**, *2019*, 1-14, doi: 10.1155/2019/3862971.
5. Pontes, K.S.D.S.; Guedes, M.R.; Cunha, M.R.D.; Mattos, S.D.S.; Barreto Silva, M.I.; Neves, M.F.; Marques, B.C.A.A.; Klein, M.R.S.T. Effects of probiotics on body adiposity and cardiovascular risk markers in individuals with overweight and obesity: A systematic review and meta-analysis of randomized controlled trials. *Clin. Nutr.* **2021**, *40*, 4915-4931, doi: 10.1016/j.clnu.2021.06.023.
6. Rouhani, M.H.; Hadi, A.; Ghaedi, E.; Salehi, M.; Mahdavi, A.; Mohammadi, H. Do probiotics, prebiotics and synbiotics affect adiponectin and leptin in adults? A systematic review and meta-analysis of clinical trials. *Clin. Nutr.* **2019**, *38*, 2031-2037, doi: 10.1016/j.clnu.2018.09.033.
7. Cozzolino, M.; Vitagliano, A.; Pellegrini, L.; Chiurazzi, M.; Andriasani, A.; Ambrosini, G.; Garrido, N. Therapy with probiotics and synbiotics for polycystic ovarian syndrome: a systematic review and meta-analysis. *Eur. J. Nutr.* **2020**, *59*, 2841-2856, doi: 10.1007/s00394-020-02233-0.

8. Li, Y.; Tan, Y.; Xia, G.; Shuai, J. Effects of probiotics, prebiotics, and synbiotics on polycystic ovary syndrome: a systematic review and meta-analysis. *Crit. Rev. Food Sci.* **2021**, 1-17, doi: 10.1080/10408398.2021.1951155.
9. Den, H.; Dong, X.; Chen, M.; Zou, Z. Efficacy of probiotics on cognition, and biomarkers of inflammation and oxidative stress in adults with Alzheimer's disease or mild cognitive impairment — a meta-analysis of randomized controlled trials. *Aging* **2020**, *12*, 4010-4039, doi: 10.18632/aging.102810.
10. Dalal, R.; Mcgee, R.G.; Riordan, S.M.; Webster, A.C. Probiotics for people with hepatic encephalopathy. *Cochrane Db. Syst. Rev.* **2021**, *2021*, doi: 10.1002/14651858.CD008716.pub3.
11. Cao, Q.; Yu, C.; Yang, S.; Cao, H.; Chen, P.; Deng, M.; Li, L. Effect of probiotic treatment on cirrhotic patients with minimal hepatic encephalopathy: A meta-analysis. *Hepatob. Pancreat. Dis.* **2018**, *17*, 9-16, doi: 10.1016/j.hbpd.2018.01.005.
12. Khalesi, S.; Johnson, D.W.; Campbell, K.; Williams, S.; Fenning, A.; Saluja, S.; Irwin, C. Effect of probiotics and synbiotics consumption on serum concentrations of liver function test enzymes: a systematic review and meta-analysis. *Eur. J. Nutr.* **2018**, *57*, 2037-2053, doi: 10.1007/s00394-017-1568-y.
13. Loman, B.R.; Hernández-Saavedra, D.; An, R.; Rector, R.S. Prebiotic and probiotic treatment of nonalcoholic fatty liver disease: a systematic review and meta-analysis. *Nutr. Rev.* **2018**, *76*, 822-839, doi: 10.1093/nutrit/nuy031.
14. Khan, M.Y.; Mihali, A.B.; Rawala, M.S.; Aslam, A.; Siddiqui, W.J. The promising role of probiotic and synbiotic therapy in aminotransferase levels and inflammatory markers in patients with nonalcoholic fatty liver disease – a systematic review and meta-analysis. *Eur. J. Gastroen. Hepat.* **2019**, *31*, 703-715, doi: 10.1097/MEG.0000000000001371.
15. Skonieczna-żydecka, K.; Kaczmarczyk, M.; łoniewski, I.; Lara, L.; Koulaouzidis, A.; Misera, A.; Maciejewska, D.; Marlicz, W. A Systematic Review, Meta-Analysis, and Meta-Regression Evaluating the Efficacy and Mechanisms of Action of Probiotics and Synbiotics in the Prevention of Surgical Site Infections and Surgery-Related Complications. *Journal of Clinical Medicine* **2018**, *7*, 556, doi: 10.3390/jcm7120556.
16. Milajerdi, A.; Mousavi, S.M.; Sadeghi, A.; Salari-Moghaddam, A.; Parohan, M.; Larijani, B.; Esmaillzadeh, A. The effect of probiotics on inflammatory biomarkers: a meta-analysis of randomized clinical trials. *Eur. J. Nutr.* **2020**, *59*, 633-649, doi: 10.1007/s00394-019-01931-8.
17. Pan, H.; Li, R.; Li, T.; Wang, J.; Liu, L. Whether Probiotic Supplementation Benefits Rheumatoid Arthritis Patients: A Systematic Review and Meta-Analysis. *Engineering* **2017**, *3*, 115-121, doi: 10.1016/J.ENG.2017.01.006.
18. Fu, Y.; Chu, Q.; Ashuro, A.A.; Di, D.; Zhang, Q.; Liu, X.; Fan, Y. The Effect of Probiotics, Prebiotics, and Synbiotics on CD4 Counts in HIV-Infected Patients: A Systematic Review and Meta-Analysis. *Biomed Res. Int.* **2020**, *2020*, 1-11, doi: 10.1155/2020/7947342.
19. Zhang, X.; Chen, M.; Geng, S.; Yu, J.; Kuang, Y.; Luo, H.; Wang, K. Effects of Probiotics on Diarrhea and CD4 Cell Count in People Living With HIV: A Systematic Review and Meta-Analysis. *Frontiers in Pharmacology* **2021**, *12*, 570520, doi: 10.3389/fphar.2021.570520.
20. Tavakoly, R.; Hadi, A.; Rafie, N.; Talaei, B.; Marx, W.; Arab, A. Effect of Probiotic Consumption on Immune Response in Athletes: A Meta-analysis. *Int. J. Sports Med.* **2021**, *42*, 769-781, doi: 10.1055/a-1463-3303.
21. Askari, G.; Ghavami, A.; Shahdadian, F.; Moravejolahkami, A.R. Effect of synbiotics and probiotics supplementation on autoimmune diseases: A systematic review and meta-analysis of clinical trials. *Clin. Nutr.* **2021**, *40*, 3221-3234, doi: 10.1016/j.clnu.2021.02.015.
22. Jia, L.; Jia, Q.; Yang, J.; Jia, R.; Zhang, H. Efficacy of Probiotics Supplementation On Chronic Kidney Disease: a Systematic Review and Meta-Analysis. *Kidney and Blood Pressure Research* **2018**, *43*, 1623-1635, doi: 10.1159/000494677.

23. Tao, S.B.; Tao, S.Y.; Cheng, Y.M.; Liu, J.; Ma, L.; Fu, P. Effects of probiotic supplements on the progression of chronic kidney disease: A meta-analysis. *Nephrology* **2019**, *24*, 1122-1130, doi: 10.1111/nep.13549.
24. Thongprayoon, C.; Kaewput, W.; Hatch, S.T.; Bathini, T.; Sharma, K.; Wijarnpreecha, K.; Ungprasert, P.; D Costa, M.; Mao, M.A.; Cheungpasitporn, W. Effects of Probiotics on Inflammation and Uremic Toxins Among Patients on Dialysis: A Systematic Review and Meta-Analysis. *Digest. Dis. Sci.* **2019**, *64*, 469-479, doi: 10.1007/s10620-018-5243-9.
25. Bakhtiary, M.; Morvaridzadeh, M.; Agah, S.; Rahimlou, M.; Christopher, E.; Zadro, J.R.; Heshmati, J. Effect of Probiotic, Prebiotic, and Synbiotic Supplementation on Cardiometabolic and Oxidative Stress Parameters in Patients With Chronic Kidney Disease: A Systematic Review and Meta-analysis. *Clin. Ther.* **2021**, *43*, e71-e96, doi: 10.1016/j.clinthera.2020.12.021.
26. Mcfarlane, C.; Ramos, C.I.; Johnson, D.W.; Campbell, K.L. Prebiotic, Probiotic, and Synbiotic Supplementation in Chronic Kidney Disease: A Systematic Review and Meta-analysis. *J. Renal Nutr.* **2019**, *29*, 209-220, doi: 10.1053/j.jrn.2018.08.008.
27. Bock, P.M.; Telo, G.H.; Ramalho, R.; Sbaraini, M.; Leivas, G.; Martins, A.F.; Schaan, B.D. The effect of probiotics, prebiotics or synbiotics on metabolic outcomes in individuals with diabetes: a systematic review and meta-analysis. *Diabetologia* **2021**, *64*, 26-41, doi: 10.1007/s00125-020-05295-1.
28. Zheng, H.J.; Guo, J.; Jia, Q.; Huang, Y.S.; Huang, W.; Zhang, W.; Zhang, F.; Liu, W.J.; Wang, Y. The effect of probiotic and synbiotic supplementation on biomarkers of inflammation and oxidative stress in diabetic patients: A systematic review and meta-analysis of randomized controlled trials. *Pharmacol. Res.* **2019**, *142*, 303-313, doi: 10.1016/j.phrs.2019.02.016.
29. Tao, Y.; Gu, Y.; Mao, X.; Zhang, L.; Pei, Y. Effects of probiotics on type II diabetes mellitus: a meta-analysis. *J. Transl. Med.* **2020**, *18*, 30, doi: 10.1186/s12967-020-02213-2.
30. Chi, C.; Li, C.; Wu, D.; Buys, N.; Wang, W.; Fan, H.; Sun, J. Effects of Probiotics on Patients with Hypertension: a Systematic Review and Meta-Analysis. *Curr. Hypertens. Rep.* **2020**, *22*, doi: 10.1007/s11906-020-01042-4.
31. Hadi, A.; Arab, A.; Khalesi, S.; Rafie, N.; Kafeshani, M.; Kazemi, M. Effects of probiotic supplementation on anthropometric and metabolic characteristics in adults with metabolic syndrome: A systematic review and meta-analysis of randomized clinical trials. *Clin. Nutr.* **2021**, *40*, 4662-4673, doi: 10.1016/j.clnu.2021.05.027.
32. As Habi, A.; Nazari, M.; Hajianfar, H.; Arab, A.; Faghfoori, Z. A systematic review and meta-analysis of probiotic consumption and metabolic status of athletes. *Int. J. Food Prop.* **2020**, *23*, 941-954, doi: 10.1080/10942912.2020.1772286.
33. Malmir, H.; Ejtahed, H.; Soroush, A.; Mortazavian, A.M.; Fahimfar, N.; Ostovar, A.; Esmaillzadeh, A.; Larijani, B.; Hasani-Ranjbar, S. Probiotics as a New Regulator for Bone Health: A Systematic Review and Meta-Analysis. *Evid.-Based Compl. Alt.* **2021**, *2021*, 1-35, doi: 10.1155/2021/3582989.
34. Mcloughlin, R.F.; Berthon, B.S.; Jensen, M.E.; Baines, K.J.; Wood, L.G. Short-chain fatty acids, prebiotics, synbiotics, and systemic inflammation: a systematic review and meta-analysis. *The American Journal of Clinical Nutrition* **2017**, *106*, n156265, doi: 10.3945/ajcn.117.156265.
35. Da Silva Borges, D.; Fernandes, R.; Thives Mello, A.; Da Silva Fontoura, E.; Soares Dos Santos, A.R.; Santos De Moraes Trindade, E.B. Prebiotics may reduce serum concentrations of C-reactive protein and ghrelin in overweight and obese adults: a systematic review and meta-analysis. *Nutr. Rev.* **2019**, *78*, 235-248, doi: 10.1093/nutrit/nuz045.
36. Qu, H.; Zhang, Y.; Chai, H.; Gao, Z.; Shi, D. Effects of microbiota-driven therapy on inflammatory responses in elderly individuals: A systematic review and meta-analysis. *PLoS One* **2019**, *14*, e211233, doi: 10.1371/journal.pone.0211233.
37. Zhu, H.; Ren, Z.; Zang, Y.; Hua, H.; Lu, J.; Xu, Q.; Zhu, S. Effects of Microecological Preparations on Obese Patients after Bariatric Surgery: A Systematic Review and Meta-Analysis. *Evid.-Based Compl. Alt.* **2020**, *2020*, 1-10, doi: 10.1155/2020/8724546.

38. Goldenberg, J.Z.; Yap, C.; Lytvyn, L.; Lo, C.K.; Beardsley, J.; Mertz, D.; Johnston, B.C. Probiotics for the prevention of Clostridium difficile-associated diarrhea in adults and children. *Cochrane Db. Syst. Rev.* **2017**, *2017*, D6095, doi: 10.1002/14651858.CD006095.pub4.
39. Wu, Z.J.; DU X; Zheng, J. Role of Lactobacillus in the prevention of Clostridium difficile-associated diarrhea: a meta-analysis of randomized controlled trials. *Chin Med J (Engl)* **2013**, *126*, 4154-4161.
40. Shen, N.T.; Maw, A.; Tmanova, L.L.; Pino, A.; Ancy, K.; Crawford, C.V.; Simon, M.S.; Evans, A.T. Timely Use of Probiotics in Hospitalized Adults Prevents Clostridium difficile Infection: A Systematic Review With Meta-Regression Analysis. *Gastroenterology* **2017**, *152*, 1889-1900, doi: 10.1053/j.gastro.2017.02.003.
41. Johnston, B.C.; Ma, S.S.Y.; Goldenberg, J.Z.; Thorlund, K.; Vandvik, P.O.; Loeb, M.; Guyatt, G.H. Probiotics for the Prevention ofClostridium difficile – Associated Diarrhea. *Ann. Intern. Med.* **2012**, *157*, 878, doi: 10.7326/0003-4819-157-12-201212180-00563.
42. Dang, X.; Xu, M.; Liu, D.; Zhou, D.; Yang, W. Assessing the efficacy and safety of fecal microbiota transplantation and probiotic VSL#3 for active ulcerative colitis: A systematic review and meta-analysis. *PLoS One* **2020**, *15*, e228846, doi: 10.1371/journal.pone.0228846.
43. Kaur, L.; Gordon, M.; Baines, P.A.; Iheozor-Ejiofor, Z.; Sinopoulou, V.; Akobeng, A.K. Probiotics for induction of remission in ulcerative colitis. *Cochrane Db. Syst. Rev.* **2020**, *2020*, D5573, doi: 10.1002/14651858.CD005573.pub3.
44. Rufino, M.N.; Da Costa, A.L.; Jorge, E.N.; Paiano, V.F.; Camparoto, M.L.; Keller, R.; Bremer-Neto, H. Synbiotics improve clinical indicators of ulcerative colitis: systematic review with meta-analysis. *Nutr. Rev.* **2022**, *80*, 157-164, doi: 10.1093/nutrit/nuab017.
45. Peng, L.; Zhong, Y.; Wang, A.; Jiang, Z. Probiotics combined with aminosalicylic acid affiliates remission of ulcerative colitis: a meta-analysis of randomized controlled trial. *Bioscience Rep.* **2019**, *39*, doi: 10.1042/BSR20180943.
46. Losurdo, G.; Iannone, A.; Contaldo, A.; Ierardi, E.; Di Leo, A.; Principi, M. Escherichia coli Nissle 1917 in Ulcerative Colitis Treatment: Systematic Review and Meta-analysis. *J. Gastrointest. Liver* **2020**, *24*, 499-505, doi: 10.15403/jgld.2014.1121.244.ecn.
47. Iheozor-Ejiofor, Z.; Kaur, L.; Gordon, M.; Baines, P.A.; Sinopoulou, V.; Akobeng, A.K. Probiotics for maintenance of remission in ulcerative colitis. *Cochrane Db. Syst. Rev.* **2020**, *3*, D7443, doi: 10.1002/14651858.CD007443.pub3.
48. Astó, E.; Méndez, I.; Audivert, S.; Farran-Codina, A.; Espadaler, J. The Efficacy of Probiotics, Prebiotic Inulin-Type Fructans, and Synbiotics in Human Ulcerative Colitis: A Systematic Review and Meta-Analysis. *Nutrients* **2019**, *11*, 293, doi: 10.3390/nu11020293.
49. Goodman, C.; Keating, G.; Georgousopoulou, E.; Hespe, C.; Levett, K. Probiotics for the prevention of antibiotic-associated diarrhoea: a systematic review and meta-analysis. *BMJ Open* **2021**, *11*, e43054, doi: 10.1136/bmjopen-2020-043054.
50. Guo, Q.; Goldenberg, J.Z.; Humphrey, C.; Dib, R.E.; Johnston, B. PROBIOTICS FOR THE PREVENTION OF PEDIATRIC ANTIBIOTIC-ASSOCIATED DIARRHEA. *Gastroenterology* **2019**, *156*, 220, doi: 10.1016/S0016-5085%2819%2937348-2.
51. Jafarnejad, S.; Shab-Bidar, S.; Speakman, J.R.; Parastui, K.; Daneshi-Maskooni, M.; Djafarian, K. Probiotics Reduce the Risk of Antibiotic-Associated Diarrhea in Adults (18–64 Years) but Not the Elderly (>65 Years). *Nutr. Clin. Pract.* **2016**, *31*, 502-513, doi: 10.1177/0884533616639399.
52. Blaabjerg, S.; Artzi, D.; Aabenhus, R. Probiotics for the Prevention of Antibiotic-Associated Diarrhea in Outpatients—A Systematic Review and Meta-Analysis. *Antibiotics* **2017**, *6*, 21, doi: 10.3390/antibiotics6040021.
53. Zhang, X.; Guan, X.; Tang, Y.; Sun, J.; Wang, X.; Wang, W.; Fan, J. Clinical effects and gut microbiota changes of using probiotics, prebiotics or synbiotics in inflammatory bowel disease: a systematic review and meta-analysis. *Eur. J. Nutr.* **2021**, *60*, 2855-2875, doi: 10.1007/s00394-021-02503-5.

54. Derwa, Y.; Gracie, D.J.; Hamlin, P.J.; Ford, A.C. Systematic review with meta-analysis: the efficacy of probiotics in inflammatory bowel disease. *Aliment. Pharm. Ther.* **2017**, *46*, 389-400, doi: 10.1111/apt.14203.
55. Pabón-Carrasco, M.; Ramirez-Baena, L.; Vilar-Palomo, S.; Castro-Méndez, A.; Martos-García, R.; Rodríguez-Gallego, I. Probiotics as a Coadjuvant Factor in Active or Quiescent Inflammatory Bowel Disease of Adults—A Meta-Analytical Study. *Nutrients* **2020**, *12*, 2628, doi: 10.3390/nu12092628.
56. Ford, A.C.; Harris, L.A.; Lacy, B.E.; Quigley, E.M.M.; Moayyedi, P. Systematic review with meta-analysis: the efficacy of prebiotics, probiotics, synbiotics and antibiotics in irritable bowel syndrome. *Aliment. Pharm. Ther.* **2018**, *48*, 1044-1060, doi: 10.1111/apt.15001.
57. Asha, M.Z.; Khalil, S.F.H. Efficacy and Safety of Probiotics, Prebiotics and Synbiotics in the Treatment of Irritable Bowel Syndrome: A systematic review and meta-analysis. *Sultan Qaboos University Medical Journal [SQUMJ]* **2020**, *20*, 13-24, doi: 10.18295/squmj.2020.20.01.003.
58. Xu, H.; Zou, L.; Chen, M.; Wang, H.; Shen, W.; Zheng, Q.; Cui, W. Efficacy of probiotic adjuvant therapy for irritable bowel syndrome in children: A systematic review and meta-analysis. *PLoS One* **2021**, *16*, e255160, doi: 10.1371/journal.pone.0255160.
59. Connell, M.; Shin, A.; James-Stevenson, T.; Xu, H.; Imperiale, T.F.; Herron, J. Systematic review and meta-analysis: Efficacy of patented probiotic, VSL#3, in irritable bowel syndrome. *Neurogastroenterology & Motility* **2018**, *30*, e13427, doi: 10.1111/nmo.13427.
60. Yuan, F.; Ni, H.; Asche, C.V.; Kim, M.; Walayat, S.; Ren, J. Efficacy ofBifidobacterium infantis 35624 in patients with irritable bowel syndrome: a meta-analysis. *Curr. Med. Res. Opin.* **2017**, *33*, 1191-1197, doi: 10.1080/03007995.2017.1292230.
61. Wen, Y.; Li, J.; Long, Q.; Yue, C.; He, B.; Tang, X. The efficacy and safety of probiotics for patients with constipation-predominant irritable bowel syndrome: A systematic review and meta-analysis based on seventeen randomized controlled trials. *Int. J. Surg.* **2020**, *79*, 111-119, doi: 10.1016/j.ijsu.2020.04.063.
62. Wu, X.; Xu, W.; Liu, M.; Hu, K.; Sun, Y.; Yang, X.; Zhu, G.; Wang, Z.; Huang, W. Efficacy of prophylactic probiotics in combination with antibiotics versus antibiotics alone for colorectal surgery: A meta-analysis of randomized controlled trials. *J. Surg. Oncol.* **2018**, *117*, 1394-1404, doi: 10.1002/jso.25038.
63. Zhong, C.; Qu, C.; Wang, B.; Liang, S.; Zeng, B. Probiotics for Preventing and Treating Small Intestinal Bacterial Overgrowth. *J. Clin. Gastroenterol.* **2017**, *51*, 300-311, doi: 10.1097/MCG.0000000000000814.
64. Zhang, C.; Jiang, J.; Tian, F.; Zhao, J.; Zhang, H.; Zhai, Q.; Chen, W. Meta-analysis of randomized controlled trials of the effects of probiotics on functional constipation in adults. *Clin. Nutr.* **2020**, *39*, 2960-2969, doi: 10.1016/j.clnu.2020.01.005.
65. Huang, R.; Hu, J. Positive Effect of Probiotics on Constipation in Children: A Systematic Review and Meta-Analysis of Six Randomized Controlled Trials. *Front. Cell. Infect. Mi.* **2017**, *7*, 153, doi: 10.3389/fcimb.2017.00153.
66. Losurdo, G.; Cubisino, R.; Barone, M.; Principi, M.; Leandro, G.; Ierardi, E.; Leo, A.D. Probiotic monotherapy andHelicobacter pylori eradication: A systematic review with pooled-data analysis. *World J. Gastroentero.* **2018**, *24*, 139-149, doi: 10.3748/wjg.v24.i1.139.
67. Ji, T.; Zhu, X.; Shang, F.; Zhang, X. Preventive Effect of Probiotics on Ventilator-Associated Pneumonia: A Meta-analysis of 2428 Patients. *Ann. Pharmacother.* **2021**, *55*, 949-962, doi: 10.1177/1060028020983021.
68. Rao, S.; Srinivasjois, R.; Patole, S. Prebiotic Supplementation in Full-term Neonates. *Archives of Pediatrics & Adolescent Medicine* **2009**, *163*, 755-764, doi: 10.1001/archpediatrics.2009.94.
69. Lohner, S.; Küllenberg, D.; Antes, G.; Decsi, T.; Meerpohl, J.J. Prebiotics in healthy infants and children for prevention of acute infectious diseases: a systematic review and meta-analysis. *Nutr. Rev.* **2014**, *72*, 523-531, doi: 10.1111/nure.12117.

70. Wilson, B.; Rossi, M.; Dimidi, E.; Whelan, K. Prebiotics in irritable bowel syndrome and other functional bowel disorders in adults: a systematic review and meta-analysis of randomized controlled trials. *The American Journal of Clinical Nutrition* **2019**, *109*, 1098-1111, doi: 10.1093/ajcn/nqy376.
71. Chi, C.; Buys, N.; Li, C.; Sun, J.; Yin, C. Effects of prebiotics on sepsis, necrotizing enterocolitis, mortality, feeding intolerance, time to full enteral feeding, length of hospital stay, and stool frequency in preterm infants: a meta-analysis. *Eur. J. Clin. Nutr.* **2019**, *73*, 657-670, doi: 10.1038/s41430-018-0377-6.
72. Sohail, G.; Xu, X.; Christman, M.C.; Tompkins, T.A. Probiotic Medilac-S® for the induction of clinical remission in a Chinese population with ulcerative colitis: A systematic review and meta-analysis. *World Journal of Clinical Cases* **2018**, *6*, 961-984, doi: 10.12998/wjcc.v6.i15.961.
73. Nakamura, H.; Lim, T.; Puri, P. Probiotics for the prevention of Hirschsprung-associated enterocolitis: a systematic review and meta-analysis. *Pediatr. Surg. Int.* **2018**, *34*, 189-193, doi: 10.1007/s00383-017-4188-y.
74. Miller, L.E.; Zimmermann, A.K.; Ouwehand, A.C. Contemporary meta-analysis of short-term probiotic consumption on gastrointestinal transit. *World J. Gastroentero.* **2016**, *22*, 5122, doi: 10.3748/wjg.v22.i21.5122.
75. Seiler, C.L.; Kiflen, M.; Stefanolo, J.P.; Bai, J.C.; Bercik, P.; Kelly, C.P.; Verdu, E.F.; Moayyedi, P.; Pinto-Sanchez, M.I. Probiotics for Celiac Disease: A Systematic Review and Meta-Analysis of Randomized Controlled Trials. *Am. J. Gastroenterol.* **2020**, *115*, 1584-1595, doi: 10.14309/ajg.0000000000000749.
76. Zhang, J.; Wu, H.M.; Wang, X.; Xie, J.; Li, X.; Ma, J.; Wang, F.; Tang, X. Efficacy of prebiotics and probiotics for functional dyspepsia. *Medicine* **2020**, *99*, e19107, doi: 10.1097/MD.0000000000019107.
77. Sorensen, K.; Cawood, A.L.; Gibson, G.R.; Cooke, L.H.; Stratton, R.J. Amino Acid Formula Containing Synbiotics in Infants with Cow's Milk Protein Allergy: A Systematic Review and Meta-Analysis. *Nutrients* **2021**, *13*, 935, doi: 10.3390/nu13030935.
78. Suzumura, E.A.; Bersch-Ferreira, Â.C.; Torreglosa, C.R.; Da Silva, J.T.; Coqueiro, A.Y.; Kuntz, M.G.F.; Chrispim, P.P.; Weber, B.; Cavalcanti, A.B. Effects of oral supplementation with probiotics or synbiotics in overweight and obese adults: a systematic review and meta-analyses of randomized trials. *Nutr. Rev.* **2019**, *77*, 430-450, doi: 10.1093/nutrit/nuz001.
79. Mohammadi, H.; Ghavami, A.; Faghihimani, Z.; Sharifi, S.; Nattagh-Eshtivani, E.; Ziaei, R.; Miraghajani, M. Effects of probiotics fermented milk products on obesity measure among adults: A systematic review and meta-analysis of clinical trials. *J. Funct. Foods* **2021**, *82*, 104494, doi: 10.1016/j.jff.2021.104494.
80. Hadi, A.; Ghaedi, E.; Khalesi, S.; Pourmasoumi, M.; Arab, A. Effects of synbiotic consumption on lipid profile: a systematic review and meta-analysis of randomized controlled clinical trials. *Eur. J. Nutr.* **2020**, *59*, 2857-2874, doi: 10.1007/s00394-020-02248-7.
81. Zhang, G.; Hu, H.; Liu, C.; Zhang, Q.; Shakya, S.; Li, Z. Probiotics for Prevention of Atopy and Food Hypersensitivity in Early Childhood. *Medicine* **2016**, *95*, e2562, doi: 10.1097/MD.0000000000002562.
82. Coffey, M.J.; Garg, M.; Homaira, N.; Jaffe, A.; Ooi, C.Y. Probiotics for people with cystic fibrosis. *Cochrane Db. Syst. Rev.* **2020**, doi: 10.1002/14651858.CD012949.pub2.
83. Miller, L.; Lehtoranta, L.; Lehtinen, M. The Effect of Bifidobacterium animalis ssp. lactis HN019 on Cellular Immune Function in Healthy Elderly Subjects: Systematic Review and Meta-Analysis. *Nutrients* **2017**, *9*, 191, doi: 10.3390/nu9030191.
84. Miller, L.E.; Lehtoranta, L.; Lehtinen, M.J. Short-term probiotic supplementation enhances cellular immune function in healthy elderly: systematic review and meta-analysis of controlled studies. *Nutr. Res.* **2019**, *64*, 1-8, doi: 10.1016/j.nutres.2018.12.011.
85. Dermyshi, E.; Wang, Y.; Yan, C.; Hong, W.; Qiu, G.; Gong, X.; Zhang, T. The "Golden Age" of Probiotics: A Systematic Review and Meta-Analysis of Randomized and Observational Studies in Preterm Infants. *Neonatology* **2017**, *112*, 9-23, doi: 10.1159/000454668.
86. Aceti, A.; Maggio, L.; Beghetti, I.; Gori, D.; Barone, G.; Callegari, M.; Fantini, M.; Indrio, F.; Meneghin, F.; Morelli, L.; et al. Probiotics Prevent Late-Onset Sepsis in Human

Milk-Fed, Very Low Birth Weight Preterm Infants: Systematic Review and Meta-Analysis. *Nutrients* **2017**, *9*, 904, doi: 10.3390/nu9080904.
87. Sawas, T.; Al Halabi, S.; Hernaez, R.; Carey, W.D.; Cho, W.K. Patients Receiving Prebiotics and Probiotics Before Liver Transplantation Develop Fewer Infections Than Controls: A Systematic Review and Meta-Analysis. *Clin. Gastroenterol. H.* **2015**, *13*, 1567-1574, doi: 10.1016/j.cgh.2015.05.027.
88. Yang, Z.; Wu, Q.; Liu, Y.; Fan, D. Effect of Perioperative Probiotics and Synbiotics on Postoperative Infections After Gastrointestinal Surgery: A Systematic Review With Meta-Analysis. *Jpen.-Parenter. Enter.* **2017**, *41*, 1051-1062, doi: 10.1177/0148607116629670.
89. Le Morvan De Sequeira, C.; Kaeber, M.; Cekin, S.E.; Enck, P.; Mack, I. The Effect of Probiotics on Quality of Life, Depression and Anxiety in Patients with Irritable Bowel Syndrome: A Systematic Review and Meta-Analysis. *Journal of Clinical Medicine* **2021**, *10*, 3497, doi: 10.3390/jcm10163497.
90. Lei, W.; Shih, P.; Liu, S.; Lin, C.; Yeh, T. Effect of Probiotics and Prebiotics on Immune Response to Influenza Vaccination in Adults: A Systematic Review and Meta-Analysis of Randomized Controlled Trials. *Nutrients* **2017**, *9*, 1175, doi: 10.3390/nu9111175.
91. Yeh, T.; Shih, P.; Liu, S.; Lin, C.; Liu, J.; Lei, W.; Lin, C. The influence of prebiotic or probiotic supplementation on antibody titers after influenza vaccination: a systematic review and meta-analysis of randomized controlled trials. *Drug Des. Dev. Ther.* **2018**, *Volume 12*, 217-230, doi: 10.2147/DDDT.S155110.
92. Hendijani, F.; Akbari, V. Probiotic supplementation for management of cardiovascular risk factors in adults with type II diabetes: A systematic review and meta-analysis. *Clin. Nutr.* **2018**, *37*, 532-541, doi: 10.1016/j.clnu.2017.02.015.
93. Du, X.; Wang, L.; Wu, S.; Yuan, L.; Tang, S.; Xiang, Y.; Qu, X.; Liu, H.; Qin, X.; Liu, C. Efficacy of probiotic supplementary therapy for asthma, allergic rhinitis, and wheeze: a meta-analysis of randomized controlled trials. *Allergy Asthma Proc.* **2019**, *40*, 250-260, doi: 10.2500/aap.2019.40.4227.
94. Sun, S.; Chang, G.; Zhang, L. The prevention effect of probiotics against eczema in children: an update systematic review and meta-analysis. *J. Dermatol. Treat.* **2021**, 1-11, doi: 10.1080/09546634.2021.1925077.
95. Boyle, R.J.; Bath-Hextall, F.J.; Leonardi-Bee, J.; Murrell, D.F.; Tang, M.L. Probiotics for the treatment of eczema: a systematic review. *Clinical & Experimental Allergy* **2009**, *39*, 1117-1127, doi: 10.1111/j.1365-2222.2009.03305.x.
96. Wang, F.; Pan, B.; Xu, S.; Xu, Z.; Zhang, T.; Zhang, Q.; Bao, Y.; Wang, Y.; Zhang, J.; Xu, C.; et al. A meta-analysis reveals the effectiveness of probiotics and prebiotics against respiratory viral infection. *Bioscience Rep.* **2021**, *41*, R20203638, doi: 10.1042/BSR20203638.
97. Wang, Y.; Li, X.; Ge, T.; Xiao, Y.; Liao, Y.; Cui, Y.; Zhang, Y.; Ho, W.; Yu, G.; Zhang, T. Probiotics for prevention and treatment of respiratory tract infections in children. *Medicine* **2016**, *95*, e4509, doi: 10.1097/MD.0000000000004509.
98. Irwin, C.; Mccartney, D.; Ben, D.; Khalesi, S. Effects of probiotics and paraprobiotics on subjective and objective sleep metrics: a systematic review and meta-analysis. *Eur. J. Clin. Nutr.* **2020**, *74*, 1536-1549, doi: 10.1038/s41430-020-0656-x.
99. Jiang, J.; Chu, C.; Wu, C.; Wang, C.; Zhang, C.; Li, T.; Zhai, Q.; Yu, L.; Tian, F.; Chen, W. Efficacy of probiotics in multiple sclerosis: a systematic review of preclinical trials and meta-analysis of randomized controlled trials. *Food Funct.* **2021**, *12*, 2354-2377, doi: 10.1039/D0FO03203D.
100. Stachowska, E.; Portincasa, P.; Jamioł-Milc, D.; Maciejewska-Markiewicz, D.; Skonieczna-żydecka, K. The Relationship between Prebiotic Supplementation and Anthropometric and Biochemical Parameters in Patients with NAFLD—A Systematic Review and Meta-Analysis of Randomized Controlled Trials. *Nutrients* **2020**, *12*, 3460, doi: 10.3390/nu12113460.
101. Sharpton, S.R.; Maraj, B.; Harding-Theobald, E.; Vittinghoff, E.; Terrault, N.A. Gut microbiome–targeted therapies in nonalcoholic fatty liver disease: a systematic review, meta-analysis, and meta-regression. *The American Journal of Clinical Nutrition* **2019**, *110*, 139-149, doi: 10.1093/ajcn/nqz042.

102. Chang, Y.; Trivedi, M.K.; Jha, A.; Lin, Y.; Dimaano, L.; García-Romero, M.T. Synbiotics for Prevention and Treatment of Atopic Dermatitis. *JAMA Pediatr.* **2016**, *170*, 236, doi: 10.1001/jamapediatrics.2015.3943.
103. Kahn, J.; Pregartner, G.; Schemmer, P. Effects of both Pro- and Synbiotics in Liver Surgery and Transplantation with Special Focus on the Gut–Liver Axis—A Systematic Review and Meta-Analysis. *Nutrients* **2020**, *12*, 2461, doi: 10.3390/nu12082461.
104. Chan, C.K.Y.; Tao, J.; Chan, O.S.; Li, H.; Pang, H. Preventing Respiratory Tract Infections by Synbiotic Interventions: A Systematic Review and Meta-Analysis of Randomized Controlled Trials. *Adv. Nutr.* **2020**, *11*, 979-988, doi: 10.1093/advances/nmaa003.
105. Kinross, J.M.; Markar, S.; Karthikesalingam, A.; Chow, A.; Penney, N.; Silk, D.; Darzi, A. A Meta-Analysis of Probiotic and Synbiotic Use in Elective Surgery. *Jpen.-Parenter. Enter.* **2013**, *37*, 243-253, doi: 10.1177/0148607112452306.
106. Sharif, S.; Meader, N.; Oddie, S.J.; Rojas-Reyes, M.X.; Mcguire, W. Probiotics to prevent necrotising enterocolitis in very preterm or very low birth weight infants. *Cochrane Db. Syst. Rev.* **2020**, *2020*, D5496, doi: 10.1002/14651858.CD005496.pub5.
107. Yang, Y.; Guo, Y.; Kan, Q.; Zhou, X.G.; Zhou, X.Y.; Li, Y. A meta-analysis of probiotics for preventing necrotizing enterocolitis in preterm neonates. *Braz. J. Med. Biol. Res.* **2014**, *47*, 804-810, doi: 10.1590/1414-431X20143857.
108. Jiang, T.; Zhang, H.; Xu, X.; Li, H.; Yang, J. Mixed probiotics decrease the incidence of stage II-III necrotizing enterocolitis and death: A systematic review and meta-analysis. *Microb. Pathogenesis* **2020**, *138*, 103794, doi: 10.1016/j.micpath.2019.103794.
109. Liu, D.; Shao, L.; Zhang, Y.; Kang, W. Safety and efficacy of Lactobacillus for preventing necrotizing enterocolitis in preterm infants. *Int. J. Surg.* **2020**, *76*, 79-87, doi: 10.1016/j.ijsu.2020.02.031.
110. Zhu, X.; Tang, X.; Qu, F.; Zheng, Y.; Zhang, W.; Diao, Y. Bifidobacterium may benefit the prevention of necrotizing enterocolitis in preterm infants: A systematic review and meta-analysis. *Int. J. Surg.* **2019**, *61*, 17-25, doi: 10.1016/j.ijsu.2018.11.026.
111. Lv, T.; Ye, M.; Luo, F.; Hu, B.; Wang, A.; Chen, J.; Yan, J.; He, Z.; Chen, F.; Qian, C.; et al. Probiotics treatment improves cognitive impairment in patients and animals: A systematic review and meta-analysis. *Neuroscience & Biobehavioral Reviews* **2021**, *120*, 159-172, doi: 10.1016/j.neubiorev.2020.10.027.
112. Krüger, J.F.; Hillesheim, E.; Pereira, A.C.S.N.; Camargo, C.Q.; Rabito, E.I. Probiotics for dementia: a systematic review and meta-analysis of randomized controlled trials. *Nutr. Rev.* **2021**, *79*, 160-170, doi: 10.1093/nutrit/nuaa037.
113. Liu, B.; He, Y.; Wang, M.; Liu, J.; Ju, Y.; Zhang, Y.; Liu, T.; Li, L.; Li, Q. Efficacy of probiotics on anxiety-A meta-analysis of randomized controlled trials. *Depress. Anxiety* **2018**, *35*, 935-945, doi: 10.1002/da.22811.
114. Mckean, J.; Naug, H.; Nikbakht, E.; Amiet, B.; Colson, N. Probiotics and Subclinical Psychological Symptoms in Healthy Participants: A Systematic Review and Meta-Analysis. *The Journal of Alternative and Complementary Medicine* **2017**, *23*, 249-258, doi: 10.1089/acm.2016.0023.
115. Goh, K.K.; Liu, Y.; Kuo, P.; Chung, Y.E.; Lu, M.; Chen, C. Effect of probiotics on depressive symptoms: A meta-analysis of human studies. *Psychiat. Res.* **2019**, *282*, 112568, doi: 10.1016/j.psychres.2019.112568.
116. Ng, Q.X.; Soh, A.Y.S.; Venkatanarayanan, N.; Ho, C.Y.X.; Lim, D.Y.; Yeo, W. A Systematic Review of the Effect of Probiotic Supplementation on Schizophrenia Symptoms. *Neuropsychobiology* **2019**, *78*, 1-6, doi: 10.1159/000498862.
117. Zhang, N.; Zhang, Y.; Li, M.; Wang, W.; Liu, Z.; Xi, C.; Huang, X.; Liu, J.; Huang, J.; Tian, D.; et al. Efficacy of probiotics on stress in healthy volunteers: A systematic review and meta-analysis based on randomized controlled trials. *Brain and Behavior* **2020**, *10*, doi: 10.1002/brb3.1699.

118. Cohen Kadosh, K.; Basso, M.; Knytl, P.; Johnstone, N.; Lau, J.Y.F.; Gibson, G.R. Psychobiotic interventions for anxiety in young people: a systematic review and meta-analysis, with youth consultation. *Transl. Psychiat.* **2021**, *11*, doi: 10.1038/s41398-021-01422-7.
119. Liu, R.T.; Walsh, R.F.L.; Sheehan, A.E. Prebiotics and probiotics for depression and anxiety: A systematic review and meta-analysis of controlled clinical trials. *Neuroscience & Biobehavioral Reviews* **2019**, *102*, 13-23, doi: 10.1016/j.neubiorev.2019.03.023.
120. Ong, T.G.; Gordon, M.; Banks, S.S.; Thomas, M.R.; Akobeng, A.K. Probiotics to prevent infantile colic. *Cochrane Db. Syst. Rev.* **2019**, doi: 10.1002/14651858.CD012473.pub2.
121. Skonieczna-żydecka, K.; Janda, K.; Kaczmarczyk, M.; Marlicz, W.; łoniewski, I.; łoniewska, B. The Effect of Probiotics on Symptoms, Gut Microbiota and Inflammatory Markers in Infantile Colic: A Systematic Review, Meta-Analysis and Meta-Regression of Randomized Controlled Trials. *Journal of Clinical Medicine* **2020**, *9*, 999, doi: 10.3390/jcm9040999.
122. Upadhyay, R.P.; Taneja, S.; Chowdhury, R.; Strand, T.A.; Bhandari, N. Effect of prebiotic and probiotic supplementation on neurodevelopment in preterm very low birth weight infants: findings from a meta-analysis. *Pediatr. Res.* **2020**, *87*, 811-822, doi: 10.1038/s41390-018-0211-9.
123. Sung, V.; Collett, S.; de Gooyer, T.; Hiscock, H.; Tang, M.; Wake, M. Probiotics to Prevent or Treat Excessive Infant Crying. *JAMA Pediatr.* **2013**, *167*, 1150, doi: 10.1001/jamapediatrics.2013.2572.
124. Grev, J.; Berg, M.; Soll, R. Maternal probiotic supplementation for prevention of morbidity and mortality in preterm infants. *Cochrane Db. Syst. Rev.* **2018**, *2018*, D12519, doi: 10.1002/14651858.CD012519.pub2.
125. Wang, C.; Tung, Y.; Chang, H.; Lin, C.; Chen, Y. Effect of Probiotic Supplementation on Newborn Birth Weight for Mother with Gestational Diabetes Mellitus or Overweight/Obesity: A Systematic Review and Meta-Analysis. *Nutrients* **2020**, *12*, 3477, doi: 10.3390/nu12113477.
126. Han, M.; Sun, J.; Su, X.; Peng, Y.; Goyal, H.; Wu, C.; Zhu, X.; Li, L. Probiotics improve glucose and lipid metabolism in pregnant women: a meta-analysis. *Annals of Translational Medicine* **2019**, *7*, 99, doi: 10.21037/atm.2019.01.61.
127. Davidson, S.J.; Barrett, H.L.; Price, S.A.; Callaway, L.K.; Dekker Nitert, M. Probiotics for preventing gestational diabetes. *Cochrane Db. Syst. Rev.* **2021**, *2021*, D9951, doi: 10.1002/14651858.CD009951.pub3.
128. Hasain, Z.; Che Roos, N.A.; Rahmat, F.; Mustapa, M.; Raja Ali, R.A.; Mokhtar, N.M. Diet and Pre-Intervention Washout Modifies the Effects of Probiotics on Gestational Diabetes Mellitus: A Comprehensive Systematic Review and Meta-Analysis of Randomized Controlled Trials. *Nutrients* **2021**, *13*, 3045, doi: 10.3390/nu13093045.
129. Sun, M.; Luo, J.; Liu, H.; Xi, Y.; Lin, Q. Can Mixed Strains of Lactobacillus and Bifidobacterium Reduce Eczema in Infants under Three Years of Age? A Meta-Analysis. *Nutrients* **2021**, *13*, 1461, doi: 10.3390/nu13051461.
130. Szajewska, H.; Horvath, A. Lactobacillus rhamnosus GG in the Primary Prevention of Eczema in Children: A Systematic Review and Meta-Analysis. *Nutrients* **2018**, *10*, 1319, doi: 10.3390/nu10091319.
