# Supplementary material for: Gastrointestinal Microbiome and Multiple Health Outcomes: Umbrella Review
Source: Nutrients. 2022 Sep 9;14(18):3726. doi: 10.3390/nu14183726 (PMC9505003; doi:10.3390/nu14183726)
Supplement: Supplementary file 1 [file nutrients-14-03726-s001.zip › Supplementary Table S10.pdf]

Supplement Table S10. Other interventions and multiple health outcomes.

outcome(s) of interest	estimated summary effect (95% CI)	number of studies / total studies	number of intervention group or total participants	Heterogeneity (I^2, %)	the first author + year of publication	Intervention	Duration of Intervention/follow-up	study design	populations	outcome comparison	meta-analysis metric	type of effect model	publication bias	1.
improvement of rosacea-related skin symptoms	1.28 (0.98 to 1.67)*	7	NP	NP	JORGENSEN A 2017[1]	treatment of Helicobacter pylori infection	NP	Case-control, cross-sectional, cohort, or nested case-control design	rosacea patients	rosacea patients with control groups.	OR	random	NP	2.
fasting blood glucose (FPG)	-0.87 (-1.26 to -0.49)	4	348	75.0	ZHENG Y 2021[2]	Chinese herbal compounds, Chinese patent medicine, and single Chinese medical herbs, which can be administered in the form of decoctions, granules, or powders	8-12 weeks	All RCTs	human	Vs. individuals that were treated with either Western medicine or placebo, or those that did not undergo any intervention	MD	random	NP	3.
HbA1c	-0.69 (-1.24 to -0.14) %	4	253	86.0										4.
2-h postprandial blood glucose (2hPG)	-0.83 (-1.01 to -0.65)	3	253	0.0										5.
HOMA-IR	-0.99 (-1.25 to -0.73)	3	263	0.0							SMD	fixed		6.
risk of UC	1.09 (0.88 to 1.34)*	7	NP	0.0	MILAJERDI A 2021[3]	dietary fiber consumption	NP	prospective cohort or nested case-control studies	Human excluded pregnant women, children, or elderly people	Vs. without dietary fiber	RR	fixed	NP	7.
risk of CD	0.59 (0.46 to 0.74)	6		0.0										8.
risk of UC	0.69 (0.55 to 0.86)	4		87.0		Fruit consumption				those in the highest category of fruit intake vs. those in the lowest category				9.
risk of CD	0.47 (0.38 to 0.58)	4		32.1										10.
risk of UC	0.56 (0.48 to 0.66)	3		72.0		vegetable consumption				individuals in the top category of vegetable consumption vs. those in the bottom category				11.
risk of CD	0.52 (0.46 to 0.59)	3		78.9										12.
the incidence of Gastrointestinal acute graft-versus-host-disease	0.69 (0.56 to 0.86)	7	495	0.0	ZAMA D 2021[4]	Enternal nutrition (EN) with or without the addition of parenteral	NP	experimental and observationa	Human with allogeneic	Vs. only PN	RR	fixed	NP	13.

(aGvHD),						nutrition (PN) (EN group)		l original studies	hematopoietic stem cell transplantation (allo-HSCT)					
The incidence of aGvHD grade III-IV	0.44 (0.30 to 0.64)	5	522	0.0										14.
Gut aGvHD	0.44 (0.30 to 0.66)	4	396	6.0										15.
the incidence of mucositis grade III-IV	0.95 (0.83 to 1.09)*	5	530	0.0										16.
overall survival at day +100	1.07 (0.95 to 1.21)*	5	530	63.0								random		17.
blood urea nitrogen (BUN)	-6.91 (-11.87 to -1.95)	4	148	0.0	JIA L 2021[5]	resistant starch type 2	NP	RCTs regardless of a design of blindness	adult CKD patients receiving regular hemodialysis	With vs. Without resistant starch type 2	WMD	random	NP	18.
serum creatinine (Scr)	-7.43 (-11.99 to -2.86)	3	119	44.0										19.
uric acid (UA)	0.17 (-0.23 to 0.58)*	3	108	0.0										20.
indoxyl sulfate (IS)	-0.33 (-0.70 to 0.04)*	3	115	0.0							SMD			21.
p-cresyl sulfate (PCS) in serum and plasma	-0.31 (-0.68 to 0.06)*	3	115	0.0										22.
interleukin (IL)-6	-1.08 (-1.64 to -0.53)	3	95	35.0										23.
high sensitive C-reaction protein (hsCRP) in serum	0.17(-0.22 to 0.56)*	3	95	14.0										24.
albumin in serum	0.06 (-0.06 to 0.18)*	4	159	0.0							WMD			25.
phosphorus in serum	-0.03 (-0.36 to 0.30)*	5	179	0.0							WMD			26.
IBS severity	-0.66 (-0.88 to -0.44)	12	772	54.0	van LANEN A 2021[6]	low-FODMAP (fermentable oligo-, di- and monosaccharides, and polyols) diet	4 days to 3 months	Intervention and observational studies	adult human subjects with IBS diagnosed according to the Rome III or IV criteria	Vs. control diet	SMD	random	0.087	27.
IBS Severity Scoring System (IBS-SSS)	-45.13 (-76.56 to -13.69)	7	503	89.0							MD		NP	28.
Quality of life	4.93 (1.77 to 8.08)	6	483	42.0										29.
fasting blood sugar (FBS)	-10.28 (-16.53 to -4.02)	7	323	58.0	SALARI A 2021[7]	kefir beverage	15-84 days	clinical trials	Type II diabetic patients	Vs. control beverages	MD	random	NP	30.
HbA1c	-0.64 (-1.36 to 0.08)*	4	271	97.0										31.
insulin levels	-2.87 (-3.96 to -1.78)	3	169	87.0								fixed		32.
depression	0.76 (0.64 to 0.90)	5	97023	43.9	Fatahi S 2021[8]	dietary fiber	NP	cross-sectional studies.	adults and children	higher dietary fiber consumption vs.	OR	random	0.31	33.

									of all ages and genders	Lower dietary fiber consumption				
duration of diarrhea episodes	-20.31 (-27.06 to -13.57)	4	224	24.0	MALAGON-ROJAS J N 2020[9]	Postbiotics (bioactive compounds produced during a fermentation process (including microbial cells, cell constituents and metabolites) that supports health and/or wellbeing): non-viable L. acidophilus LB	4 days to 5 months	All RCTs	young children (preferably younger than 5 years of age) with acute diarrhea	Vs. placebo or no intervention	MD	random	NP	34.

* No statistical significance; CI, confidence interval; RCT, randomized controlled trial; RR, relative risk; HR, hazard ratio; MD, mean difference; SMD, standard mean difference; WMD, weighted mean difference; OR, odds ratio; NA, not available; NP, not published.

References

1. Jørgensen, A.R.; Egeberg, A.; Gideonsson, R.; Weinstock, L.B.; Thyssen, E.P.; Thyssen, J.P. Rosacea is associated with Helicobacter pylori: a systematic review and meta-analysis. *J. Eur. Acad. Dermatol.* **2017**, *31*, 2010-2015, doi: 10.1111/jdv.14352.
2. Zheng, Y.; Ding, Q.; Wei, Y.; Gou, X.; Tian, J.; Li, M.; Tong, X. Effect of traditional Chinese medicine on gut microbiota in adults with type 2 diabetes: A systematic review and meta-analysis. *Phytomedicine* **2021**, *88*, 153455, doi: 10.1016/j.phymed.2020.153455.
3. Milajerdi, A.; Ebrahimi-Daryani, N.; Dieleman, L.A.; Larijani, B.; Esmaillzadeh, A. Association of Dietary Fiber, Fruit, and Vegetable Consumption with Risk of Inflammatory Bowel Disease: A Systematic Review and Meta-Analysis. *Adv. Nutr.* **2021**, *12*, 735-743, doi: 10.1093/advances/nmaa145.
4. Zama, D.; Gori, D.; Muratore, E.; Leardini, D.; Rallo, F.; Turroni, S.; Prete, A.; Brigidi, P.; Pession, A.; Masetti, R. Enteral versus Parenteral Nutrition as Nutritional Support after Allogeneic Hematopoietic Stem Cell Transplantation: a Systematic Review and Meta-Analysis. *Transplantation and Cellular Therapy* **2021**, *27*, 180-181, doi: 10.1016/j.jtct.2020.11.006.
5. Jia, L.; Dong, X.; Li, X.; Jia, R.; Zhang, H. Benefits of resistant starch type 2 for patients with end-stage renal disease under maintenance hemodialysis: a systematic review and meta-analysis. *Int. J. Med. Sci.* **2021**, *18*, 811-820, doi: 10.7150/ijms.51484.
6. van Lanen, A.; de Bree, A.; Greyling, A. Efficacy of a low-FODMAP diet in adult irritable bowel syndrome: a systematic review and meta-analysis. *Eur. J. Nutr.* **2021**, *60*, 3505-3522, doi: 10.1007/s00394-020-02473-0.
7. Salari, A.; Ghodrat, S.; Gheflati, A.; Jarahi, L.; Hashemi, M.; Afshari, A. Effect of kefir beverage consumption on glycemic control: A systematic review and meta-analysis of randomized controlled clinical trials. *Complementary Therapies in Clinical Practice* **2021**, *44*, 101443, doi: 10.1016/j.ctcp.2021.101443.

8. Fatahi, S.; Matin, S.S.; Sohouli, M.H.; Găman, M.; Raee, P.; Olang, B.; Kathirgamathamby, V.; Santos, H.O.; Guimarães, N.S.; Shidfar, F. Association of dietary fiber and depression symptom: A systematic review and meta-analysis of observational studies. *Complement. Ther. Med.* **2021**, *56*, 102621, doi: 10.1016/j.ctim.2020.102621.
9. Malagón-Rojas, J.N.; Mantziari, A.; Salminen, S.; Szajewska, H. Postbiotics for Preventing and Treating Common Infectious Diseases in Children: A Systematic Review. *Nutrients* **2020**, *12*, 389, doi: 10.3390/nu12020389.
