# Supplementary material for: Gastrointestinal Microbiome and Multiple Health Outcomes: Umbrella Review
Source: Nutrients. 2022 Sep 9;14(18):3726. doi: 10.3390/nu14183726 (PMC9505003; doi:10.3390/nu14183726)
Supplement: Supplementary file 1 [file nutrients-14-03726-s001.zip › Supplementary Table S11.pdf]

Supplement Table S11. Association between disease and changes in gastrointestinal microbiota and its metabolites.

outcome(s) of interest (cancer outcomes and non-cancer outcomes)	estimated summary effect (95% CI)	number of studies/ total studies	number of envets or total participants	Heterogeneity (I^2, %)	the first author + year of publication	Intervention	Duration of Intervention/follow-up	study design (cohort, case-control, randomized controlled trial [RCTs]	populations	outcome comparison (e.g., highest versus lowest/none, any versus none or dose-response)	meta-analysis metric(OR, odds ratio; RR, relative risk; HR, hazard ratio; MD, mean difference)	type of effect model (fixed or random)	publication bias (Egger test P value or Funnel plot)
small intestinal bacterial overgrowth (SIBO)	36% (17% to 60%)	9	NP	91.0	CAPURSO G 2016[1]	-	NP	cross-sectional or case-control design	patients with a presumed diagnosis of Chronic pancreatitis (CP), age＞18 years	-	Event rate	random	0.6
	4.1 (1.6 to 10.4)	7	513	59.7						Vs. control	OR	random	0.2
prevalence of small intestinal bacterial overgrowth (SIBO)	7.15 (4.91 to 10.41)	21	NP	0.0	SHAH A 2017[2]	-	NP	case-control studies	patients with Chronic liver disease (CLD)	Vs. control	OR	random	NP
prevalence of small intestinal bacterial overgrowth (SIBO)	6.83 (4.16 to 11.21)	21	1570	0.0	MASLENNIKOV R 2018[3]	-	NP	clinical trials	age ≥ 18 years; patients with diagnosis of cirrhosis	Vs. control	OR	random	0.158
Prevalence of ascites	2.72 (1.65 to 4.50)	5	NP	0.0					age ≥ 18 years; patients with diagnosis of cirrhosis with SIBO	age ≥ 18 years; patients with diagnosis of cirrhosis with SIBO vs. No SIBO			NP
minimal hepatic encephalopathy	6.28 (2.10 to 18.80)	3	NP	61.6									
spontaneous bacterial peritonitis	3.28 (1.36 to 7.90)	7	NP	45.7									
Nonalcoholic fatty liver disease (NAFLD)	3.82 (1.93 to 7.59)	10	NP	65.0	WIJARNPREECHA K 2020 [4]	-	NP	cross-sectional studies, case-control studies and cohort studies	patients with small intestinal bacterial overgrowth (SIBO)	with SIBO vs. No SIBO	OR	random	No serious bias
Prevalence of a positive lactulose hydrogen breath test (LHBT) in IBS	1.3 (0.8 to 1.9)*	9	1104	75.0	GHOSHAL U C 2020[5]	-	NP	case series, case-control studies	consecutive adult (>18 years) subjects meeting diagnostic criteria for IBS (physicians'	With IBS vs. control	RR	random	No serious bias
Prevalence of a	4.23 (3.02 to 5.95)	9	2193	0.0									

positive glucose hydrogen breath test (GHBT) in IBS									diagnosis, using Manning2 or Rome criteria)				
Prevalence of SIBO on upper gut aspirate culture in IBS	3.01 (1.31 to 6.90)	5	508	0.0									
Prevalence of SIBO	1.40 (0.96 to 2.02)*	6	609	30.2					Patients with diarrhea-predominant IBS	Vs. other subtypes IBS			
Prevalence of SIBO	4.7 (3.1 to 7.2)	23	4407	74.0	CHEN B 2018[6]	-	NP	Case-control or case-series studies	subjects meeting diagnostic criteria for Irritable bowel syndrome (IBS), including a physician's diagnosis, questionnaire data, or specific symptom-based criteria such as the Manning or Rome criteria	vs. healthy controls	OR	Random	No serious bias
methane positive SIBO	1.1 (0.8 to 1.5)*	9	NP	3.3	GANDHI A 2021[7]	-	NP	Case-control or prevalence studies	patients with IBS (and their subtypes)	vs. healthy controls	OR	random	NP
methane positive SIBO	0.5 (0.2 to 1.3)*	6	1055	70.8					patients with IBD (and their subtypes)				
Prevalence of SIBO	12.51 (6.51 to 24.03)	5	475	0.0	FENG X 2021[8]	-	NP	cohort study or case-control study or cross-sectional study	age > 18 years, subjects meeting the diagnostic criteria for systemic sclerosis (SSc) (such as ACR 1980 or ACR-EULAR 2013)	vs. healthy controls	OR	fixed	NA
diarrhea	8.82 (4.09 to 19.00)	6	249	9.0						the SSc patients with SIBO vs. those without SIBO			
Prevalence of SIBO	2.08 (0.82 to 5.31)*	5	1187	84.0	WIJARNPREECHA K 2020[9]	-	NP	cohort studies or cross-sectional studies	participants with vs. without Obesity (overweight 23-27.5 kg/m2 and obesity above 27.5	participants with vs. without Obesity	OR	random	The funnel plot was asymmetric

									kg/m2)			
Prevalence of SIBO	5.22 (3.33 to 8.19)	4	559	0.0	LI X Q 2021[10]	-	NP	studies that had a cross-sectional, cohort or case-control design	subjects who met the Parkinson's disease (PD) diagnostic criteria	vs. healthy controls	OR	fixed	NP
prevalence of bloating in PD patients	1.67 (0.65 to 4.27)*	5	401	72.0						patients with vs. without SIBO			
disease duration	-0.25 (-3.64 to 3.14)*	3	186	91.0									
prevalence of constipation	0.38 (0.08 to 1.78)*	4	368	85.0									
diarrhoea	1.06 (0.58 to 1.91)*	4	368	0.0									
circulating trimethylamine N-oxide (TMAO) concentrations	67.9 (52.7 to 83.2)μmol/L	6	770	93.0	ZENG Y 2021[11]	-	NP	observational studies with cohort, cross-sectional, or case-control designs	patients with advanced CKD aged >18 years	versus healthy subjects	MD	random	NP
GFR	-12.9 (-16.6 to -9.14) mL/(min 1.73 m^2)	16	28260	98.0						subjects with high concentrations of TMAO compared with subjects with low concentrations of TMAO			0.390
stroke	1.68 (0.87 to 3.24)*	5	4910	84.9	FARHANGI M A 2020[12]	-	NP	observational (case-control, analytic cross-sectional, nested case-control, case-cohort or cohort)	Human	highest versus the lowest category of trimethylamine N-oxide (TMAO) concentrations	OR	random	0.197
the circulating levels of trimethylamine N-oxide (TMAO)	2.20 (1.21 to 3.18)μmol/L	4	2484	99.7						stroke versus non-stroke patients	WMD		0.170
The relationship between TMAO plasma levels and incidence of major adverse cardiac events (MACE)	1.87 (1.41 to 2.47)	7	NP	56.5	YAO M E 2020[13]	-	NP	Prospective, observational studies	Patient with acute coronary syndrome (ACS)	high concentrations of TMAO in comparison with low concentratio	HR	random	NP

										ns			
the association between baseline TMAO and cardiovascular events (CVEs) risk	1.23 (1.07 to 1.42)	5	8139	31.4	QI J Q 2018[14]	-	NP	prospective cohort study	human	baseline TMAO (highest versus lowest category)	hazard ratios (HR)	random	No serious bias
all-cause mortality	1.79 (1.23 to 2.60)	10	13195	94.0	SCHIATTARELLA G G 2017[15]	-	NP	-	non chronic kidney disease (CKD)	patients with high TMAO plasma levels vs. patients with low TMAO levels	hazard ratios (HR)	random	NP
	2.27 (1.13 to 4.58)	5	2467	91.0					chronic kidney disease (CKD)				
the incidence of major adverse cardio and cerebrovascular events (MACCE)	1.67 (1.33 to 2.11)	5	13944	46.0					-				
mortality risk	1.466 (1.291 to 1.665)	20	31230	81.9	FARHANGI M A 2020[16]	-	NP	prospective cohort, nested case–control, case cohort, case–control, or analytical cross-sectional studies	participants >18 y of age	highest versus lowest TMAO categories	hazard ratios (HR)	random	0.002
major adverse cardiovascular events (MACE)	1.62 (1.43 to 1.85)	19	19256	23.5	HEIANZA Y 2017[17]	-	1-6 years	observational studies	human	highest versus lowest TMAO categories	RR	random	> 0.1
Associations of elevated L-carnitine or choline concentrations and the risk of MACE	1.26 (1.10 to 1.44)	6	NP	0.0									
the association of elevated betaine concentrations with the risk of MACE	1.43 (1.19 to 1.73)	6	NP	6.4									
absolute risk of MACE	2.05 (1.61 to 2.61)	3	3089	50.0	GUASTI L 2021[18]	-	1 month to 3 years	cohort	adult patients defined at high/very high cardiovascular risk	patients with a high baseline TMAO value vs. patients with a low	RR	random	NP
The absolute risk of	3.42 (2.27 to 5.15)	3	1801	0.0			6 months					fixed	

all-cause mortality							to 7 years			baseline TMAO value			
systolic blood pressure	2.16 (1.11 to 3.21) mmHg	9	15097	52.2	ABBASALIZAD F M 2021[19]	-	NP	Observational studies	participants were 18 years of age or older	the highest vs the lowest categories of TMAO concentrations	WMD	random	0.006
diastolic blood pressure	0.44 (-0.93 to 1.81)*	7	8811	84.1									No serious bias
high-density lipoprotein cholesterol	-0.55 (-1.78 to 0.69)*	13	NP	94.0									
low-density lipoprotein cholesterol	-0.52 (-2.34 to 1.29)*	13	NP	68.5									
triglycerides	1.16 (-0.45 to 2.77)*	12	NP	57.6									
total cholesterol	0.44 (-3.05 to 3.92)*	9	NP	84.3									
hypertension prevalence	1.12 (1.06 to 1.17)	8	11750	64.0	GE X 2019[20]	-	NP	Studies recording or analyzing the proportion of hypertensive patients in a certain population and their circulating TMAO concentrations		individuals with high TMAO concentrations (above the median TMAO concentration) vs. those with low TMAO concentrations (below the median TMAO concentration).	RR	random	NP
The prevalence of diabetes	1.89 (1.63 to 2.19)	9	12961	50.2	ZHUANG R 2019[21]	-	NP	cohort studies, case-control studies, cross-sectional studies	-	the high-level TMAO group vs. the low-level TMAO group	OR	random	0.275
TMAO levels	0.36 (0.30 to 0.42)	5	5330	0.0						DM vs. non-DM populations	SMD	fixed	0.205
association between TMAO level and	1.68 (1.44 to 1.96)	7	6879	0.0	LI W 2020[22]	-	NP	prospective	adults with heart failure(18 or more	the highest vs. lowest	HR	rando	No serious

MACEs in patients with heart failure								studies	years old)	TMAO tertiles		m	bias
association between TMAO level and all-cause mortality in patients with heart failure	1.67 (1.17 to 2.38)	5	NP	64.0									
BMI	0.56 (0.03 to 1.10)*	12	NP	96.8	DEHGHAN P 2020[23]	-	NP	observational studies with cohort, case-control, nested case-control, or analytic cross-sectional studies	-	highest versus lowest categories of TMAO	WMD	random	No serious bias
CRP concentrations	0.142 (0.094 to 0.190)	9	NP	84.1	FARHANGI M A 2020[24]	-	NP	observational studies with the design of prospective cohort, nested case-control, case-cohort, case-control or analytic cross-sectional studies	excluded the studies that were performed among children, pregnant or lactating women,	highest versus lowest TMAO categories	SMD	random	0.696
rate of H. pylori infection	0.64 (0.54 to 0.75)	24	NP	75.8	LUTHER J 2010[25]	-	NP	-	human	H. pylori in IBD patients vs. controls	RR	random	The funnel plot was asymmetric
the risk of Nonalcoholic fatty liver disease (NAFLD)	1.529 (1.336 to 1.750)	21	NP	95.6	LIU R Q 2019[26]	-	NP	-	human	patients with H pylori infection and those without	OR	random	0.370
Anti-H. pylori IgG	42.45 (9.66 to 186.56)	3	105	0.0	DARDIOTIS E 2020[27]	-	NP	case-control studies	human	Guillain-Barré Syndrome (GBS) patients vs. control	OR	fixed	No serious bias
serum antibody	2.31 (1.30 to 4.11)	4	325	57.0									
likelihood of H.	1.47 (0.90 to 2.40)*	9	NP	87.4	NG Q X	-	NP	case-control or	patients with IBS	patients with IBS vs.	OR	Random	0.189

pylori infection					2019[28]			cross-sectional study		Without IBS		m	
the bacterial count of F. prausnitzii	-0.94 (-1.07 to -0.80)	12	1278	96.0	CAO Y 2014[29]	-	NP	-	patients with IBD	IBD patients vs. healthy controls	SMD	fixed	No serious bias
Clostridium coccoides	-0.49 (-0.79 to -0.19)	4	NP	0.0	Prosberg M 2016[30]	-	NP	prospective and retrospective Studies (Cohort, case-control studies)	adults with IBD diagnosed with CD or UC based on endoscopy and histology	patients with active IBD compared to IBD in remission	MD	random	No serious bias
Clostridium leptum	-0.44 (-0.74 to -0.14)	4		0.0									
Escherichia coli	0.43 (-0.04 to 0.91)*	3		26.0									
Faecalibacterium prausnitzii	-0.81 (-1.23 to -0.39)	6		62.0									
Bifidobacterium	-0.37 (-0.56 to -0.17)	7		0.0									
Lactobacillus	-0.07 (-0.36 to 0.22)*	4		0.0									
acetate	-0.51 (-0.90 to -0.13)	9	596	73.0	ZHUANG X 2019[31]	-	NP	-	IBD patients with or without medication	patients with UC. vs. healthy controls	SMD	random	No serious bias
propionate	-0.31 (-0.65 to 0.02)*	8	581	65.0									
butyrate	-0.27 (-0.76 to 0.22)*	9	602	84.0									
valerate	-0.65 (-1.02 to -0.28)	5	240	41.0									
total SCFAs	-0.51 (-0.95 to -0.07)	6	454	74.0									
acetate	-1.43 (-2.81 to -0.04)	5	216	94.0						CD patients vs. healthy controls			
propionate	-0.23 (-0.79 to 0.32)*	5	216	68.0									
butyrate	-0.77 (-1.39 to -0.14)	4	282	67.0									
valerate	-0.75 (-1.47 to -0.02)	4	194	77.0									
total SCFAs	-1.37 (-3.75 to 1.02)*	3	168	97.0									
circulating levels of zonulin	0.97 (0.10 to 1.85)	4	198	86.6	SAFADI J M 2021[32]	-	NP	Case-control studies	-	patients with CFS and MDD vs. controls	SMD	random	NP
levels of antibodies against bacterial endotoxins	0.99 (0.27 to 1.70)	7	1707	97.1						patient with BPD, CFS, major depressive disorder (MDD), schizophreni			

									a (SCZ) vs. controls			
circulating levels of sCD14	0.54 (0.16 to 0.81)	2	2196	90.7					patients with bipolar disorder (BPD), chronic fatigue syndrome (CFS), MDD, SCZ vs. controls			
levels of circulating alpha-1-antitrypsin (A-1-AT)	1.23 (0.57 to 1.88)	6	551	89.3					patients with BPD, MDD, SCZ vs. controls			
levels of circulating intestinal fatty-acid binding protein (I-FABP)	0.27 (-0.07 to 0.60)*	4	193	23.0					patients with CFS and MDD vs. controls			
fecal *Lactobacillus*	-0.57 (-0.99 to -0.16) log_{10}CFU/g	17	1097	98.0	WANG L - 2020[33]	NP	Case-control study	Patients with IBS	fecal bacterial counts in IBS vs healthy controls	MD	random	No serious bias
fecal *Bifidobacterium*	-1.04 (-1.58 to -0.49) log_{10}CFU/g	17	1097	98.0								0.015
fecal *Bacteroides*	0.13 (-0.06 to 0.32) log_{10}CFU/g*	11	654	74.0								No serious bias
Enterococcus	0.12 (-0.46 to 0.70) log_{10}CFU/g*	13	839	97.0								
Escherichia coli	0.60 (0.17 to 1.03) log_{10}CFU/g	10	632	93.0								
Enterobacter	0.74 (0.01 to 1.47) log_{10}CFU/g	7	435	96.0								
acetate in the blood and feces	0.87 (0.24 to 1.50)	8	NP	88.5	KIM K N - 2019[34]	NP	Case-control studies that were conducted in humans	obese patient	obese individuals vs. nonobese individuals	SMD	Random	0.621
Propionate in feces	0.86 (0.35 to 1.36)	7		82.3								0.580
Valerate in feces	0.32 (0.00 to 0.64)	3		0.0								0.495
Butyrate	0.78 (0.29 to 1.27)	7		81.7								0.587
total Short chain fatty acids (SCFAs) in the feces	0.54 (-0.34 to 1.41)*	8		94.3								0.580

iso-butyrate in the feces	0.01 (-0.28 to 0.29)*	4		18.5								0.380
iso-valerate in the feces	-0.20 (-0.46 to 0.06)*	5		0.0								0.783
Bacteroidetes	-0.36 (-0.73 to 0.01)*	4		72.1								0.833
Firmicutes	-0.10 (-0.31 to 0.10)*	4		58.7								0.636
the alpha diversity (Simpson index)	-0.14 (-0.26 to -0.02)	12	1288	37.0	ZHOU J - 2020[35]	NP	cross-sectional studies	human	HIV+ individuals vs. HIV- individuals	SMD	Fixed	NP
the alpha diversity (Simpson index)	-0.27 (-0.43 to -0.11)	6	632	46.0					Men who have sex with men (MSM) vs. Non-MSM			
Shannon Index	0.15 (-0.12 to 0.42)*	4	NP	NP	SANADA K - 2020[36]	NP	observational studies	Major depressive disorder (MDD) patient	Major depressive disorder (MDD) patient vs. controls.	SMD	random	NP
Simpson index	0.29 (-0.11 to 0.69)*	4										
observed species	-0.26 (-0.47 to -0.06)	20	1686	75.0	NIKOLOVA V L 2021[37]	NP	observational case-control design	general adult population (age 18-65 years) with a psychiatric diagnosis of interest	Patients With Psychiatric Disorders Compared With Healthy Controls	SMD	random	NP
	-0.16 (-0.58 to 0.27)*	6	NP	83.0					Patients With major depressive disorder vs.Healthy Controls			
	-0.61 (-1.19 to -0.03)	3		80.0					Patients With bipolar disorder vs.Healthy Controls			
	-0.04 (-0.31 to 0.24)*	4		35.0					Patients With psychosis			

					and schizophrenia vs.Healthy Controls
Chao 1	-0.50 (-0.79 to -0.21)	26	1917	88.0	Patients With Psychiatric Disorders Compared With Healthy Controls
	-0.34 (-1.08 to 0.40)*	6	NP	91.0	Patients With Psychiatric Disorders Compared With Healthy Controls
	-0.53 (-1.01 to -0.05)	4	NP	62.0	Patients With bipolar disorder vs.Healthy Controls
	-0.58 (-1.29 to 0.12)*	7	NP	95.0	Patients With psychosis and schizophrenia vs.Healthy Controls
	-0.86 (-1.52 to -0.21)	4	NP	80.0	Patients With anorexia nervosa vs.Healthy Controls
Shannon index	-0.12 (-0.27 to 0.03)*	29	2348	67.0	Patients With Psychiatric Disorders Compared With Healthy Controls

	-0.28 (-0.62 to 0.06)	11	NP	80.0	Patients With major depressive disorder vs.Healthy Controls
	0.09 (-0.43 to 0.62)*	4	NP	73.0	Patients With bipolar disorder vs.Healthy Controls
	-0.02 (-0.20 to 0.17)*	8	NP	47.0	Patients With psychosis and schizophrenia vs.Healthy Controls
Simpson index	0.04 (-0.13 to 0.21)*	11	795	30.0	Patients With Psychiatric Disorders Compared With Healthy Controls
	0.14 (-0.14 to 0.43)*	5	NP	42.0	Patients With major depressive disorder vs.Healthy Controls
	0.03 (-0.34 to 0.28）*	3	NP	6.0	Patients With bipolar disorder vs.Healthy Controls
phylogenetic diversity	-0.24 (-0.47 to 0.00)*	10	866	64.0	Patients With Psychiatric Disorders Compared With Healthy Controls

	-0.42 (-0.96 to 0.13)*	4	NP	83.0						Patients With major depressive disorder vs.Healthy Controls			
	-0.01 (-0.22 to 0.20)*	3	NP	0.0						Patients With psychosis and schizophrenia vs.Healthy Controls			
F. nucleatum prevalence	10.06 (4.48 to 22.58)	6	NP	0.0	GETHINGS-BEHNCKE C 2020[38]	-	NP	-	ages over 16 individuals with colorectal cancer vs. individuals with colorectal polyp or healthy controls	colorectal tumor tissue compared with healthy tissue from controls	OR	random	NP
F. nucleatum DNA being detected	1.83 (1.07 to 3.16)	5		66.9						colorectal tumor tissue compared with polyp tissue			
	2.51 (1.20 to 5.27)	3		0.0						in colorectal polyp tissue compared with healthy tissue from controls			
	2.51 (1.20 to 5.27)	7		50.0						colorectal cancer tissue compared with adjacent, normal tissue			
	9.01 (3.39 to 23.95)	7		72.6						fecal samples from patients with colorectal cancer compared with healthy controls			

Poor overall survival among patients with colorectal cancer	1.87 (1.12 to 3.11)	5		60.6						F. nucleatum positivity in tumor tissue	HR		
disease-free survival among patients with colorectal cancer	1.48 (0.84 to 2.59)*	3		88.5									
Lactobacillus	-2.72 (-5.94 to 0.50)*	4	NP	98.3	Liu H 2016[39]	-	NP	-	microbiota studies in colorectal cancer (CRC) versus healthy controls		SMD	random	No serious bias
Bifidobacterium	-3.30 (-6.57 to -0.03)	4		98.2									
Bacteroides-Prevotella	-0.71 (-2.56 to 1.14)*	3		96.3									
Faecalibacterium	-0.33 (-0.60 to -0.05)	3		0.0									
abundance of Escherichia	1.55 (0.57 to 2.54)	6	294	92.0	LI F 2021[40]	-	NP	Any study that provides necessary data on detecting gut microbiota for NAFLD patients and comparable controls	Patients with Nonalcoholic fatty liver disease (NAFLD) diagnosed with sonographic or histologic evidence	Vs. Healthy control	SMD	random	No serious bias
abundance of Prevotella	1.89 (0.02 to 3.76)	8	502	98.0									
abundance of Streptococcus	1.33 (0.62 to 2.05)	6	418	92.0									
genera Coprococcus	-1.75 (-3.13 to -0.37),	4	197	95.0									
Faecalibacterium	-13.23 (-17.59 to -8.87)	7	557	99.0									
Ruminococcus	-1.84 (-2.41 to -1.27)	6	362	82.0									
Bacteroides	-2.12 (-4.49 to 0.25)*	7	497	98.0									
Bifidobacterium	-0.68 (-1.89 to 0.53)*	4	185	95.0									
Blautia	0.17 (-1.38 to 1.73)*	8	504	98.0									
Clostridium	0.42 (-0.80 to 1.64)*	5	352	94.0									
Dorea	1.45 (-0.20 to 3.10)*	4	241	98.0									
Lactobacillus	-0.05 (-0.82 to 0.72)*	5	274	88.0									
Parabacteroides	0.49 (-0.26 to 1.24)*	6	357	82.0									
Roseburia	-0.90 (-2.70 to 0.91)*	5	224	96.0									
concentration of acetate	0.05 (-0.16 to 0.27)*	8	NP	44.0	SUN Q 2018[41]	-	NP	the study design for comparing patients with IBS versus	Irritable bowel syndrome (IBS), age<12 years was excluded	Vs. Healthy control	SMD	fixed	0.17
proportion of acetate	-0.27 (-0.59 to 0.05)*	4		57.0								fixed	NP

concentration of propionate	-0.04 (-0.52 to 0.44)*	8		76.0				HCs was that of case-control, and that for comparing pretreatment with posttreatment was randomized controlled trial (RCT) or self-controlled study				random	0.24
proportion of propionate	0.44 (0.12 to 0.76)	4		0.0								fixed	NP
concentration of butyrate	0.12 (-0.11 to 0.35)*	7		50.0								fixed	0.64
proportion of butyrate	0.05 (-0.68 to 0.78)*	4		80.0								random	NP
concentration of iso-butyrate	-0.15 (-0.91 to 0.60)*	4		81.0								random	
proportion of iso-butyrate	0.25 (-0.07 to 0.56)	4		0.0								fixed	
concentration of valerate	-0.03 (-0.35 to 0.28)*	4		51.0								fixed	
proportion of valerate	-0.19 (-1.07 to 0.69)*	4		86.0								random	
concentration of iso-valerate	-0.38 (-1.18 to 0.42)*	4		83.0								random	
proportion of iso-valerate.	-0.43 (-1.54 to 0.69)*	4		91.0								random	
Streptococcus	-0.999 (-1.547 to -0.449)	5	642/998	21.57	ANDREO2021 [42]	-	-	controlled trial	Children with Autism	with versus without Autism	SMD+	random	NP
Bifdobacterium	-0.513 (-0.953 to -0.073)	7		62.27									0.575
Prevotellaceae	-0.37 (-0.62 to -0.11)	9	592/1038	72.0	SHEN T 2021 [43]	-	-	NP	patients with Parkinson's disease (PD)	patients with PD versus healthy controls	SMD	Fixed	No serious bias
Faecalibacterium	-0.41 (-0.57 to -0.24)	5	422/663	52.0									
Lachnospiraceae	-0.34 (-0.59 to -0.09)	7	592/997	67.0									
Bifidobacteriaceae	0.38 (0.12 to 0.63)	7	630/1080	72.0									
Ruminococcaceae	0.58 (0.07 to 1.10)	9	499/871	91.0									
Verrucomicrobiaceae	0.45 (0.21 to 0.69)	7	624/1061	68.0									
Christensenellaceae	0.20 (0.07 to 0.34)	7	532/884	0.0									
Helicobacter pylori	1.68 (1.00 to 2.84)	14	928/2500	NP	JORGENSENA 2017[44]	-	-	Case-control, cross-sectional, cohort, or nested case-control	rosacea patients	rosacea patients with control groups.	OR	random	No serious bias

	design	

* No statistical significance; CI, confidence interval; RCT, randomized controlled trial; RR, relative risk; HR, hazard ratio; MD, mean difference; SMD, standard mean difference; WMD, weighted mean difference; OR, odds ratio; NA, not available; NP, not published.

References

1. Capurso, G.; Signoretti, M.; Archibugi, L.; Stigliano, S.; Delle Fave, G. Systematic review and meta-analysis: Small intestinal bacterial overgrowth in chronic pancreatitis. *United European Gastroenterology Journal* **2016**, *4*, 697-705, doi: 10.1177/2050640616630117.
2. Shah, A.; Shanahan, E.; Macdonald, G.; Fletcher, L.; Ghasemi, P.; Morrison, M.; Jones, M.; Holtmann, G. Systematic Review and Meta-Analysis: Prevalence of Small Intestinal Bacterial Overgrowth in Chronic Liver Disease. *Semin. Liver Dis.* **2017**, *37*, 388-400, doi: 10.1055/s-0037-1608832.
3. Maslennikov, R.; Pavlov, C.; Ivashkin, V. Small intestinal bacterial overgrowth in cirrhosis: systematic review and meta-analysis. *Hepatol. Int.* **2018**, *12*, 567-576, doi: 10.1007/s12072-018-9898-2.
4. Wijarnpreecha, K.; Lou, S.; Watthanasuntorn, K.; Kroner, P.T.; Cheungpasitporn, W.; Lukens, F.J.; Pungpapong, S.; Keaveny, A.P.; Ungprasert, P. Small intestinal bacterial overgrowth and nonalcoholic fatty liver disease: a systematic review and meta-analysis. *Eur. J. Gastroen. Hepat.* **2020**, *32*, 601-608, doi: 10.1097/MEG.0000000000001541.
5. Ghoshal, U.C.; Nehra, A.; Mathur, A.; Rai, S. A meta-analysis on small intestinal bacterial overgrowth in patients with different subtypes of irritable bowel syndrome. *J. Gastroen. Hepatol.* **2020**, *35*, 922-931, doi: 10.1111/jgh.14938.
6. Chen, B.; Kim, J.J.; Zhang, Y.; Du, L.; Dai, N. Prevalence and predictors of small intestinal bacterial overgrowth in irritable bowel syndrome: a systematic review and meta-analysis. *J. Gastroenterol.* **2018**, *53*, 807-818, doi: 10.1007/s00535-018-1476-9.
7. Gandhi, A.; Shah, A.; Jones, M.P.; Koloski, N.; Talley, N.J.; Morrison, M.; Holtmann, G. Methane positive small intestinal bacterial overgrowth in inflammatory bowel disease and irritable bowel syndrome: A systematic review and meta-analysis. *Gut Microbes* **2021**, *13*, 1933313, doi: 10.1080/19490976.2021.1933313.
8. Feng, X.; Li, X.; Jiang, Z. Prevalence and predictors of small intestinal bacterial overgrowth in systemic sclerosis: a systematic review and meta-analysis. *Clin. Rheumatol.* **2021**, *40*, 3039-3051, doi: 10.1007/s10067-020-05549-8.
9. Wijarnpreecha, K.; Werlang, M.E.; Watthanasuntorn, K.; Panjawatanan, P.; Cheungpasitporn, W.; Gomez, V.; Lukens, F.J.; Ungprasert, P. Obesity and Risk of Small Intestine Bacterial Overgrowth: A Systematic Review and Meta-Analysis. *Digest. Dis. Sci.* **2020**, *65*, 1414-1422, doi: 10.1007/s10620-019-05887-x.
10. Li, X.; Feng, X.; Jiang, Z.; Jiang, Z. Association of small intestinal bacterial overgrowth with Parkinson's disease: a systematic review and meta-analysis. *Gut Pathog.* **2021**, *13*, doi: 10.1186/s13099-021-00420-w.
11. Zeng, Y.; Guo, M.; Fang, X.; Teng, F.; Tan, X.; Li, X.; Wang, M.; Long, Y.; Xu, Y. Gut Microbiota-Derived Trimethylamine N-Oxide and Kidney Function: A Systematic Review and Meta-Analysis. *Adv. Nutr.* **2021**, *12*, 1286-1304, doi: 10.1093/advances/nmab010.

12. Farhangi, M.A.; Vajdi, M.; Asghari-Jafarabadi, M. Gut microbiota-associated metabolite trimethylamine N-Oxide and the risk of stroke: a systematic review and dose–response meta-analysis. *Nutr. J.* **2020**, *19*, 76, doi: 10.1186/s12937-020-00592-2.
13. Yao, M.; Liao, P.; Zhao, X.; Wang, L. Trimethylamine-N-oxide has prognostic value in coronary heart disease: a meta-analysis and dose-response analysis. *BMC Cardiovasc. Disor.* **2020**, *20*, doi: 10.1186/s12872-019-01310-5.
14. Qi, J.; You, T.; Li, J.; Pan, T.; Xiang, L.; Han, Y.; Zhu, L. Circulating trimethylamine N-oxide and the risk of cardiovascular diseases: a systematic review and meta-analysis of 11 prospective cohort studies. *J. Cell. Mol. Med.* **2018**, *22*, 185-194, doi: 10.1111/jcmm.13307.
15. Schiattarela, G.G.; Sannino, A.; Toscano, E.; Giugliano, G.; Gargiulo, G.; Franzone, A.; Avvedimento, M.; Trimarco, B.; Esposito, G.; Perrino, C. P1482Gut microbe-generated metabolite trimethylamine-N-oxide and cardiovascular risk: a systematic review and meta-analysis of mortality outcome. *Eur. Heart J.* **2017**, *38*, 304, doi: 10.1093/eurheartj/ehx502.P1482.
16. Farhangi, M.A. Gut microbiota-dependent trimethylamine N-oxide and all-cause mortality: Findings from an updated systematic review and meta-analysis. *Nutrition* **2020**, *78*, 110856, doi: 10.1016/j.nut.2020.110856.
17. Heianza, Y.; Ma, W.; Manson, J.E.; Rexrode, K.M.; Qi, L. Gut Microbiota Metabolites and Risk of Major Adverse Cardiovascular Disease Events and Death: A Systematic Review and Meta-Analysis of Prospective Studies. *J. Am. Heart Assoc.* **2017**, *6*, e4947, doi: 10.1161/JAHA.116.004947.
18. Guasti, L.; Galliazzo, S.; Molaro, M.; Visconti, E.; Pennella, B.; Gaudio, G.V.; Lupi, A.; Grandi, A.M.; Squizzato, A. TMAO as a biomarker of cardiovascular events: a systematic review and meta-analysis. *Intern. Emerg. Med.* **2021**, *16*, 201-207, doi: 10.1007/s11739-020-02470-5.
19. Abbasalizad Farhangi, M.; Vajdi, M. Gut microbiota–associated trimethylamineN -oxide and increased cardiometabolic risk in adults: a systematic review and dose-response meta-analysis. *Nutr. Rev.* **2021**, *79*, 1022-1042, doi: 10.1093/nutrit/nuaa111.
20. Ge, X.; Zheng, L.; Zhuang, R.; Yu, P.; Xu, Z.; Liu, G.; Xi, X.; Zhou, X.; Fan, H. The Gut Microbial Metabolite Trimethylamine N-Oxide and Hypertension Risk: A Systematic Review and Dose–Response Meta-analysis. *Adv. Nutr.* **2019**, *11*, 66-76, doi: 10.1093/advances/nmz064.
21. Zhuang, R.; Ge, X.; Han, L.; Yu, P.; Gong, X.; Meng, Q.; Zhang, Y.; Fan, H.; Zheng, L.; Liu, Z.; et al. Gut microbe–generated metabolite trimethylamineN -oxide and the risk of diabetes: A systematic review and dose-response meta-analysis. *Obes. Rev.* **2019**, *20*, 883-894, doi: 10.1111/obr.12843.
22. Li, W.; Huang, A.; Zhu, H.; Liu, X.; Huang, X.; Huang, Y.; Cai, X.; Lu, J.; Huang, Y. Gut microbiota-derived trimethylamineN -oxide is associated with poor prognosis in patients with heart failure. *Med. J. Australia* **2020**, *213*, 374-379, doi: 10.5694/mja2.50781.
23. Dehghan, P.; Farhangi, M.A.; Nikniaz, L.; Nikniaz, Z.; Asghari Jafarabadi, M. Gut microbiota-derived metabolite trimethylamine N-oxide (TMAO) potentially increases the risk of obesity in adults: An exploratory systematic review and dose-response meta- analysis. *Obes. Rev.* **2020**, *21*, e12993, doi: 10.1111/obr.12993.
24. Farhangi, M.A.; Vajdi, M. Novel findings of the association between gut microbiota–derived metabolite trimethylamineN- oxide and inflammation: results from a systematic review and dose-response meta-analysis. *Crit. Rev. Food Sci.* **2020**, *60*, 2801-2823, doi: 10.1080/10408398.2020.1770199.
25. Luther, J.; Dave, M.; Higgins, P.D.R.; Kao, J.Y. Association between Helicobacter pylori infection and inflammatory bowel disease. *Inflamm. Bowel Dis.* **2010**, *16*, 1077-1084, doi: 10.1002/ibd.21116.
26. Liu, R.; Liu, Q.; He, Y.; Shi, W.; Xu, Q.; Yuan, Q.; Lin, Q.; Li, B.; Ye, L.; Min, Y.; et al. Association between Helicobacter pylori infection and nonalcoholic fatty liver. *Medicine* **2019**, *98*, e17781, doi: 10.1097/MD.0000000000017781.
27. Dardiotis, E.; Sokratous, M.; Tsouris, Z.; Siokas, V.; Mentis, A.F.A.; Aloizou, A.M.; Michalopoulou, A.; Bogdanos, D.P.; Xiromerisiou, G.; Deretzi, G.; et al. Association betweenHelicobacter py lori infection and Guillain-Barré Syndrome: A meta-analysis. *Eur. J. Clin. Invest.* **2020**, *50*, doi: 10.1111/eci.13218.

28. Ng, Q.X.; Foo, N.X.; Loke, W.; Koh, Y.Q.; Seah, V.J.M.; Soh, A.Y.S.; Yeo, W.S. Is there an association betweenHelicobacter pylori infection and irritable bowel syndrome? A meta-analysis. *World J. Gastroentero.* **2019**, *25*, 5702-5710, doi: 10.3748/wjg.v25.i37.5702.
29. Cao, Y.; Shen, J.; Ran, Z.H. Association betweenFaecalibacterium prausnitzii Reduction and Inflammatory Bowel Disease: A Meta-Analysis and Systematic Review of the Literature. *Gastroent. Res. Pract.* **2014**, *2014*, 1-7, doi: 10.1155/2014/872725.
30. Prosberg, M.; Bendtsen, F.; Vind, I.; Petersen, A.M.; Gluud, L.L. The association between the gut microbiota and the inflammatory bowel disease activity: a systematic review and meta-analysis. *Scand. J. Gastroentero.* **2016**, *51*, 1407-1415, doi: 10.1080/00365521.2016.1216587.
31. Zhuang, X.; Li, T.; Li, M.; Huang, S.; Qiu, Y.; Feng, R.; Zhang, S.; Chen, M.; Xiong, L.; Zeng, Z. Systematic Review and Meta-analysis: Short-Chain Fatty Acid Characterization in Patients With Inflammatory Bowel Disease. *Inflamm. Bowel Dis.* **2019**, *25*, 1751-1763, doi: 10.1093/ibd/izz188.
32. Safadi, J.M.; Quinton, A.M.G.; Lennox, B.R.; Burnet, P.W.J.; Minichino, A. Gut dysbiosis in severe mental illness and chronic fatigue: a novel trans-diagnostic construct? A systematic review and meta-analysis. *Mol. Psychiatr.* **2022**, *27*, 141-153, doi: 10.1038/s41380-021-01032-1.
33. Wang, L.; Alammar, N.; Singh, R.; Nanavati, J.; Song, Y.; Chaudhary, R.; Mullin, G.E. Gut Microbial Dysbiosis in the Irritable Bowel Syndrome: A Systematic Review and Meta-Analysis of Case-Control Studies. *J. Acad. Nutr. Diet.* **2020**, *120*, 565-586, doi: 10.1016/j.jand.2019.05.015.
34. Kim, K.N.; Yao, Y.; Ju, S.Y. Short Chain Fatty Acids and Fecal Microbiota Abundance in Humans with Obesity: A Systematic Review and Meta-Analysis. *Nutrients* **2019**, *11*, 2512, doi: 10.3390/nu11102512.
35. Zhou, J.; Zhang, Y.; Cui, P.; Luo, L.; Chen, H.; Liang, B.; Jiang, J.; Ning, C.; Tian, L.; Zhong, X.; et al. Gut Microbiome Changes Associated With HIV Infection and Sexual Orientation. *Front. Cell. Infect. Mi.* **2020**, *10*, 434, doi: 10.3389/fcimb.2020.00434.
36. Sanada, K.; Nakajima, S.; Kurokawa, S.; Barceló-Soler, A.; Ikuse, D.; Hirata, A.; Yoshizawa, A.; Tomizawa, Y.; Salas-Valero, M.; Noda, Y.; et al. Gut microbiota and major depressive disorder: A systematic review and meta-analysis. *J. Affect. Disorders* **2020**, *266*, 1-13, doi: 10.1016/j.jad.2020.01.102.
37. Nikolova, V.L.; Hall, M.R.B.; Hall, L.J.; Cleare, A.J.; Stone, J.M.; Young, A.H. Perturbations in Gut Microbiota Composition in Psychiatric Disorders. *JAMA Psychiat.* **2021**, *78*, 1343, doi: 10.1001/jamapsychiatry.2021.2573.
38. Gethings-Behncke, C.; Coleman, H.G.; Jordao, H.W.T.; Longley, D.B.; Crawford, N.; Murray, L.J.; Kunzmann, A.T. Fusobacterium nucleatum in the Colorectum and Its Association with Cancer Risk and Survival: A Systematic Review and Meta-analysis. *Cancer Epidemiology, Biomarkers & Prevention* **2020**, *29*, 539-548, doi: 10.1158/1055-9965.EPI-18-1295.
39. Liu, H.; Wu, H.; Bilegsaikhan, E.; Liu, T.; Lu, E.X.; Shen, X. Differential expression of intestinal microbiota in colorectal cancer compared with healthy controls: A systematic review and meta-analysis. *Int. J. Clin. Exp. Med.* **2016**, *9*, 10923-10930.
40. Li, F.; Ye, J.; Shao, C.; Zhong, B. Compositional alterations of gut microbiota in nonalcoholic fatty liver disease patients: a systematic review and Meta-analysis. *Lipids Health Dis.* **2021**, *20*, 22, doi: 10.1186/s12944-021-01440-w.
41. Sun, Q.; Jia, Q.; Song, L.; Duan, L. Alterations in fecal short-chain fatty acids in patients with irritable bowel syndrome. *Medicine* **2019**, *98*, e14513, doi: 10.1097/MD.0000000000014513.
42. Andreo-Martínez, P.; Rubio-Aparicio, M.; Sánchez-Meca, J.; Veas, A.; Martínez-González, A.E. A Meta-analysis of Gut Microbiota in Children with Autism. *Journal of Autism and Developmental Disorders* **2022**, *52*, 1374-1387, doi: 10.1007/s10803-021-05002-y.

43. Shen, T.; Yue, Y.; He, T.; Huang, C.; Qu, B.; Lv, W.; Lai, H. The Association Between the Gut Microbiota and Parkinson's Disease, a Meta-Analysis. *Front. Aging Neurosci.* **2021**, *13*, 636545, doi: 10.3389/fnagi.2021.636545.
44. Jørgensen, A.R.; Egeberg, A.; Gideonsson, R.; Weinstock, L.B.; Thyssen, E.P.; Thyssen, J.P. Rosacea is associated with Helicobacter pylori: a systematic review and meta-analysis. *J. Eur. Acad. Dermatol.* **2017**, *31*, 2010-2015, doi: 10.1111/jdv.14352.
