# Supplementary material for: Gastrointestinal Microbiome and Multiple Health Outcomes: Umbrella Review
Source: Nutrients. 2022 Sep 9;14(18):3726. doi: 10.3390/nu14183726 (PMC9505003; doi:10.3390/nu14183726)
Supplement: Supplementary file 1 [file nutrients-14-03726-s001.zip › Supplementary Table S2.pdf]

Supplement Table S2. Prebiotics/probiotics/synbiotics and mortality.

outcome(s) of interest	estimated summary effect (95% CI)	number of studies / total studies	number of intervention group or total participants	Heterogeneity (I^2, %)	the first author + year of publication	Intervention	Duration of Intervention/follow-up	study design	populations	outcome comparison	meta-analysis metric	type of effect model	publication bias
All-cause mortality	0.58(0.23 to 1.44)*	7	404	0.0	DALAL R 2017 [1]	probiotics	follow-up: 2 weeks to 3 months)	randomised clinical trials	people with any grade of acute or chronic hepatic encephalopathy	probiotics in any dosage vs. placebo or no intervention, or with any other treatment in people with hepatic encephalopathy.	RR	random	NP
Effects of Probiotics on Mortality	0.77 (0.65 to 0.92)	27	4117/8156	16.0	DERMYSHI E 2017 [2]	enteral administration of probiotics	NP	All RCTs	very low birth weight (VLBW)(<1,500 g) preterm (<34 weeks gestational age) infants with enteral administration of probiotics initiated within 10 days	enteral administration of probiotics vs. Control group	RR	fixed	0.012
Mortality (all-cause before hospital discharge)	0.76 (0.65 to 0.89)	51	10170	0.0	SHARIF S 2020 [3]	any probiotic or probiotic combination for at least one week	at least one week	RCTs and quasi-RCTs	very preterm (< 32 weeks' gestation) or very low birth weight (< 1500 g) infants	any probiotic or probiotic combination vs. placebo or no treatment.	RR	fixed	0.11
Mortality (all-cause before hospital discharge)	0.91 (0.71 to 1.16)*	6	1661	0.0					extremely preterm (< 28 weeks' gestation) or extremely low birth weight (< 1000 g) infants				NP

mortality	0.58 (0.46 to 0.75)	14	1789/3583	4.5	YANG Y 2014 [4]	enteral administration of any probiotic started within the first 10 days of life	at least 7 days	All RCTs	preterm infants (without consideration of birth weight)	probiotic vs. Placebo group	RR	fixed	No obvious bias
The effect of probiotics in decreasing death rate	0.69 (0.55 to 0.87)	27	4399/8717	36.2	JIANG T L 2020 [5]	probiotics	NP	All RCTs	preterm infants < 37 weeks and/or birth weight < 2500 g	Probiotics vs. control	RR	random	No obvious bias
	0.52 (0.34 to 0.80)	12	1635/3175	42.7		mixed probiotics							
Incidence of death.	0.48 (0.36 to 0.64)	20	4286	0.0	LIU D P 2020[6]	administration of Lactobacillus for preventing NEC	NP	All RCTs	neonates of gestational age < 37 weeks	Lactobacillus with placebo	RR	fixed	No obvious bias
Incidence of death.	0.74 (0.60 to 0.92)	15	2579/5053	29.0	ZHU X L 2019[7]	bifidobacteria treatment for preventing NEC	NP	All RCTs	neonates with gestational age (GA) < 37 weeks	Bifidobacteria vs. control	RR	fixed	NP
Mortality	1.17 (0.54 to 2.57)*	7	NP	20.4	SKONIECZNA-ZYDECKA K 2018 [8]	treatment with pro-/pre-/synbiotics	Preoperation 0-15 days, postoperation 0-until discharge	All RCTs	surgical patients	Probiotics vs. No probiotics	SMD	random	NP
mortality	0.58 (0.36 to 0.94)	9	924	0.0	CHI C 2019[9]	prebiotics	7-57 days	All RCTs	low birth weight infants (<2500 g) or preterm infants (<36 weeks)	vs. placebo	RR	random	0.73
operative mortality	1.39 (0.57 to 3.44)*	5	NP	NP	Kinross JM 2013[10]	prebiotic	NP	All RCTs	patients underwent abdominal surgery with the use of a prebiotic, probiotic, or a synbiotic agent	Vs. either received a placebo or received no therapy	WMD	random	NA

* No statistical significance; CI, confidence interval; RCT, randomized controlled trial; RR, relative risk; HR, hazard ratio; MD, mean

difference; SMD, standard mean difference; WMD, weighted mean difference; OR, odds ratio; NA, not available; NP, not published.

References

1. Dalal, R.; Mcgee, R.G.; Riordan, S.M.; Webster, A.C. Probiotics for people with hepatic encephalopathy. *Cochrane Db. Syst. Rev.* **2021**, *2021*, doi: 10.1002/14651858.CD008716.pub3.
2. Dermyshi, E.; Wang, Y.; Yan, C.; Hong, W.; Qiu, G.; Gong, X.; Zhang, T. The "Golden Age" of Probiotics: A Systematic Review and Meta-Analysis of Randomized and Observational Studies in Preterm Infants. *Neonatology* **2017**, *112*, 9-23, doi: 10.1159/000454668.
3. Sharif, S.; Meader, N.; Oddie, S.J.; Rojas-Reyes, M.X.; Mcguire, W. Probiotics to prevent necrotising enterocolitis in very preterm or very low birth weight infants. *Cochrane Db. Syst. Rev.* **2020**, *2020*, D5496, doi: 10.1002/14651858.CD005496.pub5.
4. Yang, Y.; Guo, Y.; Kan, Q.; Zhou, X.G.; Zhou, X.Y.; Li, Y. A meta-analysis of probiotics for preventing necrotizing enterocolitis in preterm neonates. *Braz. J. Med. Biol. Res.* **2014**, *47*, 804-810, doi: 10.1590/1414-431X20143857.
5. Jiang, T.; Zhang, H.; Xu, X.; Li, H.; Yang, J. Mixed probiotics decrease the incidence of stage II-III necrotizing enterocolitis and death: A systematic review and meta-analysis. *Microb. Pathogenesis* **2020**, *138*, 103794, doi: 10.1016/j.micpath.2019.103794.
6. Liu, D.; Shao, L.; Zhang, Y.; Kang, W. Safety and efficacy of Lactobacillus for preventing necrotizing enterocolitis in preterm infants. *Int. J. Surg.* **2020**, *76*, 79-87, doi: 10.1016/j.ijsu.2020.02.031.
7. Zhu, X.; Tang, X.; Qu, F.; Zheng, Y.; Zhang, W.; Diao, Y. Bifidobacterium may benefit the prevention of necrotizing enterocolitis in preterm infants: A systematic review and meta-analysis. *Int. J. Surg.* **2019**, *61*, 17-25, doi: 10.1016/j.ijsu.2018.11.026.
8. Skonieczna-żydecka, K.; Kaczmarczyk, M.; łoniewski, I.; Lara, L.; Koulaouzidis, A.; Misera, A.; Maciejewska, D.; Marlicz, W. A Systematic Review, Meta-Analysis, and Meta-Regression Evaluating the Efficacy and Mechanisms of Action of Probiotics and Synbiotics in the Prevention of Surgical Site Infections and Surgery-Related Complications. *Journal of Clinical Medicine* **2018**, *7*, 556, doi: 10.3390/jcm7120556.
9. Chi, C.; Buys, N.; Li, C.; Sun, J.; Yin, C. Effects of prebiotics on sepsis, necrotizing enterocolitis, mortality, feeding intolerance, time to full enteral feeding, length of hospital stay, and stool frequency in preterm infants: a meta-analysis. *Eur. J. Clin. Nutr.* **2019**, *73*, 657-670, doi: 10.1038/s41430-018-0377-6.
10. Kinross, J.M.; Markar, S.; Karthikesalingam, A.; Chow, A.; Penney, N.; Silk, D.; Darzi, A. A Meta-Analysis of Probiotic and Synbiotic Use in Elective Surgery. *Jpen.-Parenter. Enter.* **2013**, *37*, 243-253, doi: 10.1177/0148607112452306.
