# Supplementary material for: Gastrointestinal Microbiome and Multiple Health Outcomes: Umbrella Review
Source: Nutrients. 2022 Sep 9;14(18):3726. doi: 10.3390/nu14183726 (PMC9505003; doi:10.3390/nu14183726)
Supplement: Supplementary file 1 [file nutrients-14-03726-s001.zip › Supplementary Table S3.pdf]

Supplement Table S3. Prebiotics/probiotics/synbiotics and immune and metabolic outcomes.

outcome(s) of interest	estimated summary effect (95% CI)	number of studies / total studies	number of intervention group or total participants	Heterogeneity (I^2, %)	the first author + year of publication	Intervention	Duration of Intervention/follow-up	study design	populations	outcome comparison	meta-analysis metric	type of effect model	publication bias
total cholesterol concentrations after prebiotic supplementation	-0.25 (-0.48 to -0.02)	9	313	14.4	BESERRA B 2015[1]	prebiotic OR fructo-oligosaccharide OR fructooligosaccharide OR galacto-oligosaccharide OR galactooligosaccharide OR inulin OR lactulose OR FOS OR GOS OR Oligofructose OR synbiotic	NP	All RCTs	Overweight (body mass index ≥ 25 kg/m²) or obese individuals	any versus none	SMD	random	No serious bias
LDL-c concentrations after prebiotic supplementation	-0.2 (-0.44 to -0.00)	9	313	0.0									
triglycerides concentrations after synbiotics supplementation	-0.43 (-0.70 to 0.15)*	3	148	0.0									
fasting insulin after synbiotics supplementation	-0.39 (-0.75 to -0.02)	3	180	0.0									
triglycerides concentrations after prebiotic supplementation	-0.72 (-1.20 to -0.23)	9	313/513	32.7									
HDL-c concentrations after prebiotic supplementation	0.49 (0.01 to 0.97)	9	313/513	0.0									
diamine oxidase	-0.78(-0.93 to -0.63)	3	147/293	0.0	CHEN X R 2021[2]	early enteral nutrition (EEN)	≥ 14 days	All RCTs	All patients met the	early enteral nutrition	MD	Random	No serious

D-lactic acid	-0.06(-0.07 to -0.05)	4	162/324	0.0		combined with probiotics			criteria of severe stroke set by the fourth national Cerebrovascular Disease Conference, and were diagnosed by head CT and MRI; the onset was ≤3 days; Glasgow Coma Scale ≤9 points; gastrointestinal function was normal or basically normal. Exclusion criteria were: patients with previous history of gastrointestinal and metabolic diseases, malignant tumor and malnutrition; patients with severe heart, liver, and kidney failure.	(EEN) combined with probiotics versus EEN			bias
dysbacteriosis rate	0.17(0.07 to 0.41)	3	132/262	0.0							OR		
albumin	3.38(2.74 to 4.02)	15	637/1271	45.0							MD		
prealbumin	32.20(24.42 to 39.98)	7	302/601	44.0									
total protein	4.91(3.20 to 6.62)	7	336/672	77.0									
hemoglobin	9.62(7.92 to 11.32)	10	437/872	0.0									
immunoglobulin A	0.23(0.12 to 0.34)	4	144/280	20.0									
immunoglobulin G	0.33(0.21 to 0.45)	3	106/208	0.0									
immunoglobulin M	0.14(0.00 to 0.28)	4	147/290	46.0									
TC concentrations	-3.04(-4.88 to -1.21)mg/dL	12	767	45.9	YAN S 2019[3]	probiotics	6 weeks to 6 months	All RCTs	overweight or obesity was defined according to local standards	the probiotics vs. control groups	WMD	random	NP
LDL concentrations	-2.28(-3.60 to -0.96)mg/dL	11	737	36.9								fixed	
HDL concentrations	-0.26(-2.39 to 1.87)mg/dL*	12	767	95.5								random	
Triglyceride (TG) concentrations	-0.86(-2.54 to 0.83)17.0mg/dL*	11	726	17.0								fixed	
TC	-1.23(-3.52 to	5	NP	0.0		single probiotic						fixed	

concentrations	1.06)mg/dL*												
TC concentrations	-3.82(-6.14 to -1.50)mg/dL	7		49.8		multiple probiotic						fixed	
TG concentrations	9.10(1.38 to 16.81)mg/dL	4		0.0		single probiotic						fixed	
TG concentrations	-1.36(-3.08 to 0.37)mg/dL*	7		0.0		multiple probiotic						fixed	
Effects on Fasting Plasma Glucose (FPG)	-0.35 (-0.67 to -0.02)	6	215/436	64.0	WANG Z 2019[4]	Probiotics	NP	All RCTs	adults (≥18 years old) individuals with a BMI > 25 kg m-2	the probiotics vs. control groups	SMD	random	0.791
Effects on C-reactive protein	-0.45 (-0.99 to 0.08) mg/L*	9	314/649	84.0	PONTES K S D S 2021 [5]	probiotics	NP	All RCTs	adults (18 years or older) with overweight or obesity defined according to local standards for body mass index (BMI)	Probiotics versus placebo	MD	random	No serious bias
Effects on tumor necrosis factor α	-0.16 (-0.24 to -0.08) pg/ml	4	96/191	11.0									
Effects on interleukin-6	0.05 (-0.28 to 0.39) pg/ml*	3	66/142	57.0									
Effects on glucose	-0.07 (-0.26 to 0.13) mmol/L*	13	418/977	92.0									0.01
Effects on fasting insulin	-0.85 (-1.50 to -0.21) mcU/ml	10	322/776	30.0									No serious bias
Effects on glycated hemoglobin (HbA1C)	-0.03 (-0.14 to 0.09) %*	7	246/505	84.0									
adiponectin concentration	-0.04 (-0.28 to 0.19)*	8	NP	32.5	ROUHANI M H 2019[6]	probiotic supplementation	6-24 weeks	All RCTs	adults	probiotic versus controls	Hedges's adjusted g	random	0.65
leptin concentration	-0.30 (-0.86 to 0.26)*	6		81.6									0.81
fasting plasma glucose	-3.45 (-6.03 to -0.88)	5	169/337	84.0	COZZOLINO M 2020 [7]	therapy with probiotics or synbiotics.	administration of probiotics or synbiotics at least for 8 weeks	All RCTs	women with Polycystic ovary syndrome (PCOS) according to	administration of probiotics or Synbiotics versus without	MD	random	NP
Homeostatic Model Assessment of Insulin	-2.31 (-3.84 to -0.77)	5	169/337	94.0									

Resistance (HOMA-IR)									Rotterdam criteria undergoing therapy with probiotics or symbiotic	therapy with probiotics or synbiotics or placebo			
Insulin-Sensitivity Check Index	-0.62 (-1.07 to -0.17)	5	169/337	89.0									
Triglycerides	-23.35 (-35.23 to -11.47)	2	60/120	0.0									
total testosterone serum	-0.23 (-0.36 to -0.11)	4	113/226	52.0									
High sensitivity C reactive protein	-1.69 (-2.38 to -1.01)	4	120/240	49.0									
Nitric oxide	2.72 (0.21 to 5.23)	3	90/180	26.0									
Total antioxidant capacity	70.55 (38.84 to 102.25)	3	90/180	46.0									
Total glutathione	22.42 (2.08 to 42.75)	3	90/180	25.0									
fasting plasma glucose (FPG)	-1.35 (-2.22 to -0.49)	8	496	94.6	LI Y L 2021 [8]	probiotics, prebiotics, synbiotics intake separately or in combination with other drugs	8 weeks to 3 months	All RCTs	women with a definite diagnosis of Polycystic ovary syndrome (PCOS)	probiotics, prebiotics, synbiotics intake separately or in combination with other drugs versus placebo	SMD	random	No serious bias
fasting insulin (FINS)	-0.68 (-1.08 to -0.27)	7	216/434	76.7									
homeostatic model of assessment for insulin resistance (HOMA-IR)	-0.73 (-1.15 to -0.31)	7	434	78.1									
quantitative insulin sensitivity check index (QUICKI)	2.00 (-0.79 to 3.22)*	6	189/379	96.1									
triglycerides (TG)	-0.85 (-1.59 to -0.11)	7	428	92.2									
Total cholesterol	-1.09 (-1.98 to -0.21)	7	428	94.3									

(TC)													
high-density lipoprotein cholesterol (HDL-c)	0.53 (-0.33 to 1.39) *	7	428	94.3									
low-density lipoprotein cholesterol (LDL-c)	-0.84 (-1.64 to -0.03)	7	428	93.4									
very-low-density lipoprotein cholesterol (VLDL-c)	-0.44 (-0.70 to -0.18)	4	235	0.0									
CRP	-0.63 (-1.37 to 0.10)*	9	558	93.9									
total anti-oxidant capacity (TAC)	0.04 (-0.75 to 0.83) *	3	82	83.9	DENG H Y 2020 [9]	Probiotic	12 weeks	All RCTs	Adult human participants who had a diagnosis of AD or MCI (aged over 18 y)	Probiotics versus pla- cebo	SMD	random	NP
total glutathione (GSH)	0.04 (-0.28 to 0.35)	3	82	44.1								fixed	
malondialdehyde (MDA)	-0.60 (-0.91 to -0.28)	3	82	0.0									
nitric oxide (NO)	-0.16 (-0.47 to 0.15)	3	82	4.4									
high-sensitivity C-reactive protein (hs-CRP)	-0.57(-0.95 to -0.20)	2	57	0.0									
Plasma ammonia concentration (at one month)	-5.55 (-10.67 to -0.42) μmol/L	5	179/357	56.0	DALAL R 2017 [10]	probiotics	at one month	randomised clinical trials	people with any grade of acute or chronic hepatic encephalopathy	probiotics in any dosage vs. placebo or no intervention, or with any other treatment in people with hepatic encephalopathy.	MD	random	NP
Plasma ammonia concentration (at 2 months)	-5.11 (-14.56 to 4.34) μmol/L*	4	109/211	62.0			at 2 months						
Plasma ammonia concentration	-8.29 (-13.17, -3.41) μmol/L	10	357/705	79.0			follow-up: 1 month to 6 months						

Effect on plasma ammonia levels (week 4)	-6.86 (-19.01 to 5.30) μmol/L*	5	132/255	98.0	CAO Q 2018 [11]	Probiotic treatment	4-12 weeks	All RCTs	Patients with Minimal hepatic encephalopathy (MHE)	Probiotic treatment vs placebo or no treatment	MD	random	NP
Effect on plasma ammonia levels (week 4)	-0.80 (-1.56 to -0.08) μmol/L	4	112/220	0.0									
Reduction in serum endotoxin	-0.12 (-0.23 to -0.02)	3	58/109	10.0									
Effect on plasma ammonia levels (week 4)	-0.33 (-5.39 to 4.74) μmol/L*	4	164/325	0.0						Probiotic treatment vs lactulose treatment	MD		
serum ALT concentrations	-8.05 (-13.07 to -3.04) IU/L	16	990	93.0	KHALESI S 2018[12]	Probiotic; Synbiotic; prebiotic	NP	RCT or non-randomised controlled	Adults older than 18 years of age	probiotics vs. control	MD	random	NP
serum AST concentrations	-7.79 (-13.93 to -1.65)IU/L	16	990	97.7									
serum ALP level	-0.27 (-4.00 to 3.47)IU/L*	6	518	70.0									
serum GGT levels: gamma-glutamyl transpeptidase	-8.40 (-12.61 to -4.20)IU/L	8	438	53.0									
serum levels of albumin	-0.29 (-0.74 to 0.16)*	11	662	40.0									
serum bilirubin level	0.95 (0.48 to 1.42)μmol/L	13	806	4.0									
ALT	-7.31 (-9.89 to -4.74) U/L	27	NP	98.4	HERNANDEZ-SAAVEDRA D 2018 [13]	Any prebiotic or probiotic treatment, or a combination of both (synbiotic)	NP	All RCTs	Male and female patients of any age that presented at least 1 of the following: NAFLD, steatosis, liver fibrosis, steatohepatitis	Vs. placebo	MD	random	No serious bias
AST	-4.94 (-6.89 to -2.98) U/L	25		98.8									
γ-GT: gamma-glutamyl transferase	-7.86 (-11.36 to -4.36) U/L	9		94.8									
TNF-α	-2.04 (-4.70 to 0.61) ng/mL*	6		99.7									

hepatic cytokine CRP	-0.74 (-1.85 to 0.37) mg/L*	6		99.3									
total serum cholesterol	-7.97 (-11.41 to -4.54) mg/dL	15		99.3									
Serum LDL-c	-3.81 (-8.31 to 0.69) mg/dL*	12		97.0									
HDL-c	0.56 (-1.55 to 2.67)*	12		99.8									
Circulating TAG	-8.28 (-16.35 to -0.22)	16		97.8									
necrosis factor-α	-0.59 (-1.34 to 0.16)*	6	276	88.0	KHAN M Y 2019[14]	probiotics and/or synbiotics	NP	All RCTs	adult patients older than 18 years of age with NAFLD or NASH	Vs. placebo	SMD	random	NP
LDL	-0.48 (-1.04 to -0.08)	7	316	82.0									
HDL	-0.03 (-0.29 to 0.23)*	7	316	24.0									
total cholesterol level.	-0.48 (-0.94 to -0.01)	8	391	79.0									
triglycerides level.	-0.23 (-0.48 to 0.03)*	8	391	36.0									
insulin resistance	-0.12 (-0.63 to 0.40)*	6	289	93.0							MD		
fasting blood sugar level	-0.47 (-1.02 to 0.08)*	6	305	81.0							SMD		
high-sensitivity C-reactive protein level	-0.45 (-0.76 to -0.15)	4	234	25.0									
CRP	-0.40 (-0.79 to -0.02)	6	NP		SKONIECZNA-ZYDECKA K 2018 [15]	treatment with pro-/pre-/synbiotics	Preoperation 0-15 days, postoperation 0-until discharge	All RCTs	surgical patients	Probiotics vs. No probiotics	SMD	random	NP
IL-6	-0.41 (-0.70 o -0.12)	4											
white blood cells (WBC)	-0.60 (-1.45 to 0.24)*	6											
lactulose/mannitol (L/M) ratio	-0.28 (-0.82 to 0.27)*	4											

the concentration of butyrate ratio	0.67 (0.37 to 0.97)	4											
short chain fatty acids (SCFAs)-acetic	1.78 (0.80 to 2.76)	4											
propionic acids	0.46 (0.18 to 0.73)	4											
hs-CRP	-0.39 (-0.50 to -0.28)	31	NP	83.8	MILAJERDI A 2020[16]	probiotic	NP	All RCTs	adult	probiotics vs. placebo	SMD	fixed	NP
serum IL-6	-0.37 (-0.51 to -0.24)	16		69.7									
serum TNF-a concentrations	-0.21 (-0.34 to -0.08)	18		85.5									
serum Interleukin-10 (IL-10)	0.21 (0.04 to0.38)	11		48.5									
serum interleukin-1 beta (IL-1β)	-0.17 (-0.37 to 0.02)*	9		80.7									
serum IL-12	-0.47 (-0.67 to -0.27)	8		85.2									
serum IL-8	-0.01 (-0.30 to 0.28)*	5		73.0									
Interferon-gama (IFN-γ)	-0.08 (-0.31 to 0.15)*	5		0.0									
IL-17	006 (-0.34 to 0.46)*	3		0.0									
IL-4	-0.48 (-0.76 to -0.20)	3		0.0									
CRP	-0.77 (-1.48 to -0.05) mg/L	4	153	75.0	PAN H D 2017[17]	probiotic	Follow up: 8-48 weeks	All RCTs	patients with a clear diagnosis of RA and stable treatment of disease-modifying anti-rheumatic drugs	probiotics vs. Placebo or no probiotics	SMD	random	NP
TNF-α	-1.35 (-1.99 to -0.71)	3	118	25.0							MD		
IL-6	-8.69 (-27.32 to 9.94)*	3	118	62.0									
IL-1β	-6.13 (-11.41	3	118	50.0									

	to -0.86)								(DMARDs)				
IL-10.	0.63 (-0.05 to 1.31)*	3	118	45.0									
CD4 counts	3.86 (-24.72 to 32.45) *	16	1025	55.7	FU Y S 2020[18]	probiotics, prebiotics, or synbiotics	NP	All RCTs	HIV-1-infected adults over 18 years of age	Vs. placebo or control groups	WMD	random	0.936
CD4 counts	21.24 (-12.95 to 55.39)*	10	385	0.0	ZHANG X L 2021[19]	probiotic	2-48 weeks	RCTs, clinical cohort studies, pilot study with controls, or other clinical studies	HIV/AIDS patients and patients diagnosed with similar recognized criteria based on laboratory evidence or methods of HIV infection	Probiotic vs. No probiotic	MD	fixed	No serious bias
lymphocyte T cell count	0.00 (-0.03 to 0.02) cells*10⁹/L*	4	NP	83.8	TAVAKOLY R 2021[20]	probiotics (supplement or fortified food)	4-20 weeks	All RCTs	adult athletes	placebo or non-fortified food	WMD	random	0.005
T helper	0.00 (-0.07 to 0.07) cells*10⁹/L*	5		0.0									0.089
T cytotoxic	-0.08 (-0.15 to -0.01) cells*10⁹/L	5		59.1									0.53
lymphocyte B	0.01 (-0.07 to 0.09)*	9		0.0									0.32
Lymphocyte	0.01 (-0.05 to 0.08)*	5		27.0									0.84
NK cells	0.04 (-0.01 to 0.08)*	4		3.2									0.679
leukocyte	0.23 (-0.09 to 0.55)*	10		49.0									0.446
monocyte	-0.00 (-0.03 to 0.02)*	8		3.8									0.577
neutrophil cells count	0.14 (-0.06 to 0.33)*	7		0.0									0.460
IgM	-0.22 (-0.63 to 0.19)*	3		0.0									0.885

IgA	0.24 (-0.09 to 0.57)*	4		14.0									0.729
IgA secretion rate	4.21 (-8.35 to16.77)*	3		13.8									0.208
The efficacy of syn and probiotics on hs-CRP	-0.58 (-0.79 to-0.37)	8	359	0.0	ASKARI G 2021[21]	Synbiotic or probiotics	NP	clinical trials	age >12 years old and disease duration between 2 and 20 years	Vs. Control group	SMD	random	0.195
IL-10	-0.12 (-2.11 to 1.87) pg/ml*	5	184	93.9							WMD		0.660
IL-6	-7.79 (-13.81 to -1.77) pg/ml	6	214	91.9									0.191
TNF-α	-1.05 (-2.01 to -0.10) pg/ml	5	184	85.2									0.102
ESR: Erythrocyte Sedimentation Rate	3.05 (-7.60 to 13.7) mm/h*	3	90	71.5									0.450
GSH: Glutathione	1311 (-55.68 to 81.91) μmol/L	4	222	88.8									0.099
MDA: Malondialdehyde	-0.36 (-0.68 to -0.04)	5	268	41.0							SMD		0.058
TAC	0.08 (-0.16 to -0.32)	5	268	0.0									0.286
HOMA-IR	-0.71 (-1.05 to -0.37)	4	222	0.0							WMD		0.133
HOMA-β	-15.18 (-22.08 to -8.28)	3	174	0.0									0.096
serum creatinine	0.08 (-0.13 to 0.28) mg/dL*	5	126	0.0	JIA L P 2018[22]	probiotics	1-6 momths	All RCTs	adult chronic kidney disease (CKD) patients	probiotics vs controls	MD	random	NP
blood urea	-1.38 (-9.26 to 6.50)*	3	164	0.0									
p-cresyl sulfate	-0.57 (-0.99 to -0.14)	3	125	25.0							SMD		
hemoglobin	0.21 (-0.48 to 0.91)*	3	93	20.0							MD		

IL-6	0.37 (0.03 to 0.72)*	3	134	0.0							SMD		
CRP	0.49 (-6.45 to 3.11)*	3	NP	28.0							MD		
urea	-8.17 (-24.57 to 8.23)*	6	259	73.0	TAO S 2019 [23]	probiotics (specified probiotic or probiotic mixes) in any form (e.g., capsule, tablet or other food supplements or components), the probiotics could be administered at any dose and for any duration	Follow up: 6 weeks - 6 months	All RCTs	the participants were diagnosed with CKD, whether undergoing dialysis or not	probiotics vs placebo	MD	random	0.293
uric acid	-0.43 (-1.19 to 0.33)*	3	131	67.0									0.659
C-reactive protein	-0.48 (-1.29 to 0.33)*	6	227	52.0									0.770
creatinine	-0.18 (-0.82 to 0.47)	6	259	69.0									0.686
serum C-reactive protein	-0.37 (-0.72 to 0.03)*	7	178	0.0	THONGPRAYOON C 2019[24]	probiotic	NP	clinical trials	end-stage renal disease (ESRD) patients	Vs. Control group	SMD	random	0.09
total cholesterol	-0.25 (-0.46 to -0.04)	7	NP	0.0	BAKHTIARY M 2021 [25]	probiotic, prebiotic, or synbiotic	Follow up: 4-12 weeks	All RCTs	chronic kidney disease (CKD) patients	Vs. usual care or placebo	SMD	random	NP
HDL levels	-0.01 (-0.62 to 0.60)*	6		84.6									
LDL	-0.19 (-0.49 to 0.10)*	6		45.8									
triglyceride	-0.10 (-0.33 to 0.12)*	7		6.1									
VLDL	-0.21 (-0.47 to 0.05)*	4		0.0									
Fasting blood glucose (FBG)	-0.41 (-0.65 to -0.17)	5		0.0									
homeostatic model assessment of insulin resistance (HOMA-IR)	-0.63 (-0.95 to -0.30)	5		43.3									

insulin levels	-0.49 (-0.90 to -0.08)	5		65.2									
quantitative insulin sensitivity check index (QUICKI)	0.78 (0.51 to 1.05)	4		0.0									
hs-CRP	-0.52 (-0.81 to -0.22)	8		52.7									
CRP	0.21 (-0.17 to 0.59)*	4		0.0									
IL-6	-0.30 (-0.84 to 0.23)*	4		55.8									
Malondialdehyde	-0.79 (-1.22 to -0.37)	6		69.8									
glutathione	0.52 (0.19 to 0.86)	4		37.0									
nitric oxide	0.06 (-0.20 to 0.32)*	4		1.0									
serum urea	-0.30 (-2.20 to 1.61) mmol/L*	9	345	53.0	MCFARLANE C 2019[26]	prebiotics, probiotics, or synbiotics for a duration of at least 1 week	NP	RCT including crossover, cluster, or quasi-RCT designs	participants with CKD as defined by the Kidney Disease Outcome Quality Initiative Guidelines	Vs. comparison groups	MD	random	NP
Serum indoxyl sulfate	-0.02 (-0.09 to 0.05) mg/dL*	4	144	0.0									
serum PCS: p-cresyl sulfate	-0.13 (-0.41 to 0.15) mg/dL*	4	144	0.0									
HbA1c	-2.17 (-4.37 to 0.03) mmol/mol*	12	717	66.0	BOCK P M 2021[27]	any probiotic, prebiotic or synbiotic supplementation or combination of interventions with the aim of adjusting the gut microbiota	Follow up: 4-12 weeks	clinical trials	adult participants with type 1 or 2 diabetes diagnosis	Vs. Control group	MD	Random	No serious bias
fasting blood glucose	-0.58 (-0.86 to -0.30) mmol/l	36	1982	60.0									
insulinemia	-10.51 (-16.68 to -4.33) pmol/l	21	1194	74.0									
total cholesterol	-0.14 (-0.26 to -0.02) mmol/l	27	1524	39.0									
triacylglycerol	-0.11 (-0.20 to -0.02) mmol/l	29	1619	21.0									

HDL-cholesterol	0.04 (0.01 to 0.07) mmol/l	29	1619	24.0									
LDL-cholesterol	-0.05 (-0.14 to 0.05) mmol/l*	27	1531	37.0									
hs-CRP	-0.38 (-0.51 to -0.24)	13	NP	43.0	ZHENG H J 2019[28]	probiotics or synbiotics	6-12 weeks	All RCTs	diabetic patients (defined as age > 18 years)	Vs. placebo	SMD	fixed	0.3
TAC	0.31 (0.09 to 0.52)	13		58.9									0.10
MDA	-0.61 (-0.89 to -0.32)	10		66.7									0.03
NO	0.62 (0.25 to 0.99)	8		76.3								random	0.035
GSH	0.41 (0.26 to 0.55)	11		26.8								fixed	0.533
HbA1c	-0.24 (-0.44 to -0.04) %	8	489	27.0	TAO Y 2020[29]	probiotic	6-12 weeks	All RCTs	T2DM patients	probiotics vs placebo	MD	random	0.29
FBG	-0.44 (-0.74 to -0.15) mmol/L	14	842	0.0									0.86
HOMA-IR	-1.07 (-1.58 to -0.56)	8	524	18.0									0.07
blood glucose	-0.18 (-0.30 to -0.05)	4	186	0.0	CHI C 2020[30]	Probiotics	3-24 weeks	All RCTs	hypertension patients	probiotics vs placebo	MD	random	0.24
FBS	-2.28 (-7.37 to 2.80) mg/dL*	8	NP	81.0	HADI A 2021 [31]	probiotics or synbiotics	3-28 weeks	All RCTs	adults with metabolic syndrome	probiotics vs placebo	MD	random	0.30
HOMA-IR	-0.07 (-0.63 to 0.49)*	6		73.6									0.74
Insulin	-0.25 (-0.56 to 0.07)*	7		42.7						probiotics vs control group			0.36
TC	-6.66 (-13.25 to 0.07) mg/dL*	7		28.8									0.83
TG	-3.15 (-24.59 to 18.29)mg/dL*	7		68.5									0.22

LDL-c	-6.60 (-14.41 to 1.21) mg/dL*	7		52.7									0.69
HDL-c	0.27 (-2.80 to 3.34) mg/dL *	7		75.7									0.89
cortisol	-0.28 (-0.65 to 0.09)*	3	NP	0.0	AS'HABI A 2020 [32]	probiotics (supplement or food)	2-20 weeks	All RCTs	athletes	Vs. control	SMD	random	0.20
HDL- C	2.51 (1.04 to 6.05) mg/dL	6		50.3									0.25
LDL-C	-4.06 (-14.70 to 6.58) mg/dL*	3		0.0									0.98
TC	-5.99 (-10.83 to -6.04) mg/dL	6		67.5									0.70
TG	-32.26 (-60.44 to -4.08) mg/dL	6		93.0									0.48
serum calcium levels	3.82 (1.05 to 6.59)	4	NP	98.0	EJTAHED H 2021[33]	probiotic	NP	clinical trial	adults	Vs. control diets	SMD	Random	No serious bias
serum phosphorus levels	1.14 (-0.44 to 2.73)*	4		96.1									
parathyroid hormone (PTH)	-5.35 (-9.83 to -0.86)	3		98.2									
urinary calcium	4.85 (1.16 to 8.53)	3		97.6									
CRP	-0.60 (-0.98 to -0.23)	7	342	64.0	MCLOUGHLIN R F 2017[34]	prebiotics, delivered orally, intravenously, or perrectum (enema)	2-12 weeks	All RCTs	human participants of any age and sex	vs. placebo or control	SMD	random	NP
IL-6	0.35 (-0.84 to 0.13)*	6	313	75.0									
TNF-α	-0.49 (-1.20 to 0.22)*	4	219	84.0									
postprandial ghrelin	-71.66 (-148.83 to 5.50) pg/ml*	3	NP	84.6	DA SILVA BORGES D 2020[35]	prebiotic or synbiotic	NP	clinical trials	overweight or obesity diagnosis (BMI ≥ 25 kg/m²), (3)	Vs. control	WMD	random	NP

									adults and older adults (≥ 18 y)				
levels of TNF-α	-0.24 (-0.69 to 0.21)*	7	NP	82.7	QU H 2019[36]	microbiota-driven therapy (probiotic, prebiotic or symbiotic)	14-180 days	the study described a randomized, controlled, parallel or crossover trial	healthy elderly individuals with age>60 years	vs. placebo	SMD	random	0.37
IL-6	-0.13 (-0.74 to 0.49)*	6		90.7									0.26
IL-10	1.00 (-0.15 to 2.15)*	6		96.3									0.20
CRP	-1.28 (-2.62 to 0.06)*	4		96.2									NP
IL-8	-0.03 (-0.67 to 0.61)*	4		88.0									
CRP	0.02 (-0.32 to 0.36)*	3	135	0.0	ZHU H F 2020[37]	treated with microecological preparations (probiotics, prebiotics, synbiotics, or a combination of two preparations)	15 days to 6months	All RCTs	obese adults (age ≥18 years) who had undergone bariatric surgery (a type of surgery was not restricted)	Vs. placebo or conventional treatment	SMD	Random	NP
IL-6	-0.10 (-0.81 to 0.61)*	3	135	0.0								random	
TNF-α	-0.29 (-0.64 to 0.05)*	3	135	0.0								fixed	

* No statistical significance; CI, confidence interval; RCT, randomized controlled trial; RR, relative risk; HR, hazard ratio; MD, mean difference; SMD, standard mean difference; WMD, weighted mean difference; OR, odds ratio; NA, not available; NP, not published.

References

1. Beserra, B.T.S.; Fernandes, R.; Do Rosario, V.A.; Mocellin, M.C.; Kuntz, M.G.F.; Trindade, E.B.S.M. A systematic review and meta-analysis of the prebiotics and synbiotics effects on glycaemia, insulin concentrations and lipid parameters in adult patients with overweight or obesity. *Clin. Nutr.* **2015**, *34*, 845-858, doi: 10.1016/j.clnu.2014.10.004.
2. Chen, X.; Hu, Y.; Yuan, X.; Yang, J.; Ka, L. Effect of early enteral nutrition combined with probiotics in patients with stroke: a meta-analysis of randomized controlled trials. *Eur. J. Clin. Nutr.* **2022**, *76*, 592-603, doi: 10.1038/s41430-021-00986-3.
3. Yan, S.M.; Tian, Z.W.; Li, M.; Li, B.; Cui, W.W. Effects of probiotic supplementation on the regulation of blood lipid levels in overweight or obese subjects: a meta-analysis. *Food Funct.* **2019**, *10*, 1747-1759, doi: 10.1039/c8fo02163e.
4. Wang, Z.; Xin, S.; Ding, L.; Ding, W.; Hou, Y.; Liu, C.; Zhang, X. The Potential Role of Probiotics in Controlling Overweight/Obesity and Associated Metabolic Parameters in Adults: A Systematic Review and Meta-Analysis. *Evid.-Based Compl. Alt.* **2019**, *2019*, 1-14, doi: 10.1155/2019/3862971.

5. Pontes, K.S.D.S.; Guedes, M.R.; Cunha, M.R.D.; Mattos, S.D.S.; Barreto Silva, M.I.; Neves, M.F.; Marques, B.C.A.A.; Klein, M.R.S.T. Effects of probiotics on body adiposity and cardiovascular risk markers in individuals with overweight and obesity: A systematic review and meta-analysis of randomized controlled trials. *Clin. Nutr.* **2021**, *40*, 4915-4931, doi: 10.1016/j.clnu.2021.06.023.
6. Rouhani, M.H.; Hadi, A.; Ghaedi, E.; Salehi, M.; Mahdavi, A.; Mohammadi, H. Do probiotics, prebiotics and synbiotics affect adiponectin and leptin in adults? A systematic review and meta-analysis of clinical trials. *Clin. Nutr.* **2019**, *38*, 2031-2037, doi: 10.1016/j.clnu.2018.09.033.
7. Cozzolino, M.; Vitagliano, A.; Pellegrini, L.; Chiurazzi, M.; Andriasani, A.; Ambrosini, G.; Garrido, N. Therapy with probiotics and synbiotics for polycystic ovarian syndrome: a systematic review and meta-analysis. *Eur. J. Nutr.* **2020**, *59*, 2841-2856, doi: 10.1007/s00394-020-02233-0.
8. Li, Y.; Tan, Y.; Xia, G.; Shuai, J. Effects of probiotics, prebiotics, and synbiotics on polycystic ovary syndrome: a systematic review and meta-analysis. *Crit. Rev. Food Sci.* **2021**, 1-17, doi: 10.1080/10408398.2021.1951155.
9. Den, H.; Dong, X.; Chen, M.; Zou, Z. Efficacy of probiotics on cognition, and biomarkers of inflammation and oxidative stress in adults with Alzheimer's disease or mild cognitive impairment — a meta-analysis of randomized controlled trials. *Aging* **2020**, *12*, 4010-4039, doi: 10.18632/aging.102810.
10. Dalal, R.; Mcgee, R.G.; Riordan, S.M.; Webster, A.C. Probiotics for people with hepatic encephalopathy. *Cochrane Db. Syst. Rev.* **2021**, *2021*, doi: 10.1002/14651858.CD008716.pub3.
11. Cao, Q.; Yu, C.; Yang, S.; Cao, H.; Chen, P.; Deng, M.; Li, L. Effect of probiotic treatment on cirrhotic patients with minimal hepatic encephalopathy: A meta-analysis. *Hepatob. Pancreat. Dis.* **2018**, *17*, 9-16, doi: 10.1016/j.hbpd.2018.01.005.
12. Khalesi, S.; Johnson, D.W.; Campbell, K.; Williams, S.; Fenning, A.; Saluja, S.; Irwin, C. Effect of probiotics and synbiotics consumption on serum concentrations of liver function test enzymes: a systematic review and meta-analysis. *Eur. J. Nutr.* **2018**, *57*, 2037-2053, doi: 10.1007/s00394-017-1568-y.
13. Loman, B.R.; Hernández-Saavedra, D.; An, R.; Rector, R.S. Prebiotic and probiotic treatment of nonalcoholic fatty liver disease: a systematic review and meta-analysis. *Nutr. Rev.* **2018**, *76*, 822-839, doi: 10.1093/nutrit/nuy031.
14. Khan, M.Y.; Mihali, A.B.; Rawala, M.S.; Aslam, A.; Siddiqui, W.J. The promising role of probiotic and synbiotic therapy in aminotransferase levels and inflammatory markers in patients with nonalcoholic fatty liver disease – a systematic review and meta-analysis. *Eur. J. Gastroen. Hepat.* **2019**, *31*, 703-715, doi: 10.1097/MEG.0000000000001371.
15. Skonieczna-żydecka, K.; Kaczmarczyk, M.; łoniewski, I.; Lara, L.; Koulaouzidis, A.; Misera, A.; Maciejewska, D.; Marlicz, W. A Systematic Review, Meta-Analysis, and Meta-Regression Evaluating the Efficacy and Mechanisms of Action of Probiotics and Synbiotics in the Prevention of Surgical Site Infections and Surgery-Related Complications. *Journal of Clinical Medicine* **2018**, *7*, 556, doi: 10.3390/jcm7120556.
16. Milajerdi, A.; Mousavi, S.M.; Sadeghi, A.; Salari-Moghaddam, A.; Parohan, M.; Larijani, B.; Esmaillzadeh, A. The effect of probiotics on inflammatory biomarkers: a meta-analysis of randomized clinical trials. *Eur. J. Nutr.* **2020**, *59*, 633-649, doi: 10.1007/s00394-019-01931-8.
17. Pan, H.; Li, R.; Li, T.; Wang, J.; Liu, L. Whether Probiotic Supplementation Benefits Rheumatoid Arthritis Patients: A Systematic Review and Meta-Analysis. *Engineering* **2017**, *3*, 115-121, doi: 10.1016/J.ENG.2017.01.006.
18. Fu, Y.; Chu, Q.; Ashuro, A.A.; Di, D.; Zhang, Q.; Liu, X.; Fan, Y. The Effect of Probiotics, Prebiotics, and Synbiotics on CD4 Counts in HIV-Infected Patients: A Systematic Review and Meta-Analysis. *Biomed Res. Int.* **2020**, *2020*, 1-11, doi: 10.1155/2020/7947342.
19. Zhang, X.; Chen, M.; Geng, S.; Yu, J.; Kuang, Y.; Luo, H.; Wang, K. Effects of Probiotics on Diarrhea and CD4 Cell Count in People Living With HIV: A Systematic Review and Meta-Analysis. *Frontiers in Pharmacology* **2021**, *12*, 570520, doi: 10.3389/fphar.2021.570520.
20. Tavakoly, R.; Hadi, A.; Rafie, N.; Talaei, B.; Marx, W.; Arab, A. Effect of Probiotic Consumption on Immune Response in Athletes: A Meta-analysis. *Int. J. Sports Med.* **2021**, *42*, 769-781, doi: 10.1055/a-1463-3303.

21. Askari, G.; Ghavami, A.; Shahdadian, F.; Moravejolahkami, A.R. Effect of synbiotics and probiotics supplementation on autoimmune diseases: A systematic review and meta-analysis of clinical trials. *Clin. Nutr.* **2021**, *40*, 3221-3234, doi: 10.1016/j.clnu.2021.02.015.
22. Jia, L.; Jia, Q.; Yang, J.; Jia, R.; Zhang, H. Efficacy of Probiotics Supplementation On Chronic Kidney Disease: a Systematic Review and Meta-Analysis. *Kidney and Blood Pressure Research* **2018**, *43*, 1623-1635, doi: 10.1159/000494677.
23. Tao, S.B.; Tao, S.Y.; Cheng, Y.M.; Liu, J.; Ma, L.; Fu, P. Effects of probiotic supplements on the progression of chronic kidney disease: A meta-analysis. *Nephrology* **2019**, *24*, 1122-1130, doi: 10.1111/nep.13549.
24. Thongprayoon, C.; Kaewput, W.; Hatch, S.T.; Bathini, T.; Sharma, K.; Wijarnpreecha, K.; Ungprasert, P.; D Costa, M.; Mao, M.A.; Cheungpasitporn, W. Effects of Probiotics on Inflammation and Uremic Toxins Among Patients on Dialysis: A Systematic Review and Meta-Analysis. *Digest. Dis. Sci.* **2019**, *64*, 469-479, doi: 10.1007/s10620-018-5243-9.
25. Bakhtiary, M.; Morvaridzadeh, M.; Agah, S.; Rahimlou, M.; Christopher, E.; Zadro, J.R.; Heshmati, J. Effect of Probiotic, Prebiotic, and Synbiotic Supplementation on Cardiometabolic and Oxidative Stress Parameters in Patients With Chronic Kidney Disease: A Systematic Review and Meta-analysis. *Clin. Ther.* **2021**, *43*, e71-e96, doi: 10.1016/j.clinthera.2020.12.021.
26. Mcfarlane, C.; Ramos, C.I.; Johnson, D.W.; Campbell, K.L. Prebiotic, Probiotic, and Synbiotic Supplementation in Chronic Kidney Disease: A Systematic Review and Meta-analysis. *J. Renal Nutr.* **2019**, *29*, 209-220, doi: 10.1053/j.jrn.2018.08.008.
27. Bock, P.M.; Telo, G.H.; Ramalho, R.; Sbaraini, M.; Leivas, G.; Martins, A.F.; Schaan, B.D. The effect of probiotics, prebiotics or synbiotics on metabolic outcomes in individuals with diabetes: a systematic review and meta-analysis. *Diabetologia* **2021**, *64*, 26-41, doi: 10.1007/s00125-020-05295-1.
28. Zheng, H.J.; Guo, J.; Jia, Q.; Huang, Y.S.; Huang, W.; Zhang, W.; Zhang, F.; Liu, W.J.; Wang, Y. The effect of probiotic and synbiotic supplementation on biomarkers of inflammation and oxidative stress in diabetic patients: A systematic review and meta-analysis of randomized controlled trials. *Pharmacol. Res.* **2019**, *142*, 303-313, doi: 10.1016/j.phrs.2019.02.016.
29. Tao, Y.; Gu, Y.; Mao, X.; Zhang, L.; Pei, Y. Effects of probiotics on type II diabetes mellitus: a meta-analysis. *J. Transl. Med.* **2020**, *18*, 30, doi: 10.1186/s12967-020-02213-2.
30. Chi, C.; Li, C.; Wu, D.; Buys, N.; Wang, W.; Fan, H.; Sun, J. Effects of Probiotics on Patients with Hypertension: a Systematic Review and Meta-Analysis. *Curr. Hypertens. Rep.* **2020**, *22*, doi: 10.1007/s11906-020-01042-4.
31. Hadi, A.; Arab, A.; Khalesi, S.; Rafie, N.; Kafeshani, M.; Kazemi, M. Effects of probiotic supplementation on anthropometric and metabolic characteristics in adults with metabolic syndrome: A systematic review and meta-analysis of randomized clinical trials. *Clin. Nutr.* **2021**, *40*, 4662-4673, doi: 10.1016/j.clnu.2021.05.027.
32. As Habi, A.; Nazari, M.; Hajianfar, H.; Arab, A.; Faghfoori, Z. A systematic review and meta-analysis of probiotic consumption and metabolic status of athletes. *Int. J. Food Prop.* **2020**, *23*, 941-954, doi: 10.1080/10942912.2020.1772286.
33. Malmir, H.; Ejtahed, H.; Soroush, A.; Mortazavian, A.M.; Fahimfar, N.; Ostovar, A.; Esmaillzadeh, A.; Larijani, B.; Hasani-Ranjbar, S. Probiotics as a New Regulator for Bone Health: A Systematic Review and Meta-Analysis. *Evid.-Based Compl. Alt.* **2021**, *2021*, 1-35, doi: 10.1155/2021/3582989.
34. Mcloughlin, R.F.; Berthon, B.S.; Jensen, M.E.; Baines, K.J.; Wood, L.G. Short-chain fatty acids, prebiotics, synbiotics, and systemic inflammation: a systematic review and meta-analysis. *The American Journal of Clinical Nutrition* **2017**, *106*, n156265, doi: 10.3945/ajcn.117.156265.
35. Da Silva Borges, D.; Fernandes, R.; Thives Mello, A.; Da Silva Fontoura, E.; Soares Dos Santos, A.R.; Santos De Moraes Trindade, E.B. Prebiotics may reduce serum concentrations of C-reactive protein and ghrelin in overweight and obese adults: a systematic review and meta-analysis. *Nutr. Rev.* **2019**, *78*, 235-248, doi: 10.1093/nutrit/nuz045.
36. Qu, H.; Zhang, Y.; Chai, H.; Gao, Z.; Shi, D. Effects of microbiota-driven therapy on inflammatory responses in elderly individuals: A systematic review and meta-analysis. *PLoS One* **2019**, *14*, e211233, doi: 10.1371/journal.pone.0211233.
37. Zhu, H.; Ren, Z.; Zang, Y.; Hua, H.; Lu, J.; Xu, Q.; Zhu, S. Effects of Microecological Preparations on Obese Patients after Bariatric Surgery: A Systematic Review and Meta-Analysis. *Evid.-Based Compl. Alt.* **2020**, *2020*, 1-10, doi: 10.1155/2020/8724546.
