# Supplementary material for: Gastrointestinal Microbiome and Multiple Health Outcomes: Umbrella Review
Source: Nutrients. 2022 Sep 9;14(18):3726. doi: 10.3390/nu14183726 (PMC9505003; doi:10.3390/nu14183726)
Supplement: Supplementary file 1 [file nutrients-14-03726-s001.zip › Supplementary Table S4.pdf]

Supplement Table S4. Prebiotics/probiotics/synbiotics and immune and gastrointestinal disease.

outcome(s) of interest	estimated summary effect (95% CI)	number of studies / total studies	number of intervention group or total participants	Heterogeneity (I^2, %)	the first author + year of publication	Intervention	Duration of Intervention/follow-up	study design	populations	outcome comparison	meta-analysis metric	type of effect model	publication bias
Incidence of C. difficile-associated diarrhea	0.4 (0.3 to 0.52)	31	4525/8672	0.0	GOLDENBERG J Z 2017[1]	probiotics	NP	All RCTs	participants taking antibiotics	any versus none	RR	random	No serious bias
Antibiotic-associated diarrhea	0.58 (0.48 to 0.70)	33	8870	61.0									
the occurrence of diarrhea and positive stool C . difficile cytotoxin assay or culture	0.38 (0.22 to 0.67)	6	597/1194	39.0	WU Z J 2013[2]	Lactobacillus	NP	All RCTs	With or without antibiotics	any versus none	RR	fixed	NA
	0.63(0.31 to 1.28)*	4	431/861	0.0		Lactobacillus acidophilus							
incidence of CDI (diarrhea and either positive stool cytotoxin, culture, or polymerase chain reaction testing for C difficile)	0.42(0.3 to 0.57)	19	6261	0.0	SHEN N T 2017[3]	probiotics	NP	All RCTs	Hospitalized adults (18 years or older) receiving antibiotic therapy (intravenous, oral, or both) for any reason	any versus none	RR	random	No serious bias
	0.32 (0.22 to 0.48)	14	NP	0.0		Timing: study protocol specifying maximum interval from starting antibiotics to starting probiotics, 1-2 d							NP
	0.7 (0.4 to 1.23)*	5	NP	0.0		Timing: study protocol specifying maximum interval from starting antibiotics to starting probiotics, 3-7 d							NP
	0.73 (0.29 to 1.88)*	3	NP	0.0		L rhamnosus GG							NP
	0.63 (0.29 to 1.37)*	5	NP	0.0		S boulardii							NP

Incidence of Clostridium difficile–associated diarrhea(CDAD)	0.34(0.24 to 0.49)	20	40/1974	0.0	JOHNSTON B C 2012[4]	any dose of a specified probiotic of any strain	NP	All RCTs	adult or pediatric patients treated with antibiotics	any versus none	RR	random	No serious bias
clinical remission	2.40(1.49 to 3.88)	3	71/162	29.0	DANG X 2020[5]	probiotic VSL#3 as induction therapy	6-12weeks	All RCTs	patients with active UC	any versus none	OR	Fixed	1.34
clinical response	3.09(1.53 to 6.25)	3	89/162	46.0								Random	0.47
Clinical remission	1.73(1.19 to 2.54)	9	594	55.0	KAUR L 2020[6]	Probiotics	NP	All RCTs	People of any age with active ulcerative colitis previously diagnosed by clinical, endoscopic, histologic or surgical remissionas defined by study authors	any versus placebo	RR	Random	NP
	2.02(1.31 to 3.12)	5	374	32.0						probiotics with multiple strains versus placebo			
	1.44(0.79 to 2.63)	4	220	57.0						probiotics with single strain versus placebo			
improvement in colon endoscopic scores	-0.58(-1.11 to -0.05)	3	93	85.0	RUFINO M N 2021[7]	Synbiotic treatment	4weeks to 1 year	All RCTs	Human patients with ulcerative colitis	conventional plus a synbiotic or synbiotic treatment alone versus conventional plus placebo or placebo treatment alone	RR	Fixed	NP
remission rate	1.40(1.27 to 1.53)	27	1942	0.0	PENG L J 2019 [8]	probiotics in conjunction with mesalazine or sulfasalazine or aminosalicylic acid	4-12 weeks	All RCTs	children or adults with UC regardless of clinical setting	probiotics in conjunction with mesalazine or sulfasalazine or aminosalicylic acid versus	RR	Fixed	0.000
	1.33(1.16 to 1.54)	11	NP	33.4					children or adults with UC in both				

									mild to moderate stages	mesalazine or sulfasalazine or aminosalyicylic acid alone			
	1.40(1.27 to 1.64)	16	NP	0.0					children or adults with UC in active stage				
	1.36(1.20 to 1.53)	17	NP	19.5					children or adults with UC regardless of clinical setting	probiotics combined with aminosalicylic acid on UC in 3 types of protiotics			
	1.45(1.24 to 1.70)	8	NP	0.0						probiotics combined with aminosalicylic acid on UC in 2 types of protiotics			
	1.40(1.27 to 1.64)	2	NP	0.0						probiotics combined with aminosalicylic acid on UC in 1 types of protiotics			
the efficacy of EcN in inducing remission for ulcerative colitis	0.92(0.15 to 5.66)*	3	267	86.0	LOSURDO G 2015[9]	Escherichia coli Nissle (EcN) 1917	NP	All RCTs	Patients with UC	EcN versus standard of care (mesalazine or ciprofloxacin)	OR	Fixed	No serious bias
										Regimen or placebo			
Maintenance of remission	1.07(0.70 to 1.64)*	4	442	0.0						EcN versus standard of care (mesalazine)			

Clinical relapse	0.87(0.63 to 1.18)*	4	361	17.0	IHEOZOR-EJIOFOR Z 2020[10]	Probiotics administered in any form (drink, powder, capsule), orally as a single species, or as a cocktail of multiple species	12-52 weeks	All RCTs	People of any age with ulcerative colitis in remission	Probiotics versus placebo	RR	Random	NP
Maintenance of clinical remission	1.16(0.98 to 1.37)*	2	141	0.0									
Clinical relapse	1.01(0.84 to 1.22)*	2	452	0.0						Probiotics versus 5-aminosalicylic acid (5-ASA) (mesalazine)			
Maintenance of clinical remission	1.06(0.90 to 1.25)*	1	125	NP									
Clinical relapse	1.11(0.66 to 1.84)*	2	242	14.7						Probiotic + 5-ASA (mesalazine) versus 5-ASA (mesalazine)			
Maintenance of clinical remission	1.05(0.89 to 1.24)*	1	122	NP									
remission in active UC patients, assessed with the Ulcerative Colitis Disease Activity Index (UCDAI) and Disease Activity Index (DAI)/Mayo scores	1.55(1.13 to 2.15)	6	503	29.0	ASTO E 2019[11]	probiotic, prebiotic, inulin, fructo-oligosaccharides, FOS, and synbiotic	NP	All RCTs	adults or children with active UC	administration (oral or rectal) of probiotics versus placebo or active treatment of UC to induce remission or to maintain remission	RR	random	NP
Efficacy of inducing/maintaining remission	1.73(1.23 to 2.43)	6	424	35.0		bifidobacteria-containing probiotics							
	1.04(0.35 to 3.06)*	3	168	78.0		Probiotics without bifidobacteria							
	1.99(1.25 to 3.15)	4	348	49.0		VSL#3							
	1.38(0.86 to 2.21)*	4	313	56.0		probiotic, prebiotic, inulin, fructo-oligosaccharides, FOS, and synbiotic			adults or children with inactive UC				
Efficacy of Probiotics at Maintaining	0.92(0.82 to	3	513	0.0		the probiotic Mutaflor			adults or children with active	the probiotic Mutaflor with			

Remission	1.04)*								UC or inactive UC	mesalazine			
the incidence of abdorminal distension	0.27(0.17 to 0.43)	11	493/981	0.0	CHEN X R 2021[12]	early enteral nutrition (EEN) combined with probiotics	≥ 14 days	All RCTs	All patients met the criteria of severe stroke	early enteral nutrition (EEN) combined with probiotics versus EEN	OR	Random	No serious bias
the incidence of diarrhea	0.20(0.12 to 0.32)	14	604/1205	0.0									
the incidence of vomiting	0.20(0.12 to 0.32)	8	301/602	0.0									
the incidence of gastric retention	0.25(0.11 to 0.55)	3	148/296	0.0									
the incidence of constipation	0.20(0.12 to 0.32)	14	590/1177	0.0									
the incidence of reflux	0.47(0.21 to 1.02)*	7	316/632	19.0									
the incidence of digestive tract hemorrhage	0.31(0.18 to 0.52)	12	537/1072	0.0									
the incidence of stress ulcer	0.64(0.21 to 1.92)*	3	118/236	0.0									
the incidence of gastrointestinal complications	0.29(0.24 to 0.36)	17	3169/6352	0.0									
incidence of antibiotic-associated diarrhoea	0.63 (0.54 to 0.73)	42	5985/11305	60.0	GOODMAN C 2021 [13]	probiotic	NP	All RCTs	adult population to whom antibiotics were administered	probiotics versus any control	RR	Random	No serious bias
	0.54 (0.38 to 0.76)	4	324/651	0.0						High dose vs low dose			
incidence of antibiotic-associated diarrhoea	0.45 (0.36 to 0.56)	33	6352	57.0	GUO Q 2019 [14]	specific, identified probiotic in any form (e.g. capsule, sachet, yogurt). Trials investigating non-specific probiotic or	5 days to 12 weeks	All RCTs	children (0 to 18 years) receiving antibiotics	Probiotic Versus Control (placebo or non-active control)	RR	Random	No serious bias

	0.37 (0.30 to 0.46)	20	4038	NP		yogurt agents (e.g. products that do not label the probiotic strain and dose) were not considered				Probiotic dose (≥5 billion CFUs of probiotics/day) Versus Control			
Duration of diarrhea (days)	-0.91 (-1.38 to -0.44)	8	1263	84.0			10 days to 12 weeks			Probiotic Versus Control	MD		
incidence of antibiotic-associated diarrhoea	0.58 (0.48 to 0.71)	30	3805/7023	58.0	JAFARNEJAD S 2016[15]	oral probiotic intake (in either supplements or foods)	NP	All RCTs	individuals aged >18	Probiotic Versus Control	RR	random	No serious bias
	0.47 (0.40 to 0.56)	25	20873826	43.0					adults (18–64 years)				
	0.94 (0.76 to 1.15)*	5	1718/3434	65.0					the elderly (>65 years)				
the incidence of AAD	0.49 (0.36 to 0.66)	17	1832/3611	58.0	BLAABJERG S 2017[16]	Probiotics	NA	All RCTs	outpatients taking oral antibiotics	probiotics vs. Placebo or no treatment	RR	random	No serious bias
	0.49 (0.36 to 0.66)	4	590/1139	0.0		S. boulardii							NP
Incidence of Antibiotic-Associated Diarrhea Using the Criteria Defined by WHO	0.54 (0.36 to 0.82)	7	876/1724	27.0		Probiotics							NP
the incidence of AAD	0.42 (-0.23 to 0.77)	7	726/1446	76.0					outpatients taking oral antibiotics, children (<15 years of age)		RD (risk difference)		
	0.42 (-0.23 to 0.77)	9	975/1936	47.0					outpatients taking oral antibiotics, adults (>15 years of age)				

inducing/maintaining IBD remission	1.13 (1.02 to 1.26)	21	918/1816	55.1	ZHANG X 2021 [17]	probiotics, prebiotics or synbiotics	NP	All RCTs	Patient with Inflammatory bowel disease (IBD)	probiotics, prebiotics and synbiotics vs. Control group	RR	random	0.336
UC disease activity index	1.00 (0.27 to 1.73)	10	1026	0.0							SMD		0.463
CD disease activity index	-0.33 (-1.05 to 0.38)*	4	206	79.9							SMD		NP
IBD recurrence	0.48 (0.65 to 1.08)*	13	1372	52.7							RR		0.034
preventing relapse in active UC	1.02 (0.85 to 1.23)*	3	277/555	0.0	DERWA Y 2017 [18]	probiotics	NP	All RCTs	Adults (>90% of patients aged >16 years) with inflammatory bowel disease	probiotics vs. placebo	RR	random	NA
preventing relapse in CD in remission following a surgical resection	1.06 (0.59 to 1.92)*	4	105/213	37.0						Probiotics vs. 5-ASA			
prevention of endoscopic relapse of disease activity (Rutgeerts score 1 or more)	1.09 (0.92 to 1.29)*	4	163/233	53.0						probiotics vs. placebo			
Endoscopic relapse of disease activity (Rutgeerts score 2 or more)	1.04 (0.82 to 1.31)*	4	163/233	32.0									
Endoscopic relapse of disease activity (Rutgeerts score 3 or more)	1.13 (0.84 to 1.52)*	4	163/233	0.0									
Efficacy of probiotics in inducing remission in active UC	0.81 (0.72 to 0.91)	9	362/759	32.0	PABON-CARRASCO M 2020 [19]	probiotics	0.5-12 months	All RCTs	Adult with inflammatory bowel disease	probiotics vs. placebo	RR	fixed	No serious bias
Efficacy of probiotics in preventing relapse in quiescent UC	0.89 (0.68 to 1.15)*	3	147/284	0.0									
Efficacy of the Probiotics for the Remission of	0.90 (0.77 to	5	253/494	37.0									

Crohn's Disease	1.06)*												
effect on persistence of symptoms	0.79 (0.68 to 0.91)	21	1931	72.0	FORD A C 2018[20]	Combination probiotics	Minimum treatment duration of 7 days	All RCTs	Adults (participants aged >16 years)	probiotics vs. Control group	RR	random	0.06
	0.82 (0.63 to 1.06)*	8	893	83.0		Lactobacillus	Minimum follow-up duration of 7 days						NP
	0.67 (0.51 to 0.87)	3	314	63.0		Only Lactobacil- lus plantarum DSM 9843							
	0.70 (0.48 to 1.01)*	3	528	72.0		Bifidobacterium							
effect on global symptom or abdominal pain scores	-0.31 (-0.44 to -0.17)	19	1341	24.0		Combination probiotics					SMD		0.06
	-0.09 (-0.25 to 0.036)	9	989	25.0		Lactobacillus							NP
	0.12 (-0.27 to 0.50)*	4	388	70.0		Saccharomyces							
	-0.46 (-0.92 to 0.00)	3	501	77.0		Bifidobacterium							
bloating scores	-0.15 (-0.31 to 0.01)*	17	1155	36.0		Combination probiotics							
	-0.30 (-0.68 to 0.09)*	3	501	68.0		Bifidobacterium							
	-0.01 (-0.36 to 0.34)*	3	209	40.0		Saccharomyces							
flatulence symptom scores	-0.29 (-0.51 to -0.07)	8	318	0.0		Combination probiotics							
global symptom scores	-0.23 (-0.44 to -0.02)	17	2401	72.0	ASHA M Z 2020[21]	Probiotics or prebiotics	2-24 weeks	All RCTs	adult IBS patients (aged ≥18	Probiotics or prebiotics vs. Control	SMD	random	NP

abdominal pain scores	-0.18 (-0.43 to 0.07)*	29	3688	92.0		Probiotics or prebiotics or synbiotics			years)	group			
	-0.71 (-1.33 to -0.10)	5	660	NP		probiotics containing Lactobacillus species							
Abdominal pain score	-1.15 (-2.05 to -0.24)	7	508	95.0	XU H L 2021 [22]	probiotics	4-8 weeks	All RCTs	Patients aged between 4 and 18 years with IBS met the diagnostic criteria (Rome II~IV)	probiotics vs. Control group	SMD	rando m	NP
Abdominal pain treatment success	3.44 (1.73 to 6.87)	3	163	0.0							RR		
Abdominal pain relief	1.48 (0.96 to 2.28)*	3	167	40.0							RR		
Frequency of abdominal pain	-0.82 (-1.57 to -0.07)	3	147	2.0							MD		
Standard abdominal pain	-0.15 (-0.27 to -0.04)	6	508	94.0							MD		
Abdominal pain	0.03 (-0.22 to 0.29)*	5	243	0.0	CONNELL M 2018[23]	VSL#3	4-8 weeks	All RCTs	IBS patient	VSL#3 vs. placebo	SMD	fixed	NP
Stool consistency	-0.00 (-0.09 to 0.08)*	3	177	0.0							MD		
Overall response	1.39 (0.98 to 1.96) *	3	177	0.0							RR		
Abdominal bloating	0.15 (-0.11 to 0.40)*	5	243	5.0							SMD		
Quality of Life	-0.08 (-0.39 to 0.22)*	3	170	0.0							SMD		
Abdominal pain	0.25 (-0.27 to 0.77)*	3	554	≥ 50%	YUAN F Q 2017[24]	B. Infantis only	4-8 weeks	All RCTs	patients with IBS, age greater than 15 years old	Probiotics Including B. Infantis versus placebo	SMD	rando m	NP
	0.23 (-0.03 to	5	666	≥ 50%		B. Infantis only or composite probiotic						rando	

	0.49)*					Including B. Infantis						m	
Bloating/distention	0.20 (-0.19 to 0.58)*	3	554	≥ 50%		B. Infantis only						Fixed	
	0.21 (0.07 to 0.35)	5	666	≤ 50%		B. Infantis only or composite probiotic Including B. Infantis						fixed	
Bowel habit satisfaction	0.66 (-0.11 to 1.43)*	3	554	≥ 50%		B. Infantis only						rando m	
stool frequency	1.29 (0.69 to 1.89)*	10	1039	90.0	WEN Y 2020 [25]	Any species/strains/dose/treatment regimen of live probiotics	2-12 weeks	All RCTs	Adult populations aged ≥16 y with functional chronic constipation defined by clinical symptoms, a physician's opinion, or the Rome I, II, or III criteria	probiotics vs. placebo	MD	rando m	0.183
Effect of probiotics on stool consistency for IBS-C	0.55 (0.27 to 0.82)	9	NP	80.0							SMD		0.583
	0.46 (0.08 to 0.85)	4	NP	NP		B. lactis							NP
Satisfaction in relief	0.95 (0.77 to 1.18)*	3	315			Any species/strains/dose/treatment regimen of live probiotics					RR	fixed	
Bloating	-0.77 (-1.46 to -0.07)	4	460								SMD	rando m	
Flatulence	-0.34 (-0.70 to 0.02)*	3	158									fixed	
Incomplete evacuation	-0.77 (-1.14 to -0.39)	6	502									rando m	
Hard stools	-0.74 (-1.19 to -0.28)	5	280										
Ease of expulsion	0.81 (0.15 to 1.48)	4	392										
incidence of diarrhea	0.51 (0.37 to 0.70)	4	404	0.0	WU X D 2018 [26]	prophylactic probiotics in combination with antibiotics preoperatively	Preoperation 1-14 days, postoperation 0-14 days	All RCTs	adult patients who	Vs. placebo or other base ingredients	RR	rando m	No serious bias

abdominal distention	0.63 (0.48 to 0.83)	5	394	0.0							undergo colorectal surgery	in combination with antibiotics preoperatively			
stool frequency	-0.09 (-0.47 to 0.29)*	4	NP	0.0	ZHONG 2017[27]	C	Q	Probiotic	NP	RCTs, crossover trials, prospective single-arm trials and retrospective studies	Human	Probiotics vs. No probiotics	WMD	random	NP
AIDS-related diarrhea	0.60 (0.44 to 0.82)	6	360	0.0	ZHANG 2021[28]	X	L	probiotic	2-48 weeks	RCTs, clinical cohort studies, pilot study with controls, or other clinical studies	HIV/AIDS patients and patients diagnosed with similar recognized criteria based on laboratory evidence or methods of HIV infection	Probiotic vs. No probiotic	MD	fixed	No serious bias
Stool frequency	0.98 (0.39 to 1.56)	15	1189	87.0	ZHANG 2020[29]		C	Probiotics administered in the form of any formulation or dairy product	2-16 weeks	All RCTs	adults	probiotics vs placebo	MD	random	0.32
Gut transit time	-13.75 (-21.93 to -5.56)	5	325	69.0											0.81
Stool consistency	1.30 (0.22 to 2.38)	6	386	96.0											0.21
Bloating	-0.24 (-0.55 to 0.07)*	4	282	27.0											0.77
stool frequency	0.73 (0.14 to 1.31)	6	444	84.0	HUANG 2017[30]		R	Any type of culture/strain/dose/therapy regimen of probiotics (include synbiotics)	3-12 weeks	All RCTs	children aged ≤ 18 years with constipation identified by relative	probiotics vs control group	MD	random	No serious bias
stool consistency	-0.07 (-0.21 to	3	267	92.0											

	0.06)*								clinical symptoms, pediatric physicians, or the Rome I, II, or III criteria				
eradication of H. pylori	7.91 (2.97 to 21.05)	6	352	0.0	LOSURDO G 2018[31]	probiotic supplementation	NP	All RCTs	H. pylori-infected patients	Vs. placebo	OR	fixed	0.02
oropharyngeal colonization	0.87 (0.76 to 0.99)	6	915	0.0	JI T 2020[32]	probiotic	NP	All RCTs	adults who received mechanical ventilation (MV) therapy in hospitals	Vs. control	RR	fixed	NP
gastric colonization	0.65 (0.49 to 0.84)	4	519	30.2								fixed	
rate of gram-negative (GN) bacteria positivity	0.58 (0.58 to 0.86)	9	1247	60.0								random	
positive rate of gram-positive bacteria culture	0.95 (0.67 to 1.35)*	8	1175	31.3								fixed	
positive fungal culture rates	0.98 (0.67 to 1.43)*	7	886	26.5								fixed	
the incidence of diarrhea	0.69 (0.47 to 1.02)*	8	1219	59.4								random	
fecal zonulin	-0.63 (-1.17 to -0.08)	3	NP	79.4	AS'HABI A 2020 [33]	probiotics (supplement or food)	2-20 weeks	All RCTs	athletes	Vs. control	SMD	random	0.19
Stool pH	-0.65 (-0.76 to -0.54)	6	470	81.0	RAO S 2009[34]	combination of prebiotics and probiotics vs controls	NP	All RCTs	full-term neonates	formula had different composition vs. the control formula (apart from prebiotics)	WMD	fixed	No serious bias
Diarrheal episodes	0.71 (0.35 to 1.43)*	3	606	99.0	LOHNER S 2014[35]	prebiotics (prebiotics added to food in the manufacturing process or as a separate	a supplementation time of at least 2 months and an observation time of	All RCTs	healthy infants or children, aged 0–18	Vs. control (placebo or no	RR	random	NP

						supplement)	at least 4 months		years	supplemen-tation)		
overall symptom response to treatment	0.62 (0.07 to 5.69)*	3	191	91.0	WILSON B 2019[36]	prebiotics	2-12 weeks	All RCTs	Adult patients ≥18 and ≤64 y with Irritable bowel syndrome (IBS) or other Functional bowel disorders (FBDs)	vs. placebo	OR	random	No serious bias
integrative symptom score	-0.39 (-1.43 to 0.64)*	8	NP	97.0							SMD		
severity of abdominal pain	-0.83 (-1.84 to 0.18)*	10		97.0									
bloating	-0.57 (-1.67 to 0.52)*	9		97.0									
flatulence	-0.53 (-2.04 to 0.98)*	6		98.0									
necrotizing enterocolitis	0.79 (0.14 to 1.44)*	6	737	21.0	CHI C 2019[37]	prebiotics	7-57 days	All RCTs	low birth weight infants (<2500 g) or preterm infants (<36 weeks)	vs. placebo	RR	random	0.73
feeding intolerance	0.87 (0.52 to 1.45)*	4	413	0.0									NA
full enteral feeding	-0.99 (-1.15 to -0.83)	7	576	0.0									0.63
stool frequency	0.52 (0.30 to 0.73)	6	294	13.0									0.32
Clinical remission	1.21 (1.18 to 1.24)	45	1816/3624	NP	Sohail G 2018[38]	probiotic Medilac-S	4-96 weeks	All RCTs	human	Vs. control	RR	fixed	No serious bias
Endoscopy scores	0.71 (0.35 to 1.07)	7	270								MD	random	
Histological scores	1.07 (0.92 to 1.23)	8	501								MD	fixed	
incidence of Hirschsprung-associ	0.72 (0.37 to	5	93/198	37.0	NAKAMURA H 2018 [39]	probiotics	NP	NP	premature infants	Probiotics vs. control	OR	fixed	NP

ated entero-colitis (HAEC)	1.39)*												
intestinal transit time (ITT)	0.38 (0.23 to 0.53)	17	NP	20.0	MILLER L E 2016[40]	Probiotic or synbiotic	10-56 days	All RCTs	adult	probiotics vs. control	SMD	random	0.44
gastrointestinal symptoms (any questionnaire), continuous outcome	-0.48 (-1.21 to 0.25)*	4	197	81.0	SEILER C L 2020[41]	probiotics of any type and dose, administered orally in any form (drink/powder/capsule)	at least 2 weeks	All RCTs	patients With Celiac disease (CD)	probiotics vs placebo	MD	random	NP
Change in bifidobacteria species (log colony-forming units per gram [CFU/g]) from baseline to end of study, continuous outcome	0.85 (0.38 to 1.32)	4	224	62.0									
Change in lactobacilli species (log CFU/g) from baseline to end of study, continuous outcome	1.13 (-0.67 to 2.93)*	4	224	96.0									
functional dyspepsia (FD) symptom improvement	1.15 (1.01 to 1.30)*	5	409	0.0	ZHANG J 2020[42]	probiotic or prebiotic	≥ 7 days	All RCTs	adult patients (over the age of 16 years) with functional dyspepsia (FD)	Vs. placebo	RR	fixed	NP
percentages of Bifidobacterium species	31.75 (26.04 to 37.45)	3	NP	49.3	SORENSEN K 2021[43]	any amino acid formula (AAF) with synbiotics (AAF-Syn) or extensively hydrolysed formula (eHF) with synbiotics (eHF-Syn), Studies of infant formulae containing cow's milk protein, and eHF or AAF without synbiotics (including those with only a prebiotic or a probiotic) were excluded	7 days to 12 months	All RCTs	infants and children aged <3 years with IgE or non IgE mediated Cow's milk protein allergy (CMPA)	AAF-Syn than with AAF alone	MD	fixed	NP
percentages of Eubacterium rectale and Clostridium coccoides	-19.06 (-23.15 to -14.97)	3		18.1									

* No statistical significance; CI, confidence interval; RCT, randomized controlled trial; RR, relative risk; HR, hazard ratio; MD, mean difference; SMD, standard mean difference; WMD, weighted mean difference; OR, odds ratio; NA, not available; NP, not published.

References

1. Goldenberg, J.Z.; Yap, C.; Lytvyn, L.; Lo, C.K.; Beardsley, J.; Mertz, D.; Johnston, B.C. Probiotics for the prevention of Clostridium difficile-associated diarrhea in adults and children. *Cochrane Db. Syst. Rev.* **2017**, *2017*, D6095, doi: 10.1002/14651858.CD006095.pub4.
2. Wu, Z.J.; DU X; Zheng, J. Role of Lactobacillus in the prevention of Clostridium difficile-associated diarrhea: a meta-analysis of randomized controlled trials. *Chin Med J (Engl)* **2013**, *126*, 4154-4161.
3. Shen, N.T.; Maw, A.; Tmanova, L.L.; Pino, A.; Ancy, K.; Crawford, C.V.; Simon, M.S.; Evans, A.T. Timely Use of Probiotics in Hospitalized Adults Prevents Clostridium difficile Infection: A Systematic Review With Meta-Regression Analysis. *Gastroenterology* **2017**, *152*, 1889-1900, doi: 10.1053/j.gastro.2017.02.003.
4. Johnston, B.C.; Ma, S.S.Y.; Goldenberg, J.Z.; Thorlund, K.; Vandvik, P.O.; Loeb, M.; Guyatt, G.H. Probiotics for the Prevention ofClostridium difficile – Associated Diarrhea. *Ann. Intern. Med.* **2012**, *157*, 878, doi: 10.7326/0003-4819-157-12-201212180-00563.
5. Dang, X.; Xu, M.; Liu, D.; Zhou, D.; Yang, W. Assessing the efficacy and safety of fecal microbiota transplantation and probiotic VSL#3 for active ulcerative colitis: A systematic review and meta-analysis. *PLoS One* **2020**, *15*, e228846, doi: 10.1371/journal.pone.0228846.
6. Kaur, L.; Gordon, M.; Baines, P.A.; Iheozor-Ejiofor, Z.; Sinopoulou, V.; Akobeng, A.K. Probiotics for induction of remission in ulcerative colitis. *Cochrane Db. Syst. Rev.* **2020**, *2020*, D5573, doi: 10.1002/14651858.CD005573.pub3.
7. Rufino, M.N.; Da Costa, A.L.; Jorge, E.N.; Paiano, V.F.; Camparoto, M.L.; Keller, R.; Bremer-Neto, H. Synbiotics improve clinical indicators of ulcerative colitis: systematic review with meta-analysis. *Nutr. Rev.* **2022**, *80*, 157-164, doi: 10.1093/nutrit/nuab017.
8. Peng, L.; Zhong, Y.; Wang, A.; Jiang, Z. Probiotics combined with aminosalicylic acid affiliates remission of ulcerative colitis: a meta-analysis of randomized controlled trial. *Bioscience Rep.* **2019**, *39*, doi: 10.1042/BSR20180943.
9. Losurdo, G.; Iannone, A.; Contaldo, A.; Ierardi, E.; Di Leo, A.; Principi, M. Escherichia coli Nissle 1917 in Ulcerative Colitis Treatment: Systematic Review and Meta-analysis. *J. Gastrointest. Liver* **2020**, *24*, 499-505, doi: 10.15403/jgld.2014.1121.244.ecn.
10. Iheozor-Ejiofor, Z.; Kaur, L.; Gordon, M.; Baines, P.A.; Sinopoulou, V.; Akobeng, A.K. Probiotics for maintenance of remission in ulcerative colitis. *Cochrane Db. Syst. Rev.* **2020**, *3*, D7443, doi: 10.1002/14651858.CD007443.pub3.
11. Astó, E.; Méndez, I.; Audivert, S.; Farran-Codina, A.; Espadaler, J. The Efficacy of Probiotics, Prebiotic Inulin-Type Fructans, and Synbiotics in Human Ulcerative Colitis: A Systematic Review and Meta-Analysis. *Nutrients* **2019**, *11*, 293, doi: 10.3390/nu11020293.
12. Chen, X.; Hu, Y.; Yuan, X.; Yang, J.; Ka, L. Effect of early enteral nutrition combined with probiotics in patients with stroke: a meta-analysis of randomized controlled trials. *Eur. J. Clin. Nutr.* **2022**, *76*, 592-603, doi: 10.1038/s41430-021-00986-3.
13. Goodman, C.; Keating, G.; Georgousopoulou, E.; Hespe, C.; Levett, K. Probiotics for the prevention of antibiotic-associated diarrhoea: a systematic review and meta-analysis. *BMJ Open* **2021**, *11*, e43054, doi: 10.1136/bmjopen-2020-043054.

14. Guo, Q.; Goldenberg, J.Z.; Humphrey, C.; Dib, R.E.; Johnston, B. PROBIOTICS FOR THE PREVENTION OF PEDIATRIC ANTIBIOTIC-ASSOCIATED DIARRHEA. *Gastroenterology* **2019**, *156*, 220, doi: 10.1016/S0016-5085%2819%2937348-2.
15. Jafarnejad, S.; Shab-Bidar, S.; Speakman, J.R.; Parastui, K.; Daneshi-Maskooni, M.; Djafarian, K. Probiotics Reduce the Risk of Antibiotic-Associated Diarrhea in Adults (18–64 Years) but Not the Elderly (>65 Years). *Nutr. Clin. Pract.* **2016**, *31*, 502-513, doi: 10.1177/0884533616639399.
16. Blaabjerg, S.; Artzi, D.; Aabenhus, R. Probiotics for the Prevention of Antibiotic-Associated Diarrhea in Outpatients—A Systematic Review and Meta-Analysis. *Antibiotics* **2017**, *6*, 21, doi: 10.3390/antibiotics6040021.
17. Zhang, X.; Guan, X.; Tang, Y.; Sun, J.; Wang, X.; Wang, W.; Fan, J. Clinical effects and gut microbiota changes of using probiotics, prebiotics or synbiotics in inflammatory bowel disease: a systematic review and meta-analysis. *Eur. J. Nutr.* **2021**, *60*, 2855-2875, doi: 10.1007/s00394-021-02503-5.
18. Derwa, Y.; Gracie, D.J.; Hamlin, P.J.; Ford, A.C. Systematic review with meta-analysis: the efficacy of probiotics in inflammatory bowel disease. *Aliment. Pharm. Ther.* **2017**, *46*, 389-400, doi: 10.1111/apt.14203.
19. Pabón-Carrasco, M.; Ramirez-Baena, L.; Vilar-Palomo, S.; Castro-Méndez, A.; Martos-García, R.; Rodríguez-Gallego, I. Probiotics as a Coadjuvant Factor in Active or Quiescent Inflammatory Bowel Disease of Adults—A Meta-Analytical Study. *Nutrients* **2020**, *12*, 2628, doi: 10.3390/nu12092628.
20. Ford, A.C.; Harris, L.A.; Lacy, B.E.; Quigley, E.M.M.; Moayyedi, P. Systematic review with meta-analysis: the efficacy of prebiotics, probiotics, synbiotics and antibiotics in irritable bowel syndrome. *Aliment. Pharm. Ther.* **2018**, *48*, 1044-1060, doi: 10.1111/apt.15001.
21. Asha, M.Z.; Khalil, S.F.H. Efficacy and Safety of Probiotics, Prebiotics and Synbiotics in the Treatment of Irritable Bowel Syndrome: A systematic review and meta-analysis. *Sultan Qaboos University Medical Journal [SQUMJ]* **2020**, *20*, 13-24, doi: 10.18295/squmj.2020.20.01.003.
22. Xu, H.; Zou, L.; Chen, M.; Wang, H.; Shen, W.; Zheng, Q.; Cui, W. Efficacy of probiotic adjuvant therapy for irritable bowel syndrome in children: A systematic review and meta-analysis. *PLoS One* **2021**, *16*, e255160, doi: 10.1371/journal.pone.0255160.
23. Connell, M.; Shin, A.; James-Stevenson, T.; Xu, H.; Imperiale, T.F.; Herron, J. Systematic review and meta-analysis: Efficacy of patented probiotic, VSL#3, in irritable bowel syndrome. *Neurogastroenterology & Motility* **2018**, *30*, e13427, doi: 10.1111/nmo.13427.
24. Yuan, F.; Ni, H.; Asche, C.V.; Kim, M.; Walayat, S.; Ren, J. Efficacy ofBifidobacterium infantis 35624 in patients with irritable bowel syndrome: a meta-analysis. *Curr. Med. Res. Opin.* **2017**, *33*, 1191-1197, doi: 10.1080/03007995.2017.1292230.
25. Wen, Y.; Li, J.; Long, Q.; Yue, C.; He, B.; Tang, X. The efficacy and safety of probiotics for patients with constipation-predominant irritable bowel syndrome: A systematic review and meta-analysis based on seventeen randomized controlled trials. *Int. J. Surg.* **2020**, *79*, 111-119, doi: 10.1016/j.ijsu.2020.04.063.
26. Wu, X.; Xu, W.; Liu, M.; Hu, K.; Sun, Y.; Yang, X.; Zhu, G.; Wang, Z.; Huang, W. Efficacy of prophylactic probiotics in combination with antibiotics versus antibiotics alone for colorectal surgery: A meta-analysis of randomized controlled trials. *J. Surg. Oncol.* **2018**, *117*, 1394-1404, doi: 10.1002/jso.25038.
27. Zhong, C.; Qu, C.; Wang, B.; Liang, S.; Zeng, B. Probiotics for Preventing and Treating Small Intestinal Bacterial Overgrowth. *J. Clin. Gastroenterol.* **2017**, *51*, 300-311, doi: 10.1097/MCG.0000000000000814.
28. Zhang, X.; Chen, M.; Geng, S.; Yu, J.; Kuang, Y.; Luo, H.; Wang, K. Effects of Probiotics on Diarrhea and CD4 Cell Count in People Living With HIV: A Systematic Review and Meta-Analysis. *Frontiers in Pharmacology* **2021**, *12*, 570520, doi: 10.3389/fphar.2021.570520.
29. Zhang, C.; Jiang, J.; Tian, F.; Zhao, J.; Zhang, H.; Zhai, Q.; Chen, W. Meta-analysis of randomized controlled trials of the effects of probiotics on functional constipation in adults. *Clin. Nutr.* **2020**, *39*, 2960-2969, doi: 10.1016/j.clnu.2020.01.005.

30. Huang, R.; Hu, J. Positive Effect of Probiotics on Constipation in Children: A Systematic Review and Meta-Analysis of Six Randomized Controlled Trials. *Front. Cell. Infect. Mi.* **2017**, *7*, 153, doi: 10.3389/fcimb.2017.00153.
31. Losurdo, G.; Cubisino, R.; Barone, M.; Principi, M.; Leandro, G.; Ierardi, E.; Leo, A.D. Probiotic monotherapy andHelicobacter pylori eradication: A systematic review with pooled-data analysis. *World J. Gastroentero.* **2018**, *24*, 139-149, doi: 10.3748/wjg.v24.i1.139.
32. Ji, T.; Zhu, X.; Shang, F.; Zhang, X. Preventive Effect of Probiotics on Ventilator-Associated Pneumonia: A Meta-analysis of 2428 Patients. *Ann. Pharmacother.* **2021**, *55*, 949-962, doi: 10.1177/1060028020983021.
33. As Habi, A.; Nazari, M.; Hajianfar, H.; Arab, A.; Faghfoori, Z. A systematic review and meta-analysis of probiotic consumption and metabolic status of athletes. *Int. J. Food Prop.* **2020**, *23*, 941-954, doi: 10.1080/10942912.2020.1772286.
34. Rao, S.; Srinivasjois, R.; Patole, S. Prebiotic Supplementation in Full-term Neonates. *Archives of Pediatrics & Adolescent Medicine* **2009**, *163*, 755-764, doi: 10.1001/archpediatrics.2009.94.
35. Lohner, S.; Küllenberg, D.; Antes, G.; Decsi, T.; Meerpohl, J.J. Prebiotics in healthy infants and children for prevention of acute infectious diseases: a systematic review and meta-analysis. *Nutr. Rev.* **2014**, *72*, 523-531, doi: 10.1111/nure.12117.
36. Wilson, B.; Rossi, M.; Dimidi, E.; Whelan, K. Prebiotics in irritable bowel syndrome and other functional bowel disorders in adults: a systematic review and meta-analysis of randomized controlled trials. *The American Journal of Clinical Nutrition* **2019**, *109*, 1098-1111, doi: 10.1093/ajcn/nqy376.
37. Chi, C.; Buys, N.; Li, C.; Sun, J.; Yin, C. Effects of prebiotics on sepsis, necrotizing enterocolitis, mortality, feeding intolerance, time to full enteral feeding, length of hospital stay, and stool frequency in preterm infants: a meta-analysis. *Eur. J. Clin. Nutr.* **2019**, *73*, 657-670, doi: 10.1038/s41430-018-0377-6.
38. Sohail, G.; Xu, X.; Christman, M.C.; Tompkins, T.A. Probiotic Medilac-S® for the induction of clinical remission in a Chinese population with ulcerative colitis: A systematic review and meta-analysis. *World Journal of Clinical Cases* **2018**, *6*, 961-984, doi: 10.12998/wjcc.v6.i15.961.
39. Nakamura, H.; Lim, T.; Puri, P. Probiotics for the prevention of Hirschsprung-associated enterocolitis: a systematic review and meta-analysis. *Pediatr. Surg. Int.* **2018**, *34*, 189-193, doi: 10.1007/s00383-017-4188-y.
40. Miller, L.E.; Zimmermann, A.K.; Ouwehand, A.C. Contemporary meta-analysis of short-term probiotic consumption on gastrointestinal transit. *World J. Gastroentero.* **2016**, *22*, 5122, doi: 10.3748/wjg.v22.i21.5122.
41. Seiler, C.L.; Kiflen, M.; Stefanolo, J.P.; Bai, J.C.; Bercik, P.; Kelly, C.P.; Verdu, E.F.; Moayyedi, P.; Pinto-Sanchez, M.I. Probiotics for Celiac Disease: A Systematic Review and Meta-Analysis of Randomized Controlled Trials. *Am. J. Gastroenterol.* **2020**, *115*, 1584-1595, doi: 10.14309/ajg.0000000000000749.
42. Zhang, J.; Wu, H.M.; Wang, X.; Xie, J.; Li, X.; Ma, J.; Wang, F.; Tang, X. Efficacy of prebiotics and probiotics for functional dyspepsia. *Medicine* **2020**, *99*, e19107, doi: 10.1097/MD.0000000000019107.
43. Sorensen, K.; Cawood, A.L.; Gibson, G.R.; Cooke, L.H.; Stratton, R.J. Amino Acid Formula Containing Synbiotics in Infants with Cow's Milk Protein Allergy: A Systematic Review and Meta-Analysis. *Nutrients* **2021**, *13*, 935, doi: 10.3390/nu13030935.
