# Supplementary material for: Gastrointestinal Microbiome and Multiple Health Outcomes: Umbrella Review
Source: Nutrients. 2022 Sep 9;14(18):3726. doi: 10.3390/nu14183726 (PMC9505003; doi:10.3390/nu14183726)
Supplement: Supplementary file 1 [file nutrients-14-03726-s001.zip › Supplementary Table S5.pdf]

Supplement Table S5. Prebiotics/probiotics/synbiotics neurological and psychiatric outcomes.

outcome(s) of interest	estimated summary effect (95% CI)	number of studies / total studies	number of intervention group or total participants	Heterogeneity (I^2, %)	the first author + year of publication	Intervention	Duration of Intervention/follow-up	study design	populations	outcome comparison	meta-analysis metric	type of effect model	publication bias
effect of probiotics on cognition	0.24 (0.05 to 0.42)	7	NP	0.0	LV T 2021[1]	probiotics intervention (any dose, strain, or administration method)	8-12 weeks	All RCTs	adults age ≥18 years	probiotics intervention (any dose, strain, or administration method) versus control	SMD	Random	NP
cognitive function in individuals with Alzheimer's disease	0.56 (-0.06 to 1.18)*	3	82/161	73.0	KRÜGER J F 2021[2]	Probiotic or synbiotic, orally or enterally administered, with no restriction on strains, doses, and frequency and duration of administration, provided information was reported	NP	All RCTs	individuals with dementia who underwent an intervention with probiotics or synbiotics	probiotics or synbiotics intervention versus control or placebo group	SMD	Random	NP
cognitive enhancement	0.37 (0.14 to 0.61)	5	154/297	24.0	DENG H Y 2020 [3]	Probiotic	12 weeks	All RCTs	Adult human participants who had a diagnosis of AD or MCI (aged over 18 y)	Probiotics versus placebo	SMD	fixed	0.54
alleviating anxiety	-0.12 (-0.28 to 0.04)*	12	1551	51.0	LIU B 2018 [4]	probiotics	4-24 weeks	All RCTs	Adult (≥ 18 y)	probiotics vs. placebo	SMD	random	0.139
Preclinical psychological symptoms of anxiety, depression, and stress	0.34 (0.07 to 0.61)	9	785	67.0	MCKEAN J 2017[5]	probiotics	21-56 days	All RCTs	adult healthy volunteers (aged ≥ 18 years)	probiotics vs. placebo	SMD	random	NP

depressive symptoms	-0.31 (-0.56 to -0.07)	19	1901	82.0	GOH K K 2019 [6]	probiotics	4-20 weeks	All RCTs	general population or clinical population	probiotics vs. placebo	SMD	random	No serious bias
	-0.75 (-1.09 to -0.41)	3	144	0.0					patients with major depressive disorder				
	-0.26 (-0.70 to -0.17)	7	722	84.0					Other clinical diagnosis population				
	-0.25 (-0.60 to 0.11)*	12	1035	82.0					Health population				
	-0.01 (-0.30 to 0.27)*	11	1070	71.0		Single strain probiotics			general population or clinical population				
	-0.57 (-0.96 to -0.18)	13	831	85.0		multiple strain probiotics							
schizophrenia symptoms (total PANSS score)	-0.09 (-0.38 to 0.20)*	3	172	0.0	NG Q X 2019[7]	probiotic	NP	All RCTs	patients with at least moderately severe psychotic symptoms, aged 18–65 years	probiotics vs. Placebo	SMD	fixed	NA
Subjective stress level	-0.14 (-0.27 to -0.01)	6	1043	0.0	ZHANG N 2020[8]	Probiotics	30 days to 24 weeks	All RCTs	participants were in a healthy state, without known major health problems	probiotics vs placebo	SMD	fixed	NA
Stress-related subthreshold anxiety/depression level	-0.13 (-0.26 to -0.00)	9	940	11.0									
Anxiety	-0.23 (-0.54 to 0.08)*	3	171	0.0	WILSON B 2019[9]	prebiotics	2-12 weeks	All RCTs	Adult patients ≥18 and ≤64 y with Irritable bowel syndrome (IBS) or	vs. placebo	SMD	random	No serious bias

									other Functional bowel disorders (FBDs)				
continuous anxiety outcomes	-0.03(-0.21 to 0.14)*	5	617	12.0	COHEN KADOSH K 2021[10]	pro- or prebiotic administration (any form)	8-84 days	Controlled trials	Mean age in the range of 10–24 years old	vs. controls	SMD	random	NP
depression	-0.08 (-0.30 to 0.15)*	5	NP	NP	Liu RT 2019[11]	prebiotic	four hours to four weeks	controlled clinical trial	Patient with depression or anxiety	Vs. placebo	SMD	random	NP
anxiety	0.12 (-0.03 to 0.27)*	4											
depression	-0.24 (-0.36 to -0.12)	23		48.2		probiotic	eight days to 45 weeks						0.08
anxiety	-0.10 (-0.19 to -0.01)	22		5.0									0.49

* No statistical significance; CI, confidence interval; RCT, randomized controlled trial; RR, relative risk; HR, hazard ratio; MD, mean difference; SMD, standard mean difference; WMD, weighted mean difference; OR, odds ratio; NA, not available; NP, not published.

References

1. Lv, T.; Ye, M.; Luo, F.; Hu, B.; Wang, A.; Chen, J.; Yan, J.; He, Z.; Chen, F.; Qian, C.; et al. Probiotics treatment improves cognitive impairment in patients and animals: A systematic review and meta-analysis. *Neuroscience & Biobehavioral Reviews* **2021**, *120*, 159-172, doi: 10.1016/j.neubiorev.2020.10.027.
2. Krüger, J.F.; Hillesheim, E.; Pereira, A.C.S.N.; Camargo, C.Q.; Rabito, E.I. Probiotics for dementia: a systematic review and meta-analysis of randomized controlled trials. *Nutr. Rev.* **2021**, *79*, 160-170, doi: 10.1093/nutrit/nuaa037.
3. Den, H.; Dong, X.; Chen, M.; Zou, Z. Efficacy of probiotics on cognition, and biomarkers of inflammation and oxidative stress in adults with Alzheimer's disease or mild cognitive impairment — a meta-analysis of randomized controlled trials. *Aging* **2020**, *12*, 4010-4039, doi: 10.18632/aging.102810.
4. Liu, B.; He, Y.; Wang, M.; Liu, J.; Ju, Y.; Zhang, Y.; Liu, T.; Li, L.; Li, Q. Efficacy of probiotics on anxiety-A meta-analysis of randomized controlled trials. *Depress. Anxiety* **2018**, *35*, 935-945, doi: 10.1002/da.22811.
5. Mckean, J.; Naug, H.; Nikbakht, E.; Amiet, B.; Colson, N. Probiotics and Subclinical Psychological Symptoms in Healthy Participants: A Systematic Review and Meta-Analysis. *The Journal of Alternative and Complementary Medicine* **2017**, *23*, 249-258, doi: 10.1089/acm.2016.0023.

6. Goh, K.K.; Liu, Y.; Kuo, P.; Chung, Y.E.; Lu, M.; Chen, C. Effect of probiotics on depressive symptoms: A meta-analysis of human studies. *Psychiat. Res.* **2019**, *282*, 112568, doi: 10.1016/j.psychres.2019.112568.
7. Ng, Q.X.; Soh, A.Y.S.; Venkatanarayanan, N.; Ho, C.Y.X.; Lim, D.Y.; Yeo, W. A Systematic Review of the Effect of Probiotic Supplementation on Schizophrenia Symptoms. *Neuropsychobiology* **2019**, *78*, 1-6, doi: 10.1159/000498862.
8. Zhang, N.; Zhang, Y.; Li, M.; Wang, W.; Liu, Z.; Xi, C.; Huang, X.; Liu, J.; Huang, J.; Tian, D.; et al. Efficacy of probiotics on stress in healthy volunteers: A systematic review and meta-analysis based on randomized controlled trials. *Brain and Behavior* **2020**, *10*, doi: 10.1002/brb3.1699.
9. Wilson, B.; Rossi, M.; Dimidi, E.; Whelan, K. Prebiotics in irritable bowel syndrome and other functional bowel disorders in adults: a systematic review and meta-analysis of randomized controlled trials. *The American Journal of Clinical Nutrition* **2019**, *109*, 1098-1111, doi: 10.1093/ajcn/nqy376.
10. Cohen Kadosh, K.; Basso, M.; Knytl, P.; Johnstone, N.; Lau, J.Y.F.; Gibson, G.R. Psychobiotic interventions for anxiety in young people: a systematic review and meta-analysis, with youth consultation. *Transl. Psychiat.* **2021**, *11*, doi: 10.1038/s41398-021-01422-7.
11. Liu, R.T.; Walsh, R.F.L.; Sheehan, A.E. Prebiotics and probiotics for depression and anxiety: A systematic review and meta-analysis of controlled clinical trials. *Neuroscience & Biobehavioral Reviews* **2019**, *102*, 13-23, doi: 10.1016/j.neubiorev.2019.03.023.
