# Supplementary material for: Gastrointestinal Microbiome and Multiple Health Outcomes: Umbrella Review
Source: Nutrients. 2022 Sep 9;14(18):3726. doi: 10.3390/nu14183726 (PMC9505003; doi:10.3390/nu14183726)
Supplement: Supplementary file 1 [file nutrients-14-03726-s001.zip › Supplementary Table S6.pdf]

Supplement Table S6. Prebiotics/probiotics/synbiotics and maternal and infant outcomes.

outcome(s) of interest	estimated summary effect (95% CI)	number of studies / total studies	number of intervention group or total participants	Heterogeneity (I^2, %)	the first author + year of publication	Intervention	Duration of Intervention/follow-up	study design	populations	outcome comparison	meta-analysis metric	type of effect model	publication bias
Occurrence of new cases of colic at study end, as defined by the Wessel criteria.	0.46 (0.18 to 1.19)*	3	571/1148	72.0	ONG T G 2019 [1]	any probiotic, alone or in combination with a prebiotic (also known as synbiotics)	NP	All RCTs	newborn infants less than one month of age without the diagnosis of infantile colic	any probiotic, alone or in combination with a prebiotic (also known as synbiotics), versus no intervention, another intervention(s) or placebo	RR	random	NA
Adverse effects, including parental depression and mental illness, choking, bacterial infection or apparent life-threatening/serious events (dichotomous outcome)	1.02 (0.14 to 7.21)*	6 (only one trial got estimated)	1851(1014)	-									
Duration of crying	-32.57 (-55.60 to -9.54) min per day	3	355/707	93.0							MD		
	-20.65 (-47.23 to -5.92) min per day	2	345/687	89.0					term babies only				
Effects on Crying Duration	-2.01 (-2.76 to -1.26)	12	NP	95.2	SKONIECZNA-ŻYDECKA K 2020 [2]	probiotics/synbiotics	21-90 days	All RCTs	An infant who is less than 5 months of age when the symptoms start and finish	newborns taking probiotics vs. placebos (controls)	SMD	random	0.0021
Response (The probability of at least a 50% reduction in crying duration) to Probiotic Intervention	1.98 (1.50 to 2.62)	9		68.9									0.5

mean cognitive	0.20 (-1.42 to 1.83)*	4	1388	0.0	UPADHYAY R P 2020 [3]	probiotic and/or prebiotic supplementation	NP	All RCTs	preterm infants (<37 weeks gestation and/or birth weight <1500 g) with intervention being probiotic and/or prebiotic supplementation	probiotic vs. Control group	WMD	random	No serious bias
motor scores in children under 5 years of age	1.04 (-0.43 to 2.50)*	4	1388	0.0									
the risk of cognitive impairment	0.98 (0.75 to 1.27)*	4	1388	0.0							RR		
the risk of motor impairment	1.06 (0.79 to 1.42)*	4	1388	0.0									
the risk of neurodevelopment impairment (NDI)	0.90 (0.75 to 1.09)*	5	1637	9.9									
the risk of cerebral palsy	1.33 (0.84 to 2.11)*	4	1388	0.0									
the risk of hearing impairment	0.38 (0.14 to 1.07)*	3	1139	37.5									
Necrotising enterocolitis (before hospital discharge)	0.54 (0.45 to 0.65)	54	10604	17.0	SHARIF S 2020 [4]	any probiotic or probiotic combination for at least one week	at least one week	RCTs and quasi - RCTs	very preterm (< 32 weeks' gestation) or very low birth weight (< 1500 g) infants	any probiotic or probiotic combination vs. placebo or no treatment.	RR	fixed	0.04
Invasive infection (before hospital discharge)	0.89 (0.82 to 0.97)	47	9762	19.0									0.86
Duration of birth hospitalisation	-1.93 (-3.78 to -0.08) days	22	5458	26.0							MD		No serious bias
neurodevelopmental impairment	1.03 (0.84 to 1.26)*	5	1518	0.0							RR		NP
Cerebral palsy	1.13 (0.74 to 1.72)*	5	1512	18.0									
Visual impairment	0.50 (0.14 to 1.80)*	4	1356	0.0									
Hearing impairment	0.46 (0.18 to 1.17)*	4	1356	32.0									
Necrotising enterocolitis (before hospital discharge)	0.90 (0.68 to 1.21)*	8	1712	0.0					extremely preterm (< 28 weeks' gestation) or extremely low birth weight (< 1000 g)				
Invasive infection (before hospital discharge)	0.90 (0.76 to 1.06)*	6	1471	0.0									

									infants				
Effect of probiotics on Stage I or higher necrotizing enterocolitis (NEC) (Bell stage ≥I; gestational age <37 weeks)	0.35 (0.27 to 0.44)	27	3298/6655	0.0	YANG Y 2014 [5]	enteral administration of any probiotic started within the first 10 days of life	at least 7 days	All RCTs	preterm infants (without consideration of birth weight)	probiotic vs. Placebo group	RR	fixed	No obvious bias
the effect of probiotics on Stage II or higher NEC(Bell Stage ≥II; gestational age <37 weeks)	0.34 (0.25 to 0.48)	17	2042/4198	0.0									
the influence on NEC in early preterm infants (Bell Stage ≥I; gestational age <34 weeks)	0.39 (0.27 to 0.56)	12	1266/2495	0.0									
culture-positive sepsis	0.94 (0.83 to 1.06)*	17	1950/4043	24.2									
age reaching full feeds	-1.66 (-3.60 to 0.27)*	9	821/1621	89.9							WMD	random	
The effect of probiotics in preventing stage II-III severe NEC	0.51 (0.38 to 0.67)	27	4491/8901	28.4	JIANG T L 2020 [6]	probiotics	NP	All RCTs	preterm infants < 37 weeks and/or birth weight < 2500 g	Probiotics vs. control	RR	random	No obvious bias
	0.39 (0.26 to 0.57)	11	1590/3083	0.0		mixed probiotics							
The effect of probiotics in decreasing death rate	0.69 (0.55 to 0.87)	27	4399/8717	36.2		probiotics							
	0.52 (0.34 to 0.80)	12	1635/3175	42.7		mixed probiotics							
incidence of Hirschsprung-associated entero-colitis (HAEC)	0.72 (0.37 to 1.39)*	5	93/198	37.0	NAKAMURA H 2018 [7]	probiotics	NP	NP	premature infants	Probiotics vs. control	OR	fixed	NP
Incidence of Bell stage ≥ II NEC	0.34 (0.25 to 0.46)	23	2359/4686	0.0	LIU D P 2020[8]	administration of Lactobacillus for preventing NEC	NP	All RCTs	neonates of gestational age < 37 weeks	Lactobacillus with placebo	RR	fixed	No obvious bias
Incidence of sepsis	0.90 (0.72 to 1.12)*	10	2129	41.0								random	
Incidence of death.	0.48 (0.36	20	4286	0.0								fixed	

	to 0.64)												
Incidence of NEC Stage ≥ II (Bell staging criteria)	0.38 (0.25to 0.58)	20	2836/5568	43.0	ZHU X L 2019[9]	bifidobacteria treatment for preventing NEC	NP	All RCTs	neonates with gestational age (GA) < 37 weeks	Bifidobacteria vs. control	RR	random	NP
Incidence of sepsis	0.87 (0.73 to 1.03)*	17	2075/4034	43.0									
Incidence of death.	0.74 (0.60 to 0.92)	15	2579/5053	29.0								fixed	
infant crying/distress, measured as duration or the number of episodes	-65.10 (-85.85 to -44.38)	3	209	55.0	SUNG V 2013 [10]	probiotics	NP	All RCTs	infants who were younger than 3 months	oral probiotic supplementation vs. placebo or standard care or no care	Median Difference	Random	NP
	-67.72 (-99.79 to -35.64)	3	209	70.0							Mean Difference		
Preterm birth < 37 weeks' gestation	0.92(0.32 to 2.67)*	4	518	14.0	GREV J 2018 [11]	Before delivery (probiotics given only prior to delivery)	NP	All RCTs	pregnant women	probiotics administered to pregnant women (< 36 weeks' gestation) versus placebo or no intervention	RR	fixed	NP
Newborn Birth Weight*	-10.27 (-90.17 to 69.63)*	7	1093	34.0	WANG C C 2020[12]	probiotic or synbiotic	NP	All RCTs	Pregnant women (> 18 years) with body mass index (BMI) ≥ 25 kg/m2 or diagnosed as having GDM, gestational age < 34 weeks	probiotic or synbiotic vs. placebo	SMD	random	0.60
fasting blood glucose	-0.11 (-0.16 to -0.05)mmol/L	10	1139	71.0	HAN M M 2019 [13]	probiotic	NP	All RCTs	pregnant women >18 years-of-age with or without gestational diabetes mellitus	probiotic vs. placebo	MD	random	No obvious bias
serum insulin	-2.06 (-2.98 to -1.15)µU/mL	8	340/682	77.0									

(HOMA-IR)	-0.38 (-0.54 to -0.21)μU/mL	8	340/682	64.0					(GDM)				
QUICKI	0.01 (0.00 to 0.03)	4	165/327	85.0									
total cholesterol	-0.56 (-1.07 to -0.05)	6	579	89.0							SMD		
triglycerides	-0.66 (-1.28 to -0.04)	6	579	92.0									
HDL-C	-0.13 (-0.34 to 0.07)*	6	579	33.0									
LDL-C	-0.45 (-0.97 to 0.06)*	6	579	89.0									
Diagnosis of gestational diabetes mellitus	0.80 (0.54 to 1.20)*	6	1440	64.0	DAVIDSON S 2021[14]	probiotics	NP	All RCTs	pregnant women not previously diagnosed with diabetes mellitus	Probiotics vs. placebo	RR	random	NP
Hypertensive disorders of pregnancy (including pre-eclampsia, pregnancy-induced hypertension and eclampsia)	1.39 (0.96 to 2.01)*	4	955	0.0									
pre-eclampsia	1.85 (1.04 to 3.29)*	4	955	0.0									
Caesarean section	1.00 (0.86 to 1.17)*	6	1520	0.0									
Large-for-gestational age	0.99 (0.72 to 1.36)*	4	919	0.0									
Perinatal mortality (including stillbirth and neonatal death)	0.33 (0.01 to 8.02)*	3	709	0.0									
Maternal weight gain during pregnancy	0.30 (-0.67 to 1.26) kg*	4	853	40.0							MD		
Maternal fasting plasma glucose	-0.14 (-0.12 to 0.05) mmol/L*	7	1519	69.0									

Maternal plasma glucose at one hour of a 75 g oral glucose tolerance test (OGTT)	-0.07 (-0.27 to 0.13) mmol/L*	4	1110	0.0									
Maternal plasma glucose at two hours of a 75 g OGTT	0.02 (-0.13 to 0.18) mmol/L*	4	1186	0.0									
Infant Stillbirth	0.59 (0.14 to 2.46)*	5	1128	0.0							RR		
Preterm birth (less than 37 weeks' gestation and less than 32 weeks' gestation)	1.36 (0.82 to 2.01)*	6	1484	0.0									
Infant Macrosomia	1.13 (0.86 to 1.48)*	3	952	20.0									
Small-for-gestational age	0.51 (0.30 to 0.85)*	3	814	0.0									
Birthweight	26.87 (-49.52 to 103.26) g*	6	1524	42.0							MD		
Infant Head circumference	-0.04 (-0.27 to 0.18) cm*	3	789	21.0									
Infant Length	0.02 (-0.54 to 0.59) cm*	3	786	59.0									
fasting plasma glucose	-3.10 (-5.11 to -1.09) mg / dL	9	594	72.0	HASAIN Z 2021[15]	probiotics supplementation after GDM diagnosis, regardless of gestational age, for at least a 4-week duration	at least 4-week	All RCTs	adult pregnant women regardless of weight status (normal, overweight, or obese) diagnosed with GDM according to oral glucose tolerance test and were not	probiotics vs. placebo	MD	random	NP
insulin	-2.17 (-3.55 to -0.79) µIU/mL	7	450	72.0									
HOMA, homeostasis model assessment index	-0.56 (-0.86 to -0.26)	7	450	64.0									
QUICKI, quantitative insulin sensitivity check	0.01 (0.00 to 0.01)	4	221	60.0									

index					on any hypoglycemic agents		
total cholesterol level	-3.60 (-16.26 to 9.07) mg/dL	4	265	39.0			
high-density lipoprotein cholesterol	-1.25 (-5.48 to 2.99) mg/dL	4	265	59.0			
low-density lipoprotein cholesterol level	-6.36 (-14.89 to 2.16) mg/dL	4	265	0.0			
triglycerides levels	-10.40 (-24.68 to 3.89)mg/dL	4	265	0.0			
high-sensitivity C-reactive protein (hs-CRP)	-1.37 (-1.94 to -0.81) mg/L	4	275	24.0			
nitric oxide (NO)	2.42 (0.80 to 4.04) μmol/L	3	165	0.0			
malondialdehyde (MDA)	-0.77 (-0.99 to -0.56) μmol/L	4	221	0.0			
glutathione (GSH)	13.73 (-35.84 to 63.31) μmol/L	3	165	48.0			
total antioxidant capacity (TAC)	93.46 (-7.31 to 194.22) mmol/L	3	165	78.0			
incidence of pre-eclampsia	0.89 (0.32 to 2.46)	3	217	0.0		probiotics vs. control	OR
gestational age at delivery	0.04 (-0.30 to 0.38) week	4	301	0.0		probiotics vs. placebo	MD
the likelihood of cesarean delivery	0.66 (0.34 to 1.29)*	4	301	39.0			OR
macrosomia	0.42 (0.19	5	361	12.0			

	to 0.94)											
newborns' hospitalization	0.37 (0.18 to 0.74)	4	301	6.0								
babies with hypoglycemia at birth	0.68 (0.28 to 1.64)*	3	174	0.0						probiotics vs. control		
Infant Eczema	0.60 (0.47 to 0.78)	10	2093	67.0	SUN M 2021[16]	a mixture of Lactobacillus and Bifidobacterium	0.5-2 years	All RCTs	healthy or pregnant women and infants under three years old with a family history of atopic disease	Vs. placebo intervention, and no other probiotic interventions or no intervention	RR	random	NP
Occurrence of Adverse Events	1.09 (0.83 to 1.44)*	6	1309	0.0								
the risk of eczema in children	0.90 (0.67 to 1.21)*	5	778	45.0	Szajewska H 2018[17]	Lactobacillus rhamnosus GG regardless of the timing of LGG administration	NP	All RCTs	healthy (1) pregnant women at high risk for having an allergic child; (2) breastfeeding mothers of infants at high risk of developing allergy; or (3) healthy term infants at high risk of developing allergy	vs. placebo or no intervention	RR	random	NA

* No statistical significance; CI, confidence interval; RCT, randomized controlled trial; RR, relative risk; HR, hazard ratio; MD, mean difference; SMD, standard mean difference; WMD, weighted mean difference; OR, odds ratio; NA, not available; NP, not published.

References

1. Ong, T.G.; Gordon, M.; Banks, S.S.; Thomas, M.R.; Akobeng, A.K. Probiotics to prevent infantile colic. *Cochrane Db. Syst. Rev.* **2019**, doi: 10.1002/14651858.CD012473.pub2.
2. Skonieczna-żydecka, K.; Janda, K.; Kaczmarczyk, M.; Marlicz, W.; łoniewski, I.; łoniewska, B. The Effect of Probiotics on Symptoms, Gut Microbiota and Inflammatory Markers in Infantile Colic: A Systematic Review, Meta-Analysis and Meta-Regression of Randomized Controlled Trials. *Journal of Clinical Medicine* **2020**, *9*, 999, doi: 10.3390/jcm9040999.
3. Upadhyay, R.P.; Taneja, S.; Chowdhury, R.; Strand, T.A.; Bhandari, N. Effect of prebiotic and probiotic supplementation on neurodevelopment in preterm very low birth weight infants: findings from a meta-analysis. *Pediatr. Res.* **2020**, *87*, 811-822, doi: 10.1038/s41390-018-0211-9.
4. Sharif, S.; Meader, N.; Oddie, S.J.; Rojas-Reyes, M.X.; Mcguire, W. Probiotics to prevent necrotising enterocolitis in very preterm or very low birth weight infants. *Cochrane Db. Syst. Rev.* **2020**, *2020*, D5496, doi: 10.1002/14651858.CD005496.pub5.
5. Yang, Y.; Guo, Y.; Kan, Q.; Zhou, X.G.; Zhou, X.Y.; Li, Y. A meta-analysis of probiotics for preventing necrotizing enterocolitis in preterm neonates. *Braz. J. Med. Biol. Res.* **2014**, *47*, 804-810, doi: 10.1590/1414-431X20143857.
6. Jiang, T.; Zhang, H.; Xu, X.; Li, H.; Yang, J. Mixed probiotics decrease the incidence of stage II-III necrotizing enterocolitis and death: A systematic review and meta-analysis. *Microb. Pathogenesis* **2020**, *138*, 103794, doi: 10.1016/j.micpath.2019.103794.
7. Nakamura, H.; Lim, T.; Puri, P. Probiotics for the prevention of Hirschsprung-associated enterocolitis: a systematic review and meta-analysis. *Pediatr. Surg. Int.* **2018**, *34*, 189-193, doi: 10.1007/s00383-017-4188-y.
8. Liu, D.; Shao, L.; Zhang, Y.; Kang, W. Safety and efficacy of Lactobacillus for preventing necrotizing enterocolitis in preterm infants. *Int. J. Surg.* **2020**, *76*, 79-87, doi: 10.1016/j.ijsu.2020.02.031.
9. Zhu, X.; Tang, X.; Qu, F.; Zheng, Y.; Zhang, W.; Diao, Y. Bifidobacterium may benefit the prevention of necrotizing enterocolitis in preterm infants: A systematic review and meta-analysis. *Int. J. Surg.* **2019**, *61*, 17-25, doi: 10.1016/j.ijsu.2018.11.026.
10. Sung, V.; Collett, S.; de Gooyer, T.; Hiscock, H.; Tang, M.; Wake, M. Probiotics to Prevent or Treat Excessive Infant Crying. *JAMA Pediatr.* **2013**, *167*, 1150, doi: 10.1001/jamapediatrics.2013.2572.
11. Grev, J.; Berg, M.; Soll, R. Maternal probiotic supplementation for prevention of morbidity and mortality in preterm infants. *Cochrane Db. Syst. Rev.* **2018**, *2018*, D12519, doi: 10.1002/14651858.CD012519.pub2.
12. Wang, C.; Tung, Y.; Chang, H.; Lin, C.; Chen, Y. Effect of Probiotic Supplementation on Newborn Birth Weight for Mother with Gestational Diabetes Mellitus or Overweight/Obesity: A Systematic Review and Meta-Analysis. *Nutrients* **2020**, *12*, 3477, doi: 10.3390/nu12113477.
13. Han, M.; Sun, J.; Su, X.; Peng, Y.; Goyal, H.; Wu, C.; Zhu, X.; Li, L. Probiotics improve glucose and lipid metabolism in pregnant women: a meta-analysis. *Annals of Translational Medicine* **2019**, *7*, 99, doi: 10.21037/atm.2019.01.61.
14. Davidson, S.J.; Barrett, H.L.; Price, S.A.; Callaway, L.K.; Dekker Nitert, M. Probiotics for preventing gestational diabetes. *Cochrane Db. Syst. Rev.* **2021**, *2021*, D9951, doi: 10.1002/14651858.CD009951.pub3.
15. Hasain, Z.; Che Roos, N.A.; Rahmat, F.; Mustapa, M.; Raja Ali, R.A.; Mokhtar, N.M. Diet and Pre-Intervention Washout Modifies the Effects of Probiotics on Gestational Diabetes Mellitus: A Comprehensive Systematic Review and Meta-Analysis of Randomized Controlled Trials. *Nutrients* **2021**, *13*, 3045, doi: 10.3390/nu13093045.

16. Sun, M.; Luo, J.; Liu, H.; Xi, Y.; Lin, Q. Can Mixed Strains of Lactobacillus and Bifidobacterium Reduce Eczema in Infants under Three Years of Age? A Meta-Analysis. *Nutrients* **2021**, *13*, 1461, doi: 10.3390/nu13051461.
17. Szajewska, H.; Horvath, A. Lactobacillus rhamnosus GG in the Primary Prevention of Eczema in Children: A Systematic Review and Meta-Analysis. *Nutrients* **2018**, *10*, 1319, doi: 10.3390/nu10091319.
