# Supplementary material for: Gastrointestinal Microbiome and Multiple Health Outcomes: Umbrella Review
Source: Nutrients. 2022 Sep 9;14(18):3726. doi: 10.3390/nu14183726 (PMC9505003; doi:10.3390/nu14183726)
Supplement: Supplementary file 1 [file nutrients-14-03726-s001.zip › Supplementary Table S7.pdf]

Supplement Table S7. Prebiotics/probiotics/synbiotics and other outcomes.

outcome(s) of interest	estimated summary effect (95% CI)	number of studies / total studies	number of intervention group or total participants	Heterogeneity (I^2, %)	the first author + year of publication	Intervention	Duration of Intervention/follow-up	study design	populations	outcome comparison	meta-analysis metric	type of effect model	publication bias
Detection of C. difficile in stool	0.86 (0.67 to 1.10)*	15	1214	0.0	GOLDENBERG J Z 2017[1]	probiotics	NP	All RCTs	participants taking antibiotics	any versus none	RR	random	No serious bias
Length of hospital stay	-0.17 (-1.03 to 0.68)*	4	NP	0							MD		
the incidence of infection	0.27(0.21 to 0.36)	16	1243/2483	0.0	CHEN X R 2021[2]	early enteral nutrition (EEN) combined with probiotics	≥ 14 days	All RCTs	All patients met the criteria of severe stroke	early enteral nutrition (EEN) combined with probiotics versus EEN	OR	Random	No serious bias
the incidence of pulmonary infection	0.28(0.20 to 0.39)	16	698/1391	0.0									
the incidence of urinary tract infection	0.23(0.13 to 0.43)	9	384/768	0.0									
the incidence of gastrointestinal infection	0.30(0.14to 0.66)	4	161/324	0.0									
the enteral nutrition target time	-2.18(-2.56 to -1.80)	4	163/324	84.0							MD		
the length of hospital stay	-8.70(-13.24 to -4.16)	3	131/260	98.0									
Effects on Body Weight	-0.55 (-0.91 to -0.19) kg	10	315/641	64.0	WANG Z 2019[3]	Probiotics	NP	All RCTs	adults (≥18 years old) individuals with a BMI > 25 kg m-2	the probiotics vs. control groups	MD	random	0.446
Effects on Body Mass Index (BMI)	-0.30 (-0.43 to -0.18) kg/m^2	11	357/717	59.0									0.006
	-0.29 (-0.46 to -0.12) kg	6	213	66.6						Probiotics dosage ≥ 10^{10} CFU vs. control			NP

	m-2				groups	
	-0.18 (-0.48 to 0.12) kg m-2*	4	119	46.9	Probiotics dosage < 10^{10} CFU vs. control groups	
	-0.36 (-0.52 to 0.20) kg m-2*	4	138	56.8	Single probiotics species vs. control groups	
	-0.15 (-0.39 to 0.10) kg m-2*	6	194	55.5	Multiple probiotics species vs. control groups	
Effects on Waist Circumference	-1.20 (-2.21 to -0.19) cm	8	281/573	90.0	the probiotics vs. control groups	0.403
	-1.53 (-2.64 to -0.41) cm	6	212	86.9	Probiotics dosage ≥ 10^{10} CFU vs. control groups	NP
	-0.32 (-0.84 to 0.20) cm*	2	69	0.0	Probiotics dosage < 10^{10} CFU vs. control groups	
	-1.69 (-3.04 to 0.33) cm	4	168	91.6	Single probiotics species vs. control groups	
	-0.42 (-0.91 to 0.07) cm*	4	113	0.0	Multiple probiotics species vs. control groups	
	-1.34 (-2.76 to 0.08) cm*	6	187	92.3	Form of probiotics: Capsule or powder vs. control groups	
	-1.11 (-1.64 to -0.59) cm	2	94	0.0	Form of probiotics: Food vs. control groups	
Effects on Fat	-0.91 (-1.19 to	11	311/632	43.0	the probiotics vs. control	0.335

Mass	-0.63) kg									groups			
Effects on Fat Percentage	-0.92 (-1.27 to -0.56) %	6	224/450	57.0						the probiotics vs. control groups			0.068
the effect on body weight	-0.54 (-1.09 to 0.01) kg*	18	660/1239	0.0	SUZUMURA E A 2019 [4]	Oral supplementation with probiotics or synbiotics as an isolated intervention or as part of a dietary plan	3-24weeks	Randomized or quasi-randomized controlled trials	Overweight or obese adults (BMI ≥ 25 kg/m² and age ≥ 18 y) with no health conditions that potentially affect metabolism or body weigh	probiotics or synbiotics oersus Control	MD	random	No serious bias
the effect on BMI	-0.19 (-0.43 to 0.04) kg/m^2*	15	447/895	51.0									
the effect on waist circumference	-0.82 (-1.43 to -0.21) cm	12	455/864	46.0									
the effect on body weight	-1.24 (-2.57 to 0.01) kg*	4	147/301	0.0						synbiotics vs control			NP
the effect on BMI	-0.33 (-1.22 to 0.57) kg/m^2*	2	50/100	0.0									
the effect on waist circumference	-3.39 (-5.07 to -1.72) cm	2	72/148	34.0									
effect of the PFMP on body weight	-0.91 (-1.59 to -0.22) kg	16	NP	67.3	MOHAMMADI H 2021 [5]	Probiotics fermented milk products (PFMP)	2-12weeks	All RCTs	adult individuals >18 years old	PFMP versus placebo	WMD	random	0.555
effect of the PFMP on BMI	-0.33 (-0.48 to 0.18) kg/m^2*	15		0.0									0.404
effect of the PFMP on waist circumference	-0.59 (-1.55 to 0.36) cm*	6		0.0									0.348
effect of the PFMP on body fat percentage	-3.75 (-9.39 to 1.90)*	5		90.3									0.402
The effects of synbiotic supplementation on BMI	-0.12 (-0.40 to 0.16) kg/m^2*	22	NP	0.0	HADI A 2020 [6]	synbiotic supplements	≥4 weeks	All randomized clinical	participants over18 years	synbiotic versus placebo	WMD	random	0.21

The effects of synbiotic supplementation on WC	-2.07 (-3.11 to -1.03) cm	12	0.0	trials	synbiotic versus placebo	0.68
	-1.72 (-3.49 to 0.05) cm*	7	0.0		Baseline BMI ≥ 30 versus placebo	NP
	-2.25 (-3.53 to -0.97) cm	5	0.0		Baseline BMI 25-30 versus placebo	
	-1.30 (-3.44 to 0.83) cm*	6	0.0		Duration ≥ 12 Weeks versus placebo	
	-2.31 (-3.49 to -1.12) cm	6	0.0		Duration < 12 Weeks versus placebo	
	-1.97 (-3.06 to -0.86) cm	9	0.0		Number of used strain: Multi versus placebo	
	-2.70 (-5.54 to 0.15) cm*	3	0.0		Number of used strain: Single versus placebo	
	-2.269(-4.76 to 0.37) cm*	3	0.0		Probiotic Dose > 2×10^8 CFU versus placebo	
	-1.69 (-3.23 to -0.16) cm	8	0.0		Probiotic Dose ≤ 2×10^8 CFU versus placebo	
The effects of synbiotic supplementation on weight	-0.80 (-1.56 to -0.03) kg	20	0.0		synbiotic versus placebo	0.37
	-0.54 (-2.93 to 1.85) kg*	9	0.0		Duration ≥ 12 Weeks versus placebo	NP
	-0.83 (-1.63 to -0.02) kg	11	0.0		Duration < 12 Weeks versus placebo	
	-1.48 (-2.68 to -0.28) kg	14	0.0		Number of used strain: Multi versus placebo	

	-0.33 (-1.32 to 0.67) kg*	6		0.0						Number of used strain:<br>Single versus placebo			
The effects of synbiotic supplementation on body fat	0.02 (-1.27 to 1.87) %*	6		67.3						synbiotic versus placebo			0.38
Effects on waist-to-hip ratio	-0.01 (-0.01 to -0.01)	5	192/395	24.0	PONTES K S D S 2021 [7]	probiotics	NP	All RCTs	adults (18 years or older) with overweight or obesity defined according to local standards for body mass index (BMI)	Probiotics versus placebo	MD	random	No serious bias
Effects on total abdominal fat area	-9.69 (-14.47 to -4.91) cm^{2}	4	350	0.0									
Effects on subcutaneous abdominal fat area	-6.24 (-8.94 to -3.54) cm^{2}	5	445	0.0									
Effects on visceral abdominal fatarea	-3.46 (-6.55 to 0.38) cm^{2*}	4	350	41.0									
Effects on Homeostasis Model of Assessment for Insulin Resistance Index (HOMA-IR)	-0.08 (-0.34 to 0.18) *	9	322/765	47.0									
Effects of probiotics on systolic blood pressure	-0.23 (-0.57 to 0.12)mm Hg*	7	318/641	0.0									
Effects of probiotics on diastolic blood pressure	-0.35 (-0.76 to 0.06)mm Hg*	7	318/641	0.0									
the risk of atopic sensitization	0.89 (0.77 to 1.03)*	17	2974	9.0	ZHANG G 2016 [8]	probiotics/synbiotics	Follow-up:1y - 2y of age	All RCTs	children in whom outcome assessment performed	any species/strains/ doses regimen of live probiotics	RR	random	0.988

									during infancy or childhood (ie, up to 12 years of age), without atopic diseases at the time of probiotic administration	administered prenatally and/or postnatally within the first year of life versus placebo or no probiotics			
	0.78 (0.66 to 0.92)	12	1159/2085	0.0						probiotics were administered both prenatally and postnatally versus placebo or no probiotics			NP
Food Sensitization	0.92 (0.75 to 1.12)*	9	1506	7.0						any species/strains/ doses regimen of live probiotics administered prenatally and/or postnatally within the first year of life versus placebo or no probiotics			0.806
	0.77 (0.61 to 0.98)	5	477/807	0.0						probiotics were administered both prenatally and postnatally versus placebo or no probiotics			NP
number of pulmonary exacerbations	-0.32 (-0.68 to 0.03) episodes per participant*	4	113/225	41.0	COFFEY M J 2020 [9]	oral probiotic formulation	4-12 months	All RCTs	participants who fulfil consensus diagnostic criteria for CF (Cystic fibrosis)	Any oral probiotic formulation (any strain(s), dose or formulation, with or without a prebiotic) compared to any other probiotic formulation, placebo or no treatment	MD	random	NP
intestinal i. calprotectin	-47.41 (-93.28 to -1.54) µg/g	4	88/177	0.0									
FEV1 (% predicted)	1.36 (-1.20 to 3.91)%	5	143/284	0.0									

	*									control			
body mass index	-0.25 (-0.48 to -0.03) kg/m^2	7	208/416	97.0	COZZOLINO M 2020 [10]	therapy with probiotics or synbiotics.	administration of probiotics or synbiotics at least for 8 weeks	All RCTs	women with Polycystic ovary syndrome (PCOS) according to Rotterdam criteria undergoing therapy with probiotics or symbiotic	administration of probiotics or Synbiotics versus without therapy with probiotics or synbiotics or placebo	MD	random	NP
body weight	-0.75 (-1.45 to -0.05) kg	7	208/416	98.0									
the modified Ferriman–Gallway score	-1.00 (-1.75 to -0.26)	3	90/180	83.0									
Body Weight	-0.11 (-0.34 to 0.13)*	12	731	61.5	LI Y L 2021 [11]	probiotics, prebiotics, synbiotics intake separ- ately or in combination with other drugs	8 weeks to 3 months	All RCTs	women with a definite diagnosis of Polycystic ovary syndrome (PCOS)	probiotics, prebiotics, synbiotics intake separately or in combination with other drugs versus placebo	SMD	random	0.321
BMI	-0.10 (-0.30 to 0.09)*	13	791	47.3									0.150
Waist circumference (WC)	0.37 (-0.78 to 1.53)*	5	316	95.5									No serious bias
hip circumference (HC)	-0.25 (-0.78 to 0.27)*	4	NP	76.9									
polymorphonuclear phagocytic capacity	0.74 (0.38 to 1.11)	3	NP	NP	MILLER L E 2017 [12]	Bifidobacterium (B.) animalis ssp. lactis HN019	3-6 weeks	randomized or non-randomized controlled studies	healthy, elderly (≥60 years) adults	consumption of B. lactis HN019 vs. control	SMD	random	NP
natural killer (NK) cell tumoricidal activity	0.43 (0.08 to 0.78)	3	NP	NP									
Polymorphonuclear cell phagocytic capacity	1.37 (0.86 to 1.88)	9	NP	80.0	Miller LE 2019[13]	probiotic consumption	3-12 weeks	prospective controlled studies	healthy older adults (mean age ≥ 60 years)	consumption of probiotic vs control	SMD	random	0.38
natural killer (NK) cell tumoricidal activity	0.55 (0.37 to 0.73)	15		43.0									0.51
No-recovery (incomplete resolution of	0.67(0.56 to 0.79)	10	574	48.0	DALAL R 2017 [14]	probiotics	follow-up: 1 month to 3 months	randomised clinical trials	people with any grade of acute or	probiotics in any dosage vs. placebo or no	RR	random	NP

clinical symptoms)									chronic hepatic encephalopathy	intervention, or with any other treatment in people with hepatic encephalopathy.			
Effects of Probiotics on Late-Onset Sepsis	0.88 (0.80 to 0.97)	28	4304/8535	23.0	DERMYSHI E 2017 [15]	enteral adminis- tration of probiotics	NP	All RCTs	very low birth weight (VLBW) (<1,500 g) preterm (<34 weeks gestational age) infants with enteral administration of probiotics initiated within 10 days	enteral administration of probiotics vs. Control group	RR	fixed	0.242
incidence of Late onset sepsis (LOS)	0.79 (0.71 to 0.88)	25	2934/5868	0.0	ACETI A 2017 [16]	any probiotic	NP	Randomized and quasi-randomized controlled trials	preterm infants (gestational age (GA) <37 weeks) received within one month of age any probiotic	any probiotic vs. placebo or no treatment	RR	fixed	No serious bias
	0.75 (0.65 to 0.86)	20	1705/3402	0.0					preterm infants (gestational age (GA) <37 weeks) (exclusively human-milk-fed)				
	0.77 (0.51 to 1.17)*	16	398/800	1.0					preterm infants (gestational age (GA) <37 weeks) (mixed feeding)				
Effect on body mass index	-0.39 (-0.49 to -0.29)	20	NP	97.9	HERNANDEZ-SAAVEDRA D 2018 [17]	Any prebiotic or probiotic treatment, or a combination of	NP	All RCTs	Male and female patients of any age that presented at	Vs. placebo	MD	random	No serious bias

	kg/m²					both (synbiotic)			least 1 of the following: NAFLD, steatosis, liver fibrosis, steatohepatitis				
Liver stiffness as measured by the fibro scan	-0.62 (-0.89 to -0.35)	4	217	83.0	KHAN M Y 2019[18]	probiotics and/or synbiotics	NP	All RCTs	adult patients older than 18 years of age with NAFLD or NASH	Vs. placebo	MD	random	NP
Infections Rate	0.21 (0.11 to 0.41)	4	246	1.0	SAWAS T 2015 [19]	probiotics	NP	controlled studies	Liver transplantation	Vs. placebo	RR	fixed	NP
urinary tract infection (UTI)	0.14 (0.04 to 0.47)	4	246	0.0									
intra-abdominal infection	0.27 (0.09 to 0.78)	4	246	0.0									
length of hospital stay	-1.41 (-1.97 to -0.86) days	3	196	0.0							MD		
duration of antibiotic use	-3.89 (-4.17 to -3.60) days	3	196	80.0									
Acute graft rejection	0.73 (0.44 to 1.22)*	3	179	0.0							RR		
postoperative infection rate	0.35 (0.24 to 0.50)	23	1696	47.0	YANG Z 2017 [20]	Probiotic or synbiotic	NP	All RCTs	perioperative patient	Vs. placebo or no probiotic inter- vention.	OR	random	0.006
pneumonia	0.44 (0.28 to 0.68)	16	1276	0.0									0.238
urinary tract infection	0.30 (0.16 to 0.55)	13	1151	0.0									0.666
wound infection	0.58 (0.42 to 0.80)	18	1723	0.0									0.097
intra-abdominal abscess	0.65 (0.28 to 1.55)	10	495	0.0									0.744
Postoperative mortality	1.19 (0.52 to 2.74)	8	1648	21.0									0.179
lengths of	-3.20	12	807	89.0							MD		0.094

hospital stay	(-4.87 to -1.54) days												
antibiotic therapy	-3.40 (-4.67 to -2.13)	11	922	92.0									0.954
ICU stay	-0.46 (-1.07 to 0.14)*	7	453	70.0									0.151
the incidence of surgical site infections (SSI)	0.72 (0.56 to 0.92)	13	1374	0.0	WU X D 2018 [21]	prophylactic probiotics in combination with antibiotics preoperatively	Preoperation 1-14 days, postoperation 0-14 days	All RCTs	adult patients who undergo colorectal surgery	Vs. placebo or other base ingredients in combination with antibiotics preoperatively	RR	random	No serious bias
pneumonia	0.50 (0.29 to 0.84)	10	807	0.0									
urinary tract infection	0.50 (025 to 0.98)	8	705	0.0									
cumulative duration of antibiotic therapy	-1.24 (-1.61 to -0.86) days	5	504	0.0									
length of stay	-1.16 (-1.91 to -0.41) days	7	514	0.0							MD		
prevention of bacterial translocation	0.75 (0.46 to 1.21)*	7	NP	NP	SKONIECZNA-ZYDECKA K 2018 [22]	treatment with pro-/pre-/synbiotics	Preoperation 0-15 days, postoperation 0-until discharge	All RCTs	surgical patients	Probiotics vs. No probiotics	SMD	random	NP
pneumonia	0.55 (0.38 to 0.80)	21		0.0							RR		
surgical site infection	0.55 (0.39 to 0.78)	10		7.89									
Abdominal distention	0.64 (0.47 to 0.86)	4		0.0									
Anastomotic leakage	0.73 (0.43 to 1.24)*	9		3.69									
Deep organ space	0.62 (0.31 to 1.25)*	5		0.0									
Diarrhea	0.49 (0.37, 0.67)	7		0.0									

Intraabdominal abscess	0.69 (0.35 to 1.37)*	8		0.0									
MRSA infection	0.34 (0.11 to 1.06)*	4		0.0									
Peritonitis	0.34 (0.09 to 1.32)*	4		0.0									
Reoperation	0.43 (0.14 to 1.29)*	3		0.0									
Sepsis	0.69 (0.58, 0.81)	7		0.0									
Superficial incisional	0.53 (0.38 to 0.74)	15		0.0									
UTI	0.32 (0.18 to 0.57)	13		0.0									
Blood loss	0.004 (-0.120 to 0.128)*	7		0.0									
Duration of antibiotic therapy	-0.60 (-1.09 to -0.10)	10		91.0							SMD		
Duration of postoperative pyrexia	-0.44 (-0.68 to -0.192）	4		38.1									
Fluid diet	-0.35 (-0.58 to -0.12)	3		0.0									
Hospital stay	-0.48 (-0.66, -0.30)	10		45.7									
ICU stay	-0.10 (-0.36, 0.16)*	4		0.0									
Operating time	0.02 (-0.14 to 0.18)*	11		49.0									
Solid diet	-0.31 (-0.50, -0.12)	4		0.0									
the incidence of small intestinal bacterial	0.54 (0.19 to 1.52)*	3	NP	83.7	ZHONG C Q 2017[23]	Probiotic	NP	RCTs, crossover trials,	Human	Probiotics vs. No probiotics	RR	random	NP

overgrowth (SIBO)								prospective single-arm trials and retrospective studies					
SIBO Decontamination Rate	1.61 (1.19 to 2.17)*	5		25.7							RR		
H_2 Volume in HBT	-36.35 (-44.23 to -28.47) ppm	4		0.0							WMD		
disease activity score in 28 joints (DAS28)	-0.11 (-0.47 to 0.24)*	3	132	87.0	PAN H D 2017[24]	probiotic	Follow up: 8-48 weeks	All RCTs	patients with a clear diagnosis of RA and stable treatment of disease-modifying anti-rheumatic drugs (DMARDs)	probiotics vs. Placebo or no probiotics	MD	random	NP
quality-of-life (QoL)	0.36 (0.07 to 0.64)	11	1501	86.0	Le Morvan De Sequeira 2021[25]	probiotic	4 weeks-6 months	All RCTs	adults of both sexes and of all ages with IBS	probiotics vs. Placebo	SMD	random	NP
seroprotection rate of H1N1 strain	1.83 (1.19 to 2.82)	6	386	0.0	LEI W T 2017[26]	probiotics, prebiotics, or synbiotics	NP	All RCTs	human adults	Probiotic vs. Control group	OR	random	0.37
seroconversion rate of H1N1 strain	0.52 (0.75 to 3.09)*	6	553	51.0									0.95
seroprotection rate of H3N2 strain	2.85 (1.59 to 5.10)	7	388	0.0									0.86
seroconversion rate of H3N2 strain	2.54 (0.93 to 6.91)*	6	553	83.0									0.76
seroprotection rate of B strain	0.99 (0.65 to 1.52)*	7	389	0.0									0.61
seroconversion rate of B strain	2.11 (1.38 to 3.21)	6	553	0.0									0.47
hemagglutination inhibition titers of A/H1N1 strain after influenza	7.14 (2.73 to 11.55)	12	688	96.0	YEH T L 2018[27]	probiotics or prebiotics	NP	Control trials	adults	probiotics or prebiotics vs. placebo	MD	random	No serious bias

vaccination													
hemagglutination inhibition titers of A/H3N2 strain after influenza vaccination	17.19 (3.39 to 30.99)	12	688	100.0									
hemagglutination inhibition titers of B strain after influenza vaccination	4.17 (0.37 to 7.96)	12	688	94.0									
estimated glomerular filtration rate preservation	2.10 (-1.31 to 5.52)*	4	212	68.0	TAO S 2019 [28]	probiotics (specified probiotic or probiotic mixes) in any form (e.g., capsule, tablet or other food supplements or components), the probiotics could be administered at any dose and for any duration	Follow up: 6 weeks - 6 months	All RCTs	the participants were diagnosed with CKD, whether undergoing dialysis or not	probiotics vs placebo	MD	random	0.051
total antioxidant capacity	0.42 (0.18 to 0.66)	5	NP	0.0	BAKHTIARY M 2021 [29]	probiotic, prebiotic, or synbiotic	Follow up: 4-12 weeks	All RCTs	chronic kidney disease (CKD) patients	Vs. usual care or placebo	SMD	random	NP
BMI	0.05 (-0.17 to 0.27)*	6		0.0									
eGFR, estimated glomerular filtration rate	0.34 (-2.20 to 2.89 mL/min/1.73 m^{2*}	3	132	0.0	MCFARLANE C 2019[30]	prebiotics, probiotics, or synbiotics for a duration of at least 1 week	NP	RCT including crossover, cluster, or quasi-RCT designs	participants with CKD as defined by the Kidney Disease Outcome Quality Initiative Guidelines	Vs. comparison groups	MD	random	NP
weight	0.40 (-0.95 to 1.74) kg*	7	357	0.0									
BMI	-0.06(-0.53 to 0.41) kg/m^{2*}	NP	NP	0.0	BOCK P M 2021[31]	any probiotic, prebiotic or synbiotic supplementation or combination of interventions with the aim of adjusting the gut	Follow up: 4-12 weeks	clinical trials	adult participants with type 1 or 2 diabetes diagnosis	Vs. Control group	MD	Random	No serious bias

						microbiota							
Systolic blood pressure	-0.93 (-1.58 to -0.27)	6	NP	89.6	HENDIJANI F 2018[32]	probiotics in the form of any pharmaceutical formulations or dairy 15 products	NP	(all) 10 randomized controlled trials (RCTs) and 1 single blinded trial and 2 crossover trials	adult diabetic patients	Vs. placebo	SMD	random	No serious bias
Diastolic blood pressure	-0.88 (-1.76 to -0.01)	7		94.0									
the risk of developing asthma	0.86 (0.73 to 1.01)*	14	4138	0.0	DU X Z 2019[33]	Probiotics were supplemented since the pre- and/or postnatal periods, with ? 3 months of intervention duration	Follow up: 1-15 y	All RCTs	healthy children, with or without a high risk of developing allergic diseases and without concurrent treatments	probiotics vs placebo	RR	fixed	No serious bias
Allergic rhinitis	1.03 (0.77 to 1.39)*	5	1897	68.0								random	
Wheeze	0.94 (0.81 to 1.08)*	8	1957	20.0								fixed	
Positive aeroallergen skin-prick test (SPT) result	0.74 (0.50 to 1.09)*	4	870	0.0									
systolic blood pressure	-2.05 (-3.87 to -0.24)	11	628	10.0	CHI C 2020[34]	Probiotics	3-24 weeks	All RCTs	hypertension patients	probiotics vs placebo	MD	random	0.68
diastolic blood pressure	-1.26 (-2.51 to -0.00)	12	688	0.0									0.44
BMI	-1.03 (-1.28 to -0.79)	8	398	0.0									0.27
Weight	-1.54 (-4.83 to 1.75) kg*	5	NP	0.0	HADI A 2021 [35]	probiotics or synbiotics	3-28 weeks	All RCTs	adults with metabolic syndrome	probiotics vs control group	MD	random	0.53
BMI	-0.06 (-0.56 to 0.44) kg/m^2*	7		0.0						probiotics vs placebo			0.31
WC	-1.33 (-3.30 to 0.64) cm*	6		28.9									0.92
eczema	0.59 (0.45	17	4011	69.0	SUN S Y 2021[36]	Probiotic started	NP	All RCTs	participants	Vs. placebo	OR	rando	NP

	to 0.78)					during pregnancy or within 12 months of age			should be high risk, defined as at least one parent or older sibling of the enrolling infants who had doctor-diagnosed allergic disease			m	
atopic eczema	0.66 (0.48 to 0.91)	6	1193	8.0									
asthma	0.88 (0.69 to 1.10)*	12	3209	15.0									
rhinitis	0.83 (0.55 to 1.24)*	5	1214	42.0									
wheeze	0.82 (0.67 to 1.01)*	8	1922	0.0									
allergic disease	0.83 (0.58 to 1.17)*	5	1556	44.0									
sensation	0.99 (0.75 to 1.30)*	7	1174	10.0									
Self-assessed eczema symptoms on a 20-point visual analogue scale of itch and sleep disturbance (SCORAD part C	-0.90 (-2.84 to 1.04)*	5	309	79.0	BOYLE R J 2009[37]	live orally ingested microorganisms, including bacteria, fungi or yeasts, ingested singly or in combination-organisms were presumed to be live unless the authors specified that they were killed	NP	All RCTs	participants of any age with doctor-diagnosed eczema	Vs. control group received no treatment, placebo or another non-microbial intervention	MD	random	NP
nvestigator-rated eczema severity (total SCORAD) at the end of treatment	-2.46 (-7.45 to 2.53)*	7	588	76.0									
Ventilator-associated pneumonia (VAP) Incidence	0.67 (0.59 to 0.77)	20	2428	11.3	JI T 2020[38]	probiotic	NP	All RCTs	adults who received mechanical ventilation (MV) therapy in hospitals	Vs. control	RR	fixed	0.273
length of ICU stay	-1.42 (-2.52 to -0.31)	15	1895	90.7							WMD	random	0.891
length of hospital stay	-1.79 (-3.89 to -0.31)	8	1161	86.3							WMD		NA
mortality rates	0.83 (0.70 to 1.02)*	15	1959	0.0							RR		0.301

days of MV	-1.06 (-2.54 to 0.43)*	12	1563	96.9							WMD		0.558
Days of using antibiotics	-2.28 (-4.02 to -0.55)	5	664	72.7									NP
the onset of VAP following intubation	2.09 (-0.15 to 4.32)*	4	265	92.7									
incidence of bacteremia	0.51 (0.35 to 0.76)	6	688	39.8							RR	Random	
rate of multidrug resistant (MDR) infections	0.64 (0.52 to 0.79)	9	1487	45.3								fixed	
Symptom scores	-0.09 (-0.44 to 0.26)*	3	251	46.0	WANG F Y 2021[39]	probiotics or prebiotics	NP	All RCTs	human	Vs. placebo	SMD	random	NP
Infection rate	0.80 (0.64 to 1.01)*	5	286	73.0							RR	random	
the number of subjects having at least 1 RTI episode	0.89 (0.82 to 0.96)	17	4513	82.0	WANG Y Z 2016[40]	probiotic	5 days to 2years	All RCTs	healthy children (from birth to 18 years), who developed upper respiratory tract infections (URTIs) or lower respiratory tract infections (LRTIs) at some point during the study	Vs. placebo	RR	random	NP
the duration of RTI illness episodes	-0.60 (-1.49 to 0.30)*	9	2817	88.0							MD	random	
the number of days of RTI illness	-0.16 (-0.29 to -0.02)	6	2067	0.0									
the days absent from day care/school	-0.94 (-1.72 to -0.15)	8	1499	87.0									
ΔPittsburgh Sleep Quality Index (PSQI) score	0.78 (0.40 to 1.17)*	11	452	0.0	IRWIN C 2020 [41]	administered live bacteria (probiotics) or heat-inactivated/killed probiotic bacteria (paraprobiotics) as a daily oral supplement (e.g.,	NP	randomized controlled trials or quasi-experimental (nonrandomized controlled trials)	adults >18 years of age	Vs. placebo	MD	random	NP
sleep efficiency	0.02 (-1.21 to 1.24)*	6	453	0.0						change from baseline	MD		
sleep latency	0.69 (-1.36	7	467	0.0							MD		

	to 0.51)*					capsules/tablets, fermented milk, yoghurt,powder sachets)							
score derived from other subjective ratings scales	0.08 (-0.14 to 0.30)*	8	549	36.0							SMD		
EDSS scores	-1.22 (-2.40 to -0.03)	3	173	92.0	JIANG J 2021[42]	probiotic	NP	All RCTs	Adult human participants with ages between 18 and 55 with clinically definite relapsing-remitting multiple sclerosis (RRMS), diagnosed according to McDonald criteria and an expanded disability status scale (EDSS) score ≤4.5	probiotics vs. placebos	SMD	random	NP
BDI total scores	-1.58 (-3.03 to -0.12)	3	173	94.0									
GHQ scores	-0.71 (-1.02 to -0.40)	3	173	0.0									
Physical Growth During the First Year of Life	1.07 (0.14 to 1.99) g	4	436	0.0	RAO S 2009[43]	combination of prebiotics and probiotics vs controls	NP	All RCTs	full-term neonates	formula had different composition vs. the control formula (apart from prebiotics)	WMD	fixed	No serious bias
Incidence of infections requiring antibiotic therapy	0.68 (0.61 to 0.77)	3	1122	0.0	LOHNER S 2014[44]	prebiotics (prebiotics added to food in the manufacturing process or as a separate supplement)	a supplementation time of at least 2 months and an observation time of at least 4 months	All RCTs	healthy infants or children, aged 0–18 years	Vs. control (placebo or no supplementation)	RR	random	NP
QoL, quality of life	0.06 (-0.14 to 0.25)	4	322	0.0	WILSON B 2019[45]	prebiotics	2-12 weeks	All RCTs	Adult patients ≥18 and ≤64 y with Irritable bowel syndrome (IBS) or other Functional bowel	vs. placebo	SMD	random	No serious bias

									disorders (FBDs)				
Effects on sepsis	0.64 (0.51 to 0.78)	11	1106	0.0	CHI C 2019[46]	prebiotics	7-57 days	All RCTs	low birth weight infants (<2500 g) or preterm infants (<36 weeks)	vs. placebo	RR	random	0.80
length of hospital stay	-5.18 (-8.94 to -1.41)	8	733	83.0									0.08
BMI	-0.49 (-0.86 to -0.13)	4	NP	41.3	STACHOWSKA E 2020[47]	treatment with prebiotic (soluble fiber or insoluble fiber) in the form of a supplement, e.g., pills, powder, etc.	NP	All RCTs	patients with confirmed non-alcoholic steatohepatitis (NASH)	Vs. control	SMD	random	0.83
liver stiffness measurement (LSM)	-0.70 (-1.00 to -0.40) kpa	4	235	93.4	SHARPTON S R 2019[48]	interventions in any of the following 4 categories: antibiotics, probiotics, synbiotics, or fecal microbiota transplantation	NP	All RCTs	patients with nonalcoholic fatty liver disease (NAFLD)	Vs. placebo, usual care, or no intervention in patients with NAFLD	WMD	random	NP
improvement in hepatic steatosis, as graded by ultrasound	2.40 (1.50 to 3.84)	6	384	22.4							OR		
SCORAD values at 8 weeks	-6.56 (-11.43 to -1.68)	6	NP	77.1	CHANG Y 2016[49]	synbiotics	NP	All RCTs	patients with atopic dermatitis (AD)	Vs. control	WMD	random	NP
Quality of Life	-0.14(-0.45 to 0.17)*	3	159	0.0	ZHU H F 2020[50]	treated with microecological preparations (probiotics, prebiotics, synbiotics, or a combination of two preparations)	15 days to 6months	All RCTs	obese adults (age ≥18 years) who had undergone bariatric surgery (a type of surgery was not restricted)	Vs. placebo or conventional treatment	SMD	fixed	NP
Excess Weight Loss	0.45 (-0.16 to 1.05)*	4	154	68.0								random	
Infection Rates	0.46 (0.31 to 0.67)	11	913	43.0	KAHN J 2020[51]	pro-/synbiotics perioperatively	NP	Randomized and non-randomized control studies	Patients accepted liver resection (LR) or liver transplantation (LT).	vs. controls	RR	random	NP
the number of participants who experienced ≥1	0.84 (0.73 to 0.96)	9	2845	43.8	CHAN C K Y 2020[52]	Synbiotics were all administered through oral ingestion and	7 days to 12 months	randomized controlled trials (RCTs) and	individuals of any age (including full-term	Vs. without Synbiotics	Rate ratio	random	0.20

Respiratory Tract Infections (RTI) episode						composed of ≥1 probiotic bacterial strain plus ≥1 type of prebiotics in different forms, such as powdered milk, yogurt, or capsules		placebo-controlled trials	infants) with no pretrial symptoms				
the proportion of participants experiencing RTIs	0.84 (0.74 to 0.95)	7	7273	47.7							Risk ratio		0.55
the proportion of infants who had infections	0.35 (0.19 to 0.67)	3	NP	0.0	SORENSEN K 2021[53]	any amino acid formula (AAF) with synbiotics (AAF-Syn) or extensively hydrolysed formula (eHF) with synbiotics (eHF-Syn), Studies of infant formulae containing cow's milk protein, and eHF or AAF without synbiotics (including those with only a prebiotic or a probiotic) were excluded	7 days to 12 months	All RCTs	infants and children aged <3 years with IgE or non IgE mediated Cow's milk protein allergy (CMPA)	AAF-Syn than with AAF alone	OR	fixed	NP
septic complications	0.42 (0.23 to 0.75)	5	NP	NP	Kinross JM 2013[54]	prebiotic,	NP	All RCTs	patients underwent abdominal surgery with the use of a prebi- otic, probiotic, or a synbiotic agent	Vs. either received a placebo or received no therapy	OR	random	0.25
	0.25 (0.10 to 0.60)	9				synbiotic							0.08
Postoperative Pneumonia	0.38 (0.07 to 2.03)	3				prebiotic,							NA
	0.89 (0.31 to 2.55)	7				synbiotic							0.14
Wound Infection	0.73 (0.29 to 1.81)	3				prebiotic,							NA
	0.48 (0.19 to 1.20)	7				synbiotic							0.14
Urinary Tract Infection	0.54 (0.03 to 10.73)	3				prebiotic,							NA
	0.29 (0.07	6				synbiotic							0.14

	to1.16)												
Length of Hospital Stay	-0.07(-0.55 to 0.42)	7				synbiotic					WMD		0.15
Length of Antibiotic Treatment	-1.71(-3.2 to-0.21)	4				synbiotic							0.02
Effect of probiotics on development of overt HE (week 4)	0.22 (0.07 to 0.67)	3	95/191	0.0	CAO Q 2018 [55]	Probiotic treatment	4-12 weeks	All RCTs	Patients with Minimal hepatic encephalopathy (MHE)	Probiotic treatment vs placebo or no treatment	OR	random	NP
Effect of probiotics on development of overt HE (week 12)	0.48 (0.21 to 1.12) *	3	104/205	0.0									
Improvement in Minimal hepatic encephalopathy (MHE) (week 12)	0.15 (0.07 to 0.32)	3	104/205	0.0									

* No statistical significance; CI, confidence interval; RCT, randomized controlled trial; RR, relative risk; HR, hazard ratio; MD, mean difference; SMD, standard mean difference; WMD, weighted mean difference; OR, odds ratio; NA, not available; NP, not published.

References

1. Goldenberg, J.Z.; Yap, C.; Lytvyn, L.; Lo, C.K.; Beardsley, J.; Mertz, D.; Johnston, B.C. Probiotics for the prevention of Clostridium difficile-associated diarrhea in adults and children. *Cochrane Db. Syst. Rev.* **2017**, *2017*, D6095, doi: 10.1002/14651858.CD006095.pub4.
2. Chen, X.; Hu, Y.; Yuan, X.; Yang, J.; Ka, L. Effect of early enteral nutrition combined with probiotics in patients with stroke: a meta-analysis of randomized controlled trials. *Eur. J. Clin. Nutr.* **2022**, *76*, 592-603, doi: 10.1038/s41430-021-00986-3.
3. Wang, Z.; Xin, S.; Ding, L.; Ding, W.; Hou, Y.; Liu, C.; Zhang, X. The Potential Role of Probiotics in Controlling Overweight/Obesity and Associated Metabolic Parameters in Adults: A Systematic Review and Meta-Analysis. *Evid.-Based Compl. Alt.* **2019**, *2019*, 1-14, doi: 10.1155/2019/3862971.

4. Suzumura, E.A.; Bersch-Ferreira, Â.C.; Torreglosa, C.R.; Da Silva, J.T.; Coqueiro, A.Y.; Kuntz, M.G.F.; Chrispim, P.P.; Weber, B.; Cavalcanti, A.B. Effects of oral supplementation with probiotics or synbiotics in overweight and obese adults: a systematic review and meta-analyses of randomized trials. *Nutr. Rev.* **2019**, *77*, 430-450, doi: 10.1093/nutrit/nuz001.
5. Mohammadi, H.; Ghavami, A.; Faghihimani, Z.; Sharifi, S.; Nattagh-Eshtivani, E.; Ziaei, R.; Miraghajani, M. Effects of probiotics fermented milk products on obesity measure among adults: A systematic review and meta-analysis of clinical trials. *J. Funct. Foods* **2021**, *82*, 104494, doi: 10.1016/j.jff.2021.104494.
6. Hadi, A.; Ghaedi, E.; Khalesi, S.; Pourmasoumi, M.; Arab, A. Effects of synbiotic consumption on lipid profile: a systematic review and meta-analysis of randomized controlled clinical trials. *Eur. J. Nutr.* **2020**, *59*, 2857-2874, doi: 10.1007/s00394-020-02248-7.
7. Pontes, K.S.D.S.; Guedes, M.R.; Cunha, M.R.D.; Mattos, S.D.S.; Barreto Silva, M.I.; Neves, M.F.; Marques, B.C.A.A.; Klein, M.R.S.T. Effects of probiotics on body adiposity and cardiovascular risk markers in individuals with overweight and obesity: A systematic review and meta-analysis of randomized controlled trials. *Clin. Nutr.* **2021**, *40*, 4915-4931, doi: 10.1016/j.clnu.2021.06.023.
8. Zhang, G.; Hu, H.; Liu, C.; Zhang, Q.; Shakya, S.; Li, Z. Probiotics for Prevention of Atopy and Food Hypersensitivity in Early Childhood. *Medicine* **2016**, *95*, e2562, doi: 10.1097/MD.0000000000002562.
9. Coffey, M.J.; Garg, M.; Homaira, N.; Jaffe, A.; Ooi, C.Y. Probiotics for people with cystic fibrosis. *Cochrane Db. Syst. Rev.* **2020**, doi: 10.1002/14651858.CD012949.pub2.
10. Cozzolino, M.; Vitagliano, A.; Pellegrini, L.; Chiurazzi, M.; Andriasani, A.; Ambrosini, G.; Garrido, N. Therapy with probiotics and synbiotics for polycystic ovarian syndrome: a systematic review and meta-analysis. *Eur. J. Nutr.* **2020**, *59*, 2841-2856, doi: 10.1007/s00394-020-02233-0.
11. Li, Y.; Tan, Y.; Xia, G.; Shuai, J. Effects of probiotics, prebiotics, and synbiotics on polycystic ovary syndrome: a systematic review and meta-analysis. *Crit. Rev. Food Sci.* **2021**, 1-17, doi: 10.1080/10408398.2021.1951155.
12. Miller, L.; Lehtoranta, L.; Lehtinen, M. The Effect of Bifidobacterium animalis ssp. lactis HN019 on Cellular Immune Function in Healthy Elderly Subjects: Systematic Review and Meta-Analysis. *Nutrients* **2017**, *9*, 191, doi: 10.3390/nu9030191.
13. Miller, L.E.; Lehtoranta, L.; Lehtinen, M.J. Short-term probiotic supplementation enhances cellular immune function in healthy elderly: systematic review and meta-analysis of controlled studies. *Nutr. Res.* **2019**, *64*, 1-8, doi: 10.1016/j.nutres.2018.12.011.
14. Dalal, R.; Mcgee, R.G.; Riordan, S.M.; Webster, A.C. Probiotics for people with hepatic encephalopathy. *Cochrane Db. Syst. Rev.* **2021**, *2021*, doi: 10.1002/14651858.CD008716.pub3.
15. Dermyshi, E.; Wang, Y.; Yan, C.; Hong, W.; Qiu, G.; Gong, X.; Zhang, T. The "Golden Age" of Probiotics: A Systematic Review and Meta-Analysis of Randomized and Observational Studies in Preterm Infants. *Neonatology* **2017**, *112*, 9-23, doi: 10.1159/000454668.
16. Aceti, A.; Maggio, L.; Beghetti, I.; Gori, D.; Barone, G.; Callegari, M.; Fantini, M.; Indrio, F.; Meneghin, F.; Morelli, L.; et al. Probiotics Prevent Late-Onset Sepsis in Human Milk-Fed, Very Low Birth Weight Preterm Infants: Systematic Review and Meta-Analysis. *Nutrients* **2017**, *9*, 904, doi: 10.3390/nu9080904.
17. Loman, B.R.; Hernández-Saavedra, D.; An, R.; Rector, R.S. Prebiotic and probiotic treatment of nonalcoholic fatty liver disease: a systematic review and meta-analysis. *Nutr. Rev.* **2018**, *76*, 822-839, doi: 10.1093/nutrit/nuy031.
18. Khan, M.Y.; Mihali, A.B.; Rawala, M.S.; Aslam, A.; Siddiqui, W.J. The promising role of probiotic and synbiotic therapy in aminotransferase levels and inflammatory markers in patients with nonalcoholic fatty liver disease – a systematic review and meta-analysis. *Eur. J. Gastroen. Hepat.* **2019**, *31*, 703-715, doi: 10.1097/MEG.0000000000001371.

19. Sawas, T.; Al Halabi, S.; Hernaez, R.; Carey, W.D.; Cho, W.K. Patients Receiving Prebiotics and Probiotics Before Liver Transplantation Develop Fewer Infections Than Controls: A Systematic Review and Meta-Analysis. *Clin. Gastroenterol. H.* **2015**, *13*, 1567-1574, doi: 10.1016/j.cgh.2015.05.027.
20. Yang, Z.; Wu, Q.; Liu, Y.; Fan, D. Effect of Perioperative Probiotics and Synbiotics on Postoperative Infections After Gastrointestinal Surgery: A Systematic Review With Meta-Analysis. *Jpen.-Parenter. Enter.* **2017**, *41*, 1051-1062, doi: 10.1177/0148607116629670.
21. Wu, X.; Xu, W.; Liu, M.; Hu, K.; Sun, Y.; Yang, X.; Zhu, G.; Wang, Z.; Huang, W. Efficacy of prophylactic probiotics in combination with antibiotics versus antibiotics alone for colorectal surgery: A meta-analysis of randomized controlled trials. *J. Surg. Oncol.* **2018**, *117*, 1394-1404, doi: 10.1002/jso.25038.
22. Skonieczna-żydecka, K.; Kaczmarczyk, M.; łoniewski, I.; Lara, L.; Koulaouzidis, A.; Misera, A.; Maciejewska, D.; Marlicz, W. A Systematic Review, Meta-Analysis, and Meta-Regression Evaluating the Efficacy and Mechanisms of Action of Probiotics and Synbiotics in the Prevention of Surgical Site Infections and Surgery-Related Complications. *Journal of Clinical Medicine* **2018**, *7*, 556, doi: 10.3390/jcm7120556.
23. Zhong, C.; Qu, C.; Wang, B.; Liang, S.; Zeng, B. Probiotics for Preventing and Treating Small Intestinal Bacterial Overgrowth. *J. Clin. Gastroenterol.* **2017**, *51*, 300-311, doi: 10.1097/MCG.0000000000000814.
24. Pan, H.; Li, R.; Li, T.; Wang, J.; Liu, L. Whether Probiotic Supplementation Benefits Rheumatoid Arthritis Patients: A Systematic Review and Meta-Analysis. *Engineering* **2017**, *3*, 115-121, doi: 10.1016/J.ENG.2017.01.006.
25. Le Morvan De Sequeira, C.; Kaeber, M.; Cekin, S.E.; Enck, P.; Mack, I. The Effect of Probiotics on Quality of Life, Depression and Anxiety in Patients with Irritable Bowel Syndrome: A Systematic Review and Meta-Analysis. *Journal of Clinical Medicine* **2021**, *10*, 3497, doi: 10.3390/jcm10163497.
26. Lei, W.; Shih, P.; Liu, S.; Lin, C.; Yeh, T. Effect of Probiotics and Prebiotics on Immune Response to Influenza Vaccination in Adults: A Systematic Review and Meta-Analysis of Randomized Controlled Trials. *Nutrients* **2017**, *9*, 1175, doi: 10.3390/nu9111175.
27. Yeh, T.; Shih, P.; Liu, S.; Lin, C.; Liu, J.; Lei, W.; Lin, C. The influence of prebiotic or probiotic supplementation on antibody titers after influenza vaccination: a systematic review and meta-analysis of randomized controlled trials. *Drug Des. Dev. Ther.* **2018**, *Volume 12*, 217-230, doi: 10.2147/DDDT.S155110.
28. Tao, S.B.; Tao, S.Y.; Cheng, Y.M.; Liu, J.; Ma, L.; Fu, P. Effects of probiotic supplements on the progression of chronic kidney disease: A meta-analysis. *Nephrology* **2019**, *24*, 1122-1130, doi: 10.1111/nep.13549.
29. Bakhtiary, M.; Morvaridzadeh, M.; Agah, S.; Rahimlou, M.; Christopher, E.; Zadro, J.R.; Heshmati, J. Effect of Probiotic, Prebiotic, and Synbiotic Supplementation on Cardiometabolic and Oxidative Stress Parameters in Patients With Chronic Kidney Disease: A Systematic Review and Meta-analysis. *Clin. Ther.* **2021**, *43*, e71-e96, doi: 10.1016/j.clinthera.2020.12.021.
30. Mcfarlane, C.; Ramos, C.I.; Johnson, D.W.; Campbell, K.L. Prebiotic, Probiotic, and Synbiotic Supplementation in Chronic Kidney Disease: A Systematic Review and Meta-analysis. *J. Renal Nutr.* **2019**, *29*, 209-220, doi: 10.1053/j.jrn.2018.08.008.
31. Bock, P.M.; Telo, G.H.; Ramalho, R.; Sbaraini, M.; Leivas, G.; Martins, A.F.; Schaan, B.D. The effect of probiotics, prebiotics or synbiotics on metabolic outcomes in individuals with diabetes: a systematic review and meta-analysis. *Diabetologia* **2021**, *64*, 26-41, doi: 10.1007/s00125-020-05295-1.
32. Hendijani, F.; Akbari, V. Probiotic supplementation for management of cardiovascular risk factors in adults with type II diabetes: A systematic review and meta-analysis. *Clin. Nutr.* **2018**, *37*, 532-541, doi: 10.1016/j.clnu.2017.02.015.
33. Du, X.; Wang, L.; Wu, S.; Yuan, L.; Tang, S.; Xiang, Y.; Qu, X.; Liu, H.; Qin, X.; Liu, C. Efficacy of probiotic supplementary therapy for asthma, allergic rhinitis, and wheeze: a meta-analysis of randomized controlled trials. *Allergy Asthma Proc.* **2019**, *40*, 250-260, doi: 10.2500/aap.2019.40.4227.

34. Chi, C.; Li, C.; Wu, D.; Buys, N.; Wang, W.; Fan, H.; Sun, J. Effects of Probiotics on Patients with Hypertension: a Systematic Review and Meta-Analysis. *Curr. Hypertens. Rep.* **2020**, *22*, doi: 10.1007/s11906-020-01042-4.
35. Hadi, A.; Arab, A.; Khalesi, S.; Rafie, N.; Kafeshani, M.; Kazemi, M. Effects of probiotic supplementation on anthropometric and metabolic characteristics in adults with metabolic syndrome: A systematic review and meta-analysis of randomized clinical trials. *Clin. Nutr.* **2021**, *40*, 4662-4673, doi: 10.1016/j.clnu.2021.05.027.
36. Sun, S.; Chang, G.; Zhang, L. The prevention effect of probiotics against eczema in children: an update systematic review and meta-analysis. *J. Dermatol. Treat.* **2021**, 1-11, doi: 10.1080/09546634.2021.1925077.
37. Boyle, R.J.; Bath-Hextall, F.J.; Leonardi-Bee, J.; Murrell, D.F.; Tang, M.L. Probiotics for the treatment of eczema: a systematic review. *Clinical & Experimental Allergy* **2009**, *39*, 1117-1127, doi: 10.1111/j.1365-2222.2009.03305.x.
38. Ji, T.; Zhu, X.; Shang, F.; Zhang, X. Preventive Effect of Probiotics on Ventilator-Associated Pneumonia: A Meta-analysis of 2428 Patients. *Ann. Pharmacother.* **2021**, *55*, 949-962, doi: 10.1177/1060028020983021.
39. Wang, F.; Pan, B.; Xu, S.; Xu, Z.; Zhang, T.; Zhang, Q.; Bao, Y.; Wang, Y.; Zhang, J.; Xu, C.; et al. A meta-analysis reveals the effectiveness of probiotics and prebiotics against respiratory viral infection. *Bioscience Rep.* **2021**, *41*, R20203638, doi: 10.1042/BSR20203638.
40. Wang, Y.; Li, X.; Ge, T.; Xiao, Y.; Liao, Y.; Cui, Y.; Zhang, Y.; Ho, W.; Yu, G.; Zhang, T. Probiotics for prevention and treatment of respiratory tract infections in children. *Medicine* **2016**, *95*, e4509, doi: 10.1097/MD.0000000000004509.
41. Irwin, C.; Mccartney, D.; Ben, D.; Khalesi, S. Effects of probiotics and paraprobiotics on subjective and objective sleep metrics: a systematic review and meta-analysis. *Eur. J. Clin. Nutr.* **2020**, *74*, 1536-1549, doi: 10.1038/s41430-020-0656-x.
42. Jiang, J.; Chu, C.; Wu, C.; Wang, C.; Zhang, C.; Li, T.; Zhai, Q.; Yu, L.; Tian, F.; Chen, W. Efficacy of probiotics in multiple sclerosis: a systematic review of preclinical trials and meta-analysis of randomized controlled trials. *Food Funct.* **2021**, *12*, 2354-2377, doi: 10.1039/D0FO03203D.
43. Rao, S.; Srinivasjois, R.; Patole, S. Prebiotic Supplementation in Full-term Neonates. *Archives of Pediatrics & Adolescent Medicine* **2009**, *163*, 755-764, doi: 10.1001/archpediatrics.2009.94.
44. Lohner, S.; Küllenberg, D.; Antes, G.; Decsi, T.; Meerpohl, J.J. Prebiotics in healthy infants and children for prevention of acute infectious diseases: a systematic review and meta-analysis. *Nutr. Rev.* **2014**, *72*, 523-531, doi: 10.1111/nure.12117.
45. Wilson, B.; Rossi, M.; Dimidi, E.; Whelan, K. Prebiotics in irritable bowel syndrome and other functional bowel disorders in adults: a systematic review and meta-analysis of randomized controlled trials. *The American Journal of Clinical Nutrition* **2019**, *109*, 1098-1111, doi: 10.1093/ajcn/nqy376.
46. Chi, C.; Buys, N.; Li, C.; Sun, J.; Yin, C. Effects of prebiotics on sepsis, necrotizing enterocolitis, mortality, feeding intolerance, time to full enteral feeding, length of hospital stay, and stool frequency in preterm infants: a meta-analysis. *Eur. J. Clin. Nutr.* **2019**, *73*, 657-670, doi: 10.1038/s41430-018-0377-6.
47. Stachowska, E.; Portincasa, P.; Jamioł-Milc, D.; Maciejewska-Markiewicz, D.; Skonieczna-żydecka, K. The Relationship between Prebiotic Supplementation and Anthropometric and Biochemical Parameters in Patients with NAFLD—A Systematic Review and Meta-Analysis of Randomized Controlled Trials. *Nutrients* **2020**, *12*, 3460, doi: 10.3390/nu12113460.
48. Sharpton, S.R.; Maraj, B.; Harding-Theobald, E.; Vittinghoff, E.; Terrault, N.A. Gut microbiome–targeted therapies in nonalcoholic fatty liver disease: a systematic review, meta-analysis, and meta-regression. *The American Journal of Clinical Nutrition* **2019**, *110*, 139-149, doi: 10.1093/ajcn/nqz042.
49. Chang, Y.; Trivedi, M.K.; Jha, A.; Lin, Y.; Dimaano, L.; García-Romero, M.T. Synbiotics for Prevention and Treatment of Atopic Dermatitis. *JAMA Pediatr.* **2016**, *170*, 236, doi: 10.1001/jamapediatrics.2015.3943.

50. Zhu, H.; Ren, Z.; Zang, Y.; Hua, H.; Lu, J.; Xu, Q.; Zhu, S. Effects of Microecological Preparations on Obese Patients after Bariatric Surgery: A Systematic Review and Meta-Analysis. *Evid.-Based Compl. Alt.* **2020**, *2020*, 1-10, doi: 10.1155/2020/8724546.
51. Kahn, J.; Pregartner, G.; Schemmer, P. Effects of both Pro- and Synbiotics in Liver Surgery and Transplantation with Special Focus on the Gut–Liver Axis—A Systematic Review and Meta-Analysis. *Nutrients* **2020**, *12*, 2461, doi: 10.3390/nu12082461.
52. Chan, C.K.Y.; Tao, J.; Chan, O.S.; Li, H.; Pang, H. Preventing Respiratory Tract Infections by Synbiotic Interventions: A Systematic Review and Meta-Analysis of Randomized Controlled Trials. *Adv. Nutr.* **2020**, *11*, 979-988, doi: 10.1093/advances/nmaa003.
53. Sorensen, K.; Cawood, A.L.; Gibson, G.R.; Cooke, L.H.; Stratton, R.J. Amino Acid Formula Containing Synbiotics in Infants with Cow's Milk Protein Allergy: A Systematic Review and Meta-Analysis. *Nutrients* **2021**, *13*, 935, doi: 10.3390/nu13030935.
54. Kinross, J.M.; Markar, S.; Karthikesalingam, A.; Chow, A.; Penney, N.; Silk, D.; Darzi, A. A Meta-Analysis of Probiotic and Synbiotic Use in Elective Surgery. *Jpen.-Parenter. Enter.* **2013**, *37*, 243-253, doi: 10.1177/0148607112452306.
55. Cao, Q.; Yu, C.; Yang, S.; Cao, H.; Chen, P.; Deng, M.; Li, L. Effect of probiotic treatment on cirrhotic patients with minimal hepatic encephalopathy: A meta-analysis. *Hepatob. Pancreat. Dis.* **2018**, *17*, 9-16, doi: 10.1016/j.hbpd.2018.01.005.
