# Supplementary material for: Gastrointestinal Microbiome and Multiple Health Outcomes: Umbrella Review
Source: Nutrients. 2022 Sep 9;14(18):3726. doi: 10.3390/nu14183726 (PMC9505003; doi:10.3390/nu14183726)
Supplement: Supplementary file 1 [file nutrients-14-03726-s001.zip › Supplementary Table S8.pdf]

Supplement Table S8. Prebiotics/probiotics/synbiotics and side effects.

outcome(s) of interest	estimated summary effect (95% CI)	number of studies / total studies	number of intervention group or total participants	Heterogeneity (I^2, %)	the first author + year of publication	Intervention	Duration of Intervention/follow-up	study design	populations	outcome comparison	meta-analysis metric	type of effect model	publication bias
Incidence of adverse events	0.83 (0.71 to 0.97)	32	4329/8305	49.0	GOLDENBERG J Z 2017[1]	probiotics	NP	All RCTs	participants taking antibiotics	any versus none	RR	random	No serious bias
Adverse Events	0.97 (0.86 to 1.09)*	15	6431	NP	SHEN N T 2017[2]	probiotics	NP	All RCTs	Hospitalized adults (18 years or older) receiving antibiotic therapy (intravenous, oral, or both) for any reason	any versus none	RR	random	NP
Incidence of Adverse Events	0.82(0.65 to 1.05)*	17	166/1783	18.0	JOHNSTON B C 2012[3]	any dose of a specified probiotic of any strain	NP	All RCTs	adult or pediatric patients treated with antibiotics	any versus none	RR	random	No serious bias
the side effects observed in EcN treatment.	1.44(0.80 to 2.59)*	6	719	48.0	LOSURDO G 2015[4]	Escherichia coli Nissle (EcN) 1917	NP	All RCTs	Patients with UC	EcN versus standard of care (mesalazine or ciprofloxacin)<br><br>Regimen or placebo	OR	Random	No serious bias
Adverse events	0.00 (-0.01 to 0.01)*	24	4415	75.0	GUO Q 2019 [5]	specific, identified probiotic in any form (e.g. capsule, sachet, yogurt). Trials investigating non-specific probiotic or yogurt agents (e.g. products that do not label the	5 days to 4 weeks	All RCTs	children (0 to 18 years) receiving antibiotics	Probiotic Versus Control	RD(RD: Risk difference)	Random	No serious bias

						probiotic strain and dose) were not considered							
Incidence of Adverse Events	0.00 (-0.02 to 0.02)*	7	1183/2363	66.0	BLAABJERG S 2017[6]	Probiotics	NA	All RCTs	outpatients taking oral antibiotics	probiotics vs. Placebo or no treatment	RD (risk difference)	random	NP
Adverse events - Overt hepatic encephalopathy	0.29(0.16 to 0.51)*	10	585	0.0	DALAL R 2017 [7]	probiotics	follow-up: 2 weeks to 3 months	randomised clinical trials	people with any grade of acute or chronic hepatic encephalopathy	probiotics in any dosage vs. placebo or no intervention, or with any other treatment in people with hepatic encephalopathy.	RR	random	NP
Adverse events - Change of/or withdrawal from treatment	0.70(0.46 to 1.07)*	9	551	11.0			follow-up: 1 month to 3 months						
any adverse event	1.09 (0.91 to 1.29)*	36	4183	36.0	FORD A C 2018[8]	probiotics	Minimum treatment duration of 7 days<br>Minimum follow-up duration of 7 days	All RCTs	Adults (participants aged >16 years)	probiotics vs. Control group	RR	random	0.08
Adverse events in active UC	1.21 (0.64 to 2.27)*	6	281/507	-	DERWA Y 2017 [9]	probiotics	NP	All RCTs	Adults (>90% of patients aged >16 years) with inflammatory bowel disease	probiotics vs. placebo	RR	random	NA
Adverse events in quiescent UC	1.09 (0.71 to 1.67)*	3	277/555	-						Probiotics vs. 5-ASA			
Adverse events in CD in remission following a surgical resection	0.81 (0.61 tp 1.08)*	3	129/263	-						probiotics vs. placebo			
the incidence of adverse effect	0.80 (0.43 to 1.50)*	10	762	0.0	YEH T L 2018[10]	probiotics or prebiotics	NP	Control trials	adults	probiotics or prebiotics vs. placebo	OR	random	No serious bias
Number of adverse events, dichotomous outcome	-0.00 (-0.03 to 0.03)*	6	279	0.0	SEILER C L 2020[11]	Probiotics of any type and dose, administered orally in any form (drink/<br>powder/capsule	at least 2 weeks	All RCTs	Patients with Celiac disease (CD)	probiotics vs placebo	MD	random	NP

							)							
Adverse events	0.77 (0.47 to 1.26)*	4	355	0.0	WILSON 2019[12]	B	prebiotics	2-12 weeks	All RCTs	Adult patients ≥18 and ≤64 y with Irritable bowel syndrome (IBS) or other Functional bowel disorders (FBDs)	vs. placebo	OR	random	No serious bias
Adverse events	0.27 (0.07 to 1.05)*	5	409	0.0	Zhang 2020[13]	J	prebiotics, probiotics, and synbiotics	At least 7 days	All RCTs	adult patients (over the age of 16 years) with functional dyspepsia (FD)	Vs. placebo	RR	fixed	NP
adverse events	0.72 (0.55 to 0.94)	30	2430	NP	Sohail 2018[14]	G	probiotic Medilac-S	4-96 weeks	All RCTs	human	Vs. control	RR	fixed	No serious bias

* No statistical significance; CI, confidence interval; RCT, randomized controlled trial; RR, relative risk; HR, hazard ratio; MD, mean difference; SMD, standard mean difference; WMD, weighted mean difference; OR, odds ratio; NA, not available; NP, not published.

References

1. Goldenberg, J.Z.; Yap, C.; Lytvyn, L.; Lo, C.K.; Beardsley, J.; Mertz, D.; Johnston, B.C. Probiotics for the prevention of Clostridium difficile-associated diarrhea in adults and children. *Cochrane Db. Syst. Rev.* **2017**, *2017*, D6095, doi: 10.1002/14651858.CD006095.pub4.
2. Shen, N.T.; Maw, A.; Tmanova, L.L.; Pino, A.; Ancy, K.; Crawford, C.V.; Simon, M.S.; Evans, A.T. Timely Use of Probiotics in Hospitalized Adults Prevents Clostridium difficile Infection: A Systematic Review With Meta-Regression Analysis. *Gastroenterology* **2017**, *152*, 1889-1900, doi: 10.1053/j.gastro.2017.02.003.
3. Johnston, B.C.; Ma, S.S.Y.; Goldenberg, J.Z.; Thorlund, K.; Vandvik, P.O.; Loeb, M.; Guyatt, G.H. Probiotics for the Prevention ofClostridium difficile – Associated Diarrhea. *Ann. Intern. Med.* **2012**, *157*, 878, doi: 10.7326/0003-4819-157-12-201212180-00563.
4. Losurdo, G.; Iannone, A.; Contaldo, A.; Ierardi, E.; Di Leo, A.; Principi, M. Escherichia coli Nissle 1917 in Ulcerative Colitis Treatment: Systematic Review and Meta-analysis. *J. Gastrointest. Liver* **2020**, *24*, 499-505, doi: 10.15403/jgld.2014.1121.244.ecn.
5. Guo, Q.; Goldenberg, J.Z.; Humphrey, C.; Dib, R.E.; Johnston, B. PROBIOTICS FOR THE PREVENTION OF PEDIATRIC ANTIBIOTIC-ASSOCIATED DIARRHEA. *Gastroenterology* **2019**, *156*, 220, doi: 10.1016/S0016-5085%2819%2937348-2.
6. Blaabjerg, S.; Artzi, D.; Aabenhus, R. Probiotics for the Prevention of Antibiotic-Associated Diarrhea in Outpatients—A Systematic Review and Meta-Analysis. *Antibiotics* **2017**, *6*, 21, doi: 10.3390/antibiotics6040021.

7. Dalal, R.; Mcgee, R.G.; Riordan, S.M.; Webster, A.C. Probiotics for people with hepatic encephalopathy. *Cochrane Db. Syst. Rev.* **2021**, *2021*, doi: 10.1002/14651858.CD008716.pub3.
8. Ford, A.C.; Harris, L.A.; Lacy, B.E.; Quigley, E.M.M.; Moayyedi, P. Systematic review with meta-analysis: the efficacy of prebiotics, probiotics, synbiotics and antibiotics in irritable bowel syndrome. *Aliment. Pharm. Ther.* **2018**, *48*, 1044-1060, doi: 10.1111/apt.15001.
9. Derwa, Y.; Gracie, D.J.; Hamlin, P.J.; Ford, A.C. Systematic review with meta-analysis: the efficacy of probiotics in inflammatory bowel disease. *Aliment. Pharm. Ther.* **2017**, *46*, 389-400, doi: 10.1111/apt.14203.
10. Yeh, T.; Shih, P.; Liu, S.; Lin, C.; Liu, J.; Lei, W.; Lin, C. The influence of prebiotic or probiotic supplementation on antibody titers after influenza vaccination: a systematic review and meta-analysis of randomized controlled trials. *Drug Des. Dev. Ther.* **2018**, *Volume 12*, 217-230, doi: 10.2147/DDDT.S155110.
11. Seiler, C.L.; Kiflen, M.; Stefanolo, J.P.; Bai, J.C.; Bercik, P.; Kelly, C.P.; Verdu, E.F.; Moayyedi, P.; Pinto-Sanchez, M.I. Probiotics for Celiac Disease: A Systematic Review and Meta-Analysis of Randomized Controlled Trials. *Am. J. Gastroenterol.* **2020**, *115*, 1584-1595, doi: 10.14309/ajg.0000000000000749.
12. Wilson, B.; Rossi, M.; Dimidi, E.; Whelan, K. Prebiotics in irritable bowel syndrome and other functional bowel disorders in adults: a systematic review and meta-analysis of randomized controlled trials. *The American Journal of Clinical Nutrition* **2019**, *109*, 1098-1111, doi: 10.1093/ajcn/nqy376.
13. Zhang, J.; Wu, H.M.; Wang, X.; Xie, J.; Li, X.; Ma, J.; Wang, F.; Tang, X. Efficacy of prebiotics and probiotics for functional dyspepsia. *Medicine* **2020**, *99*, e19107, doi: 10.1097/MD.0000000000019107.
14. 14. Sohail, G.; Xu, X.; Christman, M.C.; Tompkins, T.A. Probiotic Medilac-S® for the induction of clinical remission in a Chinese population with ulcerative colitis: A systematic review and meta-analysis. *World Journal of Clinical Cases* **2018**, *6*, 961-984, doi: 10.12998/wjcc.v6.i15.961.
