# Supplementary material for: Gastrointestinal Microbiome and Multiple Health Outcomes: Umbrella Review
Source: Nutrients. 2022 Sep 9;14(18):3726. doi: 10.3390/nu14183726 (PMC9505003; doi:10.3390/nu14183726)
Supplement: Supplementary file 1 [file nutrients-14-03726-s001.zip › Supplementary Table S9.pdf]

Supplement Table S9. Fecal microbiota transplant and multiple health outcomes.

outcome(s) of interest	estimated summary effect (95% CI)	number of studies / total studies	number of intervention group or total participants	Heterogeneity (I^2, %)	the first author + year of publication	Intervention	Duration of Intervention/follow-up	study design	populations	outcome comparison	meta-analysis metric	type of effect model	publication bias
FMT effectiveness (Recovery was defined as the absence of diarrhea between 8 and 13 weeks following treatment)	0.82 (0.75 to 0.89)	15	1168	20.0	POMARES B R 2021[1]	fecal microbiota transplant (FMT)	NP	clinical trials (n=12) and cohort (n=3)	adult human participants with diagnoses of C. difficile diarrhea aged over 19 years old	any versus none	the proportion of treated patients	fixed	0.511
	-0.74 (-0.90 to -0.58)	NP	NP	NP						administration of FMT by colonoscopy versus enema	MD	NP	NP
	0.44 (0.20 to 0.69)	NP	NP	NP						colonoscopy versus capsule			
	-2.28 (-2.63 to -1.93)	NP	NP	NP						colonoscopy versus esophagogastroduodenoscopy			
Clinical cure from rCDI defined as resolution of diarrhea after single FMT infusion.	2.45 (0.78 to 7.71)*	5	79/112	69.0	KHAN M Y 2018[2]	fecal microbiota transplant (FMT)	NP	All RCTs	Patients with documented recurrent recurrent Clostridium difficile infection (CDI)	Fresh FMT compared with intervention (frozen FMT and MT)	OR	random	NP
	0.62 (0.15 to 2.54)*	2	57/63	0.0						frozen FMT using upper versus lower route for diarrhea resolution			
Resolution of diarrhea after multiple fecal microbiota transplantation infusions	1.82 (0.6 to 5.59)*	3	37/43	0.0						Fresh FMT versus rest of the treatments			
Clostridium difficile recurrence	0.18 (0.02 to 2.23)*	3	12/52	82.0						Fresh FMT versus rest of the treatments			
Resolution of diarrhea	2.98 (0.56 to 15.93)*	3	33/52	72.0						single FMT infusion versus medical treatment (MT) only			
Resolution of diarrhea	3.68 (0.74 to 18.22)*	2	7/10	0.0						multiple FMT infusion versus MT only			
not achieving Combined Clinical Remission or Endoscopic	0.80 (0.71 to 0.89)	4	101/140	0.0	NARULA N 2017[3]	fecal microbiota transplantation in	6-12weeks	All RCTs	adult subjects with endoscopically and clinically active UC based on clinical	any versus none	RR	random	NP

Remission/Response with donor FMT						ulcerative colitis(UC)			assessment scores commonly used for UC				
not achieving Clinical remission with donor FMT	0.76(0.62 to 0.93)	4	81/140	31.0									
not achieving endoscopic remission with donor FMT	0.85(0.69 to 1.05)*	4	103/140	77.0									
clinical remission	40.5%(24.7% to 58.7%)	13	141	36.5	SHI Y 2016[4]	fecal microbiota transplantation in ulcerative colitis(UC)	NP	cohort	patients of any age with ulcerative colitis undertaken FMT	any versus none	pooled rates	Random	No serious bias
clinical response	66.1%(43.7%to 83.0%)	11	132	40.2									
combined clinical remission with endoscopic remission/response	0.76(0.65 to 0.89)	3	60/86	0.0	LIU X L 2021[5]	Fecal microbiota transplantation from pooled donor in ulcerative colitis(UC)	NP	All RCTs	patients of any age with ulcerative colitis undertaken FMT	any versus none	RR	Random	NP
	0.79(0.70 to 0.97)	2	45/61	0.0		Fecal microbiota transplantation from single donor in ulcerative colitis(UC)							
	0.79(0.70 to 0.89)	3	85/117	0.0		FMT via the lower gastrointestinal tract							
	0.79(0.58 to 1.09)*	2	20/30	8.0		FMT via the upper gastrointestinal tract							
	0.79(0.69 to 0.90)	3	63/86	0.0		FET with higher frequency							
	0.79(0.65 to 0.96)	2	42/61	0.0		FET with lower							

						frequency							
pooled response ratio after delivery of a single treatment with FMT capsules	85%(82% to 88%)	16	723	0.0	COLD F 2021[6]	treatment with capsule FMT for recurrent C. difficile	At least 8 weeks	RCTs, cohort and cases	adult participants (aged at least 18 years) of encapsulated FMT treatment for recurrent C. difficile	clinical efficacy of encapsulated FMT versus FMT delivered through colonoscopy or enemas	pooled clinical efficacy proportion	Random	0.22
serious adverse events	1.29(0.46 to 3.57)*	4	9/140	0.0	DANG X 2020[7]	fecal microbiota transplantation in ulcerative colitis(UC)	6-12weeks	All RCTs	patients with active UC	any versus none	OR	Fixed	NP
clinical response	2.48(1.18 to 5.21)	4	69/140	52.0									0.508
clinical remission	3.47(1.193to 6.25)	4	53/140	0.0								Random	0.961
Efficacy of Faecal microbiota transplantation in irritable bowel syndrome	0.98(0.58 to 1.66)*	5	267	78.0	IANIROG 2019[8]	fecal microbiota transplantation	NP	All RCTs	adult patients (≥18 years) with IBS	FMT versus placebo	RR	Random	NP
HDL cholesterol	0.09 mmol/L (0.02 to 0.15)	6	80/146	5.0	PROENÇA I M 2020[9]	fecal microbiota transplantation	2 to 6 weeks after intervention	All RCTs	Adults diagnosed with metabolic syndrome by any valid definition (NCEP ATP3 2005, IDF 2006, EGIR 1999, WHO 1999, AACE 2003) and/or obesity—defined as body mass index (BMI) ≥30.	FMT from lean donor(s) versus placebo	MD	fixed	NP
HbA1c	-1.69 mmol/L (-2.81 to -0.05)	6	80/146	46.0								fixed	
LDL cholesterol	0.19 mmol/L (0.05 to 0.34)	6	80/146	0.0								fixed	
Fasting glucose	-0.09 mmol/L (-0.22 to 0.04)*	6	78/144	19.0								fixed	
triglycerides	-0.02 mmol/L (-0.52 to 0.49)*	6	66/120	91.0								Random	
total cholesterol	-1.69 mmol/L (-2.81 to -0.05)	5	69/124	0.0								fixed	
BMI	-0.86 mmol/L (-2.90 to 1.20)*	3	46/78	70.0								Random	
weight	1.67 mmol/L (-9.50 to 12.84)*	4	56/110	86.0								Random	
HOMA-IR	-0.35 mmol/L (-1.56 to 0.86)*	4	59/115	69.0								Random	
clinical remission	1.70(1.22 to 2.56)	6	179/355	45.0	CALDEIRA L D 2020[10]	fecal microbiota transplantation	NP	All RCTs	patients of any age or gender diagnosed with IBD (namely Ulcerative colitis, Crohn's	FMT versus placebo	RR	Random	0.25
clinical response	1.68(1.04 to 2.72)	4	140/277	55.0									

									disease, and Pouchitis				
not achieving antimicrobial resistance (AMR) remission	0.44(0.20 to 0.99)<br>0.37(0.18 to 0.79)	4<br>2	57/111<br>26/56	65.0<br>23.0	TARIQ R 2019[11]	fecal microbiota transplantation	NP	All case series studies with a control arm	adult population (>18 years) With AMR colonization	FMT versus treatment naive	RR	Random	NP

* No statistical significance; CI, confidence interval; RCT, randomized controlled trial; RR, relative risk; HR, hazard ratio; MD, mean difference; SMD, standard mean difference; WMD, weighted mean difference; OR, odds ratio; NA, not available; NP, not published.

References

1. 1. Pomares Bascuñana, R.Á.; Veses, V.; Sheth, C.C. Effectiveness of fecal microbiota transplant for the treatment ofClostridioides difficile diarrhea: a systematic review and meta-analysis. *Lett. Appl. Microbiol.* **2021**, *73*, 149-158, doi: 10.1111/lam.13486.
2. Khan, M.Y.; Dirweesh, A.; Khurshid, T.; Siddiqui, W.J. Comparing fecal microbiota transplantation to standard-of-care treatment for recurrent Clostridium difficile infection: a systematic review and meta-analysis. *Eur. J. Gastroen. Hepat.* **2018**, *30*, 1309-1317, doi: 10.1097/MEG.0000000000001243.
3. Narula, N.; Kassam, Z.; Yuan, Y.; Colombel, J.; Ponsioen, C.; Reinisch, W.; Moayyedi, P. Systematic Review and Meta-analysis. *Inflamm. Bowel Dis.* **2017**, *23*, 1702-1709, doi: 10.1097/MIB.0000000000001228.
4. Shi, Y.; Dong, Y.; Huang, W.; Zhu, D.; Mao, H.; Su, P. Fecal Microbiota Transplantation for Ulcerative Colitis: A Systematic Review and Meta-Analysis. *PLoS One* **2016**, *11*, e157259, doi: 10.1371/journal.pone.0157259.
5. Liu, X.; Li, Y.; Wu, K.; Shi, Y.; Chen, M. Fecal Microbiota Transplantation as Therapy for Treatment of Active Ulcerative Colitis: A Systematic Review and Meta-Analysis. *Gastroent. Res. Pract.* **2021**, *2021*, 1-13, doi: 10.1155/2021/6612970.
6. Cold, F.; Baunwall, S.M.D.; Dahlerup, J.F.; Petersen, A.M.; Hvas, C.L.; Hansen, L.H. Systematic review with meta-analysis: encapsulated faecal microbiota transplantation – evidence for clinical efficacy. *Therapeutic Advances in Gastroenterology* **2021**, *14*, 1320557622, doi: 10.1177/17562848211041004.
7. Dang, X.; Xu, M.; Liu, D.; Zhou, D.; Yang, W. Assessing the efficacy and safety of fecal microbiota transplantation and probiotic VSL#3 for active ulcerative colitis: A systematic review and meta-analysis. *PLoS One* **2020**, *15*, e228846, doi: 10.1371/journal.pone.0228846.
8. Ianiro, G.; Eusebi, L.H.; Black, C.J.; Gasbarrini, A.; Cammarota, G.; Ford, A.C. Systematic review with meta-analysis: efficacy of faecal microbiota transplantation for the treatment of irritable bowel syndrome. *Aliment. Pharm. Ther.* **2019**, *50*, 240-248, doi: 10.1111/apt.15330.
9. Proença, I.M.; Allegretti, J.R.; Bernardo, W.M.; de Moura, D.T.H.; Ponte Neto, A.M.; Matsubayashi, C.O.; Flor, M.M.; Kotinda, A.P.S.T.; de Moura, E.G.H. Fecal microbiota transplantation improves metabolic syndrome parameters: systematic review with meta-analysis based on randomized clinical trials. *Nutr. Res.* **2020**, *83*, 1-14, doi: 10.1016/j.nutres.2020.06.018.

10. Caldeira, L.D.F.; Borba, H.H.; Tonin, F.S.; Wiens, A.; Fernandez-Llimos, F.; Pontarolo, R. Fecal microbiota transplantation in inflammatory bowel disease patients: A systematic review and meta-analysis. *PLoS One* **2020**, *15*, e238910, doi: 10.1371/journal.pone.0238910.
11. Tariq, R.; Furqan, F.; Pardi, D.; Khanna, S. 201 Efficacy of Fecal Microbiota Transplantation for Acute Graft Versus Host Disease in the Gut: A Systematic Review and Meta-Analysis. *Am. J. Gastroenterol.* **2019**, *114*, S123, doi: 10.14309/01.ajg.0000590336.17703.14.
